# Unsupervised clustering of gene expression data points at hypoxia as possible trigger for metabolic syndrome

**DOI:** 10.1186/1471-2164-7-318

**Published:** 2006-12-19

**Authors:** Andrey Ptitsyn, Matthew Hulver, William Cefalu, David York, Steven R Smith

**Affiliations:** 1 Department of Microbiology, Immunology and Pathology, Colorado State University, Fort Collins, CO 80523, USA; 2 Virginia Polytechnic Institute and State University, Department of Human Nutrition, Foods and Exercise Corporate Research Center, Blacksburg, VA 24061, USA; 3 Pennington Biomedical Research Center, 6400 Perkins Rd. Baton Rouge, LA 70808, USA; 4 Center for Advanced Nutrition, Utah State University, 4715 Old Main Hill, Logan, UT 84322, USA

## Abstract

**Background:**

Classification of large volumes of data produced in a microarray experiment allows for the extraction of important clues as to the nature of a disease.

**Results:**

Using multi-dimensional unsupervised FOREL (FORmal ELement) algorithm we have re-analyzed three public datasets of skeletal muscle gene expression in connection with insulin resistance and type 2 diabetes (DM2). Our analysis revealed the major line of variation between expression profiles of normal, insulin resistant, and diabetic skeletal muscle. A cluster of most "metabolically sound" samples occupied one end of this line. The distance along this line coincided with the classic markers of diabetes risk, namely obesity and insulin resistance, but did not follow the accepted clinical diagnosis of DM2 as defined by the presence or absence of hyperglycemia. Genes implicated in this expression pattern are those controlling skeletal muscle fiber type and glycolytic metabolism. Additionally myoglobin and hemoglobin were upregulated and ribosomal genes deregulated in insulin resistant patients.

**Conclusion:**

Our findings are concordant with the changes seen in skeletal muscle with altitude hypoxia. This suggests that hypoxia and shift to glycolytic metabolism may also drive insulin resistance.

## Background

Diabetes affects hundreds of millions world wide, contributing to cardiovascular disease, blindness, amputation, kidney failure and many other diseases. Obesity and impaired insulin sensitivity are among the major factors responsible for development of type 2 diabetes (DM2). Skeletal muscle and white adipose tissue are believed to play a major role in insulin resistance [1,2]. However, long-term studies indicate that even major factors such as insulin resistance are not sufficient to fully predict the onset of disease [3]. Recently a series of papers connected insulin sensitivity and type 2 diabetes to expression of a group of oxidation phosphorylation genes that are co-regulated by the peroxisome proliferator activator protein (PGC-1*α*) [4,5]. These experiments, as well as others [6,7], suggest that mitochondrial dysfunction plays a role in the genesis of DM2 and have fuelled discussions about energy metabolism as a primary factor in insulin resistance.

Microarray expression profiling allows researchers to monitor expression levels of thousands of genes in a single analysis. Classification of samples by such molecular signatures allows for improved stratification of patients, rational application of treatment, and better risk assessment. Importantly, these techniques often uncover previously unanticipated pathways and identify of new targets for therapy. These experimental and computational approaches were first developed and applied in numerous cancer-related research projects [8]. Applying these same techniques to classify skeletal muscle samples of DM2 and non-diabetic patients has encountered very serious problems. When comparing the differences in gene expression of DM2 and non-diabetic patients, the differences are modest, with analytic noise masking the underlying informative changes in gene expression. Two different approaches have been suggested to counter this challenge. Mootha et al. have developed the Gene Set Enrichment Approach (GSEA). In the absence of significantly over- or under-expressed genes, they identified groups of genes to discriminate between DM2 and normal samples based on function, gene ontology (GO) annotation, chromosomal location and other factors. Joining genes from common functional groups is effectively the same as using multiple replicates as it dramatically increases the power of the experiment. Similarly, Patti et al. found no single gene differentially expressed between diabetic and non-diabetic muscle samples after correction for multiple comparisons. They also used extensive functional annotation to identify genes differentially expressed between DM2 and non-diabetic patients. Even though statistical significance of differential expression of these genes was lacking, classification by occurrence of GO terms [9] revealed disparate expression of genes involved in energy metabolism between DM2 and normal. Taken together, both papers implicate genes involved in energy metabolism as the major contributors to DM2 status of the patients. These findings are logical from a biological standpoint and build upon prior data [10,11].

The analytical strategies employed by Mootha et al. and Patti et al. were based on presumption of two distinct categories (DM2 and not DM2) and that these clinical categories should manifest themselves through the gene expression patterns in skeletal muscle. Instinctively, we perceive diabetic and non-diabetic patients in two different categories. However, the onset of diabetes depends on other factors such as lipotoxicity [12-14], a failure of leptin signaling [15], abnormalities in hypothalamic function [16], to name a few. These and many other factors can mitigate the effect of gene expression in skeletal muscle with regards to the onset of diabetes. Taking into account the complexity of the disease, the very existence of distinct categories such as diabetics and non-diabetics when analyzing gene expression data cannot be taken for granted.

The approach described herein does not assign patients' transcriptomes (interrogated by the microarray experiment) to a diagnostic category (DM2 vs. IGT vs. NGT or normal). Instead, we rely on the "natural classification" to identify the groups of samples within the data that are similar to each other by their transcriptome. The concept of natural classification is well established in computer analysis of biological data [17,18]. In the first step of our analysis, our goal was to identify natural categories (clusters) in the expression data. To accomplish this goal we applied a high-dimension unsupervised cluster analysis algorithm developed based on FOREL (see Methods). This algorithm performs a "class discovery" type of clustering [19,20] without pre-selection of a small set of genes to reduce dimensionality. The output consists of a set of finished clusters, which can be further analyzed and hyper-clustered in order to establish the relationship between natural classes. The second step of the analysis is to relate the observations in the data set to the clinical characteristics, and to identify the underlying discriminant genes implicated in formation of specific clusters. Our strategy is based entirely on the observation of similarities within the data and avoids speculative assumptions about gene function. When using a "natural classification" strategy the only assumption being made is that the most common gene expression patterns associated with the development of diabetes are expected to be found many times, providing that a sufficiently large number of samples are included and the microarray technique accurately reflects the underlying molecular mechanisms.

## Results

**Figure 1:**
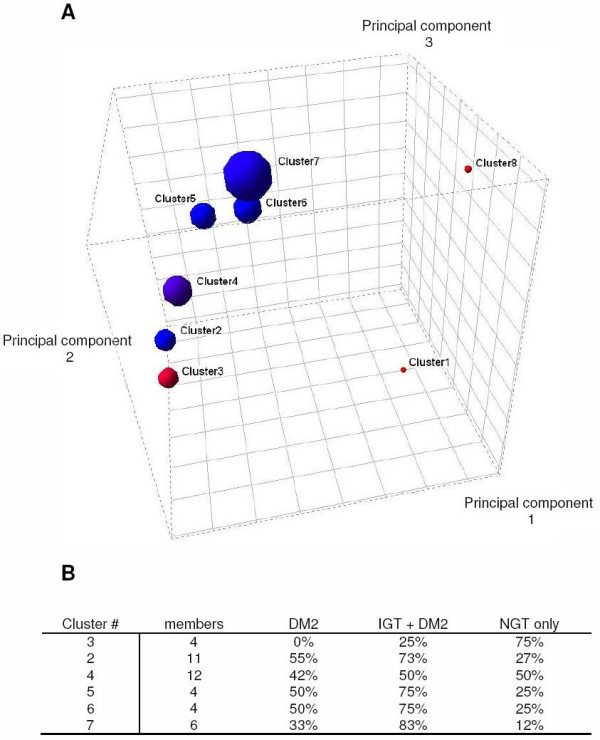
**FOREL Clustering Partially Separates Normal from DM2 and IGT**. **A**. The set of 43 samples (Mootha et al. data set) from the skeletal muscle of normal glucose tolerance (NGT), impaired glucose tolerance (IGT) and diabetic (DM2) breaks into a series of clusters spread along a line in the gene expression space. Each cluster is represented by a sphere with the radius equal to the distance from the centroid to the most distant member of the cluster. The color is proportional to the percentage of diabetic samples in the cluster, ranging from red (100% normal) to blue (DM2). Cluster 8 and cluster 1 represent singletons (i.e. single subjects that do not cluster with the other 41 samples). The percentage of diabetes in each cluster varied along principal component 2. **B**. Analysis of the prevalence of NGT and IGT across clusters revealed that the number of subjects with NGT dropped as distance from Cluster 3 increased. Similarly, the number of subjects with IGT increased as distance from Cluster 3 increased. The two clusters with the greatest distance from the 'normal' cluster 3, Cluster 6 and Cluster 7, 1/2 were diagnosed with DM2 and all other members of these clusters have IGT. Principle Components are presented as the value × 10^4^.

FOREL analysis of the Mootha et al. data set revealed 6 clusters and 2 singletons. No clear-cut categories emerged that were related to the presence or absence of diabetes in these patients. In other words, diabetic and non-diabetic subjects did not form dichotomous groups in the expression space. However, one of the clusters (Cluster #3) was found to contain mostly non-diabetics and one glucose-tolerance impaired (IGT) sample. The centroids of all 4 clusters were stretched along a single line (Figure 1).

**Figure 2:**
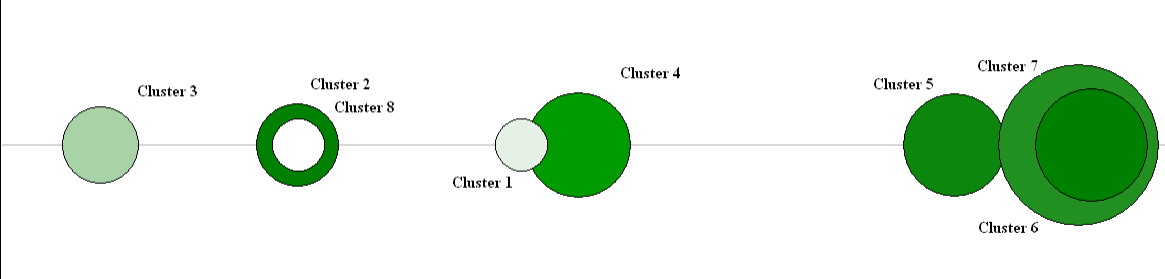
**FOREL Classification Of The Transcriptome Relates To Insulin Sensitivity**. Clusters are graphed along a line connecting the centroids of the clusters in the expression space (see Figure 1). Each cluster is represented by a sphere with the radius equal to the distance from the centroid to the most distant member of the cluster. The color is proportional to the average cluster insulin sensitivity as measured by M-value. The color to the percentage of diabetic samples in the cluster, ranging from white (insulin sensitive) to green (insulin resistant). The M-value for each cluster (Cluster, M-value, units); 3, 10.08; 2, 5.28; 4, 6.41; 5, 5.78; 6, 5.35; 7, 6.25.

To visualize this trend we used the centroids of clusters as control points to reduce the dimensionality down to the number of clusters. The clusters were projected into the space of the first 3 principal components and plotted as 3D spheres with radius equal to the distance between the centroid and the most distant member (subject) of the corresponding cluster. Unsupervised class discovery analysis suggests that the population of samples is highly variable along a single smoothly arched line in the multidimensional expression space. To simplify the presentation of the data, all clusters were graphed along a line connecting the centroids of the clusters in the expression space (Figure 2). There is a modest gradient of the average insulin sensitivity and the position of the cluster along the "main line" of variation. On one end of this line there is a cluster composed of 4 normal samples and one sample with slightly impaired glucose tolerance. The subjects in this cluster are also the most metabolically sound among the data set. For the purpose of this paper, metabolically sound is defined as having they have high insulin sensitivity and an ideal body mass index (BMI).

The juxtaposition and separability of resulting clusters has been analyzed using MANOVA and hierarchical classification of clusters (hyperclustering) based on inter-centroid and inter-class distances. The results of this analysis are presented in the supplementary materials (Additional file 1, Supplementary Figures 1–4). Considering visualization and statistic analysis we suggest the following interpretation: the data set represents one core cluster of the most metabolically sound samples (cluster 3 on Figure 1) and a continuum of samples extended in one direction, like a head and a tail of a comet. The imaginary line stretched between the core and the most distant FOREL cluster represents the major trend in gene expression space. Although there is no clear separation between diabetic and non-diabetic samples as two categories, the distance along this main line of variation coincides with the occurrence of diabetes and severity of the common risk factors.

### Differences in gene expression between the FOREL clusters

Next, we identified genes that were most differentially expressed between cluster 3 (metabolically healthy) and cluster 6 (high prevalence of DM2, low insulin sensitivity). To select such genes we applied caGEDA tools [21] on unabbreviated list of genes (see Methods). We found that clusters occupying the extremes ends of the line connecting the centroids varied with respect to the expression of genes encoding ribosomal proteins. Other significant function categories included actin, myosin, hemo- and myoglobins, enzymes involved in glucose and fructose metabolism, lipid metabolism and genes encoding proteins involved in oxidative phosphorylation (see Additional file 2 in supporting materials [22]).

We also performed functional annotation of these same genes at the extremes of clusters 3 and 6 using EASE [23]. We found several categories of genes that were significantly overrepresented as compared to their expected occurrence (see Additional file 3 in supporting materials [22]). The most prominent functional categories included ribosomal genes, genes involved in skeletal muscle contraction, actin, cytoskeleton and protein biosynthesis.

**Figure 3:**
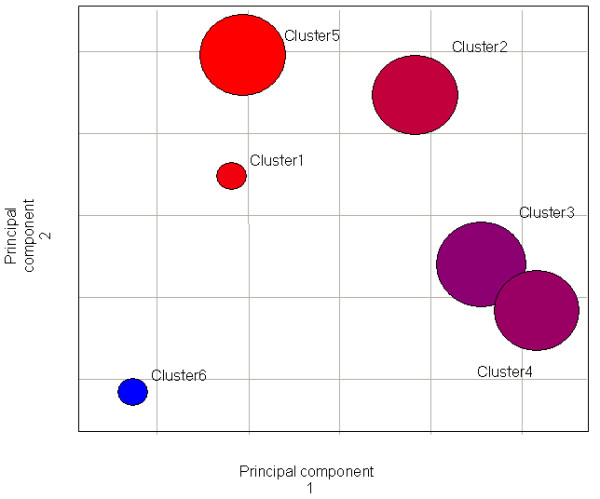
**Results of clustering of 24 samples of skeletal muscle from patients with various degrees of obesity (Hulver at al.)**. The data breaks into 4 clusters and 2 singletons. Color indicates the prevalence of average BMI for the cluster (Cluster#-BMI): 1–25, 2–31.6, 3–40.9, 4–38.4, 5–22.0, 6–62.0.

The dataset produced at the East Carolina University [24] has a different purpose, it is focused on skeletal muscle fatty acid metabolism in connection with obesity, but utilized the Affymetrix GeneChip (U133). This chip is different from those used in experiments of Mootha et al. (U95) and Patti et al. (HS6800). Given that obesity and insulin resistance are clearly linked [25,26] it is interesting to compare this study with our re-analyses of the Mootha et al. dataset. The 24 samples were collected from 3 groups of patients with normal weight (BMI 23.8 ± 0.58), obese (BMI 30.9 ± 0.81) and morbidly obese (BMI 53.8 ± 3.5). Surprisingly, the FOREL clustering results from the Hulver dataset presented on Figure 3 are highly similar to the clustering results from the Mootha et al. dataset. Four large clusters, produced by FOREL analysis form a line in the gene expression space, one end of the line is occupied by a cluster composed almost entirely by the most metabolically sound specimens, with the opposite cluster again containing the least sound samples. Analysis of the genes most differentially expressed between the most distant clusters along this line reveals a remarkably similar list of genes to the first analysis (see Additional file 5 in supporting materials [22]).

The other data set used in this study (Patti et al.) is much smaller; it contains 15 samples of which 10 represent normal and 5 represent DM2. Again using the FOREL clustering process, we found that all samples fell into 4 clusters. The largest cluster (#4) contains 5 samples, of which 3 are DM2, Cluster #2 is entirely composed of samples from normoglycemic subjects, clusters #1 and #3 include mostly normoglycemic subjects but each includes one DM2 subject. It is reasonable to suppose that clusters #2 and #4 represent the most contrast groups of samples in the data and the line that stretches between centroids of these clusters would be analogous to the trend we observe in the Mootha et al. data set. The results of our re-analysis of the Patti et al. data can be found in supporting materials [22] (see Additional file 4, supplementary figure 7 in Additional file 1).

When comparing these three studies, the samples are absolutely independent and differ in many ways (age, gender, type of microarray used, feature extraction algorithm etc.), yet the genes that are most differentially expressed between the most and least metabolically sound are again remarkably similar to those found in our re-analyses of the Mootha and Hulver datasets. (see supporting materials [22], Additional files 2, 3, 4, 5) These discriminant genes include GAPDH, myoglobins, titin, FHL1 and a score of ribosomal proteins. Although genes involved in oxidative phosphorylation are among those differentially expressed in all three datasets, they are not at the top of the list when ranked by the fold change between clusters. Thus these analyses identified common genes and functional categories that were unanticipated based on the earlier analyses.

After these efforts to cluster the subjects, we next clustered the genes independent of the subjects. Cluster analysis of the genes throughout the data set revealed a number of highly correlated clusters. Again using the FOREL technique with the Mootha dataset, we found several interesting clusters of genes. Based on a functional annotation analysis [23] of these genes, we found that one of the clusters contains a group of genes related to lipid transport and metabolism. However, the largest cluster includes 221 genes and among these genes are the same ribosomal, glucose and fructose metabolism genes that discriminated between the most and least metabolically sound subjects.

### Natural classification of subjects based on their transcriptome separates subjects into categories recognizable by their clinical characteristics

**Figure 4:**
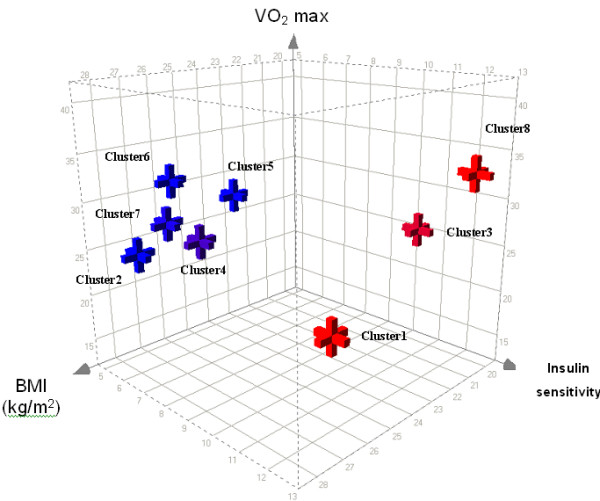
**A Three-dimensional plot of the FOREL clusters from Mootha et al. data set in relationship to Body Mass Index (BMI), total oxygen capacity (VO2max) and insulin sensitivity (M-value)**. Members of cluster 3, occupying the extreme end of the variation spectrum and two singletons (both non-diabetic) are the most insulin sensitive. Some non-diabetic phenotypes are found in clusters characterized by high risk factors (↓M-value, ↑BMI) particularly cluster 4. Conversely, there is only one subject with IGT in cluster 3. On this plot clusters are represented by corresponding centroids.

We next asked whether the natural classification of the molecular phenotypes using the FOREL analysis corresponded to the clinical phenotype as measured by BMI, insulin sensitivity, etc. The clinical data, provided by Mootha et al. was used to compare the 'metabolic soundness' of the patients across cluster membership from the FOREL analysis (Figure 4). Patients from clusters 2, 5, 6 and 7 have a higher body mass index (BMI) and lower insulin sensitivity (M-value) when compared to the 'normal' cluster. This visual analysis was confirmed by ANOVA (supplementary data, supplementary Figures 1–4). From this and the other results (Figures 1 and 2) the subjects represented in cluster 3 are in exceptional physical form for their age. They form a tight group with a normal BMI and normal insulin sensitivity. Interestingly, maximal aerobic capacity (VO_2_max) does not clearly discriminate this group from the least metabolically sound cluster with a high proportion of DM2. The two singletons, both NGT have either normal (#8) or almost normal (#1) BMI and high insulin sensitivity (M-value). A few NGT samples are also found in clusters 2, 4, 5, even less are found in clusters 6, and 7 that are dominated by DM2 and IGT samples. Thus in spite of the high BMI and low insulin sensitivity these individuals manage to remain normo-glycemic. This is consistent with the natural history of DM2 where beta cell failure is a late event with a variable onset but invariably preceded by insulin resistance. We speculate that the metabolically unsound individuals are at risk for the development of DM2 as beta cell apoptosis ensues and islet neogenesis fails to compensate [27]. These 'natural' clusters coincided with the classic markers of diabetes risk, namely obesity and insulin resistance, but did not follow the accepted clinical diagnosis of DM2 as defined by the presence or absence of hyperglycemia. Unfortunately, there are other important parameters missing in all three data sets, such as time of onset of hyperglycemia and estimation of physical activity. Since all samples were collected from the patients of general hospitals we can speculate that the level of physical activity and its distribution among sample donors is typical for the urban population of US East Coast and on average not very high. It is possible that molecular mechanisms of insulin resistance discussed below are significantly influenced by the level of patients' physical activity.

## Discussion

Type 2 Diabetes (DM2) is a complex multifactorial disease where dozens or perhaps even hundreds of different genetic and environmental factors play a role. It is likely that there is more than one reason for a patient to develop hyperglycemia (i.e. beta cell failure), although insulin resistance is a dominant factor. Similarly, it is likely that genetic and environmental factors play a role in the development of insulin resistance, a metabolic feature that invariably precedes beta cell failure. Moreover, DM2 is not a disorder caused by malfunction of a single tissue. Skeletal muscle plays very important, but not exclusive role along with pancreas, adipose tissue, etc. DM2 is diagnosed on the level of the whole organism as increased blood sugar [28]. Thus, there is no reason to believe that muscle samples from a single tissue would contain just one single gene expression signature associated with the onset of the DM2. Classification models based on only two classes separated by DM2 diagnosis may be at a disadvantage as they have limited separation performance. Our initial motivation for application of unsupervised clustering algorithms was based on the hypothesis that DM2 samples as well as normal NGT controls could possibly be subdivided into many classes with more distinctive gene expression signatures than those revealed by the original analysis. Indeed this was the case. We found that individuals could be separated based on their gene expression profiles along a single continuum.

### Relationship of the FOREL classifications with clinical characteristics

In the next step of analysis we superimposed the clusters in the space of first principal component. For all three datasets, the centroids of the clusters were situated along a single line or plane. Clusters with the most metabolically sound subjects with NGT and the least sound subjects (with DM2) occupy the extreme ends of this line, while most IGT samples are found in the mixed clusters between the extremes. In the Mootha dataset, the healthiest group is represented by a single cluster, which has 3 non-diabetic and 1 IGT subjects. On the opposite end, two dominant clusters contain only one non-diabetic sample each, while all other members are either diabetic or insulin resistant. This picture suggests a simple interpretation: instead of categorical classes of normal and diabetic samples we observe a continuous trend. On one end of the trend are the samples from the most metabolically sound individuals with a low prevalence of DM2. Prevalence of DM2 increases along the line, but can be mitigated by a number of factors. Many of these factors are beyond the scope of this research and probably relate to *β*-cell apoptosis and/or failure of beta cell neogenesis/replication [27,29,30]. As expected, unsupervised clustering revealed a few distinct classes. However, we did not anticipate that these clusters would be aligned along a single principal component. Rather than multiple 'subtypes' these analyses suggest a single underlying disorder. Importantly, these natural classes differ in average clinical characteristics and in the number of diabetic and non-diabetic members.

### Discriminant genes

Once the direction of the trend is established it is relatively easy to select and annotate the genes that are most differentially expressed between the extremities of the trend. The list of such genes does include those involved in the energy metabolism and oxidative phosphorylation, as reported by Mootha et al. and Patti et al. The expression level of such genes changes dramatically between clusters on the extreme ends of the trend. Oxidative phosphorylation genes possibly play an important role in the etiology of DM2 although this role is not decisive and is insufficient for discrimination between classes. This also explains the difficulties Mootha et al. had selecting the genes that discriminate between DM2 and NGT samples.

These discriminant genes fall into several interesting gene ontogeny categories. First, we observed an increase in expression of genes encoding proteins required for glycolytic rather than oxidative metabolism such as GAPDH. This is consistent with prior data demonstrating a reduction in oxidative capacity of skeletal muscle from insulin resistant diabetics [31].

Second, we discovered that myoglobin, lactate dehydrogenase A, GAPDH, aldolase A, and pyruvate kinase, genes that are classically upregulated when oxygen tension falls [32] were upregulated in the least metabolically sound samples. Human skeletal muscle adaptation to altitude hypoxia has been well characterized and includes a seemingly paradoxical reduction in oxidative capacity, an increase in myoglobin, and a shift to glycolytic metabolism [32]. All of these are dominant features of the gene expression data reported herein. Altitude hypoxia reduces the overall mitochondrial volume by 20%, but with a preferential reduction in sub-sarcolemmal mitochondria (-43%) [33] compared to a modest change in the intra-myofibrilar mitochondria (-13%), a finding strikingly similar to the mitochondrial defects observed in type 2 diabetes [6]. Reduced oxidative enzymes are also observed in S. Cerevisiae as hypoxia ensues [34]. Hypoxia/anoxia also shifts substrate metabolism from aerobic to glycolytic in diverse species from S. Cerevisiae to man [32,35]. Multiple transcription factors are involved in the transcriptional response to hypoxia in S. Cerevisiae including Rox1 [34], Hap1–4, Sut1, UPC2, and Mox 1–3, (reviewed in [35-37]) and in man HIF-1a [38]. The role of these transcription factors in DM2 is unknown and warrants exploration. If hypoxia is involved in the observed molecular and cellular phenotype it does not cause a compensatory effect on the capillary density. The capillary density and maximum oxygen capacity (VO2max) as reported in the supplementary materials (Supplementary Table 6) by Mootha et al. shows no significant difference across FOREL clusters. This again, is consistent with the literature on structural adaptations to hypoxia where no change in capillary density are seen unless intense exercise was employed as part of the experimental paradigm [33,39,40]. This finding does not necessarily present a contradiction: a shift from oxidative to glycolytic pathway metabolism in skeletal muscle tissue may reduce demand for oxygen as an alternative to capillary growth. Alternately, insulin is capable of activating the bundle of genes that are similar to those activated by HIF-1a. In vivo, insulin upregulates aldolase A, and GAPDH, both of which are classic targets of HIF-1a [38,41]. There is some controversy whether these effects are dependent on changes HIF-1 mRNA [42], occur through the stabilization of HIF-1a protein or through an unknown HIF response element binding protein [43]. It is established, however, that insulin upregulation of these genes requires phosphoinositol 3-kinase [42,44]. In summary, the unsupervised FOREL analysis identified upregulation of a cassette of genes strikingly similar to hypoxia inducible genes in the obese, insulin resistant state.

Lastly, we discovered a set of genes encoding ribosomal proteins separated metabolically sound from unsound subjects with DM2 and IGT. On the surface, this is an anomalous finding. However, microarray studies of petite, mitochondria deficient (*ρ*-) yeast data demonstrate an upregulation of genes encoding ribosomal proteins as compared to mitochondria replete (*ρ*+) yeast [45]. Similarly, as yeast become anoxic and shift from an oxidative to ethanol producing metabolism (the diauxic shift) many of these same genes encoding ribosomal proteins are up/downregulated. This is congruous with prior data showing a downregulation of oxidative phosphorylation genes and disrupted mitochondria in DM2 [31]. An alternate explanation for the increased expression of ribosomal and other genes associated with cell growth reflects alterations upstream of the mammalian Target of Rapamycin (mTOR) pathway. mTOR is a major regulator of metabolic processes in cell. It has been reported to stimulate ribosomal biogenesis and repress nutrient turnover [46,47]. Prior studies have implicated mTOR in nutrient sensing and diabetes [46,48]. Acute insulin infusion upregulates the expression of many ribosomal genes [49], providing an alternate pathway for the observed changes in ribosomal gene expression.

In contrast to the subtle changes in gene expression of the oxidative phosphorylation genes identified by Mootha et al., we found several genes that discriminanted lean healthy people and individuals with DM2 or IGT. For example, Aldolase A was 3.3 times overexpressed in obesity/insulin resistance and GAPDH 4 times higher. Similarly, hemoglobin was upregulated in obesity/insulin resistance; by 13, 12.1 and 11.8 fold for subunits alpha 2, beta and alpha 1 respectively. The novel transcription factor FHL1 was ~3.5 fold upregulated. The second and third data sets (Hulver et al. and Patti et al.) although limited by sample size and without complete clinical phenotypes, produced almost identical cluster layout with a single group of the most metabolically sound subjects and several mixed groups with differing prevalence of DM2. Similarly, the genes most differentially expressed between the two most and least metabolically sound clusters were remarkably similar across the three datasets. This suggests all three datasets identified similar underlying pathophysiological mechanisms leading to DM2.

Further study will be required to determine which factor plays the primary role, whether impaired oxidative phosphorylation causes a shift to glycolytic metabolism or oxidative phosphorylation is repressed as a result of unknown upstream transcriptional events [50]. Regardless, the observed upregulation of ribosomal gene expression might be a marker of the risk to develop type 2 diabetes.

## Conclusion

In summary, using a novel unsupervised clustering technique, FOREL, we found a single set of genes in skeletal muscle that separate subjects that are metabolically sound from those with insulin resistance, IGT and diabetes. This finding was confirmed in two additional datasets from different populations and suggests a single underlying pathophysiological process lies underneath the clinical diagnosis of insulin resistance. The genes that discriminate between metabolically sound and metabolically unsound patients include the anticipated gene categories of fat oxidation, oxidative phosphorylation, and glycolytic metabolism. We also found several categories of genes not previously associated with insulin resistance or diabetes, namely, genes involved in ribosomal function and oxygen sensing (myoglobin and hemoglobin). A separate analysis, where we identified clusters of genes that were co-regulated within the datasets, showed a large degree of overlap. Based on prior literature in energy metabolism and mitochondrial dysfunction in Saccharomyces cerevisiae, these results suggest that hypoxia, changes in ribosomal function, and glycolytic metabolism as key players in insulin resistance and diabetes.

## Methods

### Data sets

We downloaded the data published by Mootha et al [5], Patti et al [4] and Hulver et al. [24]. The Mootha et al. data set consists of 43 age-matched samples representing 17 normal, 8 with impaired glucose tolerance and 18 with DM2. Affymetrix U133 biochips were used to measure expression of over 22,000 genes. This data, along with the clinical information, was downloaded from the author's data portal at the Broad Institute [51]. The other data set [22] consists of 10 normal non-diabetic skeletal muscle tissue samples and 5 samples from the patients diagnosed with DM2. Five replicate microarrays are present in this dataset. In our analysis these samples are always found in one cluster close to each other. We have excluded these samples from the final data set.

### Clustering algorithm

As a starting point for the algorithm development we took the heuristic concept proposed by Zagoruiko et al. [52,53], which included the original idea of a limited radius hypersphere, moving stepwise to the mass center of captured objects. This idea represents a departure from widely used k-means and other hypersphere-based algorithms. This algorithm has been also extensively discussed in a reference book for applied statistics in economics [54]. Since their first introduction algorithms of the FOREL family have been widely applied in taxonomic analysis of biomedical data, pattern recognition in geology and image analysis [55-58]. The original algorithm underwent a significant development the Pennington Center to accommodate for extreme dimensionality of gene expression data. The results of this development and case study in classification of molecular subtypes of lung cancer has been recently published [22]. Our algorithm is based on dynamic amalgamation of objects (for example, expression profiles) in vicinity of an artificially introduced object (FORmal ELement). The vicinity is defined by equal distance from a point in all directions by selected inter-object distance metric (such as Euclidean, correlation, binary, etc.). Although theoretically the vicinity could be defined as any geometrical shape around the given point, only hyperspheric vicinity has been implemented and used in this study. FOREL clustering is based on the perception of the data set *O *as:

O=∪i=1kO(i);
 MathType@MTEF@5@5@+=feaafiart1ev1aaatCvAUfKttLearuWrP9MDH5MBPbIqV92AaeXatLxBI9gBaebbnrfifHhDYfgasaacH8akY=wiFfYdH8Gipec8Eeeu0xXdbba9frFj0=OqFfea0dXdd9vqai=hGuQ8kuc9pgc9s8qqaq=dirpe0xb9q8qiLsFr0=vr0=vr0dc8meaabaqaciaacaGaaeqabaqabeGadaaakeaacqWGpbWtcqGH9aqpdaWeWbqaaiabd+eapjabcIcaOiabdMgaPjabcMcaPiabcUda7aWcbaGaemyAaKMaeyypa0JaeGymaedabaGaem4AaSganiablQIivbaaaa@3A8E@

where *O*(*i*) is a cluster of n_*i *_elements. Clusters are extracted from the general population in order of their statistical fitness (see Cluster validation). This perception is fundamentally different from the popular *k-means *algorithm, which shares certain similarity with FOREL, but in k-means concept the whole data set is a sum of distinct classes rather then a union. FOREL clusters can partially or completely overlap in space or even share the same centroid, but can be separated as long as they differ in other statistical characteristics, for example density. In a nutshell, a white and a yolk of an egg would be separate classes by FOREL, while inseparable by *k-means*. Other hypersphere-based algorithms such as k-means imply Gaussian distribution of objects (phenotypes) in clusters [59]. FOREL is more flexible and does not require such assumptions. FOREL effectively combines the best features of k-means and hierarchical clustering approaches for the price of increased computation complexity. However, the performance of our C++ implementation is acceptable; up to a few hundreds of microarrays can be clustered on a Pentium IV PC within one hour. The algorithm starts with positioning of a hypersphere with a radius *R*_0 _and a center C_0 _in a certain coordinate, which can be one of the objects or a centroid of pre-defined cluster or any other point of interest in the expression space. Position of the "formal element" element is calculated as a center of mass of all objects, for which the distance *ρ*_*i*_(*C*_*i*_) ≤ *R*_*i*_. After the mass center of all captured objects is calculated, its center is moved to the new position. If new objects are found within the radius from the new position, they are added to the provisional cluster and the mass center is recalculated. This process is repeated until the no more objects can be added on the current step of the algorithm and the hypersphere stops.

### Cluster validation

The version of FOREL developed at Pennington Biomedical Research Center (PBRC) consists of alternating steps of cluster isolation and cluster validation. Each completed walks of a hypersphere with *R*_*n *_and a center C_*m *_produces a provisional cluster *O*(*R*_*n*_*C*_*m*_), which is temporarily stored. We perform an exhaustive coverage of the data trying each element of the original data set as potential starting point. For each starting point we perform series of clustering steps with different hypersphere radius, ranging between minimum *R *= *min*(*D*(*C_i_*, *C_j_*)) + *μ *and maximum *R *= *min*(*D*(*C_i_*, *C_j_*)) - *μ*. Here *D*(*C_i_*, *C_j_*) is a distance (for example, Euclidean) between any two objects in the data set and *μ *is a margin, introduced to reduce computation time. The step of *R *increment is also a parameter. The resulting provisional clusters are fuzzy subsets, each captured by a hypersphere with specific radius as it moves gradually from the starting to the resting point. The validity of the provisional cluster can be verified by a statistical utility measure based on density, variance, sum of inter-cluster distances, etc (see [60] for review). If the cluster meets the selection criteria, it is removed from the original data set and the process reiterated until no more statistically valid (according to the chosen metric) unclassified objects left or the best provisional cluster does not satisfy the minimal fitness requirement. The PBRC implementation accommodates a few different metrics for computational cluster validation, but only one density-based metric has been applied in this study:

F=1σi2
 MathType@MTEF@5@5@+=feaafiart1ev1aaatCvAUfKttLearuWrP9MDH5MBPbIqV92AaeXatLxBI9gBaebbnrfifHhDYfgasaacH8akY=wiFfYdH8Gipec8Eeeu0xXdbba9frFj0=OqFfea0dXdd9vqai=hGuQ8kuc9pgc9s8qqaq=dirpe0xb9q8qiLsFr0=vr0=vr0dc8meaabaqaciaacaGaaeqabaqabeGadaaakeaacqWGgbGrcqGH9aqpdaWccaqaaiabigdaXaqaaGGaciab=n8aZnaaDaaaleaacqWGPbqAaeaacqaIYaGmaaaaaaaa@340D@ if *n*_*i *_≥ 2 and *F *= 0 otherwise.

This metric is a reasonable compromise between precision and performance, which has proven to be effective in analysis of microarray data [22]. The "brute force" approach to computational cluster validation provides more reliable results compared to re-sampling, but results in considerably longer execution times. Depending on the validation metric applied and the parameter settings complete clustering of a large data set, such as Mootha et al. data can take up to a few hours on a typical computer. The demand for computational power is significantly mitigated by effective C++ implementation and generally affordable, considering the time required to collect such data.

### Software implementation

The implementation developed by A. Ptitsyn [22] and available for anonymous download [61] employs a complete test of every object as a possible cluster seed or hypersphere starting point. By default the current version of the program implements an iterative solution: all possible radii are tested with a certain step within a limited range. The step (or precision) is derived from the analysis of variation of distances within the whole data set. The range is defined by the minimal and maximal distance found within the whole dataset. These are extreme values, with a radius less then a minimal distance, the algorithms can produce only singletons, and on the other hand with radius equal to the maximal distance, all objects are guaranteed to fall into one large cluster. The best radius is one that produces a provisional cluster with the best quality. Cutting percentile margins from the possible radius range can reduce the computation demands of the program. By default 20% of the range is cut from both minimal and maximal radius values.

The implementation of FOREL clustering algorithm made at the PBRC runs on the standard PC under MS Windows (win32 console application) or Linux. FOREL execution time is practically unaffected by the dimensionality of the data, but can be sensitive to the number of objects (samples) in the set. Large data sets of over 1000 microarrays would require a high-performance computer.

### Identification of informative genes

We have selected genes differentially expressed between most distant clusters using J5 algorithm [21] with Jackknife option set to 4. We used the software implemented at the University of Pittsburg Cancer Institute with access through the Web interface [62].

### Functional Annotation and Interpretation of the results

The consequent hyper-cluster analysis was performed in order to reveal relations between clusters and their juxtaposition in the gene expression space. Centroids of the original FOREL clusters have been used to produce a hierarchical classification (Euclidean distance, UPGMA) with the help of Spotfire Decisionsite for Molecuar Genetics (Spotfire Inc.). Functional annotation of the discriminating genes has been performed with EASE and DAVID tools [23]. Only a small portion of the results can be included within this publication. The additional supplementary materials can be found at [22].

## Authors' contributions

AP has developed and implemented the algorithm, analyzed the data. SRS and AP have interpreted the data and wrote the paper. MH contributed to interpretation of the data and writing paper. WC and DY contributed to data interpretation.

## Supplementary Material

Additional file 1 **Supplementary figures**. This PDF file contains supplementary figures with additional comments and method description, illustrating different detail of analysis, results and interpretation. Click here for file

Additional file 2 **Supplementary Table 1**. This PDF file contains the table of genes, found differentially expressed between the most and the least metabolically sound clusters in our analysis of Mootha et al. data set. Click here for file

Additional file 3 **Supplementary Table 2**. This PDF file contains the table of Gene Ontology categories overrepresented among genes differentially expressed between the most and the least metabolically sound clusters in our analysis of Mootha et al. data set. Click here for file

Additional file 5 **Supplementary Table 4**. This PDF file contains the table of genes, found differentially expressed between the most and the least metabolically sound clusters in our analysis of Hulver et al. data set. Click here for file

Additional file 4 **Supplementary Table 3**. This PDF file contains the table of genes, found differentially expressed between the most and the least metabolically sound clusters in our analysis of Patti et al. data set. Click here for file

Additional file 6 **Supplementary Table 5**. This PDF file contains the table of genes, forming the largest cluster in FOREL clustering of genes in Mootha et al. data set. Click here for file

Additional file 7 **Supplementary Table 6**. This MS Excel file contains clinical data for the patients and samples collected in Mootha et al. study. The file is almost unchanged from the supplementary data provided by Mootha et al. except one added column designating FOREL cluster numbers. Click here for file
