# Supplementary material for: Unsupervised clustering of gene expression data points at hypoxia as possible trigger for metabolic syndrome
Source: BMC Genomics. 2006 Dec 19;7:318. doi: 10.1186/1471-2164-7-318 (PMC1770922; doi:10.1186/1471-2164-7-318)
Supplement: Additional file 1 — Supplementary figures. This PDF file contains supplementary figures with additional comments and method description, illustrating different detail of analysis, results and interpretation. [file 1471-2164-7-318-S1.pdf]

Interpretation of FOREL clusters

Inter-cluster relations analyzed with MEGA2 software:
Kumar S, Tamura K, Jakobsen IB, Nei M. MEGA2: molecular evolutionary genetics analysis software. Bioinformatics. 2001 Dec;17(12):1244-5.

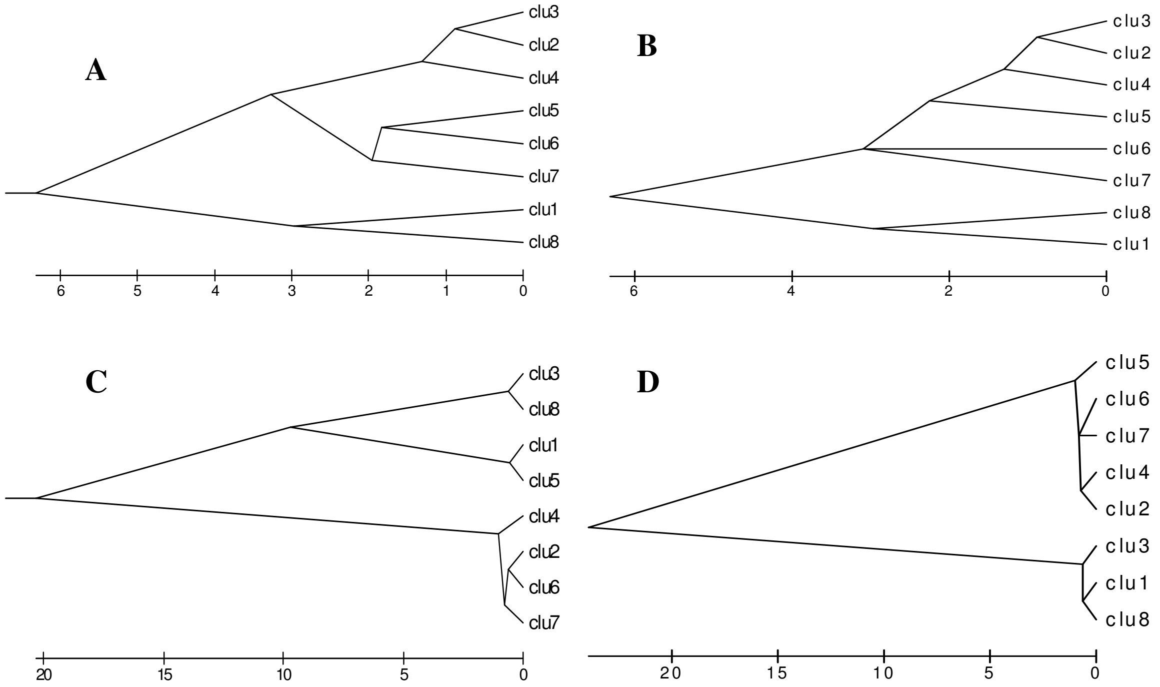

Supplementary Figure 1. Alternative analysis of cluster centroid juxtaposition with MEGA2 software. **A**: UPGMA tree based on Euclidean distance between cluster centroids; **B**: Neighbor-joining tree based on inter-centroid distance matrix; **C**: UPGMA tree based on average Euclidean distance between cluster elements; **D**: Neighbor-joining tree based on average Euclidean distance between cluster elements. All alternative analyses of relative positions of cluster centroids are consistent with the results of 2-step dimensionality reduction visualization presented in the paper. Clusters 1 and 8 are singletons represented normal samples, closely associated with each other and/or cluster 3. Cluster 3 is a dense group of very similar "most metabolically fit" samples. All other clusters contain a mixture of normal, impaired or diabetic samples situated approximately along one line stretching between cluster 3 and cluster 7 (least metabolically fit cluster).

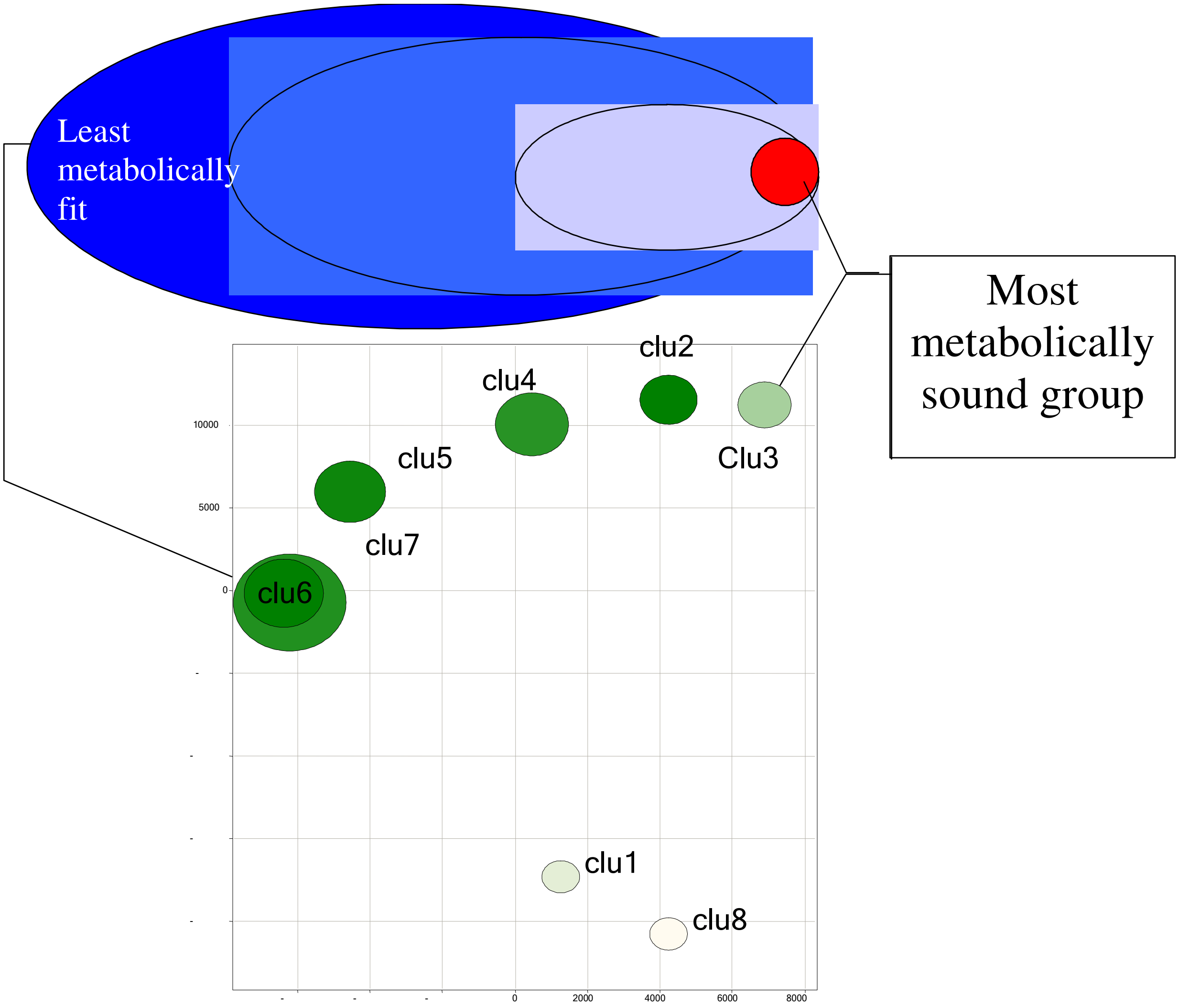

Supplementary figure 2. FOREL clusters are numbered in order they are extracted from the initial data set. The order is important for interpretation of the clustering results because it follows the cluster fitness metric. The best clusters are extracted first, after each extraction clustering is repeated. The process is stopped when all data is clustered or when the best possible cluster has fitness metric below selected threshold. This allows separation of clusters partially or completely overlapping in space. For example, clusters 6 and 7 (the last and the worst among non-singleton clusters) are sharing the same space. However, they are listed separately because union of these two clusters when considered as a provisional cluster in absence of previously extracted clusters 2, 3, 4 and 5 (see algorithm pseudocode below) was rated lower than cluster 6. In a nutshell, FOREL can separate 2 or more nuts in a way similar to k-means, but unlike k-means a nut, a shell and a worm can also be considered separate objects in FOREL classification. Following analysis of cluster fitness and position in relation to other clusters (i.e. hyper-clustering) can identify patterns and relations, which might be very hard to detect by other methods currently widely applied in gene expression analysis.

Insulin sensitivity

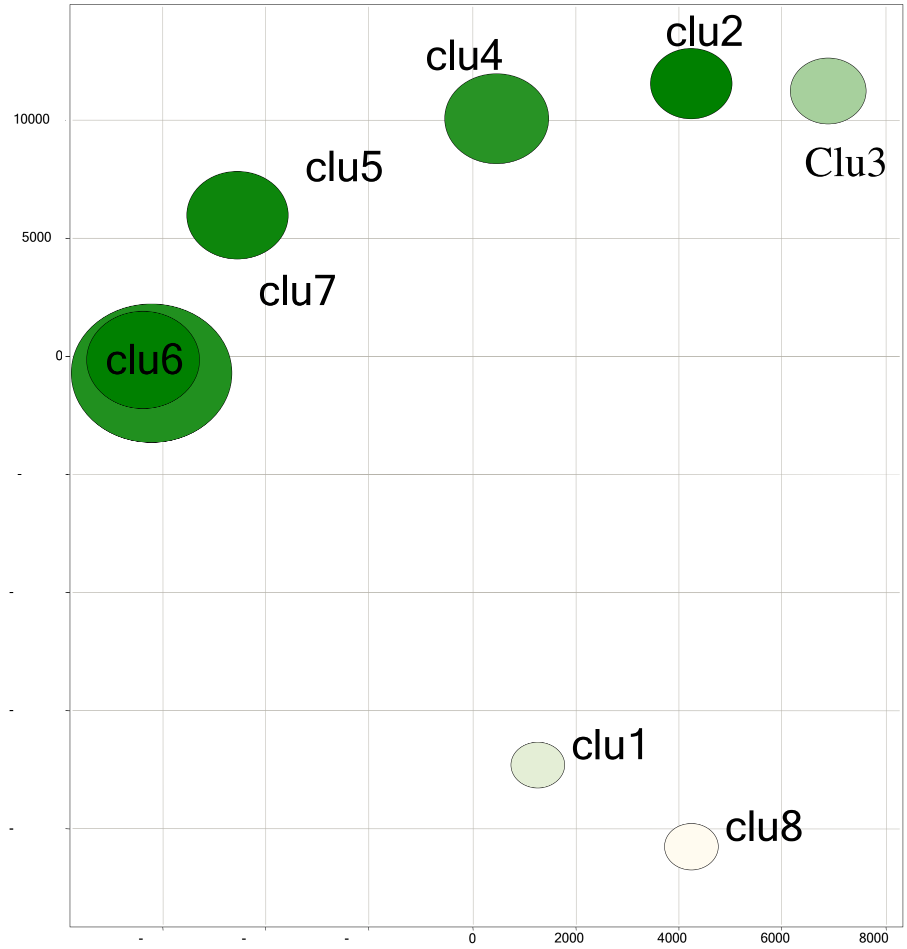

Yellow-marked differences are significant at p < .05, blue-marked at p<0.1									
		{1}	{2}	{3}	{4}	{5}	{6}	{7}	{8}
		M=25.680	M=28.271	M=20.370	M=26.197	M=24.440	M=26.378	M=25.997	M=20.130
G_1:1	{1}		0.530282	0.231237	0.899369	0.77763	0.873732	0.940459	0.320927
G_2:2	{2	0.530282		0.00161	0.222217	0.1057	0.4171	0.26622	0.054423
G_3:3	{3	0.231237	0.00161		0.014022	0.148797	0.03623	0.031932	0.956385
G_4:4	{4	0.899369	0.222217	0.014022		0.440248	0.936399	0.918832	0.14388
G_5:5	{5	0.77763	0.1057	0.148797	0.440248		0.486679	0.540068	0.329443
G_6:6	{6	0.873732	0.4171	0.03623	0.936399	0.486679		0.880534	0.160646
G_7:7	{7	0.940459	0.26622	0.031932	0.918832	0.540068	0.880534		0.172346
G_8:8	{8	0.320927	0.054423	0.956385	0.14388	0.329443	0.160646	0.172346	

Supplementary Figure 3. Relative position and radii of the clusters in connection with insulin sensitivity (M-value). Cluster 3 and singletons 1 and 8 demonstrate normal sensitivity, other clusters contain mixed samples. Clusters 6 and 7 are predominantly diabetic and insulin resistant. The table below the plot area contains the results of MANOVA.

BMI

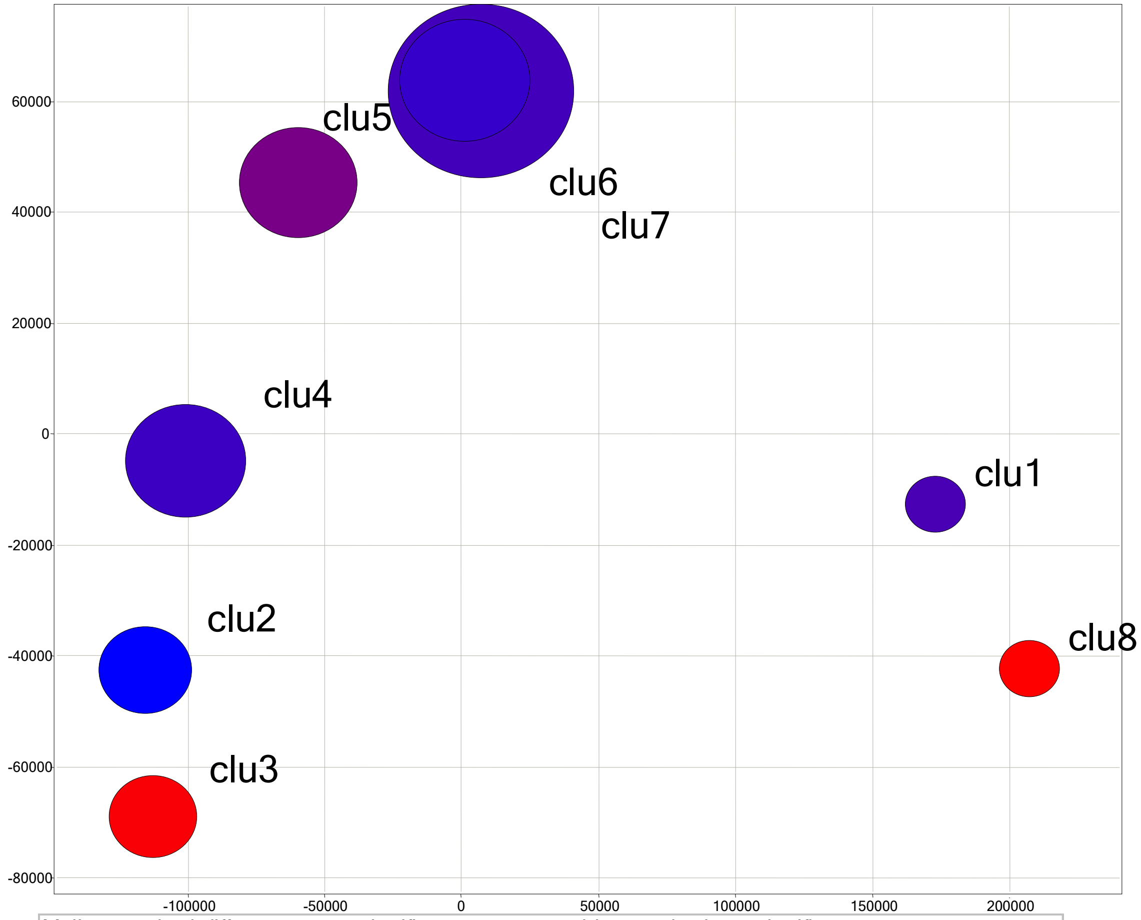

Yellow-marked differences are significant at p < .05, blue-marked are significant at p<0.1								
	{1}	{2}	{3}	{4}	{5}	{6}	{7}	{8}
	M=11.680	M=4.5780	M=10.083	M=6.4033	M=5.7750	M=5.3475	M=6.2433	M=12.340
G_1:1 {1}		0.026108	0.626693	0.090632	0.078458	0.059997	0.092868	0.873579
G_2:2 {2	0.026108		0.003004	0.152256	0.491749	0.657842	0.275714	0.015736
G_3:3 {3	0.626693	0.003004		0.035553	0.043918	0.027693	0.048852	0.492628
G_4:4 {4	0.090632	0.152256	0.035553		0.710833	0.534059	0.913114	0.058311
G_5:5 {5	0.078458	0.491749	0.043918	0.710833		0.836714	0.804676	0.051633
G_6:6 {6	0.059997	0.657842	0.027693	0.534059	0.836714		0.636593	0.038885
G_7:7 {7	0.092868	0.275714	0.048852	0.913114	0.804676	0.636593		0.06084
G_8:8 {8	0.873579	0.015736	0.492628	0.058311	0.051633	0.038885	0.06084	

Supplementary Figure 4. Relative position and radii of the clusters in connection with Body Mass Index (BMI). Cluster 3 and singleton 8 demonstrate normal weight (BMI<25), other clusters contain mixed samples with cluster average over 25. The table below the plot area contains the results of MANOVA.

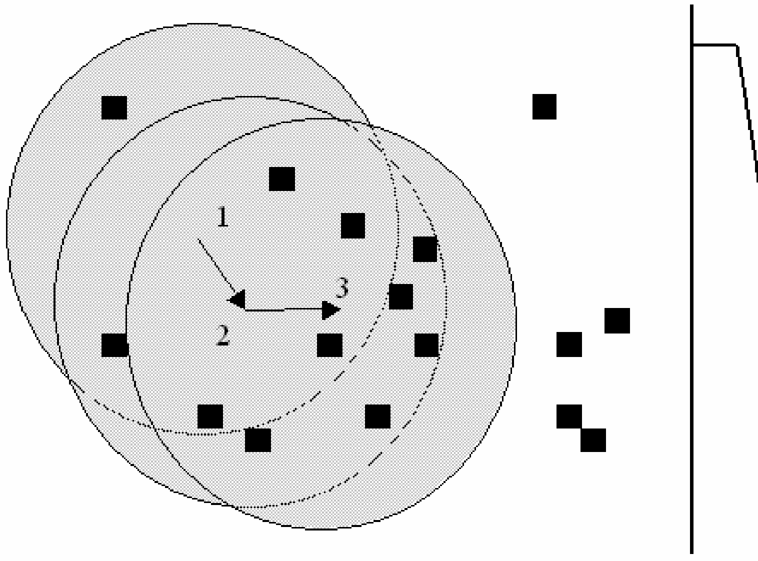


```
select starting points;
select radius range;
do{
  for each starting point Sj;
    for each radius within range with increment r;
      do{
        place a starting point Sj;
        mark each point within the Rjr distance;
        calculate the mass center of all marked;
        move the hypersphere center to the mass center;
      }until the hypersphere stops;
      collect all marked points in a provisional cluster;
      estimate the cluster quality;
    }
  }
  select the best-rated provisional cluster by quality;
  remove the best cluster from consideration;
}until no more objects left or the best cluster is a singleton;
```

Supplementary Figure 5. Pseudocode for the FOREL algorithm. The highlighted block is illustrated graphically above the code. Clusters are rounded up by a sphere, moving from arbitrary stating point to the mass center of all objects within its' radius. Out of all provisional clusters the best is selected according to the statistical quality metric and removed analysis. Extraction of clusters re-iterated on the remaining data until all objects are classified or the best possible cluster does not satisfy the minimal quality threshold.

Algorithm description: The algorithm starts with placement of the hypersphere in a certain coordinate, which can be one of the objects or centroid of a pre-defined cluster or any other point of interest in the feature space. The position of the "formal element" element is calculated as a center of mass of the provisional cluster, in which all elements have differential weights. Provisional clusters consist of the elements, captured by the hypersphere while it moves gradually from the starting to the resting point. Weights can be assigned to the objects based on the on the special interest (for example, genes,

involved in a certain metabolic pathway) or re-assigned during the clustering process (for example, re-scaled in respect with the distance from the hypersphere starting position). After the mass center of all captured objects is calculated, its center is moved to the new position. If new objects are found within the radius from the new position, they are added to the provisional cluster and the mass center is recalculated. This process is repeated until the no more objects can be added on the current step of the algorithm. The validity of the provisional cluster can be verified by some statistical utility measure like density, variance, sum of inter-cluster distances, etc. If the cluster meets the selection criteria, it is removed from the original data set and the process reiterated until no more statistically valid (according to the chosen metric) clusters are left.

The implementation developed at PBRC employs a complete test of every object as a possible cluster seed or hypersphere starting point. Computation efficiency can be significantly improved by limiting the number of objects serving as potential cluster seeds. This can be done by preliminary analysis of data and pre-selection of cluster seed candidates. It is also possible to introduce artificial or naturally derived expression profiles as starting points with overwhelmingly high preference. In this case a strictly supervised clustering can be performed around the limited number of "models" only. Otherwise, any degree of partial supervision can be introduced by allowing starting points at low, but non-zero priority objects. The default settings make the algorithm completely unsupervised with no pre-set number of clusters.

The radius of a hypersphere, delimiting the vicinity of the provisional cluster centroid is a parameter. In some cases the radius can be assigned a constant value, not changing during clustering process. In case of microarray data, variation of spot intensity on the duplicated samples can be estimated and used to determine the radius value. For example, it can be set so that objects (expression profiles) will only be found in one cluster if they are show the same expression pattern, with variation explained by stochastic reasons. By default the current version of the program implements an iterative solution: all possible radii are tested with a certain step within a limited range. The step (or precision) can be derived from the analysis of variation of distances within the whole data set. The range is defined by the minimal and maximal distance found within the whole dataset. These are extreme values, with a radius less then a minimal distance, the algorithms can produce only singletons, and on the other hand with radius equal to the maximal distance, all objects are guaranteed to fall into one big cluster. The best radius is one that produces a provisional cluster with the best quality. Cutting percentile margins from the possible radius range can reduce the computation demands of the program. By default 20% of the range are cut from both minimal and maximal radius values, but these parameters can be changed to suit particular data.

Introduction of different cluster fitness metrics modifies the algorithm and makes it very flexible to the demands of researcher. In the class discovery analysis we applied variance-based fitness metric.

$F_i = {}^{1}\!/_{\sigma_i^2}$ if $n_i \geq 2$ and $F_i=0$ otherwise.

Some variants of classification presented in the supplementary materials use a different fitness metric

$$F_i = \frac{n_i}{\sum_{j=0}^{n_i}\sum_{j=0}^{n_i} d\left(\vec{g}_j, \vec{g}_k\right)} \quad if \quad n_i \geq 2 \quad and \quad F_i = 0 \quad otherwise$$

Here $d\left(\vec{g}_j, \vec{g}_k\right)$ is a Euclidean distance between data vectors *g* representing specimens of the same *i*-th provisional cluster. In this project we have used only Euclidean distance, although the algorithm allows using almost any measure of similarity between data objects. This metric makes clustering algorithm similar to the complete linkage hierarchical tree, although it still produces finished clusters in the output file. Using this metric helps identifying small tight groups of specimens. This metric required significantly more computation compared to the metrics based on standard deviation or a distance from the cluster centroid. We have also implemented metrics that give preference to big sparse or long slender, or other shape of clusters. Some of our cluster fitness metrics are based on relative distances between objects within and outside the cluster. The metric is chosen before the clustering starts and remains constant. On each iteration all unclassified objects are tested as potential cluster seeds and all possible radius values are tested. At this point provisional clusters are fuzzy and may overlap completely or partially. The best cluster is chosen from the list of provisional clusters, drafted with different seeds and radius values. Each iteration isolates the best cluster according to the particular cluster quality metric. It is also possible to utilize a few concurrent metrics, isolating the clusters that score good by a few or exceptionally by one metric. In a sense, the algorithm works on the data like a little boy on a Christmas pudding: first it goes after raisins, then pick out nuts, then decides weather it worth to eat what has left on the plate. Gradual removal of the best clusters from iteration to iteration makes possible to separate mixtures of clusters completely or partially included in each other, like raisins in a doe, even in cases when the centroids of such clusters may be undistinguishable.

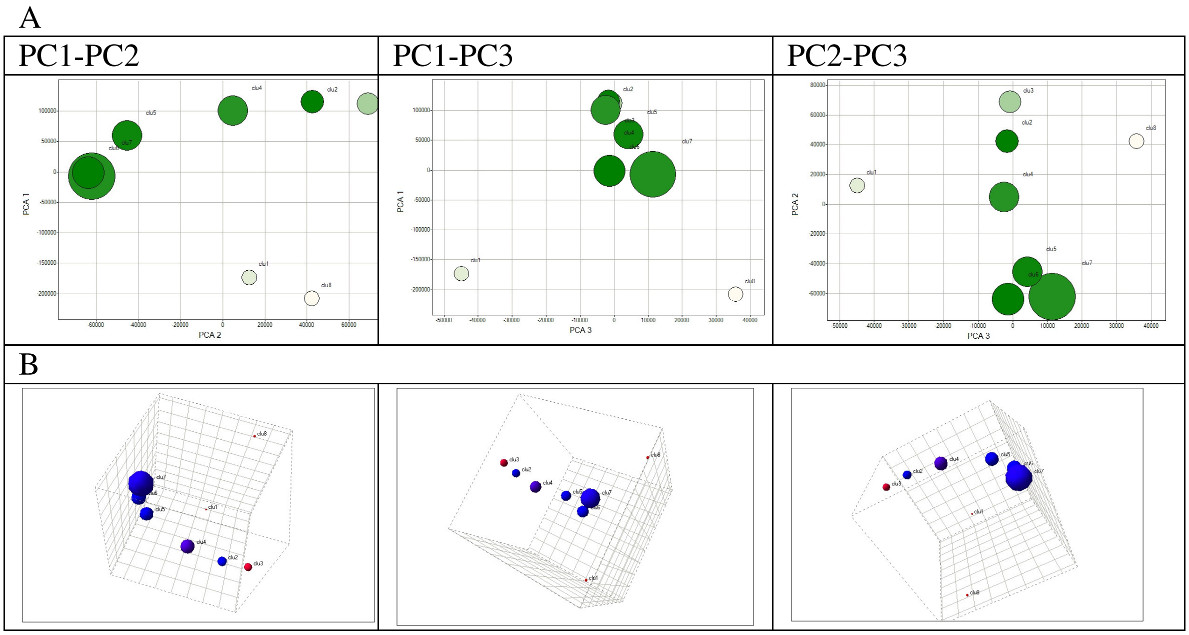

Supplementary Figure 6. **Different angles and projections of cluster juxtaposition in principal component space**. **A**: three projections into space of two principal components; **B**: position of clusters viewed from three different angles.

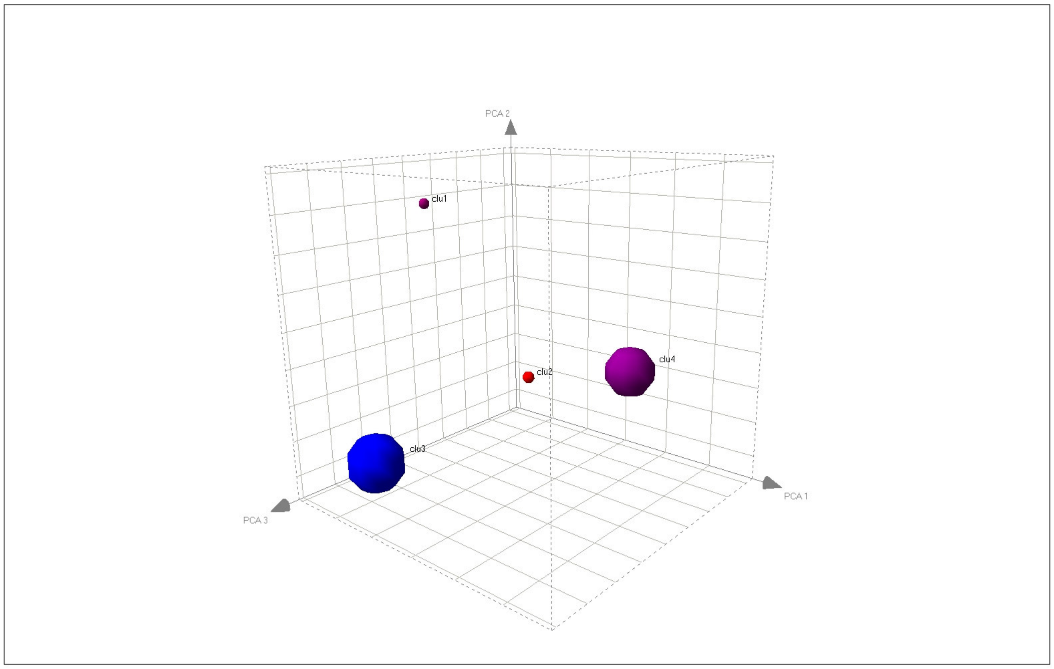

Supplementary figure 7. Relative position of FOREL clusters in the Patti et al. data set. Only 15 samples total are available, they fall into 4 clusters: 2 of 3 elements each, one of 4 and one of 5 elements. Clusters are colored by the prevalence of diabetic samples. Cluster 2 (the furthest) contains only non-diabetic samples, cluster 3 (the nearest) contains only diabetic samples, other two clusters are mixed. The low number of samples makes the interpretation unreliable, but the layout of clusters is at least consistent with the hypothesis of the “metabolically sound” core (cluster 2) and a “comet tail” type of variation with mixed clusters (1 and 4) connecting to the opposite least “metabolically sound” group (cluster 3).
