# Supplementary material for: Unsupervised clustering of gene expression data points at hypoxia as possible trigger for metabolic syndrome
Source: BMC Genomics. 2006 Dec 19;7:318. doi: 10.1186/1471-2164-7-318 (PMC1770922; doi:10.1186/1471-2164-7-318)
Supplement: Additional file 2 — Supplementary Table 1. This PDF file contains the table of genes, found differentially expressed between the most and the least metabolically sound clusters in our analysis of Mootha et al. data set. [file 1471-2164-7-318-S2.pdf]

Functional annotation for the Mootha dataset FOREL analysis

Probe	Gene Symbol	LocusLink	Name	GENEONTOLOGY	Annotation	Intensity on the fat end	Intensity on the lean end	Distance	Fold change (Log2)
217414_x_at	HBA2	3040	hemoglobin, alpha 2	hemoglobin complex	[SUMMARY:] The human alpha globin gene cluster located on chromosome 16 spans about 30 kb and includes the following five loci: 5'- zeta - pseudozeta - pseudoalpha-1 - alpha-2 - alpha-1 -3'. The alpha-2 (HBA2) and alpha-1 (HBA1) coding sequences are identical. These genes differ slightly over the 5' untranslated regions and the introns, but they differ significantly over the 3' untranslated regions. Two alpha chains plus two beta chains constitute HbA, which in normal adult life comprises about 97% of the total hemoglobin; alpha chains combine with delta chains to constitute HbA-2, which with HbF (fetal hemoglobin) makes up the remaining 3% of adult hemoglobin. Alpha thalassemias result from deletions of each of the alpha genes as well as deletions of both HBA2 and HBA1 respectively; some nondeletion alpha thalassemias have also been reported.	12458	900.3	12490.49	3.79
217232_x_at						21626.6	1732.6	21695.89	3.64
211074_at						3015.92	245.3	3025.88	3.62
209116_x_at	HBB	3043	hemoglobin, beta	hemoglobin complex; oxygen transport; oxygen transporter activity; transport	[SUMMARY:] The alpha (HBA) and beta (HBB) loci determine the structure of the 2 types of polypeptide chains in adult hemoglobin, Hb A. The normal adult hemoglobin tetramer consists of two alpha chains and two beta chains. Mutant beta globin causes sickle cell anemia. Absence of beta chain causes beta-zero-thalassemia. Reduced amounts of detectable beta globin causes beta-plus-thalassemia. The order of the genes in the beta-globin cluster is 5'-epsilon -- gamma-G -- gamma-A -- delta -- beta--3'.	22040.6	1816.22	22115.3	3.6
211699_x_at	HBA1	3039	hemoglobin, alpha 1	hemoglobin complex; oxygen transport; oxygen transporter activity; protein binding; transport	[SUMMARY:] The human alpha globin gene cluster located on chromosome 16 spans about 30 kb and includes the following five loci: 5'- zeta - pseudozeta - pseudoalpha-1 - alpha-2 - alpha-1 -3'. The alpha-2 (HBA2) and alpha-1 (HBA1) coding sequences are identical. These genes differ slightly over the 5' untranslated regions and the introns, but they differ significantly over the 3' untranslated regions. Two alpha chains plus two beta chains constitute HbA, which in normal adult life comprises about 97% of the total hemoglobin; alpha chains combine with delta chains to constitute HbA-2, which with HbF (fetal hemoglobin) makes up the remaining 3% of adult hemoglobin. Alpha thalassemias result from deletions of each of the alpha genes as well as deletions of both HBA2 and HBA1 respectively; some nondeletion alpha thalassemias have also been reported.	11978.3	1010.33	12020.83	3.57
211745_x_at	HBA1	3039	hemoglobin, alpha 1	hemoglobin complex; oxygen transport; oxygen transporter activity; protein binding; transport	[SUMMARY:] The human alpha globin gene cluster located on chromosome 16 spans about 30 kb and includes the following five loci: 5'- zeta - pseudozeta - pseudoalpha-1 - alpha-2 - alpha-1 -3'. The alpha-2 (HBA2) and alpha-1 (HBA1) coding sequences are identical. These genes differ slightly over the 5' untranslated regions and the introns, but they differ significantly over the 3' untranslated regions. Two alpha chains plus two beta chains constitute HbA, which in normal adult life comprises about 97% of the total hemoglobin; alpha chains combine with delta chains to constitute HbA-2, which with HbF (fetal hemoglobin) makes up the remaining 3% of adult hemoglobin. Alpha thalassemias result from deletions of each of the alpha genes as well as deletions of both HBA2 and HBA1 respectively; some nondeletion alpha thalassemias have also been reported.	18041.55	1532	18106.48	3.56
204018_x_at	HBA1	3039	hemoglobin, alpha 1	hemoglobin complex; oxygen transport; oxygen transporter activity; protein binding; transport	[SUMMARY:] The human alpha globin gene cluster located on chromosome 16 spans about 30 kb and includes the following five loci: 5'- zeta - pseudozeta - pseudoalpha-1 - alpha-2 - alpha-1 -3'. The alpha-2 (HBA2) and alpha-1 (HBA1) coding sequences are identical. These genes differ slightly over the 5' untranslated regions and the introns, but they differ significantly over the 3' untranslated regions. Two alpha chains plus two beta chains constitute HbA, which in normal adult life comprises about 97% of the total hemoglobin; alpha chains combine with delta chains to constitute HbA-2, which with HbF (fetal hemoglobin) makes up the remaining 3% of adult hemoglobin. Alpha thalassemias result from deletions of each of the alpha genes as well as deletions of both HBA2 and HBA1 respectively; some nondeletion alpha thalassemias have also been reported.	16755.8	1429.55	16816.67	3.55
209458_x_at	HBA1	3039	hemoglobin, alpha 1	hemoglobin complex; oxygen transport; oxygen transporter activity; protein binding; transport	[SUMMARY:] The human alpha globin gene cluster located on chromosome 16 spans about 30 kb and includes the following five loci: 5'- zeta - pseudozeta - pseudoalpha-1 - alpha-2 - alpha-1 -3'. The alpha-2 (HBA2) and alpha-1 (HBA1) coding sequences are identical. These genes differ slightly over the 5' untranslated regions and the introns, but they differ significantly over the 3' untranslated regions. Two alpha chains plus two beta chains constitute HbA, which in normal adult life comprises about 97% of the total hemoglobin; alpha chains combine with delta chains to constitute HbA-2, which with HbF (fetal hemoglobin) makes up the remaining 3% of adult hemoglobin. Alpha thalassemias result from deletions of each of the alpha genes as well as deletions of both HBA2 and HBA1 respectively; some nondeletion alpha thalassemias have also been reported.	14409.33	1327.47	14470.34	3.44
221419_s_at						3184.45	305.9	3199.11	3.38
211696_x_at	HBB	3043	hemoglobin, beta	hemoglobin complex; oxygen transport; oxygen transporter activity; transport	[SUMMARY:] The alpha (HBA) and beta (HBB) loci determine the structure of the 2 types of polypeptide chains in adult hemoglobin, Hb A. The normal adult hemoglobin tetramer consists of two alpha chains and two beta chains. Mutant beta globin causes sickle cell anemia. Absence of beta chain causes beta-zero-thalassemia. Reduced amounts of detectable beta globin causes beta-plus-thalassemia. The order of the genes in the beta-globin cluster is 5'-epsilon -- gamma-G -- gamma-A -- delta -- beta--3'.	23297.73	2309.32	23411.9	3.33
214414_x_at	HBA1	3039	hemoglobin, alpha 1	hemoglobin complex; oxygen transport; oxygen transporter activity; protein binding; transport	[SUMMARY:] The human alpha globin gene cluster located on chromosome 16 spans about 30 kb and includes the following five loci: 5'- zeta - pseudozeta - pseudoalpha-1 - alpha-2 - alpha-1 -3'. The alpha-2 (HBA2) and alpha-1 (HBA1) coding sequences are identical. These genes differ slightly over the 5' untranslated regions and the introns, but they differ significantly over the 3' untranslated regions. Two alpha chains plus two beta chains constitute HbA, which in normal adult life comprises about 97% of the total hemoglobin; alpha chains combine with delta chains to constitute HbA-2, which with HbF (fetal hemoglobin) makes up the remaining 3% of adult hemoglobin. Alpha thalassemias result from deletions of each of the alpha genes as well as deletions of both HBA2 and HBA1 respectively; some nondeletion alpha thalassemias have also been reported.	14654.9	1765.92	14760.91	3.05

AFFX-HUMGAPDH/M33197_5_at	GAPD	2597	glyceraldehyde-3-phosphate dehydrogenase	cytoplasm; glucose metabolism; glyceraldehyde-3-phosphate dehydrogenase (phosphorylating) activity; glycolysis; oxidoreductase activity	[SUMMARY:] Glyceraldehyde-3-phosphate dehydrogenase catalyzes an important energy-yielding step in carbohydrate metabolism, the reversible oxidative phosphorylation of glyceraldehyde-3-phosphate in the presence of inorganic phosphate and nicotinamide adenine dinucleotide (NAD). The enzyme exists as a tetramer of identical chains. A GAPD pseudogene has been mapped to Xp21-p11 and 15 GAPD-like loci have been identified.	21964.3	5356.38	22607.99	2.04
217398_x_at	GAPD	2597	glyceraldehyde-3-phosphate dehydrogenase	cytoplasm; glucose metabolism; glyceraldehyde-3-phosphate dehydrogenase (phosphorylating) activity; glycolysis; oxidoreductase activity	[SUMMARY:] Glyceraldehyde-3-phosphate dehydrogenase catalyzes an important energy-yielding step in carbohydrate metabolism, the reversible oxidative phosphorylation of glyceraldehyde-3-phosphate in the presence of inorganic phosphate and nicotinamide adenine dinucleotide (NAD). The enzyme exists as a tetramer of identical chains. A GAPD pseudogene has been mapped to Xp21-p11 and 15 GAPD-like loci have been identified.	22813.43	5741.8	23524.89	1.99
209742_s_at	MYL2	4633	myosin, light polypeptide 2, regulatory, cardiac, slow		[SUMMARY:] MYL2 encodes the regulatory light chain associated with cardiac myosin beta (or slow) heavy chain. Ca+ triggers the phosphorylation of regulatory light chain that in turn triggers contraction. Mutations in MYL2 are associated with mid-left ventricular chamber type hypertrophic cardiomyopathy.	23448.95	6005.65	24205.81	1.97
212581_x_at	GAPD	2597	glyceraldehyde-3-phosphate dehydrogenase	cytoplasm; glucose metabolism; glyceraldehyde-3-phosphate dehydrogenase (phosphorylating) activity; glycolysis; oxidoreductase activity	[SUMMARY:] Glyceraldehyde-3-phosphate dehydrogenase catalyzes an important energy-yielding step in carbohydrate metabolism, the reversible oxidative phosphorylation of glyceraldehyde-3-phosphate in the presence of inorganic phosphate and nicotinamide adenine dinucleotide (NAD). The enzyme exists as a tetramer of identical chains. A GAPD pseudogene has been mapped to Xp21-p11 and 15 GAPD-like loci have been identified.	22474.95	6071.9	23280.71	1.89
211943_x_at	TPT1	7178	tumor protein, translationally-	cytoplasm; extracellular space; molecular_function		17170.12	4739.5	17812.24	1.86
204810_s_at	CKM	1158	creatine kinase, muscle	creatine kinase activity; transferase activity, transferring phosphorus-containing groups	[SUMMARY:] The protein encoded by this gene is a cytoplasmic enzyme involved in energy homeostasis and is an important serum marker for myocardial infarction. The encoded protein reversibly catalyzes the transfer of phosphate between ATP and various phosphogens such as creatine phosphate. It acts as a homodimer in striated muscle as well as in other tissues, and as a heterodimer with a similar brain isozyme in heart. The encoded protein is a member of the ATP:guanido phosphotransferase protein family.	21913.28	6066.75	22737.57	1.85
201540_at	FHL1	2273	four and a half LIM domains 1	cell differentiation; cell growth; cellular_component unknown; molecular_function unknown; muscle		17534.7	4945.75	18218.84	1.83
213453_x_at	GAPD	2597	glyceraldehyde-3-phosphate dehydrogenase	cytoplasm; glucose metabolism; glyceraldehyde-3-phosphate dehydrogenase (phosphorylating) activity; glycolysis; oxidoreductase activity	[SUMMARY:] Glyceraldehyde-3-phosphate dehydrogenase catalyzes an important energy-yielding step in carbohydrate metabolism, the reversible oxidative phosphorylation of glyceraldehyde-3-phosphate in the presence of inorganic phosphate and nicotinamide adenine dinucleotide (NAD). The enzyme exists as a tetramer of identical chains. A GAPD pseudogene has been mapped to Xp21-p11 and 15 GAPD-like loci have been identified.	18788.35	5315.08	19525.68	1.82
AFFX-HUMGAPDH/M33197_3_at	GAPD	2597	glyceraldehyde-3-phosphate dehydrogenase	cytoplasm; glucose metabolism; glyceraldehyde-3-phosphate dehydrogenase (phosphorylating) activity; glycolysis; oxidoreductase activity	[SUMMARY:] Glyceraldehyde-3-phosphate dehydrogenase catalyzes an important energy-yielding step in carbohydrate metabolism, the reversible oxidative phosphorylation of glyceraldehyde-3-phosphate in the presence of inorganic phosphate and nicotinamide adenine dinucleotide (NAD). The enzyme exists as a tetramer of identical chains. A GAPD pseudogene has been mapped to Xp21-p11 and 15 GAPD-like loci have been identified.	18175.8	5191.67	18902.73	1.81
204326_x_at	MT1X	4501	metallothionein 1X	metal ion binding; response to metal ion		3139.3	914.67	3269.84	1.78
209888_s_at	MYL1	4632	myosin, light polypeptide 1, alkali; skeletal, fast	calcium ion binding; muscle development; muscle myosin; myosin; structural constituent of muscle	[SUMMARY:] Myosin is a hexameric ATPase cellular motor protein. It is composed of two heavy chains, two nonphosphorylatable alkali light chains, and two phosphorylatable regulatory light chains. This gene encodes a myosin alkali light chain expressed in fast skeletal muscle. Two transcript variants have been identified for this gene.	21170.85	6243	22072.15	1.76
214687_x_at	ALDOA	226	aldolase A, fructose-bisphosphate	fructose metabolism; fructose-bisphosphate aldolase activity; glycolysis; lyase activity; striated muscle contraction	[SUMMARY:] This gene product, Aldolase A (fructose-bisphosphate aldolase) is a glycolytic enzyme that catalyzes the reversible conversion of fructose-1,6-bisphosphate to glyceraldehyde 3-phosphate and dihydroxyacetone phosphate. Three aldolase isozymes (A, B, and C), encoded by three different genes, are differentially expressed during development. Aldolase A is found in the developing embryo and is produced in even greater amounts in adult muscle. Aldolase A expression is repressed in adult liver, kidney and intestine and similar to aldolase C levels in brain and other nervous tissue. Aldolase A deficiency has been associated with myopathy and hemolytic anemia. Alternative splicing of this gene results in multiple transcript variants which encode the same protein.	17523.45	5197.9	18278.11	1.75
212284_x_at	TPT1	7178	tumor protein, translationally-	cytoplasm; extracellular space; molecular_function		20994.73	6267.15	21910.17	1.74
200966_x_at	ALDOA	226	aldolase A, fructose-bisphosphate	fructose metabolism; fructose-bisphosphate aldolase activity; glycolysis; lyase activity; striated muscle contraction	[SUMMARY:] This gene product, Aldolase A (fructose-bisphosphate aldolase) is a glycolytic enzyme that catalyzes the reversible conversion of fructose-1,6-bisphosphate to glyceraldehyde 3-phosphate and dihydroxyacetone phosphate. Three aldolase isozymes (A, B, and C), encoded by three different genes, are differentially expressed during development. Aldolase A is found in the developing embryo and is produced in even greater amounts in adult muscle. Aldolase A expression is repressed in adult liver, kidney and intestine and similar to aldolase C levels in brain and other nervous tissue. Aldolase A deficiency has been associated with myopathy and hemolytic anemia. Alternative splicing of this gene results in multiple transcript variants which encode the same protein.	16771.3	5164.25	17548.39	1.7
AFFX-HUMGAPDH/M33197_M_at	GAPD	2597	glyceraldehyde-3-phosphate dehydrogenase	cytoplasm; glucose metabolism; glyceraldehyde-3-phosphate dehydrogenase (phosphorylating) activity; glycolysis; oxidoreductase activity	[SUMMARY:] Glyceraldehyde-3-phosphate dehydrogenase catalyzes an important energy-yielding step in carbohydrate metabolism, the reversible oxidative phosphorylation of glyceraldehyde-3-phosphate in the presence of inorganic phosphate and nicotinamide adenine dinucleotide (NAD). The enzyme exists as a tetramer of identical chains. A GAPD pseudogene has been mapped to Xp21-p11 and 15 GAPD-like loci have been identified.	17897.57	5527.12	18731.58	1.7
208581_x_at	MT1X	4501	metallothionein 1X	metal ion binding; response to metal ion		2776.8	872.42	2910.63	1.67
AFFX-HSAC07/X00351_5_at	ACTB	60	actin, beta	actin filament; cell motility; motor activity; structural constituent of cytoskeleton	[SUMMARY:] Beta actin is one of six different actin isoforms which have been identified. ACTB is one of the two nonmuscle cytoskeletal actins. Actins are highly conserved proteins that are involved in cell motility, structure and integrity. Alpha actins are a major constituent of the contractile apparatus.	3928.27	1263.3	4126.41	1.64
207783_x_at	TPT1	7178	tumor protein, translationally-	cytoplasm; extracellular space; molecular_function		21551.73	6966.62	22649.74	1.63

212869_x_at	TPT1	7178	tumor protein, translationally-	cytoplasm; extracellular space; molecular_function		22499.38	7417.92	23690.66	1.6
214327_x_at	TPT1	7178	tumor protein, translationally-	cytoplasm; extracellular space; molecular_function		13548.27	4457.83	14262.82	1.6
206323_x_at	OPHN1	4983	oligophrenin 1	Rho GTPase activator activity; axon guidance; neurogenesis; signal transduction; substrate-bound cell migration, cell	[SUMMARY:] Oligophrenin 1 has 25 exons and encodes a Rho-GTPase-activating protein. The Rho proteins are important mediators of intracellular signal transduction, which affects cell migration and cell morphogenesis. Mutations in this gene are responsible for non-specific X-linked mental retardation.	2692.52	907.5	2841.35	1.57
206117_at	TPM1	7168	tropomyosin 1 (alpha)	actin binding; biological_process unknown; cellular_component unknown; cytoskeleton; molecular_function unknown; muscle development; muscle thin filament tropomyosin; regulation of heart rate; regulation of muscle contraction; structural	[SUMMARY:] Tropomyosins are ubiquitous proteins of 35 to 45 kD associated with the actin filaments of myofibrils and stress fibers. In vertebrates, 4 known tropomyosin genes code for diverse isoforms that are expressed in a tissue-specific manner and regulated by an alternative splicing mechanism (Lees-Miller and Helfman, 1991 [PubMed 1796905]). The vertebrate alpha-tropomyosin gene consists of 15 exons; 5 exons are found in all transcripts, while 10 exons are alternatively used in different alpha-tropomyosin RNAs (Lees-Miller and Helfman, 1991 [PubMed 1796905]). The striated muscle isoform is expressed in both cardiac and skeletal muscle tissues.[supplied by OMIM]	8922.65	3036.68	9425.24	1.55
216520_s_at	TPT1	7178	tumor protein, translationally-	cytoplasm; extracellular space; molecular_function		15072.12	5310.52	15980.32	1.5
211296_x_at	UBC	7316	ubiquitin C	polyubiquitin; ubiquitin	[Proteome FUNCTION:] Ubiquitin C; polyubiquitin protein precursor that marks cellular proteins for degradation	11571.12	4177.75	12302.22	1.47
205163_at	HUMMLC2B	29895	myosin light chain 2	calcium ion binding; muscle myosin; myosin; structural constituent of muscle		16502.43	6058.5	17579.4	1.45
208695_s_at	RPL39	6170	ribosomal protein L39	RNA binding; cytosolic large ribosomal subunit (sensu Eukarya); protein biosynthesis; structural protein of ribosome	[SUMMARY:] Ribosomes, the organelles that catalyze protein synthesis, consist of a small 40S subunit and a large 60S subunit. Together these subunits are composed of 4 RNA species and approximately 80 structurally distinct proteins. This gene encodes a ribosomal protein that is a component of the 60S subunit. The protein belongs to the S39E family of ribosomal proteins. It is located in the cytoplasm. In rat, the protein is the smallest, and one of the most basic, proteins of the ribosome. This gene is co-transcribed with the U69 small nucleolar RNA gene, which is located in its second intron. As is typical for genes encoding ribosomal proteins, there are multiple processed pseudogenes of this gene dispersed through the genome.	10704.88	3967	11416.28	1.43
AFFX-HSAC07/X00351_M_at	ACTB	60	actin, beta	actin filament; cell motility; motor activity; structural constituent of cytoskeleton	[SUMMARY:] Beta actin is one of six different actin isoforms which have been identified. ACTB is one of the two nonmuscle cytoskeletal actins. Actins are highly conserved proteins that are involved in cell motility, structure and integrity. Alpha actins are a major constituent of the contractile apparatus.	2712.43	1029.18	2901.11	1.4
204179_at	MB	4151	myoglobin		[SUMMARY:] The human myoglobin gene is 10.4 kb long and has a three exon/two intron structure with long non-coding regions. It encodes the protein myoglobin, which is a haemoprotein contributing to intracellular oxygen storage and transcellular facilitated diffusion of oxygen. Myoglobin is a member of the globin superfamily and present in skeletal and cardiac muscle. At least three alternatively spliced transcript variants encoding the same protein have been reported.	13721.6	5231.62	14685.1	1.39
205388_at	TNNC2	7125	troponin C2, fast		[SUMMARY:] Troponin (Tn), a key protein complex in the regulation of striated muscle contraction, is composed of 3 subunits. The Tn-I subunit inhibits actomyosin ATPase, the Tn-T subunit binds tropomyosin and Tn-C, while the Tn-C subunit binds calcium and overcomes the inhibitory action of the troponin complex on actin filaments. The protein encoded by this gene is the Tn-C subunit.	15295.55	5818.08	16364.71	1.39
204865_at	CA3	761	carbonic anhydrase III, muscle specific		[SUMMARY:] Carbonic anhydrase III (CAIII) is a member of a multigene family (at least six separate genes are known) that encode carbonic anhydrase isozymes. These carbonic anhydrases are a class of metalloenzymes that catalyze the reversible hydration of carbon dioxide and are differentially expressed in a number of cell types. The expression of the CA3 gene is strictly tissue specific and present at high levels in skeletal muscle and much lower levels in cardiac and smooth muscle. A proportion of carriers of Duchenne muscle dystrophy have a higher CA3 level than normal. The gene spans 10.3 kb and contains seven exons and six introns.	3140.83	1244.05	3378.23	1.34
205589_at	MYL3	4634	myosin, light polypeptide 3, alkali; ventricular, skeletal, slow	calcium ion binding; muscle contraction; muscle development; muscle myosin; myosin; structural	[SUMMARY:] MYL3 encodes myosin light chain 3, an alkali light chain also referred to in the literature as both the ventricular isoform and the slow skeletal muscle isoform. Mutations in MYL3 have been identified as a cause of mid-left ventricular chamber type hypertrophic cardiomyopathy.	5557.25	2205.23	5978.8	1.33
201841_s_at	HSPB1	3315	heat shock 27kDa protein 1	cytoplasm; heat shock protein activity; regulation of translational initiation		3327.92	1342.73	3588.59	1.31
214505_s_at	FHL1	2273	four and a half LIM domains 1	cell differentiation; cell growth; cellular_component unknown; molecular_function unknown; muscle		8599.43	3467.45	9272.18	1.31
219509_at	MYOZ1	58529	myozenin 1			8572.03	3495.65	9257.39	1.29
200869_at	RPL18A	6142	ribosomal protein L18a	RNA binding; cytosolic large ribosomal subunit (sensu Eukarya); intracellular; protein biosynthesis; ribosome; structural constituent of ribosome	[SUMMARY:] Ribosomes, the organelles that catalyze protein synthesis, consist of a small 40S subunit and a large 60S subunit. Together these subunits are composed of 4 RNA species and approximately 80 structurally distinct proteins. This gene encodes a ribosomal protein that is a component of the 60S subunit. The protein belongs to the L18AE family of ribosomal proteins. It is located in the cytoplasm. This gene is co-transcribed with the U68 snoRNA, which is located in its third intron. As is typical for genes encoding ribosomal proteins, there are multiple processed pseudogenes of this gene dispersed through the genome.	5724.07	2386.75	6201.74	1.26
205177_at	TNNI1	7135	troponin I, skeletal, slow	actin binding; muscle development; regulation of striated muscle contraction; tropomyosin binding;		7044.95	2940.73	7634.08	1.26

201094_at	RPS29	6235	ribosomal protein S29		[SUMMARY:] Ribosomes, the organelles that catalyze protein synthesis, consist of a small 40S subunit and a large 60S subunit. Together these subunits are composed of 4 RNA species and approximately 80 structurally distinct proteins. This gene encodes a ribosomal protein that is a component of the 40S subunit and a member of the S14P family of ribosomal proteins. The protein, which contains a C2-C2 zinc finger-like domain that can bind to zinc, can enhance the tumor suppressor activity of Ras-related protein 1A (KREV1). It is located in the cytoplasm. Variable expression of this gene in colorectal cancers compared to adjacent normal tissues has been observed, although no correlation between the level of expression and the severity of the disease has been found. As is typical for genes encoding ribosomal proteins, there are multiple processed pseudogenes of this gene dispersed through the genome.	9469.47	4031.25	10291.84	1.23
208980_s_at	UBC	7316	ubiquitin C	polyubiquitin; ubiquitin	[Proteome FUNCTION:] Ubiquitin C; polyubiquitin protein precursor that marks cellular proteins for degradation	4307.23	1883.57	4701.07	1.19
AFFX-hum_alu_at						11131.1	4864.7	12147.7	1.19
201160_s_at	CSDA	8531	cold shock domain protein A	DNA binding; RNA polymerase II transcription factor activity; cytoplasm; double-stranded DNA binding; negative regulation of transcription from Pol II promoter; perinuclear space; regulation of transcription, DNA-dependent; response to cold; transcription		6892.08	3051.55	7537.42	1.18
208645_s_at	RPS14	6208	ribosomal protein S14	RNA binding; cytosolic small ribosomal subunit (sensu Eukarya); intracellular; protein biosynthesis; ribosome; structural constituent of ribosome	[SUMMARY:] Ribosomes, the organelles that catalyze protein synthesis, consist of a small 40S subunit and a large 60S subunit. Together these subunits are composed of 4 RNA species and approximately 80 structurally distinct proteins. This gene encodes a ribosomal protein that is a component of the 40S subunit. The protein belongs to the S11P family of ribosomal proteins. It is located in the cytoplasm. Transcript variants utilizing alternative transcription initiation sites have been described in the literature. As is typical for genes encoding ribosomal proteins, there are multiple processed pseudogenes of this gene dispersed through the genome. In Chinese hamster ovary cells, mutations in this gene can lead to resistance to emetine, a protein synthesis inhibitor.	8605	3835.17	9420.97	1.17
209904_at	TNNC1	7134	troponin C, slow	calcium ion binding; muscle development; regulation of muscle contraction		12998.72	5806.8	14236.78	1.16
201429_s_at	RPL37A	6168	ribosomal protein L37a		[SUMMARY:] Ribosomes, the organelles that catalyze protein synthesis, consist of a small 40S subunit and a large 60S subunit. Together these subunits are composed of 4 RNA species and approximately 80 structurally distinct proteins. This gene encodes a ribosomal protein that is a component of the 60S subunit. The protein belongs to the L37AE family of ribosomal proteins. It is located in the cytoplasm. The protein contains a C4-type zinc finger-like domain. As is typical for genes encoding ribosomal proteins, there are multiple processed pseudogenes of this gene dispersed through the genome.	13384.2	6015.85	14674.03	1.15
200763_s_at	RPLP1	6176	ribosomal protein, large, P1	RNA binding; cytosolic large ribosomal subunit (sensu Eukarya); intracellular; protein biosynthesis; ribosome; structural constituent of ribosome; translational elongation	[SUMMARY:] Ribosomes, the organelles that catalyze protein synthesis, consist of a small 40S subunit and a large 60S subunit. Together these subunits are composed of 4 RNA species and approximately 80 structurally distinct proteins. This gene encodes a ribosomal phosphoprotein that is a component of the 60S subunit. The protein, which is a functional equivalent of the E. coli L7/L12 ribosomal protein, belongs to the L12P family of ribosomal proteins. It plays an important role in the elongation step of protein synthesis. Unlike most ribosomal proteins, which are basic, the encoded protein is acidic. Its C-terminal end is nearly identical to the C-terminal ends of the ribosomal phosphoproteins P0 and P2. The P1 protein can interact with P0 and P2 to form a pentameric complex consisting of P1 and P2 dimers, and a P0 monomer. The protein is located in the cytoplasm. As is typical for genes encoding ribosomal proteins, there are multiple processed pseudogenes of this gene dispersed through the genome.	9555.53	4428.02	10531.64	1.11
210298_x_at	FHL1	2273	four and a half LIM domains 1	cell differentiation; cell growth; cellular_component unknown; molecular_function unknown; muscle		7326.55	3398.22	8076.28	1.11
213890_x_at	RPS16	6217	ribosomal protein S16		[SUMMARY:] Ribosomes, the organelles that catalyze protein synthesis, consist of a small 40S subunit and a large 60S subunit. Together these subunits are composed of 4 RNA species and approximately 80 structurally distinct proteins. This gene encodes a ribosomal protein that is a component of the 40S subunit. The protein belongs to the S9P family of ribosomal proteins. It is located in the cytoplasm. As is typical for genes encoding ribosomal proteins, there are multiple processed pseudogenes of this gene dispersed through the genome.	6550.6	3027.97	7216.58	1.11
200024_at	RPS5	6193	ribosomal protein S5	RNA binding; cytosolic small ribosomal subunit (sensu Eukarya); intracellular; protein biosynthesis; structural constituent of ribosome	[SUMMARY:] Ribosomes, the organelles that catalyze protein synthesis, consist of a small 40S subunit and a large 60S subunit. Together these subunits are composed of 4 RNA species and approximately 80 structurally distinct proteins. This gene encodes a ribosomal protein that is a component of the 40S subunit. The protein belongs to the S7P family of ribosomal proteins. It is located in the cytoplasm. Variable expression of this gene in colorectal cancers compared to adjacent normal tissues has been observed, although no correlation between the level of expression and the severity of the disease has been found. As is typical for genes encoding ribosomal proteins, there are multiple processed pseudogenes of this gene dispersed through the genome.	4375.75	2036.93	4826.62	1.1

201257_x_at	RPS3A	6189	ribosomal protein S3A		[SUMMARY:] Ribosomes, the organelles that catalyze protein synthesis, consist of a small 40S subunit and a large 60S subunit. Together these subunits are composed of 4 RNA species and approximately 80 structurally distinct proteins. This gene encodes a ribosomal protein that is a component of the 40S subunit. The protein belongs to the S3AE family of ribosomal proteins. It is located in the cytoplasm. Disruption of the gene encoding rat ribosomal protein S3a, also named v-fos transformation effector protein, in v-fos-transformed rat cells results in reversion of the transformed phenotype. Transcript variants utilizing alternative transcription start sites have been described. This gene is co-transcribed with the U73A and U73B small nucleolar RNA genes, which are located in its fourth and third introns, respectively. As is typical for genes encoding ribosomal proteins, there are multiple processed pseudogenes of this gene dispersed through the genome.	12017.4	5607.55	13261.32	1.1
205374_at	SLN	6588	sarcolipin	enzyme regulator activity; integral to membrane; sarcoplasmic reticulum; smooth endoplasmic		11339.7	5277.77	12507.75	1.1
200633_at	UBB	7314	ubiquitin B		[SUMMARY:] This gene encodes ubiquitin, one of the most conserved proteins known. Ubiquitin is required for ATP-dependent, nonlysosomal intracellular protein degradation of abnormal proteins and normal proteins with a rapid turnover. Ubiquitin is covalently bound to proteins to be degraded, and presumably labels these proteins for degradation. Ubiquitin also binds to histone H2A in actively transcribed regions but does not cause histone H2A degradation, suggesting that ubiquitin is also involved in regulation of gene expression. This gene consists of three direct repeats of the ubiquitin coding sequence with no spacer sequence. Consequently, the protein is expressed as a polyubiquitin precursor with a final amino acid after the last repeat. Aberrant form of this protein has been noticed in patients with Alzheimer's and Down syndrome.	7593.15	3588.85	8398.56	1.08
212391_x_at	RPS3A	6189	ribosomal protein S3A		[SUMMARY:] Ribosomes, the organelles that catalyze protein synthesis, consist of a small 40S subunit and a large 60S subunit. Together these subunits are composed of 4 RNA species and approximately 80 structurally distinct proteins. This gene encodes a ribosomal protein that is a component of the 40S subunit. The protein belongs to the S3AE family of ribosomal proteins. It is located in the cytoplasm. Disruption of the gene encoding rat ribosomal protein S3a, also named v-fos transformation effector protein, in v-fos-transformed rat cells results in reversion of the transformed phenotype. Transcript variants utilizing alternative transcription start sites have been described. This gene is co-transcribed with the U73A and U73B small nucleolar RNA genes, which are located in its fourth and third introns, respectively. As is typical for genes encoding ribosomal proteins, there are multiple processed pseudogenes of this gene dispersed through the genome.	11597.5	5472.77	12823.93	1.08
205577_at	PYGM	5837	phosphorylase, glycogen; muscle (McArdle syndrome, glycogen storage			8303.58	4009.73	9221.02	1.05
201492_s_at	RPL41	6171	ribosomal protein L41	RNA binding; cytosolic large ribosomal subunit (sensu Eukarya); protein biosynthesis; structural constituent of ribosome	[SUMMARY:] Ribosomes, the organelles that catalyze protein synthesis, consist of a small 40S subunit and a large 60S subunit. Together these subunits are composed of 4 RNA species and approximately 80 structurally distinct proteins. This gene encodes a ribosomal protein that is a component of the 60S subunit. The protein, which shares sequence similarity with the yeast ribosomal protein YL41, belongs to the L41E family of ribosomal proteins. It is located in the cytoplasm. The protein can interact with the beta subunit of protein kinase CKII and can stimulate the phosphorylation of DNA topoisomerase II-alpha by CKII. As is typical for genes encoding ribosomal proteins, there are multiple processed pseudogenes of this gene dispersed through the genome.	9730.4	4745.92	10826.1	1.04
209283_at	CRYAB	1410	crystallin, alpha B	chaperone activity; cytoplasm; muscle contraction; nucleus; protein folding; structural constituent of eye lens; visual perception	[SUMMARY:] Crystallins are separated into two classes: taxon-specific, or enzyme, and ubiquitous. The latter class constitutes the major proteins of vertebrate eye lens and maintains the transparency and refractive index of the lens. Since lens central fiber cells lose their nuclei during development, these crystallins are made and then retained throughout life, making them extremely stable proteins. Mammalian lens crystallins are divided into alpha, beta, and gamma families; beta and gamma crystallins are also considered as a superfamily. Alpha and beta families are further divided into acidic and basic groups. Seven protein regions exist in crystallins: four homologous motifs, a connecting peptide, and N- and C-terminal extensions. Alpha crystallins are composed of two gene products: alpha-A and alpha-B, for acidic and basic, respectively. Alpha crystallins can be induced by heat shock and are members of the small heat shock protein (sHSP also known as the HSP20) family. They act as molecular chaperones although they do not renature proteins and release them in the fashion of a true chaperone; instead they hold them in larg	5016.77	2507.6	5608.57	1
204631_at	MYH2	4620	myosin, heavy polypeptide 2, skeletal muscle, adult	ATP binding; actin binding; calmodulin binding; microfilament motor activity; muscle development; muscle myosin; myosin; striated muscle contraction; striated muscle thick filament		9308.03	4701.12	10427.84	0.99
211956_s_at	SUI1	10209	putative translation initiation factor	cell growth and/or maintenance; cellular_component unknown; cytoplasm; regulation of protein biosynthesis; regulation of translation; regulation of translational initiation;		6438.73	3271.07	7221.99	0.98
201105_at	LGALS1	3956	lectin, galactoside-binding, soluble, 1 (galectin 1)		[SUMMARY:] The galectins are a family of beta-galactoside-binding proteins implicated in modulating cell-cell and cell-matrix interactions. LGALS1 may act as an autocrine negative growth factor that regulates cell proliferation.	2878.7	1483.77	3238.6	0.96
200674_s_at	RPL32	6161	ribosomal protein L32		[SUMMARY:] Ribosomes, the organelles that catalyze protein synthesis, consist of a small 40S subunit and a large 60S subunit. Together these subunits are composed of 4 RNA species and approximately 80 structurally distinct proteins. This gene encodes a ribosomal protein that is a component of the 60S subunit. The protein belongs to the L32E family of ribosomal proteins. It is located in the cytoplasm. Although some studies have mapped this gene to 3q13.3-q21, it is believed to map to 3p25-p24. As is typical for genes encoding ribosomal proteins, there are multiple processed pseudogenes of this gene dispersed through the genome.	7829.1	4091.08	8833.56	0.94

214087_s_at	MYBPC1	4604	myosin binding protein C, slow type	actin binding; cell adhesion; muscle development; protein binding; striated muscle contraction; striated muscle thick filament; structural constituent of muscle		7365.9	3848.6	8310.73	0.94
211600_at	PTPRO	5800	protein tyrosine phosphatase, receptor type, O	hydrolase activity; integral to membrane; integral to plasma membrane; protein amino acid dephosphorylation; protein-tyrosine-phosphatase activity; receptor activity; transmembrane receptor	[SUMMARY:] This gene encodes a receptor-type protein tyrosine phosphatase containing a single intracellular catalytic domain with a characteristic signature motif. The gene product, which has a transmembrane domain, is an integral membrane protein. Several alternatively spliced transcript variants, some of which encode different isoforms of the protein, have been described. These variants exhibit tissue-specific expression.	3961.13	2083.07	4475.46	0.93
200025_s_at	RPL27	6155	ribosomal protein L27	intracellular; protein biosynthesis; ribosome; structural constituent of ribosome	[SUMMARY:] Ribosomes, the organelles that catalyze protein synthesis, consist of a small 40S subunit and a large 60S subunit. Together these subunits are composed of 4 RNA species and approximately 80 structurally distinct proteins. This gene encodes a ribosomal protein that is a component of the 60S subunit. The protein belongs to the L27E family of ribosomal proteins. It is located in the cytoplasm. As is typical for genes encoding ribosomal proteins, there are multiple processed pseudogenes of this gene dispersed through the genome.	6842.7	3624.1	7743.17	0.92
201049_s_at						7989.17	4378.15	9110.17	0.87
212788_x_at	FTL	2512	ferritin, light polypeptide	binding; ferric iron binding; iron ion homeostasis; iron ion		2639.73	1446.75	3010.19	0.87
213201_s_at	TNNT1	7138	troponin T1, skeletal,			6633.92	3646.55	7570.09	0.86
201539_s_at	FHL1	2273	four and a half LIM domains 1	cell differentiation; cell growth; cellular_component unknown; molecular_function unknown; muscle		4949.2	2737.63	5655.9	0.85
205444_at	ATP2A1	487	ATPase, Ca++ transporting, cardiac muscle, fast twitch 1	ATP binding; calcium ion binding; calcium ion transport; calcium-transporting ATPase activity; cation transport; hydrolase activity; hydrolase activity, acting on acid anhydrides, catalyzing transmembrane movement of substances; integral to membrane; magnesium ion binding; metabolism; proton transport; regulation of striated muscle	[SUMMARY:] This gene encodes one of the SERCA Ca(2+)-ATPases, which are intracellular pumps located in the sarcoplasmic or endoplasmic reticula of muscle cells. This enzyme catalyzes the hydrolysis of ATP coupled with the translocation of calcium from the cytosol to the sarcoplasmic reticulum lumen, and is involved in muscular excitation and contraction. Mutations in this gene cause some autosomal recessive forms of Brody disease, characterized by increasing impairment of muscular relaxation during exercise. Alternative splicing results in two transcript variants encoding different isoforms.	7106.32	4036.63	8172.77	0.82
219106_s_at	KBTBD10	10324	kelch repeat and BTB (POZ) domain	cytoskeleton; protein binding; striated muscle contraction		4302.1	2482.5	4966.98	0.79
202029_x_at	RPL38	6169	ribosomal protein L38		[SUMMARY:] Ribosomes, the organelles that catalyze protein synthesis, consist of a small 40S subunit and a large 60S subunit. Together these subunits are composed of 4 RNA species and approximately 80 structurally distinct proteins. This gene encodes a ribosomal protein that is a component of the 60S subunit. The protein belongs to the L38E family of ribosomal proteins. It is located in the cytoplasm. As is typical for genes encoding ribosomal proteins, there are multiple processed pseudogenes of this gene dispersed through the genome, including one located in the promoter region of the type 1 angiotensin II receptor gene.	6415.25	3734.1	7422.87	0.78
212933_x_at	RPL13	6137	ribosomal protein L13		[SUMMARY:] Ribosomes, the organelles that catalyze protein synthesis, consist of a small 40S subunit and a large 60S subunit. Together these subunits are composed of 4 RNA species and approximately 80 structurally distinct proteins. This gene encodes a ribosomal protein that is a component of the 60S subunit. The protein belongs to the L13E family of ribosomal proteins. It is located in the cytoplasm. This gene is expressed at significantly higher levels in benign breast lesions than in breast carcinomas. Transcript variants derived from alternative splicing and/or alternative polyadenylation exist; these variants encode the same protein. As is typical for genes encoding ribosomal proteins, there are multiple processed pseudogenes of this gene dispersed through the genome.	3320.12	1931.9	3841.28	0.78
213084_x_at	RPL23A	6147	ribosomal protein L23a	cytosolic large ribosomal subunit (sensu Eukarya); intracellular; protein biosynthesis; rRNA binding; ribosome; structural constituent of ribosome	[SUMMARY:] Ribosomes, the organelles that catalyze protein synthesis, consist of a small 40S subunit and a large 60S subunit. Together these subunits are composed of 4 RNA species and approximately 80 structurally distinct proteins. This gene encodes a ribosomal protein that is a component of the 60S subunit. The protein belongs to the L23P family of ribosomal proteins. It is located in the cytoplasm. The protein may be one of the target molecules involved in mediating growth inhibition by interferon. In yeast, the corresponding protein binds to a specific site on the 26S rRNA. This gene is co-transcribed with the U42A, U42B, U101A, and U101B small nucleolar RNA genes, which are located in its third, first, second, and fourth introns, respectively. As is typical for genes encoding ribosomal proteins, there are multiple processed pseudogenes of this gene dispersed through the genome.	8409.67	4898.3	9732.21	0.78
200095_x_at	RPS10	6204	ribosomal protein S10	RNA binding; cytosolic small ribosomal subunit (sensu Eukarya); protein biosynthesis; structural constituent of ribosome	[SUMMARY:] Ribosomes, the organelles that catalyze protein synthesis, consist of a small 40S subunit and a large 60S subunit. Together these subunits are composed of 4 RNA species and approximately 80 structurally distinct proteins. This gene encodes a ribosomal protein that is a component of the 40S subunit. The protein belongs to the S10E family of ribosomal proteins. It is located in the cytoplasm. Variable expression of this gene in colorectal cancers compared to adjacent normal tissues has been observed, although no correlation between the level of expression and the severity of the disease has been found. As is typical for genes encoding ribosomal proteins, there are multiple processed pseudogenes of this gene dispersed through the genome.	7053.03	4137.85	8177.22	0.77
201318_s_at	MRCL3	10627	myosin regulatory light chain MRCL3			5084.5	3023.4	5915.5	0.75
204540_at	EEF1A2	1917	eukaryotic translation elongation factor 1 alpha 2	GTP binding; cytoplasm; nucleus; protein biosynthesis; protein-synthesizing GTPase activity; translation elongation factor activity; translational elongation	[SUMMARY:] This gene encodes an isoform of the alpha subunit of the elongation factor-1 complex, which is responsible for the enzymatic delivery of aminoacyl tRNAs to the ribosome. This isoform (alpha 2) is expressed in brain, heart and skeletal muscle, and the other isoform (alpha 1) is expressed in brain, placenta, lung, liver, kidney, and pancreas. This gene may be critical in the development of ovarian cancer.	4569.48	2713.75	5314.56	0.75

208692_at	RPS3	6188	ribosomal protein S3	RNA binding; cytosolic small ribosomal subunit (sensu Eukarya); intracellular; protein biosynthesis; structural constituent of ribosome	[SUMMARY:] Ribosomes, the organelles that catalyze protein synthesis, consist of a small 40S subunit and a large 60S subunit. Together these subunits are composed of 4 RNA species and approximately 80 structurally distinct proteins. This gene encodes a ribosomal protein that is a component of the 40S subunit, where it forms part of the domain where translation is initiated. The protein belongs to the S3P family of ribosomal proteins. Studies of the mouse and rat proteins have demonstrated that the protein has an extraribosomal role as an endonuclease involved in the repair of UV-induced DNA damage. The protein appears to be located in both the cytoplasm and nucleus but not in the nucleolus. Higher levels of expression of this gene in colon adenocarcinomas and adenomatous polyps compared to adjacent normal colonic mucosa have been observed. This gene is co-transcribed with the small nucleolar RNA genes U15A and U15B, which are located in its first and fifth introns, respectively. As is typical for genes encoding ribosomal proteins, there are multiple processed pseudogenes of this gene dispersed thro	3770.9	2259.4	4395.97	0.74
208834_x_at	RPL23A	6147	ribosomal protein L23a	cytosolic large ribosomal subunit (sensu Eukarya); intracellular; protein biosynthesis; rRNA binding; ribosome; structural constituent of ribosome	[SUMMARY:] Ribosomes, the organelles that catalyze protein synthesis, consist of a small 40S subunit and a large 60S subunit. Together these subunits are composed of 4 RNA species and approximately 80 structurally distinct proteins. This gene encodes a ribosomal protein that is a component of the 60S subunit. The protein belongs to the L23P family of ribosomal proteins. It is located in the cytoplasm. The protein may be one of the target molecules involved in mediating growth inhibition by interferon. In yeast, the corresponding protein binds to a specific site on the 26S rRNA. This gene is co-transcribed with the U42A, U42B, U101A, and U101B small nucleolar RNA genes, which are located in its third, first, second, and fourth introns, respectively. As is typical for genes encoding ribosomal proteins, there are multiple processed pseudogenes of this gene dispersed through the genome.	8511.5	5081.7	9913.09	0.74
213735_s_at	COX5B	1329	cytochrome c oxidase subunit Vb	cytochrome-c oxidase activity; electron transport; inner membrane; mitochondrial membrane; oxidoreductase activity; respiratory gaseous exchange	[SUMMARY:] Cytochrome C oxidase (COX) is the terminal enzyme of the mitochondrial respiratory chain. It is a multi-subunit enzyme complex that couples the transfer of electrons from cytochrome c to molecular oxygen and contributes to a proton electrochemical gradient across the inner mitochondrial membrane. The complex consists of 13 mitochondrial- and nuclear-encoded subunits. The mitochondrially-encoded subunits perform the electron transfer and proton pumping activities. The functions of the nuclear-encoded subunits are unknown but they may play a role in the regulation and assembly of the complex. This gene encodes the nuclear-encoded subunit Vb of the human mitochondrial respiratory chain enzyme.	4595.83	2751.57	5356.56	0.74
210299_s_at	FHL1	2273	four and a half LIM domains 1	cell differentiation; cell growth; cellular_component unknown; molecular_function unknown; muscle		3439.43	2071	4014.81	0.73
200725_x_at	RPL10	6134	ribosomal protein L10		[SUMMARY:] Ribosomes, the organelles that catalyze protein synthesis, consist of a small 40S subunit and a large 60S subunit. Together these subunits are composed of 4 RNA species and approximately 80 structurally distinct proteins. This gene encodes a ribosomal protein that is a component of the 60S subunit. The protein belongs to the L10E family of ribosomal proteins. It is located in the cytoplasm. In vitro studies have shown that the chicken protein can bind to c-Jun and can repress c-Jun-mediated transcriptional activation, but these activities have not been demonstrated in vivo. This gene was initially identified as a candidate for a Wilms tumor suppressor gene, but later studies determined that this gene is not involved in the suppression of Wilms tumor. This gene has been referred to as 'laminin receptor homolog' because a chimeric transcript consisting of sequence from this gene and sequence from the laminin receptor gene was isolated; however, it is not believed that this gene encodes a laminin receptor. Transcript variants utilizing alternative polyA signals exist. The variant with the	4903.12	2982.28	5738.87	0.72
213738_s_at	ATP5A1	498	ATP synthase, H+ transporting, mitochondrial F1 complex, alpha subunit, isoform 1, cardiac muscle	ATP binding; ATP biosynthesis; ATP-binding and phosphorylation-dependent chloride channel activity; hydrogen ion transporter activity; hydrolase activity; membrane fraction; mitochondrion; proton transport; proton-transporting ATP synthase complex (sensu Eukarya); proton-		3460.23	2120.75	4058.42	0.71
200031_s_at	RPS11	6205	ribosomal protein S11	intracellular; protein biosynthesis; rRNA binding; ribosome; structural constituent of ribosome	[SUMMARY:] Ribosomes, the organelles that catalyze protein synthesis, consist of a small 40S subunit and a large 60S subunit. Together these subunits are composed of 4 RNA species and approximately 80 structurally distinct proteins. This gene encodes a ribosomal protein that is a component of the 40S subunit. The protein belongs to the S17P family of ribosomal proteins. It is located in the cytoplasm. The gene product of the E. coli ortholog (ribosomal protein S17) is thought to be involved in the recognition of termination codons. This gene is co-transcribed with a small nucleolar RNA gene, which is located in its third intron. As is typical for genes encoding ribosomal proteins, there are multiple processed pseudogenes of this gene dispersed through the genome.	6389.25	3958.45	7516.1	0.69
211542_x_at						8419.65	5235.38	9914.62	0.69
208825_x_at	RPL23A	6147	ribosomal protein L23a	cytosolic large ribosomal subunit (sensu Eukarya); intracellular; protein biosynthesis; rRNA binding; ribosome; structural constituent of ribosome	[SUMMARY:] Ribosomes, the organelles that catalyze protein synthesis, consist of a small 40S subunit and a large 60S subunit. Together these subunits are composed of 4 RNA species and approximately 80 structurally distinct proteins. This gene encodes a ribosomal protein that is a component of the 60S subunit. The protein belongs to the L23P family of ribosomal proteins. It is located in the cytoplasm. The protein may be one of the target molecules involved in mediating growth inhibition by interferon. In yeast, the corresponding protein binds to a specific site on the 26S rRNA. This gene is co-transcribed with the U42A, U42B, U101A, and U101B small nucleolar RNA genes, which are located in its third, first, second, and fourth introns, respectively. As is typical for genes encoding ribosomal proteins, there are multiple processed pseudogenes of this gene dispersed through the genome.	8253.6	5141.7	9724.14	0.68

200909_s_at	RPLP2	6181	ribosomal protein, large P2	RNA binding; cytosolic large ribosomal subunit (sensu Eukarya); intracellular; protein biosynthesis; ribosome; structural constituent of ribosome; translational elongation	[SUMMARY:] Ribosomes, the organelles that catalyze protein synthesis, consist of a small 40S subunit and a large 60S subunit. Together these subunits are composed of 4 RNA species and approximately 80 structurally distinct proteins. This gene encodes a ribosomal phosphoprotein that is a component of the 60S subunit. The protein, which is a functional equivalent of the E. coli L7/L12 ribosomal protein, belongs to the L12P family of ribosomal proteins. It plays an important role in the elongation step of protein synthesis. Unlike most ribosomal proteins, which are basic, the encoded protein is acidic. Its C-terminal end is nearly identical to the C-terminal ends of the ribosomal phosphoproteins P0 and P1. The P2 protein can interact with P0 and P1 to form a pentameric complex consisting of P1 and P2 dimers, and a P0 monomer. The protein is located in the cytoplasm. As is typical for genes encoding ribosomal proteins, there are multiple processed pseudogenes of this gene dispersed through the genome.	3456.3	2165.3	4078.55	0.67
204083_s_at	TPM2	7169	tropomyosin 2 (beta)	actin binding; cytoskeleton; muscle development; muscle thin filament tropomyosin; structural constituent of		4147.05	2615.7	4903.05	0.66
200018_at	RPS13	6207	ribosomal protein S13		[SUMMARY:] Ribosomes, the organelles that catalyze protein synthesis, consist of a small 40S subunit and a large 60S subunit. Together these subunits are composed of 4 RNA species and approximately 80 structurally distinct proteins. This gene encodes a ribosomal protein that is a component of the 40S subunit. The protein belongs to the S15P family of ribosomal proteins. It is located in the cytoplasm. The protein has been shown to bind to the 5.8S rRNA in rat. The gene product of the E. coli ortholog (ribosomal protein S15) functions at early steps in ribosome assembly. This gene is co-transcribed with two U14 small nucleolar RNA genes, which are located in its third and fifth introns. As is typical for genes encoding ribosomal proteins, there are multiple processed pseudogenes of this gene dispersed through the genome.	5461.55	3475.73	6473.73	0.65
200718_s_at	SKP1A	6500	S-phase kinase-associated protein 1A (p19A)	biological_process unknown; cellular_component unknown; molecular_function unknown	[SUMMARY:] This gene encodes an F-box protein which functions as a substrate recognition component of the SCF ubiquitin ligase complex. It binds to cyclin F, S-phase kinase-associated protein 2, and other regulatory proteins involved in ubiquitin proteolysis through an F-box motif. The encoded protein also collaborates with a network of proteins to control beta-catenin levels and affects the activity level of beta-catenin dependent TCF transcription factors. Studies have also characterized the protein as an RNA polymerase II elongation factor. Alternative splicing of this gene results in two transcript variants. A related pseudogene has been identified on chromosome 7.	2773.52	1766.93	3288.54	0.65
203034_s_at	RPL27A	6157	ribosomal protein L27a		[SUMMARY:] Ribosomes, the organelles that catalyze protein synthesis, consist of a small 40S subunit and a large 60S subunit. Together these subunits are composed of 4 RNA species and approximately 80 structurally distinct proteins. This gene encodes a ribosomal protein that is a component of the 60S subunit. The protein belongs to the L15P family of ribosomal proteins. It is located in the cytoplasm. Variable expression of this gene in colorectal cancers compared to adjacent normal tissues has been observed, although no correlation between the level of expression and the severity of the disease has been found. As is typical for genes encoding ribosomal proteins, multiple processed pseudogenes derived from this gene are dispersed through the genome.	6857.1	4373.12	8132.9	0.65
208845_at	VDAC3	7419	voltage-dependent anion channel 3	adenine transport; anion transport; integral to plasma membrane; mitochondrial outer membrane; mitochondrion; voltage-dependent anion channel porin activity; voltage-		3349.95	2136.72	3973.38	0.65
200650_s_at	LDHA	3939	lactate dehydrogenase A		[SUMMARY:] Lactate dehydrogenase A catalyzes the conversion of L-lactate and NAD to pyruvate and NADH in the final step of anaerobic glycolysis. LDHA is found predominantly in muscle tissue and belongs to the lactate dehydrogenase family. Mutations in LDHA have been linked to exertional myoglobinuria.	3888.68	2497.15	4621.42	0.64
202343_x_at	COX5B	1329	cytochrome c oxidase subunit Vb	cytochrome-c oxidase activity; electron transport; inner membrane; mitochondrial membrane; oxidoreductase activity; respiratory gaseous exchange	[SUMMARY:] Cytochrome C oxidase (COX) is the terminal enzyme of the mitochondrial respiratory chain. It is a multi-subunit enzyme complex that couples the transfer of electrons from cytochrome c to molecular oxygen and contributes to a proton electrochemical gradient across the inner mitochondrial membrane. The complex consists of 13 mitochondrial- and nuclear-encoded subunits. The mitochondrially-encoded subunits perform the electron transfer and proton pumping activities. The functions of the nuclear-encoded subunits are unknown but they may play a role in the regulation and assembly of the complex. This gene encodes the nuclear-encoded subunit Vb of the human mitochondrial respiratory chain enzyme.	3463.33	2223.95	4115.89	0.64
200030_s_at	SLC25A3	5250	solute carrier family 25 (mitochondrial carrier; phosphate carrier), member 3	binding; energy pathways; integral to plasma membrane; mitochondrial inner membrane; mitochondrion; phosphate carrier activity; symporter activity; transport	[SUMMARY:] The phosphate carrier (SLC25A3) catalyzes the transport of phosphate into the mitochondrial matrix, either by proton cotransport or in exchange for hydroxyl ions. The protein contains three related segments arranged in tandem which are related to those found in other characterized members of the mitochondrial carrier family. Both the N-terminal and C-terminal regions of the phosphate carrier protrude toward the cytosol. Two transcripts containing either exon IIIA or exon IIIB have been isolated. The variant containing exon IIIA is predominant in heart and liver, while that containing IIIB has greater expression in lung tissue.	3770.55	2440.73	4491.57	0.63
211939_x_at	BTF3	689	basic transcription			2406.95	1552.6	2864.26	0.63
211275_s_at	GYG	2992	glycogenin			2411.38	1580.45	2883.15	0.61
213011_s_at	TPI1	7167	triosephosphate			2547.95	1672.43	3047.79	0.61
204284_at	PPP1R3C	5507	protein phosphatase 1, regulatory (inhibitor) subunit 3C	hydrolase activity; phosphoprotein phosphatase activity; protein phosphatase type 1 activity	[SUMMARY:] Protein phosphatase-1 (PP1; see MIM 176875) participates in the regulation of a wide variety of cellular functions by reversible protein phosphorylation. The ability of PP1 to regulate diverse functions resides in its capacity to interact with a variety of regulatory subunits that may target PP1 to specific subcellular locations, modulate its substrate specificity, and allow its activity to be responsive to extracellular signals. Several targeting subunits of PP1 have been identified, including PPP1R5, the glycogen-binding subunits PPP1R3 (MIM 600917) and PPP1R4, and the nuclear inhibitor of PP1 (PPP1R8; MIM 602636).[supplied by OMIM]	4351.25	2873.42	5214.4	0.6
219645_at	CASQ1	844	calsequestrin 1 (fast-twitch, skeletal muscle)	calcium ion storage activity; mitochondrial matrix; muscle development; smooth endoplasmic reticulum	[SUMMARY:] The protein encoded by this gene is a mitochondrial calcium-binding protein located in the luminal space of the terminal cisternae of the sarcoplasmic reticulum. The protein binds and putatively stores calcium ions. The protein is absent in patients with Duchenne and Becker types of muscular dystrophy.	4541.85	3027.3	5458.29	0.59
200099_s_at						5502.7	3707.05	6634.9	0.57

212361_s_at	ATP2A2	488	ATPase, Ca++ transporting, cardiac muscle, slow twitch 2		[SUMMARY:] This gene encodes one of the SERCA Ca(2+)-ATPases, which are intracellular pumps located in the sarcoplasmic or endoplasmic reticula of muscle cells. This enzyme catalyzes the hydrolysis of ATP coupled with the translocation of calcium from the cytosol to the sarcoplasmic reticulum lumen, and is involved in regulation of the contraction/relaxation cycle. Mutations in this gene cause Darier-White disease, also known as keratosis follicularis, an autosomal dominant skin disorder characterized by loss of adhesion between epidermal cells and abnormal keratinization. Alternative splicing results in two transcript variants encoding different isoforms.	3888.9	2651.85	4707	0.55
201968_s_at	PGM1	5236	phosphoglucomutase 1	carbohydrate metabolism; cytoplasm; glucose metabolism; isomerase activity; magnesium ion binding;		4405.82	3031.33	5347.92	0.54
202021_x_at	SUI1	10209	putative translation initiation factor	cell growth and/or maintenance; cellular_component unknown; cytoplasm; regulation of protein biosynthesis; regulation of translation; regulation of translational initiation;		4388.52	3039.62	5338.4	0.53
214003_x_at	RPS20	6224	ribosomal protein S20	RNA binding; cytosolic small ribosomal subunit (sensu Eukarya); intracellular; protein biosynthesis; structural constituent of ribosome	[SUMMARY:] Ribosomes, the organelles that catalyze protein synthesis, consist of a small 40S subunit and a large 60S subunit. Together these subunits are composed of 4 RNA species and approximately 80 structurally distinct proteins. This gene encodes a ribosomal protein that is a component of the 40S subunit. The protein belongs to the S10P family of ribosomal proteins. It is located in the cytoplasm. This gene is co-transcribed with the small nucleolar RNA gene U54, which is located in its second intron. As is typical for genes encoding ribosomal proteins, there are multiple processed pseudogenes of this gene dispersed through the genome.	6156.52	4273.12	7494.16	0.53
202649_x_at	RPS19	6223	ribosomal protein S19		[SUMMARY:] Ribosomes, the organelles that catalyze protein synthesis, consist of a small 40S subunit and a large 60S subunit. Together these subunits are composed of 4 RNA species and approximately 80 structurally distinct proteins. This gene encodes a ribosomal protein that is a component of the 40S subunit. The protein belongs to the S19E family of ribosomal proteins. It is located in the cytoplasm. Mutations in this gene cause Diamond-Blackfan anemia (DBA), a constitutional erythroblastopenia characterized by absent or decreased erythroid precursors, in a subset of patients. This suggests a possible extra-ribosomal function for this gene in erythropoietic differentiation and proliferation, in addition to its ribosomal function. Higher expression levels of this gene in some primary colon carcinomas compared to matched normal colon tissues has been observed. As is typical for genes encoding ribosomal proteins, there are multiple processed pseudogenes of this gene dispersed through the genome.	2547.42	1778.12	3106.62	0.52
210338_s_at	HSPA8	3312	heat shock 70kDa protein 8	ATP binding; ATPase activity, coupled; heat shock protein activity; intracellular; protein folding	[SUMMARY:] The product encoded by this gene belongs to the heat shock protein 70 family which contains both heat-inducible and constitutively expressed members. The latter are called heat-shock cognate proteins. This gene encodes a heat-shock cognate protein. This protein binds to nascent polypeptides to facilitate correct folding. It also functions as an ATPase in the disassembly of clathrin-coated vesicles during transport of membrane components through the cell. Two alternatively spliced variants have been characterized to date.	2490.92	1734.53	3035.34	0.52
200781_s_at	RPS15A	6210	ribosomal protein S15a	3'-5'-exoribonuclease activity; RNA binding; RNA processing	[SUMMARY:] Ribosomes, the organelles that catalyze protein synthesis, consist of a small 40S subunit and a large 60S subunit. Together these subunits are composed of 4 RNA species and approximately 80 structurally distinct proteins. This gene encodes a ribosomal protein that is a component of the 40S subunit. The protein belongs to the S8P family of ribosomal proteins. It is located in the cytoplasm. As is typical for genes encoding ribosomal proteins, there are multiple processed pseudogenes of this gene dispersed through the genome.	5276.65	3711.2	6451.05	0.51
202110_at	COX7B	1349	cytochrome c oxidase subunit VIIb	cytochrome-c oxidase activity; mitochondrion; oxidoreductase activity	[SUMMARY:] Cytochrome c oxidase (COX), the terminal component of the mitochondrial respiratory chain, catalyzes the electron transfer from reduced cytochrome c to oxygen. This component is a heteromeric complex consisting of 3 catalytic subunits encoded by mitochondrial genes and multiple structural subunits encoded by nuclear genes. The mitochondrially-encoded subunits function in electron transfer, and the nuclear-encoded subunits may function in the regulation and assembly of the complex. This nuclear gene encodes subunit VIIb, which is highly similar to bovine COX VIIb protein and is found in all tissues. This gene may have several pseudogenes on chromosomes 1, 2, 20 and 22, respectively.	3407.67	2399.7	4167.83	0.51
213414_s_at	RPS19	6223	ribosomal protein S19		[SUMMARY:] Ribosomes, the organelles that catalyze protein synthesis, consist of a small 40S subunit and a large 60S subunit. Together these subunits are composed of 4 RNA species and approximately 80 structurally distinct proteins. This gene encodes a ribosomal protein that is a component of the 40S subunit. The protein belongs to the S19E family of ribosomal proteins. It is located in the cytoplasm. Mutations in this gene cause Diamond-Blackfan anemia (DBA), a constitutional erythroblastopenia characterized by absent or decreased erythroid precursors, in a subset of patients. This suggests a possible extra-ribosomal function for this gene in erythropoietic differentiation and proliferation, in addition to its ribosomal function. Higher expression levels of this gene in some primary colon carcinomas compared to matched normal colon tissues has been observed. As is typical for genes encoding ribosomal proteins, there are multiple processed pseudogenes of this gene dispersed through the genome.	3276.45	2301.58	4004.04	0.51
217773_s_at	NDUFA4	4697	NADH dehydrogenase (ubiquinone) 1 alpha subcomplex, 4, 9kDa	NADH dehydrogenase (ubiquinone) activity; NADH dehydrogenase activity; mitochondrion; oxidoreductase activity	[SUMMARY:] The protein encoded by this gene belongs to the complex I 9kDa subunit family. Mammalian complex I of mitochondrial respiratory chain is composed of 45 different subunits. This protein has NADH dehydrogenase activity and oxidoreductase activity. It transfers electrons from NADH to the respiratory chain. The immediate electron acceptor for the enzyme is believed to be ubiquinone.	3445.1	2421.9	4211.21	0.51
204102_s_at	EEF2	1938	eukaryotic translation elongation factor 2		[SUMMARY:] This gene encodes a member of the GTP-binding translation elongation factor family. This protein is an essential factor for protein synthesis. It promotes the GTP-dependent translocation of the nascent protein chain from the A-site to the P-site of the ribosome. This protein is completely inactivated by EF-2 kinase phosporylation.	2908.48	2057.73	3562.79	0.5

200019_s_at	FAU	2197	Finkel-Biskis-Reilly murine sarcoma virus (FBR-MuSV) ubiquitously expressed (fox derived); ribosomal protein S30	RNA binding; protein biosynthesis; ribosome; structural constituent of ribosome	[SUMMARY:] This gene is the cellular homolog of the fox sequence in the Finkel-Biskis-Reilly murine sarcoma virus (FBR-MuSV). It encodes a fusion protein consisting of the ubiquitin-like protein fubi at the N terminus and ribosomal protein S30 at the C terminus. It has been proposed that the fusion protein is post-translationally processed to generate free fubi and free ribosomal protein S30. Fubi is a member of the ubiquitin family, and ribosomal protein S30 belongs to the S30E family of ribosomal proteins. Whereas the function of fubi is currently unknown, ribosomal protein S30 is a component of the 40S subunit of the cytoplasmic ribosome. Pseudogenes derived from this gene are present in the genome. Similar to ribosomal protein S30, ribosomal proteins S27a and L40 are synthesized as fusion proteins with ubiquitin.	2449.9	1747.93	3009.53	0.49
204173_at	MLC1SA	1E+05	myosin light chain 1 slow a	calcium ion binding; muscle development; muscle myosin; myosin; structural constituent of muscle	[SUMMARY:] Myosin is a hexameric ATPase cellular motor protein. It is composed of two heavy chains, two nonphosphorylatable alkali light chains, and two phosphorylatable regulatory light chains. This gene encodes a myosin alkali light chain expressed in both slow-twitch skeletal muscle and in nonmuscle tissue.	2325	1657.08	2855.09	0.49
208929_x_at	RPL13	6137	ribosomal protein L13		[SUMMARY:] Ribosomes, the organelles that catalyze protein synthesis, consist of a small 40S subunit and a large 60S subunit. Together these subunits are composed of 4 RNA species and approximately 80 structurally distinct proteins. This gene encodes a ribosomal protein that is a component of the 60S subunit. The protein belongs to the L13E family of ribosomal proteins. It is located in the cytoplasm. This gene is expressed at significantly higher levels in benign breast lesions than in breast carcinomas. Transcript variants derived from alternative splicing and/or alternative polyadenylation exist; these variants encode the same protein. As is typical for genes encoding ribosomal proteins, there are multiple processed pseudogenes of this gene dispersed through the genome.	3037.1	2165.9	3730.29	0.49
200651_at	GNB2L1	10399	guanine nucleotide binding protein (G protein), beta			3095.55	2219.8	3809.19	0.48
200822_x_at	TPI1	7167	triosephosphate			2833.57	2038.4	3490.59	0.48
203663_s_at	COX5A	9377	cytochrome c oxidase subunit Va	cytochrome-c oxidase activity; electron transport; electron transporter activity; inner membrane; mitochondrion; oxidoreductase activity	[SUMMARY:] Cytochrome c oxidase (COX) is the terminal enzyme of the mitochondrial respiratory chain. It is a multi-subunit enzyme complex that couples the transfer of electrons from cytochrome c to molecular oxygen and contributes to a proton electrochemical gradient across the inner mitochondrial membrane. The complex consists of 13 mitochondrial- and nuclear-encoded subunits. The mitochondrially-encoded subunits perform the electron transfer of proton pumping activities. The functions of the nuclear-encoded subunits are unknown but they may play a role in the regulation and assembly of the complex. This gene encodes the nuclear-encoded subunit Va of the human mitochondrial respiratory chain enzyme. A pseudogene COX5AP1 has been found in chromosome 14q22.	2585.65	1848.47	3178.43	0.48
211927_x_at	EEF1G	1937	eukaryotic translation elongation factor 1 gamma	eukaryotic translation elongation factor 1 complex; intracellular; nucleic acid binding; protein biosynthesis; translation elongation factor activity; translational	[SUMMARY:] This gene encodes a subunit of the elongation factor-1 complex, which is responsible for the enzymatic delivery of aminoacyl tRNAs to the ribosome. This subunit contains an N-terminal glutathione transferase domain, which may be involved in regulating the assembly of multisubunit complexes containing this elongation factor and aminoacyl-tRNA synthetases.	5801.6	4152.1	7134.32	0.48
200834_s_at	RPS21	6227	ribosomal protein S21	RNA binding; cytosolic small ribosomal subunit (sensu Eukarya); intracellular; protein biosynthesis; ribosome; structural constituent of ribosome	[SUMMARY:] Ribosomes, the organelles that catalyze protein synthesis, consist of a small 40S subunit and a large 60S subunit. Together these subunits are composed of 4 RNA species and approximately 80 structurally distinct proteins. This gene encodes a ribosomal protein that is a component of the 40S subunit. The protein belongs to the S21E family of ribosomal proteins. It is located in the cytoplasm. Alternative splice variants that encode different protein isoforms have been described, but their existence has not been verified. As is typical for genes encoding ribosomal proteins, there are multiple processed pseudogenes of this gene dispersed through the genome.	4160.15	3016.93	5138.94	0.46
211025_x_at	COX5B	1329	cytochrome c oxidase subunit Vb	cytochrome-c oxidase activity; electron transport; inner membrane; mitochondrial membrane; oxidoreductase activity; respiratory gaseous exchange	[SUMMARY:] Cytochrome C oxidase (COX) is the terminal enzyme of the mitochondrial respiratory chain. It is a multi-subunit enzyme complex that couples the transfer of electrons from cytochrome c to molecular oxygen and contributes to a proton electrochemical gradient across the inner mitochondrial membrane. The complex consists of 13 mitochondrial- and nuclear-encoded subunits. The mitochondrially-encoded subunits perform the electron transfer and proton pumping activities. The functions of the nuclear-encoded subunits are unknown but they may play a role in the regulation and assembly of the complex. This gene encodes the nuclear-encoded subunit Vb of the human mitochondrial respiratory chain enzyme.	2436.9	1778.93	3017.13	0.45
212130_x_at	SUI1	10209	putative translation initiation factor	cell growth and/or maintenance; cellular_component unknown; cytoplasm; regulation of protein biosynthesis; regulation of translation; regulation of translational initiation;		4523.15	3310.07	5604.95	0.45
221700_s_at	UBA52	7311	ubiquitin A-52 residue ribosomal protein fusion product 1	intracellular; protein biosynthesis; ribosome; structural constituent of ribosome	[SUMMARY:] Ubiquitin is a highly conserved nuclear and cytoplasmic protein that has a major role in targeting cellular proteins for degradation by the 26S proteosome. It is also involved in the maintenance of chromatin structure, the regulation of gene expression, and the stress response. Ubiquitin is synthesized as a precursor protein consisting of either polyubiquitin chains or a single ubiquitin moiety fused to an unrelated protein. This gene encodes a fusion protein consisting of ubiquitin at the N terminus and ribosomal protein L40 at the C terminus. When expressed in yeast, the protein is post-translationally processed, generating free ubiquitin monomer and the 52-amino acid ribosomal protein L40. Ribosomal protein L40 is a component of the 60S subunit of the ribosome and belongs to the L40E family of ribosomal proteins. It contains a zinc finger-like domain and is located in the cytoplasm. Multiple processed pseudogenes derived from this gene are present in the genome. As with ribosomal protein L40, ribosomal protein S27a is also synthesized as a fusion protein with ubiquitin; similarly, ribosomal protein S30 i	2585.3	1894.92	3205.39	0.45
204892_x_at	EEF1A1	1915	eukaryotic translation elongation factor 1 alpha 1	GTP binding; cytoplasm; eukaryotic translation elongation factor 1 complex; oncogenesis; regulation of cell shape; regulation of translation; translational elongation	[SUMMARY:] This gene encodes an isoform of the alpha subunit of the elongation factor-1 complex, which is responsible for the enzymatic delivery of aminoacyl tRNAs to the ribosome. This isoform (alpha 1) is expressed in brain, placenta, lung, liver, kidney, and pancreas, and the other isoform (alpha 2) is expressed in brain, heart and skeletal muscle. This isoform is identified as an autoantigen in 66% of patients with Felty's syndrome. This gene has been found to have multiple copies on many chromosomes, some of which, if not all, represent different pseudogenes.	2996.92	2210.85	3724.17	0.44

203861_s_at	ACTN2	88	actinin, alpha 2	actin binding; actin filament; calcium ion binding; cytoskeleton; protein binding; structural constituent of muscle	[SUMMARY:] Alpha actinins belong to the spectrin gene superfamily which represents a diverse group of cytoskeletal proteins, including the alpha and beta spectrins and dystrophins. Alpha actinin is an actin-binding protein with multiple roles in different cell types. In nonmuscle cells, the cytoskeletal isoform is found along microfilament bundles and adherens-type junctions, where it is involved in binding actin to the membrane. In contrast, skeletal, cardiac, and smooth muscle isoforms are localized to the Z-disc and analogous dense bodies, where they help anchor the myofibrillar actin filaments. This gene encodes a muscle-specific, alpha actinin isoform that is expressed in both skeletal and cardiac muscles. Transcript variants resulting from the use of multiple poly_A sites have been observed.	3181.98	2375.2	3970.71	0.42
212227_x_at	SUI1	10209	putative translation initiation factor	cell growth and/or maintenance; cellular_component unknown; cytoplasm; regulation of protein biosynthesis; regulation of translation; regulation of translational initiation;		4522.33	3378.05	5644.7	0.42
214365_at	TPM3	7170	tropomyosin 3	actin binding; cell growth and/or maintenance; cytoskeleton; molecular_function unknown; muscle development; muscle thin filament tropomyosin;		3368.07	2532.8	4214.14	0.41
206559_x_at						3050.25	2315.82	3829.76	0.4
221798_x_at	RPS2	6187	ribosomal protein S2	RNA binding; cytosolic small ribosomal subunit (sensu Eukarya); intracellular; protein biosynthesis; small ribosomal subunit; structural constituent of ribosome	[SUMMARY:] Ribosomes, the organelles that catalyze protein synthesis, consist of a small 40S subunit and a large 60S subunit. Together these subunits are composed of 4 RNA species and approximately 80 structurally distinct proteins. This gene encodes a ribosomal protein that is a component of the 40S subunit. The protein belongs to the S5P family of ribosomal proteins. It is located in the cytoplasm. This gene shares sequence similarity with mouse LLRep3. It is co-transcribed with the small nucleolar RNA gene U64, which is located in its third intron. As is typical for genes encoding ribosomal proteins, there are multiple processed pseudogenes of this gene dispersed through the genome.	5617.8	4261.4	7051.19	0.4
200933_x_at	RPS4X	6191	ribosomal protein S4, X-linked		[SUMMARY:] Cytoplasmic ribosomes, organelles that catalyze protein synthesis, consist of a small 40S subunit and a large 60S subunit. Together these subunits are composed of 4 RNA species and approximately 80 structurally distinct proteins. This gene encodes ribosomal protein S4, a component of the 40S subunit. Ribosomal protein S4 is the only ribosomal protein known to be encoded by more than one gene, namely this gene and ribosomal protein S4, Y-linked (RPS4Y). The 2 isoforms encoded by these genes are not identical, but are functionally equivalent. Ribosomal protein S4 belongs to the S4E family of ribosomal proteins. This gene is not subject to X-inactivation. It has been suggested that haploinsufficiency of the ribosomal protein S4 genes plays a role in Turner syndrome; however, this hypothesis is controversial. As is typical for genes encoding ribosomal proteins, there are multiple processed pseudogenes of this gene dispersed through the genome.	4088.3	3117.25	5141.15	0.39
202222_s_at	DES	1674	desmin		[SUMMARY:] This gene encodes a muscle-specific class III intermediate filament. Homopolymers of this protein form a stable intracytoplasmic filamentous network connecting myofibrils to each other and to the plasma membrane. Mutations in this gene are associated with desmin-related myopathy, a familial cardiac and skeletal myopathy (CSM), and with distal myopathies.	4994.5	3823.32	6289.9	0.39
202698_x_at	COX4I1	1327	cytochrome c oxidase subunit IV isoform 1	cytochrome-c oxidase activity; electron transport; energy pathways; inner membrane; mitochondrion; oxidoreductase activity	[SUMMARY:] Cytochrome c oxidase (COX) is the terminal enzyme of the mitochondrial respiratory chain. It is a multi-subunit enzyme complex that couples the transfer of electrons from cytochrome c to molecular oxygen and contributes to a proton electrochemical gradient across the inner mitochondrial membrane. The complex consists of 13 mitochondrial- and nuclear-encoded subunits. The mitochondrially-encoded subunits perform the electron transfer and proton pumping activities. The functions of the nuclear-encoded subunits are unknown but they may play a role in the regulation and assembly of the complex. This gene encodes the nuclear-encoded subunit IV isoform 1 of the human mitochondrial respiratory chain enzyme. It is located at the 3' of the NOC4 (neighbor of COX4) gene in a head-to-head orientation, and shares a promoter with it.	2667.27	2031.27	3352.68	0.39
200022_at	RPL18	6141	ribosomal protein L18		[SUMMARY:] Ribosomes, the organelles that catalyze protein synthesis, consist of a small 40S subunit and a large 60S subunit. Together these subunits are composed of 4 RNA species and approximately 80 structurally distinct proteins. This gene encodes a ribosomal protein that is a component of the 60S subunit. The protein belongs to the L18E family of ribosomal proteins. It is located in the cytoplasm. As is typical for genes encoding ribosomal proteins, there are multiple processed pseudogenes of this gene dispersed through the genome.	2372.68	1830.5	2996.72	0.37
200062_s_at	RPL30	6156	ribosomal protein L30		[SUMMARY:] Ribosomes, the organelles that catalyze protein synthesis, consist of a small 40S subunit and a large 60S subunit. Together these subunits are composed of 4 RNA species and approximately 80 structurally distinct proteins. This gene encodes a ribosomal protein that is a component of the 60S subunit. The protein belongs to the L30E family of ribosomal proteins. It is located in the cytoplasm. This gene is co-transcribed with the U72 small nucleolar RNA gene, which is located in its fourth intron. As is typical for genes encoding ribosomal proteins, there are multiple processed pseudogenes of this gene dispersed through the genome.	5356.65	4158.75	6781.51	0.37
200077_s_at	OAZ1	4946	ornithine decarboxylase antizyme 1		[SUMMARY:] Ornithine decarboxylase catalyzes the conversion of ornithine to putrescine in the first and apparently rate-limiting step in polyamine biosynthesis. The ornithine decarboxylase antizymes play a role in the regulation of polyamine synthesis by binding to and inhibiting ornithine decarboxylase. Antizyme expression is auto-regulated by polyamine-enhanced translational frameshifting. The antizyme encoded by this gene inhibits ornithine decarboxylase and accelerates its degradation.	2404.85	1872.97	3048.17	0.36

204570_at	COX7A1	1346	cytochrome c oxidase subunit VIIa polypeptide 1 (muscle)		[SUMMARY:] Cytochrome c oxidase (COX), the terminal component of the mitochondrial respiratory chain, catalyzes the electron transfer from reduced cytochrome c to oxygen. This component is a heteromeric complex consisting of 3 catalytic subunits encoded by mitochondrial genes and multiple structural subunits encoded by nuclear genes. The mitochondrially-encoded subunits function in electron transfer, and the nuclear-encoded subunits may function in the regulation and assembly of the complex. This nuclear gene encodes polypeptide 1 (muscle isoform) of subunit VIIa and the polypeptide 1 is present only in muscle tissues. Other polypeptides of subunit VIIa are present in both muscle and nonmuscle tissues, and are encoded by different genes.	5488.03	4278.75	6958.89	0.36
206116_s_at	TPM1	7168	tropomyosin 1 (alpha)	actin binding; biological_process unknown; cellular_component unknown; cytoskeleton; molecular_function unknown; muscle development; muscle thin filament tropomyosin; regulation of heart rate; regulation of muscle contraction; structural	[SUMMARY:] Tropomyosins are ubiquitous proteins of 35 to 45 kD associated with the actin filaments of myofibrils and stress fibers. In vertebrates, 4 known tropomyosin genes code for diverse isoforms that are expressed in a tissue-specific manner and regulated by an alternative splicing mechanism (Lees-Miller and Helfman, 1991 [PubMed 1796905]). The vertebrate alpha-tropomyosin gene consists of 15 exons; 5 exons are found in all transcripts, while 10 exons are alternatively used in different alpha-tropomyosin RNAs (Lees-Miller and Helfman, 1991 [PubMed 1796905]). The striated muscle isoform is expressed in both cardiac and skeletal muscle tissues.[supplied by OMIM]	4041.05	3142.75	5119.27	0.36
200741_s_at	RPS27	6232	ribosomal protein S27 (metallopanstimulin 1)		[SUMMARY:] Ribosomes, the organelles that catalyze protein synthesis, consist of a small 40S subunit and a large 60S subunit. Together these subunits are composed of 4 RNA species and approximately 80 structurally distinct proteins. This gene encodes a ribosomal protein that is a component of the 40S subunit. The protein belongs to the S27E family of ribosomal proteins. It contains a C4-type zinc finger domain that can bind to zinc. The encoded protein has been shown to be able to bind to nucleic acid. It is located in the cytoplasm as a ribosomal component, but it has also been detected in the nucleus. Studies in rat indicate that ribosomal protein S27 is located near ribosomal protein S18 in the 40S subunit and is covalently linked to translation initiation factor eIF3. As is typical for genes encoding ribosomal proteins, there are multiple processed pseudogenes of this gene dispersed through the genome.	4844.25	3795.5	6154.07	0.35
208904_s_at	RPS28	6234	ribosomal protein S28	RNA binding; cytosolic small ribosomal subunit (sensu Eukarya); intracellular; protein biosynthesis; ribosome; structural constituent of ribosome	[SUMMARY:] Ribosomes, the organelles that catalyze protein synthesis, consist of a small 40S subunit and a large 60S subunit. Together these subunits are composed of 4 RNA species and approximately 80 structurally distinct proteins. This gene encodes a ribosomal protein that is a component of the 40S subunit. The protein belongs to the S28E family of ribosomal proteins. It is located in the cytoplasm. As is typical for genes encoding ribosomal proteins, there are multiple processed pseudogenes of this gene dispersed through the genome.	4198.1	3293.23	5335.67	0.35
201258_at	RPS16	6217	ribosomal protein S16		[SUMMARY:] Ribosomes, the organelles that catalyze protein synthesis, consist of a small 40S subunit and a large 60S subunit. Together these subunits are composed of 4 RNA species and approximately 80 structurally distinct proteins. This gene encodes a ribosomal protein that is a component of the 40S subunit. The protein belongs to the S9P family of ribosomal proteins. It is located in the cytoplasm. As is typical for genes encoding ribosomal proteins, there are multiple processed pseudogenes of this gene dispersed through the genome.	2981.42	2356.28	3800.12	0.34
200926_at	RPS23	6228	ribosomal protein S23		[SUMMARY:] Ribosomes, the organelles that catalyze protein synthesis, consist of a small 40S subunit and a large 60S subunit. Together these subunits are composed of 4 RNA species and approximately 80 structurally distinct proteins. This gene encodes a ribosomal protein that is a component of the 40S subunit. The protein belongs to the S12P family of ribosomal proteins. It is located in the cytoplasm. The protein shares significant amino acid similarity with S. cerevisiae ribosomal protein S28. As is typical for genes encoding ribosomal proteins, there are multiple processed pseudogenes of this gene dispersed through the genome.	5810.47	4615.23	7420.37	0.33
212734_x_at	RPL13	6137	ribosomal protein L13		[SUMMARY:] Ribosomes, the organelles that catalyze protein synthesis, consist of a small 40S subunit and a large 60S subunit. Together these subunits are composed of 4 RNA species and approximately 80 structurally distinct proteins. This gene encodes a ribosomal protein that is a component of the 60S subunit. The protein belongs to the L13E family of ribosomal proteins. It is located in the cytoplasm. This gene is expressed at significantly higher levels in benign breast lesions than in breast carcinomas. Transcript variants derived from alternative splicing and/or alternative polyadenylation exist; these variants encode the same protein. As is typical for genes encoding ribosomal proteins, there are multiple processed pseudogenes of this gene dispersed through the genome.	4078.48	3249.1	5214.46	0.33
200817_x_at	RPS10	6204	ribosomal protein S10	RNA binding; cytosolic small ribosomal subunit (sensu Eukarya); protein biosynthesis; structural constituent of ribosome	[SUMMARY:] Ribosomes, the organelles that catalyze protein synthesis, consist of a small 40S subunit and a large 60S subunit. Together these subunits are composed of 4 RNA species and approximately 80 structurally distinct proteins. This gene encodes a ribosomal protein that is a component of the 40S subunit. The protein belongs to the S10E family of ribosomal proteins. It is located in the cytoplasm. Variable expression of this gene in colorectal cancers compared to adjacent normal tissues has been observed, although no correlation between the level of expression and the severity of the disease has been found. As is typical for genes encoding ribosomal proteins, there are multiple processed pseudogenes of this gene dispersed through the genome.	6059.68	4856.83	7765.85	0.32
213969_x_at	RPL29	6159	ribosomal protein L29	GTPase activity; small GTPase mediated signal transduction	[SUMMARY:] Ribosomes, the organelles that catalyze protein synthesis, consist of a small 40S subunit and a large 60S subunit. Together these subunits are composed of 4 RNA species and approximately 80 structurally distinct proteins. This gene encodes a cytoplasmic ribosomal protein that is a component of the 60S subunit. The protein belongs to the L29E family of ribosomal proteins. The protein is also a peripheral membrane protein expressed on the cell surface that directly binds heparin. Although this gene was previously reported to map to 3q29-qter, it is believed that it is located at 3p21.3-p21.2. As is typical for genes encoding ribosomal proteins, there are multiple processed pseudogenes of this gene dispersed through the genome.	3938.53	3160.38	5049.75	0.32
213614_x_at	EEF1A1	1915	eukaryotic translation elongation factor 1 alpha 1	GTP binding; cytoplasm; eukaryotic translation elongation factor 1 complex; oncogenesis; regulation of cell shape; regulation of translation; translational elongation	[SUMMARY:] This gene encodes an isoform of the alpha subunit of the elongation factor-1 complex, which is responsible for the enzymatic delivery of aminoacyl tRNAs to the ribosome. This isoform (alpha 1) is expressed in brain, placenta, lung, liver, kidney, and pancreas, and the other isoform (alpha 2) is expressed in brain, heart and skeletal muscle. This isoform is identified as an autoantigen in 66% of patients with Felty's syndrome. This gene has been found to have multiple copies on many chromosomes, some of which, if not all, represent different pseudogenes.	2540.68	2055.88	3268.28	0.31

218190_s_at	HSPC051	29796	ubiquinol-cytochrome c reductase complex (7.2 kD)	electron transport; inner membrane; mitochondrial electron transport chain; mitochondrial electron transport, ubiquinol to cytochrome c; mitochondrial membrane; oxidoreductase activity; ubiquinol-cytochrome-c reductase activity; ubiquinol-		2670.12	2150.68	3428.55	0.31
200032_s_at	RPL9	6133	ribosomal protein L9	RNA binding; intracellular; protein biosynthesis; ribosome; structural constituent of ribosome	[SUMMARY:] Ribosomes, the organelles that catalyze protein synthesis, consist of a small 40S subunit and a large 60S subunit. Together these subunits are composed of 4 RNA species and approximately 80 structurally distinct proteins. This gene encodes a ribosomal protein that is a component of the 60S subunit. The protein belongs to the L6P family of ribosomal proteins. It is located in the cytoplasm. As is typical for genes encoding ribosomal proteins, there are multiple processed pseudogenes of this gene dispersed through the genome.	4883.62	3953.17	6283.1	0.3
203107_x_at	RPS2	6187	ribosomal protein S2	RNA binding; cytosolic small ribosomal subunit (sensu Eukarya); intracellular; protein biosynthesis; small ribosomal subunit; structural constituent of ribosome	[SUMMARY:] Ribosomes, the organelles that catalyze protein synthesis, consist of a small 40S subunit and a large 60S subunit. Together these subunits are composed of 4 RNA species and approximately 80 structurally distinct proteins. This gene encodes a ribosomal protein that is a component of the 40S subunit. The protein belongs to the S5P family of ribosomal proteins. It is located in the cytoplasm. This gene shares sequence similarity with mouse LLRep3. It is co-transcribed with the small nucleolar RNA gene U64, which is located in its third intron. As is typical for genes encoding ribosomal proteins, there are multiple processed pseudogenes of this gene dispersed through the genome.	4924.27	4008.23	6349.36	0.3
213347_x_at	RPS4X	6191	ribosomal protein S4, X-linked		[SUMMARY:] Cytoplasmic ribosomes, organelles that catalyze protein synthesis, consist of a small 40S subunit and a large 60S subunit. Together these subunits are composed of 4 RNA species and approximately 80 structurally distinct proteins. This gene encodes ribosomal protein S4, a component of the 40S subunit. Ribosomal protein S4 is the only ribosomal protein known to be encoded by more than one gene, namely this gene and ribosomal protein S4, Y-linked (RPS4Y). The 2 isoforms encoded by these genes are not identical, but are functionally equivalent. Ribosomal protein S4 belongs to the S4E family of ribosomal proteins. This gene is not subject to X-inactivation. It has been suggested that haploinsufficiency of the ribosomal protein S4 genes plays a role in Turner syndrome; however, this hypothesis is controversial. As is typical for genes encoding ribosomal proteins, there are multiple processed pseudogenes of this gene dispersed through the genome.	3002.8	2446.45	3873.23	0.3
219772_s_at	SMPX	23676	small muscle protein,	striated muscle contraction		2475.27	2010.78	3189.08	0.3
210646_x_at	RPL13A	23521	ribosomal protein L13a	intracellular; large ribosomal subunit; protein biosynthesis; structural constituent of ribosome	[SUMMARY:] Ribosomes, the organelles that catalyze protein synthesis, consist of a small 40S subunit and a large 60S subunit. Together these subunits are composed of 4 RNA species and approximately 80 structurally distinct proteins. This gene encodes a ribosomal protein that is a component of the 60S subunit. The protein belongs to the L13P family of ribosomal proteins. It is located in the cytoplasm. Transcript variants utilizing alternative polyA signals have been observed. This gene is co-transcribed with the small nucleolar RNA genes U32, U33, U34, and U35, which are located in its second, fourth, fifth, and sixth introns, respectively. As is typical for genes encoding ribosomal proteins, there are multiple processed pseudogenes of this gene dispersed through the genome.	6178.38	5103.58	8013.66	0.28
210976_s_at	PFKM	5213	phosphofructokinase,			2654.48	2181.52	3435.88	0.28
206121_at	AMPD1	270	adenosine monophosphate deaminase 1 (isoform M)		[SUMMARY:] Adenosine monophosphate deaminase 1 catalyzes the deamination of AMP to IMP in skeletal muscle and plays an important role in the purine nucleotide cycle. Two other genes have been identified, AMPD2 and AMPD3, for the liver- and erythocyte-specific isoforms, respectively. Deficiency of the muscle-specific enzyme is apparently a common cause of exercise-induced myopathy and probably the most common cause of metabolic myopathy in the human.	2429.88	2012.25	3154.91	0.27
206393_at	TNNI2	7136	troponin I, skeletal,			4139.67	3428.82	5375.29	0.27
212790_x_at	RPL13A	23521	ribosomal protein L13a	intracellular; large ribosomal subunit; protein biosynthesis; structural constituent of ribosome	[SUMMARY:] Ribosomes, the organelles that catalyze protein synthesis, consist of a small 40S subunit and a large 60S subunit. Together these subunits are composed of 4 RNA species and approximately 80 structurally distinct proteins. This gene encodes a ribosomal protein that is a component of the 60S subunit. The protein belongs to the L13P family of ribosomal proteins. It is located in the cytoplasm. Transcript variants utilizing alternative polyA signals have been observed. This gene is co-transcribed with the small nucleolar RNA genes U32, U33, U34, and U35, which are located in its second, fourth, fifth, and sixth introns, respectively. As is typical for genes encoding ribosomal proteins, there are multiple processed pseudogenes of this gene dispersed through the genome.	4920.4	4079.15	6391.38	0.27
201322_at	ATP5B	506	ATP synthase, H+ transporting, mitochondrial F1 complex, beta polypeptide	ATP binding; ATP synthesis coupled proton transport; ATP-binding and phosphorylation-dependent chloride channel activity; energy pathways; hydrogen-exporting ATPase activity, phosphorylative mechanism; hydrogen-transporting ATP synthase activity, rotational mechanism; hydrolase activity; mitochondrion; nucleotide binding; proton transport; proton-transporting ATP synthase complex (sensu Eukarya); proton-transporting ATP synthase,		2256.32	1909.62	2955.96	0.24

212191_x_at	RPL13	6137	ribosomal protein L13		[SUMMARY:] Ribosomes, the organelles that catalyze protein synthesis, consist of a small 40S subunit and a large 60S subunit. Together these subunits are composed of 4 RNA species and approximately 80 structurally distinct proteins. This gene encodes a ribosomal protein that is a component of the 60S subunit. The protein belongs to the L13E family of ribosomal proteins. It is located in the cytoplasm. This gene is expressed at significantly higher levels in benign breast lesions than in breast carcinomas. Transcript variants derived from alternative splicing and/or alternative polyadenylation exist; these variants encode the same protein. As is typical for genes encoding ribosomal proteins, there are multiple processed pseudogenes of this gene dispersed through the genome.	2501.8	2135.75	3289.44	0.23
212433_x_at	RPS2	6187	ribosomal protein S2	RNA binding; cytosolic small ribosomal subunit (sensu Eukarya); intracellular; protein biosynthesis; small ribosomal subunit; structural constituent of ribosome	[SUMMARY:] Ribosomes, the organelles that catalyze protein synthesis, consist of a small 40S subunit and a large 60S subunit. Together these subunits are composed of 4 RNA species and approximately 80 structurally distinct proteins. This gene encodes a ribosomal protein that is a component of the 40S subunit. The protein belongs to the S5P family of ribosomal proteins. It is located in the cytoplasm. This gene shares sequence similarity with mouse LLRep3. It is co-transcribed with the small nucleolar RNA gene U64, which is located in its third intron. As is typical for genes encoding ribosomal proteins, there are multiple processed pseudogenes of this gene dispersed through the genome.	2781.58	2365.9	3651.66	0.23
205553_s_at	CSRP3	8048	cysteine and glycine-rich protein 3 (cardiac LIM protein)	myogenesis; nucleus	[SUMMARY:] CSRP3 is a member of the CSRP family of genes encoding a group of LIM domain proteins, which may be involved in regulatory processes important for development and cellular differentiation. The LIM/double zinc-finger motif found in CRP3 is found in a group of proteins with critical functions in gene regulation, cell growth, and somatic differentiation Other genes in the family include CSRP1 and CSRP2.	2645.1	2278.12	3490.9	0.22
213477_x_at	EEF1A1	1915	eukaryotic translation elongation factor 1 alpha 1	GTP binding; cytoplasm; eukaryotic translation elongation factor 1 complex; oncogenesis; regulation of cell shape; regulation of translation; translational elongation	[SUMMARY:] This gene encodes an isoform of the alpha subunit of the elongation factor-1 complex, which is responsible for the enzymatic delivery of aminoacyl tRNAs to the ribosome. This isoform (alpha 1) is expressed in brain, placenta, lung, liver, kidney, and pancreas, and the other isoform (alpha 2) is expressed in brain, heart and skeletal muscle. This isoform is identified as an autoantigen in 66% of patients with Felty's syndrome. This gene has been found to have multiple copies on many chromosomes, some of which, if not all, represent different pseudogenes.	2711.2	2350.35	3588.14	0.21
200780_x_at	GNAS	2778	GNAS complex locus	G-protein coupled receptor protein signaling pathway; G-protein signaling, adenylate cyclase activating pathway; GTP binding; Golgi to secretory vesicle transport; Golgi trans cisterna; adenylate cyclase activation; cell growth and/or maintenance; extracellular; heterotrimeric G-protein GTPase activity; heterotrimeric G-protein complex; molecular_function unknown; perception of	[SUMMARY:] This gene has a highly complex imprinted expression pattern. It encodes maternally, paternally, and biallelically expressed proteins which are derived from alternatively spliced transcripts with alternate 5' exons. Each of the upstream exons is within a differentially methylated region, commonly found in imprinted genes. However, the close proximity (14 kb) of two oppositely expressed promoter regions is unusual. In addition, one of the alternate 5' exons introduces a frameshift relative to the other transcripts, resulting in one isoform which is structurally unrelated to the others. An antisense transcript exists, and may regulate imprinting in this region. Mutations in this gene result in pseudohypoparathyroidism type 1a (PHP1a), which has an atypical autosomal dominant inheritance pattern requiring maternal transmission for full penetrance. There are RefSeqs representing four transcript variants of this gene. Other transcript variants including four additional exons have been described; however, their full length sequences have not been determined.	2583.62	2249.2	3425.5	0.2
203012_x_at	RPL23A	6147	ribosomal protein L23a	cytosolic large ribosomal subunit (sensu Eukarya); intracellular; protein biosynthesis; rRNA binding; ribosome; structural constituent of ribosome	[SUMMARY:] Ribosomes, the organelles that catalyze protein synthesis, consist of a small 40S subunit and a large 60S subunit. Together these subunits are composed of 4 RNA species and approximately 80 structurally distinct proteins. This gene encodes a ribosomal protein that is a component of the 60S subunit. The protein belongs to the L23P family of ribosomal proteins. It is located in the cytoplasm. The protein may be one of the target molecules involved in mediating growth inhibition by interferon. In yeast, the corresponding protein binds to a specific site on the 26S rRNA. This gene is co-transcribed with the U42A, U42B, U101A, and U101B small nucleolar RNA genes, which are located in its third, first, second, and fourth introns, respectively. As is typical for genes encoding ribosomal proteins, there are multiple processed pseudogenes of this gene dispersed through the genome.	5323	4637.85	7060.03	0.2
209134_s_at	RPS6	6194	ribosomal protein S6	RNA binding; cytosolic small ribosomal subunit (sensu Eukarya); intracellular; protein biosynthesis; ribosome; structural constituent of ribosome	[SUMMARY:] Ribosomes, the organelles that catalyze protein synthesis, consist of a small 40S subunit and a large 60S subunit. Together these subunits are composed of 4 RNA species and approximately 80 structurally distinct proteins. This gene encodes a cytoplasmic ribosomal protein that is a component of the 40S subunit. The protein belongs to the S6E family of ribosomal proteins. It is the major substrate of protein kinases in the ribosome, with subsets of five C-terminal serine residues phosphorylated by different protein kinases. Phosphorylation is induced by a wide range of stimuli, including growth factors, tumor-promoting agents, and mitogens. Dephosphorylation occurs at growth arrest. The protein may contribute to the control of cell growth and proliferation through the selective translation of particular classes of mRNA. As is typical for genes encoding ribosomal proteins, there are multiple processed pseudogenes of this gene dispersed through the genome.	3244.82	2831.35	4306.44	0.2
209621_s_at	PDLIM3	27295	PDZ and LIM domain	protein binding		3455.95	3025.08	4592.89	0.19
204483_at	ENO3	2027	enolase 3, (beta, muscle)		[SUMMARY:] This gene encodes one of the three enolase isoenzymes found in mammals. This isoenzyme, a homodimer, is found in skeletal muscle cells in the adult. A switch from alpha enolase to beta enolase occurs in muscle tissue during development in rodents. Mutations in this gene can be associated with metabolic myopathies that may result from decreased stability of the enzyme. Two transcripts have been identified for this gene that differ only in their 5' UTR.	4505.52	3986.85	6016.21	0.18
205951_at	MYH1	4619	myosin, heavy polypeptide 1, skeletal muscle, adult	ATP binding; actin binding; calmodulin binding; microfilament motor activity; muscle development; muscle myosin; myosin; striated muscle contraction; striated muscle thick filament	[SUMMARY:] Myosin is a major contractile protein which converts chemical energy into mechanical energy through the hydrolysis of ATP. Myosin is a hexameric protein composed of a pair of myosin heavy chains (MYH) and two pairs of nonidentical light chains. Myosin heavy chains are encoded by a multigene family. In mammals at least 10 different myosin heavy chain (MYH) isoforms have been described from striated, smooth, and nonmuscle cells. These isoforms show expression that is spatially and temporally regulated during development.	2611.08	2317.07	3490.92	0.17

200823_x_at	RPL29	6159	ribosomal protein L29	GTPase activity; small GTPase mediated signal transduction	[SUMMARY:] Ribosomes, the organelles that catalyze protein synthesis, consist of a small 40S subunit and a large 60S subunit. Together these subunits are composed of 4 RNA species and approximately 80 structurally distinct proteins. This gene encodes a cytoplasmic ribosomal protein that is a component of the 60S subunit. The protein belongs to the L29E family of ribosomal proteins. The protein is also a peripheral membrane protein expressed on the cell surface that directly binds heparin. Although this gene was previously reported to map to 3q29-qter, it is believed that it is located at 3p21.3-p21.2. As is typical for genes encoding ribosomal proteins, there are multiple processed pseudogenes of this gene dispersed through the genome.	2686.75	2411.88	3610.51	0.16
200716_x_at	RPL13A	23521	ribosomal protein L13a	intracellular; large ribosomal subunit; protein biosynthesis; structural constituent of ribosome	[SUMMARY:] Ribosomes, the organelles that catalyze protein synthesis, consist of a small 40S subunit and a large 60S subunit. Together these subunits are composed of 4 RNA species and approximately 80 structurally distinct proteins. This gene encodes a ribosomal protein that is a component of the 60S subunit. The protein belongs to the L13P family of ribosomal proteins. It is located in the cytoplasm. Transcript variants utilizing alternative polyA signals have been observed. This gene is co-transcribed with the small nucleolar RNA genes U32, U33, U34, and U35, which are located in its second, fourth, fifth, and sixth introns, respectively. As is typical for genes encoding ribosomal proteins, there are multiple processed pseudogenes of this gene dispersed through the genome.	4903.1	4426.62	6605.71	0.15
208909_at	UQCRFS1	7386	ubiquinol-cytochrome c reductase, Rieske iron-sulfur polypeptide 1	electron transport; inner membrane; integral to membrane; mitochondrion; oxidoreductase activity; ubiquinol-cytochrome-c reductase activity; ubiquinol-cytochrome-c reductase		2468.6	2229.75	3326.53	0.15
217740_x_at	RPL7A	6130	ribosomal protein L7a		[SUMMARY:] Cytoplasmic ribosomes, organelles that catalyze protein synthesis, consist of a small 40S subunit and a large 60S subunit. Together these subunits are composed of 4 RNA species and approximately 80 structurally distinct proteins. This gene encodes a ribosomal protein that is a component of the 60S subunit. The protein belongs to the L7AE family of ribosomal proteins. It can interact with a subclass of nuclear hormone receptors, including thyroid hormone receptor, and inhibit their ability to transactivate by preventing their binding to their DNA response elements. This gene is included in the surfeit gene cluster, a group of very tightly linked genes that do not share sequence similarity. It is co-transcribed with the U24, U36a, U36b, and U36c small nucleolar RNA genes, which are located in its second, fifth, fourth, and sixth introns, respectively. This gene rearranges with the trk proto-oncogene to form the chimeric oncogene trk-2h, which encodes an oncoprotein consisting of the N terminus of ribosomal protein L7a fused to the receptor tyrosine kinase domain of trk. As is typical for genes encoding ribo	3147.6	2828.07	4231.48	0.15
211345_x_at						4279.27	3873.02	5771.7	0.14
212654_at	TPM2	7169	tropomyosin 2 (beta)	actin binding; cytoskeleton; muscle development; muscle thin filament tropomyosin; structural constituent of		4238.27	3876.7	5743.85	0.13
214351_x_at	RPL13	6137	ribosomal protein L13		[SUMMARY:] Ribosomes, the organelles that catalyze protein synthesis, consist of a small 40S subunit and a large 60S subunit. Together these subunits are composed of 4 RNA species and approximately 80 structurally distinct proteins. This gene encodes a ribosomal protein that is a component of the 60S subunit. The protein belongs to the L13E family of ribosomal proteins. It is located in the cytoplasm. This gene is expressed at significantly higher levels in benign breast lesions than in breast carcinomas. Transcript variants derived from alternative splicing and/or alternative polyadenylation exist; these variants encode the same protein. As is typical for genes encoding ribosomal proteins, there are multiple processed pseudogenes of this gene dispersed through the genome.	2145.45	1956.42	2903.54	0.13
206353_at	COX6A2	1339	cytochrome c oxidase subunit VIa polypeptide 2	cytochrome-c oxidase activity; electron transport; energy pathways; inner membrane; mitochondrial membrane; oxidoreductase activity	[SUMMARY:] Cytochrome c oxidase (COX), the terminal enzyme of the mitochondrial respiratory chain, catalyzes the electron transfer from reduced cytochrome c to oxygen. It is a heteromeric complex consisting of 3 catalytic subunits encoded by mitochondrial genes and multiple structural subunits encoded by nuclear genes. The mitochondrially-encoded subunits function in electron transfer, and the nuclear-encoded subunits may be involved in the regulation and assembly of the complex. This nuclear gene encodes polypeptide 2 (heart/muscle isoform) of subunit VIa, and polypeptide 2 is present only in striated muscles. Polypeptide 1 (liver isoform) of subunit VIa is encoded by a different gene, and is found in all non-muscle tissues. These two polypeptides share 66% amino acid sequence identity.	2433.8	2258.27	3320.12	0.11
210046_s_at	IDH2	3418	isocitrate dehydrogenase 2 (NADP+), mitochondrial	glyoxylate cycle; isocitrate dehydrogenase (NADP+) activity; main pathways of carbohydrate metabolism; metabolism; mitochondrion; oxidoreductase activity; tricarboxylic acid cycle	[SUMMARY:] Isocitrate dehydrogenases catalyze the oxidative decarboxylation of isocitrate to 2-oxoglutarate. These enzymes belong to two distinct subclasses, one of which utilizes NAD(+) as the electron acceptor and the other NADP(+). Five isocitrate dehydrogenases have been reported: three NAD(+)-dependent isocitrate dehydrogenases, which localize to the mitochondrial matrix, and two NADP(+)-dependent isocitrate dehydrogenases, one of which is mitochondrial and the other predominantly cytosolic. Each NADP(+)-dependent isozyme is a homodimer. The protein encoded by this gene is the NADP(+)-dependent isocitrate dehydrogenase found in the mitochondria. It plays a role in intermediary metabolism and energy production. This protein may tightly associate or interact with the pyruvate dehydrogenase complex.	2093.02	1940.5	2854.17	0.11
213801_x_at	LAMR1	3921	laminin receptor 1 (ribosomal protein SA, 67kDa)	cell adhesion; cell surface receptor linked signal transduction; cytosolic small ribosomal subunit (sensu Eukarya); integrin complex; intracellular; laminin receptor activity; protein biosynthesis; regulation of translation; structural constituent of ribosome	[SUMMARY:] Laminins, a family of extracellular matrix glycoproteins, are the major noncollagenous constituent of basement membranes. They have been implicated in a wide variety of biological processes including cell adhesion, differentiation, migration, signaling, neurite outgrowth and metastasis. Many of the effects of laminin are mediated through interactions with cell surface receptors. These receptors include members of the integrin family, as well as non-integrin laminin-binding proteins. This gene encodes a high-affinity, non-integrin family, laminin receptor 1. This receptor has been variously called 67 kD laminin receptor, 37 kD laminin receptor precursor (37LRP) and p40 ribosome-associated protein. The amino acid sequence of laminin receptor 1 is highly conserved through evolution, suggesting a key biological function. It has been observed that the level of the laminin receptor transcript is higher in colon carcinoma tissue and lung cancer cell line than their normal counterparts. Also, there is a correlation between the upregulation of this polypeptide in cancer cells and their invasive and metastatic phenotype. Multip	2353.38	2317.92	3303.2	0.02

200029_at	RPL19	6143	ribosomal protein L19	RNA binding; cytosolic large ribosomal subunit (sensu Eukarya); intracellular; protein biosynthesis; ribosome; structural constituent of ribosome	[SUMMARY:] Ribosomes, the organelles that catalyze protein synthesis, consist of a small 40S subunit and a large 60S subunit. Together these subunits are composed of 4 RNA species and approximately 80 structurally distinct proteins. This gene encodes a ribosomal protein that is a component of the 60S subunit. The protein belongs to the L19E family of ribosomal proteins. It is located in the cytoplasm. As is typical for genes encoding ribosomal proteins, there are multiple processed pseudogenes of this gene dispersed through the genome.	2426.85	2414.12	3423.1	0.01
200088_x_at	RPL12	6136	ribosomal protein L12	RNA binding; cytosolic large ribosomal subunit (sensu Eukarya); intracellular; protein biosynthesis; ribosome; structural constituent of ribosome	[SUMMARY:] Ribosomes, the organelles that catalyze protein synthesis, consist of a small 40S subunit and a large 60S subunit. Together these subunits are composed of 4 RNA species and approximately 80 structurally distinct proteins. This gene encodes a ribosomal protein that is a component of the 60S subunit. The protein belongs to the L11P family of ribosomal proteins. It is located in the cytoplasm. The protein binds directly to the 26S rRNA. This gene is co-transcribed with the U65 snoRNA, which is located in its fourth intron. As is typical for genes encoding ribosomal proteins, there are multiple processed pseudogenes of this gene dispersed through the genome.	2505	2525.85	3557.38	-0.01
200936_at	RPL8	6132	ribosomal protein L8	cytosolic large ribosomal subunit (sensu Eukarya); intracellular; protein biosynthesis; rRNA binding; ribosome; structural constituent of ribosome	[SUMMARY:] Ribosomes, the organelles that catalyze protein synthesis, consist of a small 40S subunit and a large 60S subunit. Together these subunits are composed of 4 RNA species and approximately 80 structurally distinct proteins. This gene encodes a ribosomal protein that is a component of the 60S subunit. The protein belongs to the L2P family of ribosomal proteins. It is located in the cytoplasm. In rat, the protein associates with the 5.8S rRNA, very likely participates in the binding of aminoacyl-tRNA, and is a constituent of the elongation factor 2-binding site at the ribosomal subunit interface. Alternatively spliced transcript variants encoding the same protein exist. As is typical for genes encoding ribosomal proteins, there are multiple processed pseudogenes of this gene dispersed through the genome.	2469.52	2493.35	3509.32	-0.01
211942_x_at	RPL13A	23521	ribosomal protein L13a	intracellular; large ribosomal subunit; protein biosynthesis; structural constituent of ribosome	[SUMMARY:] Ribosomes, the organelles that catalyze protein synthesis, consist of a small 40S subunit and a large 60S subunit. Together these subunits are composed of 4 RNA species and approximately 80 structurally distinct proteins. This gene encodes a ribosomal protein that is a component of the 60S subunit. The protein belongs to the L13P family of ribosomal proteins. It is located in the cytoplasm. Transcript variants utilizing alternative polyA signals have been observed. This gene is co-transcribed with the small nucleolar RNA genes U32, U33, U34, and U35, which are located in its second, fourth, fifth, and sixth introns, respectively. As is typical for genes encoding ribosomal proteins, there are multiple processed pseudogenes of this gene dispersed through the genome.	2821.82	2837.75	4001.94	-0.01
200689_x_at	EEF1G	1937	eukaryotic translation elongation factor 1 gamma	eukaryotic translation elongation factor 1 complex; intracellular; nucleic acid binding; protein biosynthesis; translation elongation factor activity; translational	[SUMMARY:] This gene encodes a subunit of the elongation factor-1 complex, which is responsible for the enzymatic delivery of aminoacyl tRNAs to the ribosome. This subunit contains an N-terminal glutathione transferase domain, which may be involved in regulating the assembly of multisubunit complexes containing this elongation factor and aminoacyl-tRNA synthetases.	3773.82	3834.68	5380.19	-0.02
205766_at	TCAP	8557	titin-cap (telethonin)	cytoplasm; protein complex assembly; sarcomere alignment; structural constituent of muscle	[SUMMARY:] Sarcomere assembly is regulated by the muscle protein titin. Titin is a giant elastic protein with kinase activity that extends half the length of a sarcomere. It serves as a scaffold to which myofibrils and other muscle related proteins are attached. This gene encodes a protein found in striated and cardiac muscle that binds to the titin Z1-Z2 domains and is a substrate of titin kinase, interactions thought to be critical to sarcomere assembly. Mutations in this gene are associated with limb-girdle muscular dystrophy type 2G.	2224.5	2266.45	3175.72	-0.03
200012_x_at	RPL21	6144	ribosomal protein L21		[SUMMARY:] Ribosomes, the organelles that catalyze protein synthesis, consist of a small 40S subunit and a large 60S subunit. Together these subunits are composed of 4 RNA species and approximately 80 structurally distinct proteins. This gene encodes a ribosomal protein that is a component of the 60S subunit. The protein belongs to the L21E family of ribosomal proteins. It is located in the cytoplasm. As is typical for genes encoding ribosomal proteins, there are multiple processed pseudogenes of this gene dispersed through the genome.	2670.15	2739.95	3825.84	-0.04
200061_s_at	RPS24	6229	ribosomal protein S24	RNA binding; cytosolic small ribosomal subunit (sensu Eukarya); intracellular; protein biosynthesis; ribosome; structural constituent of ribosome	[SUMMARY:] Ribosomes, the organelles that catalyze protein synthesis, consist of a small 40S subunit and a large 60S subunit. Together these subunits are composed of 4 RNA species and approximately 80 structurally distinct proteins. This gene encodes a ribosomal protein that is a component of the 40S subunit. The protein belongs to the S24E family of ribosomal proteins. It is located in the cytoplasm. Alternative splice variants that encode different protein isoforms exist. As is typical for genes encoding ribosomal proteins, there are multiple processed pseudogenes of this gene dispersed through the genome.	3312.85	3399.08	4746.44	-0.04
200981_x_at	GNAS	2778	GNAS complex locus	G-protein coupled receptor protein signaling pathway; G-protein signaling, adenylate cyclase activating pathway; GTP binding; Golgi to secretory vesicle transport; Golgi trans cisterna; adenylate cyclase activation; cell growth and/or maintenance; extracellular; heterotrimeric G-protein GTPase activity; heterotrimeric G-protein complex; molecular_function unknown; perception of	[SUMMARY:] This gene has a highly complex imprinted expression pattern. It encodes maternally, paternally, and biallelically expressed proteins which are derived from alternatively spliced transcripts with alternate 5' exons. Each of the upstream exons is within a differentially methylated region, commonly found in imprinted genes. However, the close proximity (14 kb) of two oppositely expressed promoter regions is unusual. In addition, one of the alternate 5' exons introduces a frameshift relative to the other transcripts, resulting in one isoform which is structurally unrelated to the others. An antisense transcript exists, and may regulate imprinting in this region. Mutations in this gene result in pseudohypoparathyroidism type 1a (PHP1a), which has an atypical autosomal dominant inheritance pattern requiring maternal transmission for full penetrance. There are RefSeqs representing four transcript variants of this gene. Other transcript variants including four additional exons have been described; however, their full length sequences have not been determined.	2194.23	2255.05	3146.41	-0.04
201161_s_at	CSDA	8531	cold shock domain protein A	DNA binding; RNA polymerase II transcription factor activity; cytoplasm; double-stranded DNA binding; negative regulation of transcription from Pol II promoter; perinuclear space; regulation of transcription, DNA-dependent; response to cold; transcription		2901.5	3024.25	4191.04	-0.06

204737_s_at	MYH7	4625	myosin, heavy polypeptide 7, cardiac muscle, beta	ATP binding; actin binding; calmodulin binding; motor activity; muscle development; muscle myosin; myosin; striated muscle contraction; striated muscle thick filament;	[SUMMARY:] MYH7 encodes the cardiac muscle beta (or slow) isoform of myosin. Changes in the relative abundance of MYH7 and MYH6 (the alpha, or fast, isoform of cardiac myosin heavy chain) correlate with the contractile velocity of cardiac muscle. Mutations in MYH7 are associated with familial hypertrophic cardiomyopathy.	3654.38	3837.4	5299.07	-0.07
201568_at	QP-C	27089	low molecular mass ubiquinone-binding protein (9.5kD)	electron transport; inner membrane; mitochondrion; oxidoreductase activity; ubiquinol-cytochrome-c reductase activity; ubiquinol-cytochrome-c reductase	[SUMMARY:] This gene encodes a ubiquinone-binding protein of low molecular mass. This function of this protein has not yet been determined; however, it is a homologue of bovine low molecular mass ubiquinone-binding protein gene.	2131.88	2249.9	3099.51	-0.08
214143_x_at	RPL24	6152	ribosomal protein L24		[SUMMARY:] Ribosomes, the organelles that catalyze protein synthesis, consist of a small 40S subunit and a large 60S subunit. Together these subunits are composed of 4 RNA species and approximately 80 structurally distinct proteins. This gene encodes a ribosomal protein that is a component of the 60S subunit. The protein belongs to the L24E family of ribosomal proteins. It is located in the cytoplasm. This gene has been referred to as ribosomal protein L30 because the encoded protein shares amino acid identity with the L30 ribosomal proteins from S. cerevisiae; however, its official name is ribosomal protein L24. As is typical for genes encoding ribosomal proteins, there are multiple processed pseudogenes of this gene dispersed through the genome.	2462.82	2642.67	3612.37	-0.1
219728_at	TTID	9499	titin immunoglobulin domain protein (myotilin)	actin cytoskeleton; muscle contraction; protein binding; structural constituent of		2494.8	2671.98	3655.61	-0.1
211487_x_at						1943.47	2106.4	2866.01	-0.12
200809_x_at	RPL12	6136	ribosomal protein L12	RNA binding; cytosolic large ribosomal subunit (sensu Eukarya); intracellular; protein biosynthesis; ribosome; structural constituent of ribosome	[SUMMARY:] Ribosomes, the organelles that catalyze protein synthesis, consist of a small 40S subunit and a large 60S subunit. Together these subunits are composed of 4 RNA species and approximately 80 structurally distinct proteins. This gene encodes a ribosomal protein that is a component of the 60S subunit. The protein belongs to the L11P family of ribosomal proteins. It is located in the cytoplasm. The protein binds directly to the 26S rRNA. This gene is co-transcribed with the U65 snoRNA, which is located in its fourth intron. As is typical for genes encoding ribosomal proteins, there are multiple processed pseudogenes of this gene dispersed through the genome.	2327.1	2539.1	3444.19	-0.13
200092_s_at	RPL37	6167	ribosomal protein L37		[SUMMARY:] Ribosomes, the organelles that catalyze protein synthesis, consist of a small 40S subunit and a large 60S subunit. Together these subunits are composed of 4 RNA species and approximately 80 structurally distinct proteins. This gene encodes a ribosomal protein that is a component of the 60S subunit. The protein belongs to the L37E family of ribosomal proteins. It is located in the cytoplasm. The protein contains a C2C2-type zinc finger-like motif. As is typical for genes encoding ribosomal proteins, there are multiple processed pseudogenes of this gene dispersed through the genome.	2523.23	2783.32	3756.8	-0.14
201254_x_at	RPS6	6194	ribosomal protein S6	RNA binding; cytosolic small ribosomal subunit (sensu Eukarya); intracellular; protein biosynthesis; ribosome; structural constituent of ribosome	[SUMMARY:] Ribosomes, the organelles that catalyze protein synthesis, consist of a small 40S subunit and a large 60S subunit. Together these subunits are composed of 4 RNA species and approximately 80 structurally distinct proteins. This gene encodes a cytoplasmic ribosomal protein that is a component of the 40S subunit. The protein belongs to the S6E family of ribosomal proteins. It is the major substrate of protein kinases in the ribosome, with subsets of five C-terminal serine residues phosphorylated by different protein kinases. Phosphorylation is induced by a wide range of stimuli, including growth factors, tumor-promoting agents, and mitogens. Dephosphorylation occurs at growth arrest. The protein may contribute to the control of cell growth and proliferation through the selective translation of particular classes of mRNA. As is typical for genes encoding ribosomal proteins, there are multiple processed pseudogenes of this gene dispersed through the genome.	2852.08	3161.35	4257.75	-0.15
200013_at	RPL24	6152	ribosomal protein L24		[SUMMARY:] Ribosomes, the organelles that catalyze protein synthesis, consist of a small 40S subunit and a large 60S subunit. Together these subunits are composed of 4 RNA species and approximately 80 structurally distinct proteins. This gene encodes a ribosomal protein that is a component of the 60S subunit. The protein belongs to the L24E family of ribosomal proteins. It is located in the cytoplasm. This gene has been referred to as ribosomal protein L30 because the encoded protein shares amino acid identity with the L30 ribosomal proteins from S. cerevisiae; however, its official name is ribosomal protein L24. As is typical for genes encoding ribosomal proteins, there are multiple processed pseudogenes of this gene dispersed through the genome.	1903.15	2188.12	2899.98	-0.2
208856_x_at	RPLP0	6175	ribosomal protein, large, P0	RNA binding; cytosolic large ribosomal subunit (sensu Eukarya); intracellular; protein biosynthesis; ribosome; structural constituent of ribosome; translational elongation	[SUMMARY:] Ribosomes, the organelles that catalyze protein synthesis, consist of a small 40S subunit and a large 60S subunit. Together these subunits are composed of 4 RNA species and approximately 80 structurally distinct proteins. This gene encodes a ribosomal protein that is a component of the 60S subunit. The protein, which is the functional equivalent of the E. coli L10 ribosomal protein, belongs to the L10P family of ribosomal proteins. It is a neutral phosphoprotein with a C-terminal end that is nearly identical to the C-terminal ends of the acidic ribosomal phosphoproteins P1 and P2. The P0 protein can interact with P1 and P2 to form a pentameric complex consisting of P1 and P2 dimers, and a P0 monomer. The protein is located in the cytoplasm. Transcript variants derived from alternative splicing exist; they encode the same protein. As is typical for genes encoding ribosomal proteins, there are multiple processed pseudogenes of this gene dispersed through the genome.	3394.15	3900.4	5170.43	-0.2
201033_x_at	RPLP0	6175	ribosomal protein, large, P0	RNA binding; cytosolic large ribosomal subunit (sensu Eukarya); intracellular; protein biosynthesis; ribosome; structural constituent of ribosome; translational elongation	[SUMMARY:] Ribosomes, the organelles that catalyze protein synthesis, consist of a small 40S subunit and a large 60S subunit. Together these subunits are composed of 4 RNA species and approximately 80 structurally distinct proteins. This gene encodes a ribosomal protein that is a component of the 60S subunit. The protein, which is the functional equivalent of the E. coli L10 ribosomal protein, belongs to the L10P family of ribosomal proteins. It is a neutral phosphoprotein with a C-terminal end that is nearly identical to the C-terminal ends of the acidic ribosomal phosphoproteins P1 and P2. The P0 protein can interact with P1 and P2 to form a pentameric complex consisting of P1 and P2 dimers, and a P0 monomer. The protein is located in the cytoplasm. Transcript variants derived from alternative splicing exist; they encode the same protein. As is typical for genes encoding ribosomal proteins, there are multiple processed pseudogenes of this gene dispersed through the genome.	3469.4	4007.5	5300.64	-0.21

201812_s_at	TOMM7	54543	translocase of outer mitochondrial membrane 7 homolog (yeast)	integral to membrane; intracellular protein transport; mitochondrion; outer membrane; protein		1974.48	2276.1	3013.17	-0.21
201891_s_at	B2M	567	beta-2-microglobulin	MHC class I receptor activity; antigen presentation, endogenous antigen; antigen processing, endogenous antigen via MHC class I;		1858.93	2144.62	2838.14	-0.21
200717_x_at	RPL7	6129	ribosomal protein L7	RNA binding; cytosolic large ribosomal subunit (sensu Eukarya); intracellular; protein biosynthesis; structural constituent of ribosome; transcription regulator activity	[SUMMARY:] Ribosomes, the organelles that catalyze protein synthesis, consist of a small 40S subunit and a large 60S subunit. Together these subunits are composed of 4 RNA species and approximately 80 structurally distinct proteins. This gene encodes a ribosomal protein that is a component of the 60S subunit. The protein belongs to the L30P family of ribosomal proteins. It contains an N-terminal basic region-leucine zipper (BZIP)-like domain and the RNP consensus submotif RNP2. In vitro the BZIP-like domain mediates homodimerization and stable binding to DNA and RNA, with a preference for 28S rRNA and mRNA. The protein can inhibit cell-free translation of mRNAs, suggesting that it plays a regulatory role in the translation apparatus. It is located in the cytoplasm. The protein has been shown to be an autoantigen in patients with systemic autoimmune diseases, such as systemic lupus erythematosus. As is typical for genes encoding ribosomal proteins, there are multiple processed pseudogenes of this gene dispersed through the genome.	2978.08	3533.4	4621.02	-0.25
200949_x_at	RPS20	6224	ribosomal protein S20	RNA binding; cytosolic small ribosomal subunit (sensu Eukarya); intracellular; protein biosynthesis; structural constituent of ribosome	[SUMMARY:] Ribosomes, the organelles that catalyze protein synthesis, consist of a small 40S subunit and a large 60S subunit. Together these subunits are composed of 4 RNA species and approximately 80 structurally distinct proteins. This gene encodes a ribosomal protein that is a component of the 40S subunit. The protein belongs to the S10P family of ribosomal proteins. It is located in the cytoplasm. This gene is co-transcribed with the small nucleolar RNA gene U54, which is located in its second intron. As is typical for genes encoding ribosomal proteins, there are multiple processed pseudogenes of this gene dispersed through the genome.	2261.98	2695.62	3518.94	-0.25
200819_s_at	RPS15	6209	ribosomal protein S15		[SUMMARY:] Ribosomes, the organelles that catalyze protein synthesis, consist of a small 40S subunit and a large 60S subunit. Together these subunits are composed of 4 RNA species and approximately 80 structurally distinct proteins. This gene encodes a ribosomal protein that is a component of the 40S subunit. The protein belongs to the S19P family of ribosomal proteins. It is located in the cytoplasm. This gene has been found to be activated in various tumors, such as insulinomas, esophageal cancers, and colon cancers. As is typical for genes encoding ribosomal proteins, there are multiple processed pseudogenes of this gene dispersed through the genome.	2707.4	3244.18	4225.48	-0.26
200963_x_at	RPL31	6160	ribosomal protein L31	RNA binding; cytosolic large ribosomal subunit (sensu Eukarya); intracellular; protein biosynthesis; ribosome; structural constituent of ribosome	[SUMMARY:] Ribosomes, the organelles that catalyze protein synthesis, consist of a small 40S subunit and a large 60S subunit. Together these subunits are composed of 4 RNA species and approximately 80 structurally distinct proteins. This gene encodes a ribosomal protein that is a component of the 60S subunit. The protein belongs to the L31E family of ribosomal proteins. It is located in the cytoplasm. Higher levels of expression of this gene in familial adenomatous polyps compared to matched normal tissues have been observed. As is typical for genes encoding ribosomal proteins, there are multiple processed pseudogenes of this gene dispersed through the genome.	2743.35	3280.55	4276.44	-0.26
200002_at	RPL35	11224	ribosomal protein L35		[SUMMARY:] Ribosomes, the organelles that catalyze protein synthesis, consist of a small 40S subunit and a large 60S subunit. Together these subunits are composed of 4 RNA species and approximately 80 structurally distinct proteins. This gene encodes a ribosomal protein that is a component of the 60S subunit. The protein belongs to the L29P family of ribosomal proteins. It is located in the cytoplasm. As is typical for genes encoding ribosomal proteins, there are multiple processed pseudogenes of this gene dispersed through the genome.	2124.5	2591.73	3351.2	-0.29
201665_x_at	RPS17	6218	ribosomal protein S17		[SUMMARY:] Ribosomes, the organelles that catalyze protein synthesis, consist of a small 40S subunit and a large 60S subunit. Together these subunits are composed of 4 RNA species and approximately 80 structurally distinct proteins. This gene encodes a ribosomal protein that is a component of the 40S subunit. The protein belongs to the S17E family of ribosomal proteins. It is located in the cytoplasm. As is typical for genes encoding ribosomal proteins, there are multiple processed pseudogenes of this gene dispersed through the genome.	1878.68	2348.6	3007.55	-0.32
208768_x_at	RPL22	6146	ribosomal protein L22		[SUMMARY:] Ribosomes, the organelles that catalyze protein synthesis, consist of a small 40S subunit and a large 60S subunit. Together these subunits are composed of 4 RNA species and approximately 80 structurally distinct proteins. This gene encodes a cytoplasmic ribosomal protein that is a component of the 60S subunit. The protein belongs to the L22E family of ribosomal proteins. Its initiating methionine residue is post-translationally removed. The protein can bind specifically to Epstein-Barr virus-encoded RNAs (EBERs) 1 and 2. The mouse protein has been shown to be capable of binding to heparin. Transcript variants utilizing alternative polyA signals exist. As is typical for genes encoding ribosomal proteins, there are multiple processed pseudogenes of this gene dispersed through the genome. It was previously thought that this gene mapped to 3q26 and that it was fused to the acute myeloid leukemia 1 (AML1) gene located at 21q22 in some therapy-related myelodysplastic syndrome patients with 3;21 translocations; however, these fusions actually involve a ribosomal protein L22 pseudogene located at 3	1845.8	2336.45	2977.58	-0.34
211720_x_at	RPLP0	6175	ribosomal protein, large, P0	RNA binding; cytosolic large ribosomal subunit (sensu Eukarya); intracellular; protein biosynthesis; ribosome; structural constituent of ribosome; translational elongation	[SUMMARY:] Ribosomes, the organelles that catalyze protein synthesis, consist of a small 40S subunit and a large 60S subunit. Together these subunits are composed of 4 RNA species and approximately 80 structurally distinct proteins. This gene encodes a ribosomal protein that is a component of the 60S subunit. The protein, which is the functional equivalent of the E. coli L10 ribosomal protein, belongs to the L10P family of ribosomal proteins. It is a neutral phosphoprotein with a C-terminal end that is nearly identical to the C-terminal ends of the acidic ribosomal phosphoproteins P1 and P2. The P0 protein can interact with P1 and P2 to form a pentameric complex consisting of P1 and P2 dimers, and a P0 monomer. The protein is located in the cytoplasm. Transcript variants derived from alternative splicing exist; they encode the same protein. As is typical for genes encoding ribosomal proteins, there are multiple processed pseudogenes of this gene dispersed through the genome.	3171.62	4017.3	5118.39	-0.34

211972_x_at	RPLP0	6175	ribosomal protein, large, P0	RNA binding; cytosolic large ribosomal subunit (sensu Eukarya); intracellular; protein biosynthesis; ribosome; structural constituent of ribosome; translational elongation	[SUMMARY:] Ribosomes, the organelles that catalyze protein synthesis, consist of a small 40S subunit and a large 60S subunit. Together these subunits are composed of 4 RNA species and approximately 80 structurally distinct proteins. This gene encodes a ribosomal protein that is a component of the 60S subunit. The protein, which is the functional equivalent of the E. coli L10 ribosomal protein, belongs to the L10P family of ribosomal proteins. It is a neutral phosphoprotein with a C-terminal end that is nearly identical to the C-terminal ends of the acidic ribosomal phosphoproteins P1 and P2. The P0 protein can interact with P1 and P2 to form a pentameric complex consisting of P1 and P2 dimers, and a P0 monomer. The protein is located in the cytoplasm. Transcript variants derived from alternative splicing exist; they encode the same protein. As is typical for genes encoding ribosomal proteins, there are multiple processed pseudogenes of this gene dispersed through the genome.	2466.08	3213.55	4050.73	-0.38
221775_x_at	RPL22	6146	ribosomal protein L22		[SUMMARY:] Ribosomes, the organelles that catalyze protein synthesis, consist of a small 40S subunit and a large 60S subunit. Together these subunits are composed of 4 RNA species and approximately 80 structurally distinct proteins. This gene encodes a cytoplasmic ribosomal protein that is a component of the 60S subunit. The protein belongs to the L22E family of ribosomal proteins. Its initiating methionine residue is post-translationally removed. The protein can bind specifically to Epstein-Barr virus-encoded RNAs (EBERs) 1 and 2. The mouse protein has been shown to be capable of binding to heparin. Transcript variants utilizing alternative polyA signals exist. As is typical for genes encoding ribosomal proteins, there are multiple processed pseudogenes of this gene dispersed through the genome. It was previously thought that this gene mapped to 3q26 and that it was fused to the acute myeloid leukemia 1 (AML1) gene located at 21q22 in some therapy-related myelodysplastic syndrome patients with 3;21 translocations; however, these fusions actually involve a ribosomal protein L22 pseudogene located at 3	1675.53	2389.38	2918.3	-0.51
201134_x_at	COX7C	1350	cytochrome c oxidase subunit VIIc	cytochrome-c oxidase activity; electron transport; energy pathways; mitochondrion; oxidoreductase activity	[SUMMARY:] Cytochrome c oxidase (COX), the terminal component of the mitochondrial respiratory chain, catalyzes the electron transfer from reduced cytochrome c to oxygen. This component is a heteromeric complex consisting of 3 catalytic subunits encoded by mitochondrial genes and multiple structural subunits encoded by nuclear genes. The mitochondrially-encoded subunits function in electron transfer, and the nuclear-encoded subunits may function in the regulation and assembly of the complex. This nuclear gene encodes subunit VIIc, which shares 87% and 85% amino acid sequence identity with mouse and bovine COX VIIc, respectively, and is found in all tissues. A pseudogene COX7CP1 has been found on chromosome 13.	1699.23	2565.25	3076.99	-0.59
201154_x_at	RPL4	6124	ribosomal protein L4	RNA binding; cytosolic large ribosomal subunit (sensu Eukarya); intracellular; protein biosynthesis; ribosome; structural constituent of ribosome	[SUMMARY:] Ribosomes, the organelles that catalyze protein synthesis, consist of a small 40S subunit and a large 60S subunit. Together these subunits are composed of 4 RNA species and approximately 80 structurally distinct proteins. This gene encodes a ribosomal protein that is a component of the 60S subunit. The protein belongs to the L4E family of ribosomal proteins. It is located in the cytoplasm. As is typical for genes encoding ribosomal proteins, there are multiple processed pseudogenes of this gene dispersed through the genome.	1589.65	2424.5	2899.17	-0.61
211710_x_at	RPL4	6124	ribosomal protein L4	RNA binding; cytosolic large ribosomal subunit (sensu Eukarya); intracellular; protein biosynthesis; ribosome; structural constituent of ribosome	[SUMMARY:] Ribosomes, the organelles that catalyze protein synthesis, consist of a small 40S subunit and a large 60S subunit. Together these subunits are composed of 4 RNA species and approximately 80 structurally distinct proteins. This gene encodes a ribosomal protein that is a component of the 60S subunit. The protein belongs to the L4E family of ribosomal proteins. It is located in the cytoplasm. As is typical for genes encoding ribosomal proteins, there are multiple processed pseudogenes of this gene dispersed through the genome.	1616.55	2564.83	3031.76	-0.67
205054_at	NEB	4703	nebulin	actin binding; actin cytoskeleton; biological_process unknown; molecular_function unknown; regulation of actin filament length; sarcomere; somatic muscle development; striated muscle thin filament; structural constituent of muscle	[SUMMARY:] Nebulin is a giant protein component of the cytoskeletal matrix that coexists with the thick and thin filaments within the sarcomeres of skeletal muscle. In most vertebrates, nebulin accounts for 3 to 4% of the total myofibrillar protein and its size varies from 600 to 800 kD in a manner that is tissue-, species-,and developmental stage-specific. The 6,669-residue predicted protein, the short form, contains 185 copies of 35-amino acid modules that can be classified into 7 types. It is suggested that alternative splicing may explain the developmental or tissue-specific size variants of nebulin. However, the nucleotide sequence information is not available for other spliced products. The mutations in the NEB gene are associated with recessive nemaline myopathy.	2145.8	3633.12	4219.49	-0.76
213377_x_at	RPS12	6206	ribosomal protein S12	RNA binding; cytosolic small ribosomal subunit (sensu Eukarya); intracellular; protein biosynthesis; ribosome; structural constituent of ribosome	[SUMMARY:] Ribosomes, the organelles that catalyze protein synthesis, consist of a small 40S subunit and a large 60S subunit. Together these subunits are composed of 4 RNA species and approximately 80 structurally distinct proteins. This gene encodes a ribosomal protein that is a component of the 40S subunit. The protein belongs to the S12E family of ribosomal proteins. It is located in the cytoplasm. Increased expression of this gene in colorectal cancers compared to matched normal colonic mucosa has been observed. As is typical for genes encoding ribosomal proteins, there are multiple processed pseudogenes of this gene dispersed through the genome.	1763.05	3147.53	3607.67	-0.84
208195_at	TTN	7273	titin	ATP binding; Z disc; biological_process unknown; hematopoietin/interferon-class (D200-domain) cytokine receptor activity; membrane; muscle development; myosin binding; protein amino acid phosphorylation; protein serine/threonine kinase activity; sarcomere; striated muscle contraction; structural constituent of muscle; transferase activity	[SUMMARY:] This gene encodes a large abundant protein of striated muscle. The product of this gene is divided into two regions, a N-terminal I-band and a C-terminal A-band. The I-band, which is the elastic part of the molecule, contains two regions of tandem immunoglobulin domains on either side of a PEVK region that is rich in proline, glutamate, valine and lysine. The A-band, which is thought to act as a protein-ruler, contains a mixture of immunoglobulin and fibronectin repeats, and possesses kinase activity. A N-terminal Z-disc region and a C-terminal M-line region bind to the Z-line and M-line of the sarcomere respectively so that a single titin molecule spans half the length of a sarcomere. Titin also contains binding sites for muscle associated proteins so it serves as an adhesion template for the assembly of contractile machinery in muscle cells. It has also been identified as a structural protein for chromosomes. Considerable variability exists in the I-band, the M-line and the Z-disc regions of titin. Variability in the I-band region contributes to the	2178.85	4964.12	5421.25	-1.19
