# Supplementary material for: Unsupervised clustering of gene expression data points at hypoxia as possible trigger for metabolic syndrome
Source: BMC Genomics. 2006 Dec 19;7:318. doi: 10.1186/1471-2164-7-318 (PMC1770922; doi:10.1186/1471-2164-7-318)
Supplement: Additional file 3 — Supplementary Table 2. This PDF file contains the table of Gene Ontology categories overrepresented among genes differentially expressed between the most and the least metabolically sound clusters in our analysis of Mootha et al. data set. [file 1471-2164-7-318-S3.pdf]

EASE RESULTS

System	Category	LH	LT	PH	PT	EASE Score	Fisher Exact
cellular_component	cytoplasm	40	44	2145	7038	1.74E-16	5.75E-17
molecular_function	structural molecule activity	23	49	440	8126	5.73E-16	6.31E-17
cellular_component	cytosolic ribosome (sensu Eukarya)	10	44	32	7038	1.23E-13	1.76E-15
biological_process	muscle development	13	46	101	7825	2.21E-13	8.71E-15
cellular_component	muscle fiber	10	44	35	7038	3.06E-13	4.94E-15
biological_process	muscle contraction	12	46	106	7825	1.15E-11	5.37E-13
cellular_component	ribosome	12	44	103	7038	1.49E-11	7.18E-13
molecular_function	structural constituent of ribosome	12	49	110	8126	2.5E-11	1.25E-12
biological_process	protein biosynthesis	16	46	348	7825	3.99E-10	4.88E-11
molecular_function	structural constituent of muscle	8	49	32	8126	4.79E-10	9.06E-12
biological_process	cell motility	14	46	242	7825	5.2E-10	5.01E-11
cellular_component	cytosol	13	44	200	7038	1.41E-09	1.28E-10
biological_process	striated muscle contraction	7	46	28	7825	8.78E-09	1.63E-10
cellular_component	myofibril	7	44	27	7038	9.74E-09	1.83E-10
cellular_component	sarcomere	7	44	27	7038	9.74E-09	1.83E-10
cellular_component	ribonucleoprotein complex	12	44	192	7038	1.26E-08	1.18E-09
cellular_component	actin cytoskeleton	10	44	153	7038	0.000000257	2.31E-08
biological_process	macromolecule biosynthesis	16	46	570	7825	0.000000318	6.46E-08
cellular_component	cytosolic small ribosomal subunit (sensu Eukarya)	5	44	13	7038	0.00000083	9.37E-09
cellular_component	eukaryotic 48S initiation complex	5	44	13	7038	0.00000083	9.37E-09
biological_process	biosynthesis	16	46	678	7825	0.00000292	0.000000707
cellular_component	intracellular	40	44	4121	7038	0.00000343	0.00000219
molecular_function	RNA binding	12	49	344	8126	0.00000368	0.000000613
cellular_component	cytosolic large ribosomal subunit (sensu Eukarya)	5	44	19	7038	0.00000438	8.24E-08
cellular_component	eukaryotic 43S preinitiation complex	5	44	24	7038	0.0000117	0.000000294
cellular_component	large ribosomal subunit	5	44	26	7038	0.0000164	0.000000451
cellular_component	small ribosomal subunit	5	44	26	7038	0.0000164	0.000000451
cellular_component	striated muscle thin filament	4	44	9	7038	0.0000174	0.000000164
cellular_component	muscle myosin	4	44	15	7038	0.0000919	0.00000173
biological_process	regulation of muscle contraction	4	46	16	7825	0.0000945	0.00000181
biological_process	organogenesis	14	46	696	7825	0.0000973	0.0000276
cellular_component	myosin II	4	44	17	7038	0.000136	0.00000298
cellular_component	cytoskeleton	11	44	435	7038	0.000222	0.0000528
biological_process	morphogenesis	14	46	797	7825	0.00038	0.000123
cellular_component	smooth endoplasmic reticulum	3	44	6	7038	0.000539	0.0000045
cellular_component	hemoglobin complex	3	44	7	7038	0.000751	0.00000784
molecular_function	cytoskeletal protein binding	7	49	187	8126	0.000753	0.000116
molecular_function	actin binding	6	49	146	8126	0.00161	0.000225
cellular_component	myosin	4	44	40	7038	0.00179	0.000103
biological_process	protein metabolism	19	46	1635	7825	0.00268	0.00131
biological_process	translational elongation	3	46	15	7825	0.00324	0.0000823
biological_process	translation	5	46	111	7825	0.00365	0.000453
biological_process	development	14	46	1270	7825	0.0231	0.0118
molecular_function	translation factor activity, nucleic acid binding	3	49	68	8126	0.0608	0.00785
molecular_function	translation regulator activity	3	49	69	8126	0.0624	0.00817
molecular_function	structural constituent of cytoskeleton	3	49	75	8126	0.0722	0.0103
molecular_function	calcium ion binding	6	49	384	8126	0.0747	0.0269
cellular_component	cell	42	44	6178	7038	0.0895	0.0816
molecular_function	nucleic acid binding	16	49	1830	8126	0.103	0.067
molecular_function	motor activity	3	49	94	8126	0.106	0.0188
biological_process	physiological process	42	46	6633	7825	0.163	0.15
biological_process	cellular physiological process	21	46	2909	7825	0.195	0.149
molecular_function	binding	32	49	4944	8126	0.354	0.313
molecular_function	metal ion binding	7	49	826	8126	0.361	0.226
molecular_function	molecular_function unknown	5	49	543	8126	0.401	0.228
molecular_function	protein binding	10	49	1375	8126	0.426	0.311
biological_process	biological_process unknown	4	46	500	7825	0.556	0.339

cellular_component	endoplasmic reticulum	3	44	302	7038	0.557	0.292
cellular_component	cellular_component unknown	4	44	532	7038	0.641	0.428
molecular_function	enzyme regulator activity	3	49	380	8126	0.664	0.404
biological_process	metabolism	25	46	4356	7825	0.681	0.631
molecular_function	purine nucleotide binding	4	49	1009	8126	0.948	0.874
molecular_function	nucleotide binding	4	49	1022	8126	0.951	0.88
biological_process	cellular process	21	46	4393	7825	0.958	0.943
molecular_function	ATP binding	3	49	811	8126	0.96	0.88
molecular_function	adenyl nucleotide binding	3	49	819	8126	0.961	0.884
molecular_function	transporter activity	4	49	1098	8126	0.966	0.913
biological_process	transport	4	46	1198	7825	0.977	0.937
biological_process	response to stimulus	4	46	1230	7825	0.98	0.945
molecular_function	hydrolase activity	4	49	1256	8126	0.985	0.957
biological_process	response to external stimulus	3	46	1033	7825	0.987	0.953
biological_process	organismal physiological process	3	46	1211	7825	0.995	0.981
biological_process	cell growth and/or maintenance	8	46	2478	7825	0.996	0.991
molecular_function	catalytic activity	9	49	2935	8126	0.999	0.998
cellular_component	integral to membrane	3	44	2126	7038	1	1
cellular_component	nucleus	3	44	2079	7038	1	1
cellular_component	membrane	6	44	2745	7038	1	1
