# Supplementary material for: Unsupervised clustering of gene expression data points at hypoxia as possible trigger for metabolic syndrome
Source: BMC Genomics. 2006 Dec 19;7:318. doi: 10.1186/1471-2164-7-318 (PMC1770922; doi:10.1186/1471-2164-7-318)
Supplement: Additional file 5 — Supplementary Table 4. This PDF file contains the table of genes, found differentially expressed between the most and the least metabolically sound clusters in our analysis of Hulver et al. data set. [file 1471-2164-7-318-S5.pdf]

AFFYID	SYMBOL	LOCUSLINK	GENENAME	GENEONTOLOGY	SUMMARY	expr.1(leas	expr.2(mos	difference	logratio
217399_s_at	FOXO3A	2309	forkhead box O3A	apoptosis; cell growth and/or maintenance; cytoplasm; induction of apoptosis; nucleus; regulation of transcription, DNA-dependent; transcription factor activity; transcription from Pol II promoter	[SUMMARY:] This gene belongs to the forkhead family of transcription factors which are characterized by a distinct forkhead domain. This gene likely functions as a trigger for apoptosis through expression of genes necessary for cell death. Translocation of this gene with the MLL gene is associated with secondary acute leukemia. Alternatively spliced transcript variants encoding the same protein have been observed.	84214.03	28399.64	55814.39	1.568188
212870_at	SOS2	6655	son of sevenless homolog 2 (Drosophila)	cellular_component unknown; guanyl-nucleotide exchange factor activity; small GTPase mediated signal transduction		102593.2	49956.96	52636.27	1.038178
214506_at	ADMR	11318	adrenomedullin receptor	G-protein coupled receptor activity, unknown ligand; G-protein coupled receptor protein signaling pathway; integral to membrane; rhodopsin-like receptor activity	[SUMMARY:] This gene encodes a seven-transmembrane receptor that shares sequence similarity with the rat adrenomedullin receptor. This similarity suggests that potential ligands of the encoded protein will be members of the calcitonin gene-related peptide/amylin/adrenomedullin peptide family.	97564.03	46626.86	50937.17	1.065188
208196_x_at	NFATC1	4772	nuclear factor of activated T-cells, cytoplasmic, calcineurin-dependent 1	FK506 binding; cytoplasm; nucleus; regulation of transcription, DNA-dependent; transcription factor activity; transcription from Pol II promoter	[SUMMARY:] The product of this gene is a component of the nuclear factor of activated T cells DNA-binding transcription complex. This complex consists of at least two components: a preexisting cytosolic component that translocates to the nucleus upon T cell receptor (TCR) stimulation, and an inducible nuclear component. Proteins belonging to this family of transcription factors play a central role in inducible gene transcription during immune response. The product of this gene is an inducible nuclear component. It functions as a major molecular target for the immunosuppressive drugs such as cyclosporin A. Five transcript variants encoding distinct isoforms have been identified for this gene. Different isoforms of this protein may regulate inducible expression of different cytokine genes.	107206.3	58276.98	48929.36	0.879392
AFFX-HUMGAPDH/M33197_3_at	GAPD	2597	glyceraldehyde-3-phosphate dehydrogenase	cytoplasm; glucose metabolism; glyceraldehyde-3-phosphate dehydrogenase (phosphorylating) activity; glycolysis; oxidoreductase activity	[SUMMARY:] Glyceraldehyde-3-phosphate dehydrogenase catalyzes an important energy-yielding step in carbohydrate metabolism, the reversible oxidative phosphorylation of glyceraldehyde-3-phosphate in the presence of inorganic phosphate and nicotinamide adenine dinucleotide (NAD). The enzyme exists as a tetramer of identical chains. A GAPD pseudogene has been mapped to Xp21-p11 and 15 GAPD-like loci have been identified.	79876.47	32027.2	47849.27	1.318473

203873_at	SMARCA1	6594	SWI/SNF related, matrix associated, actin dependent regulator of chromatin, subfamily a, member 1		[SUMMARY:] The protein encoded by this gene is a member of the SWI/SNF family of proteins. Members of this family have helicase and ATPase activities and are thought to regulate transcription of certain genes by altering the chromatin structure around those genes. Two transcript variants encoding different isoforms have been found for this gene.	91352.47	47149.5	44202.97	0.954201
212582_at	OSBPL8	114882	oxysterol binding protein-like 8	lipid transport; steroid metabolism	[SUMMARY:] This gene encodes a member of the oxysterol-binding protein (OSBP) family, a group of intracellular lipid receptors. Like most members, the encoded protein contains an N-terminal pleckstrin homology domain and a highly conserved C-terminal OSBP-like sterol-binding domain. Transcript variants encoding different isoforms have been identified, but their full-length natures have not been determined.	83306.26	40020.36	43285.9	1.057691
205389_s_at	ANK1	286	ankyrin 1, erythrocytic	actin cytoskeleton; basolateral plasma membrane; cytoskeletal adaptor activity; cytoskeleton; cytoskeleton organization and biogenesis; enzyme binding; exocytosis; maintenance of epithelial cell polarity; plasma membrane; signal transduction; spectrin binding; structural constituent of cytoskeleton; structural molecule activity	[SUMMARY:] Ankyrins are a family of proteins that are believed to link the integral membrane proteins to the underlying spectrin-actin cytoskeleton and play key roles in activities such as cell motility, activation, proliferation, contact and the maintenance of specialized membrane domains. Multiple isoforms of ankyrin with different affinities for various target proteins are expressed in a tissue-specific, developmentally regulated manner. Most ankyrins are typically composed of three structural domains: an amino-terminal domain containing multiple ankyrin repeats; a central region with a highly conserved spectrin binding domain; and a carboxy-terminal regulatory domain which is the least conserved and subject to variation. Ankyrin 1, the prototype of this family, was first discovered in the erythrocytes, but since has also been found in brain and muscles. Mutations in erythrocytic ankyrin 1 have been associated in approximately half of all patients with hereditary spherocytosis. Complex patterns of alternative splicing in the regulatory domain, giving rise to different	79985.37	45734.06	34251.31	0.806467
210299_s_at	FHL1	2273	four and a half LIM domains 1	cell differentiation; cell growth; cellular_component unknown; molecular_function unknown; muscle development		86193.16	52300.22	33892.95	0.720756
204811_s_at	CACNA2D2	9254	calcium channel, voltage-dependent, alpha 2/delta subunit 2	membrane		85889.6	52197.26	33692.34	0.718509
AFFX-r2-Ec-						81443.63	47926.24	33517.39	0.764986

209889_at	SEC31L2	25956	SEC31-like 2 (S. cerevisiae)		[SUMMARY:] This gene encodes a protein of unknown function. The protein has moderate similarity to rat VAP1 protein which is an endosomal membrane-associated protein, containing a putative Ca2+/calmodulin-dependent kinase II phosphorylation site.	85621.37	52358.58	33262.79	0.709545
209905_at	HOXA9	3205	homeo box A9	biological_process unknown; cell growth and/or maintenance; development; molecular_function unknown; nucleus; regulation of transcription, DNA-dependent; transcription; transcription factor activity; transcriptional activator activity	[SUMMARY:] In vertebrates, the genes encoding the class of transcription factors called homeobox genes are found in clusters named A, B, C, and D on four separate chromosomes. Expression of these proteins is spatially and temporally regulated during embryonic development. This gene is part of the A cluster on chromosome 7 and encodes a DNA-binding transcription factor which may regulate gene expression, morphogenesis, and differentiation. This gene is highly similar to the abdominal-B (Abd-B) gene of Drosophila. A specific translocation event which causes a fusion between this gene and the NUP98 gene has been associated with myeloid leukemogenesis. Two transcript variants encoding different isoforms have been found for this gene.	89253.1	59252.96	30000.14	0.591015
AFFX-HSAC07/X00351_5_at	ACTB	60	actin, beta	actin filament; cell motility; motor activity; structural constituent of cytoskeleton	[SUMMARY:] Beta actin is one of six different actin isoforms which have been identified. ACTB is one of the two nonmuscle cytoskeletal actins. Actins are highly conserved proteins that are involved in cell motility, structure and integrity. Alpha actins are a major constituent of the contractile apparatus.	72701.94	43114.08	29587.86	0.753835
205375_at	MDFI	4188	MyoD family inhibitor	cell differentiation; cytoplasm; cytoplasmic sequestering of transcription factor; embryonic development	[SUMMARY:] This protein is a transcription factor that negatively regulates other myogenic family proteins. Studies of the mouse homolog, I-mf, show that it interfers with myogenic factor function by masking nuclear localization signals and preventing DNA binding. Knockout mouse studies show defects in the formation of vertebrae and ribs that also involve cartilage formation in these structures.	79819.23	51592.62	28226.62	0.629572

211297_s_at	CDK7	1022	cyclin-dependent kinase 7 (MO15 homolog, Xenopus laevis, cdk-activating kinase)	ATP binding; DNA repair; cyclin-dependent protein kinase activity; cytokinesis; nucleus; protein amino acid phosphorylation; regulation of CDK activity; regulation of transcription, DNA-dependent; transcription initiation from Pol II promoter; transferase activity	[SUMMARY:] The protein encoded by this gene is a member of the cyclin-dependent protein kinase (CDK) family. CDK family members are highly similar to the gene products of Saccharomyces cerevisiae cdc28, and Schizosaccharomyces pombe cdc2, and are known to be important regulators of cell cycle progression. This protein forms a trimeric complex with cyclin H and MAT1, which functions as a Cdk-activating kinase (CAK). It is an essential component of the transcription factor TFIIH, that is involved in transcription initiation and DNA repair. This protein is thought to serve as a direct link between the regulation of transcription and the cell cycle.	76318.96	48382.2	27936.76	0.657565
216266_s_at	BIG1	10565	brefeldin A-inhibited guanine nucleotide-exchange protein 1	exocytosis; guanyl-nucleotide exchange factor activity	[SUMMARY:] ADP-ribosylation factors (ARFs) play an important role in intracellular vesicular trafficking. Brefeldin A-inhibited guanine nucleotide-exchange protein 1 (BIG1) is involved in the activation of ARFs by accelerating replacement of bound GDP with GTP. It is an approximately 209 kDa protein involved in Golgi transport. BIG1 contains a Sec7 domain highly similar to BIG2, which may be responsible for the guanine-nucleotide exchange (GEP) activity and also the brefeldin A (BFA) inhibition	51216.56	23939.6	27276.96	1.097211
213454_at	CORT	1325	cortistatin	DNA binding; extracellular; hormone activity	[SUMMARY:] The product of this gene is a neuropeptide with strong structural similarity to somatostatin. It binds to all known somatostatin receptors, and shares many pharmacological and functional properties with somatostatin including the depression of neuronal activity. However, it also has many properties distinct from somatostatin including induction of slow-wave sleep, apparently by antagonism of the excitatory effects of acetylcholine on the cortex, reduction of locomotor activity, and activation of cation selective currents not responsive to somatostatin. Alternatively spliced transcript variants encoding different isoforms have been identified.	74380.77	47295.1	27085.67	0.653239
214688_at	TLE4	7091	transducin-like enhancer of split 4 (E(sp1) homolog, Drosophila)	biological_process unknown; frizzled signaling pathway; molecular_function unknown; nucleus; regulation of transcription, DNA-dependent		77831.9	50856.55	26975.34	0.613928
200967_at	PPIB	5479	peptidylprolyl isomerase B (cyclophilin B)			77718.5	51619.64	26098.86	0.590338

209743_s_at	ITCH	83737	itchy homolog E3 ubiquitin protein ligase (mouse)	ligase activity; nucleus; ubiquitin cycle; ubiquitin-protein ligase activity	[SUMMARY:] Atrophin-1 contains a polyglutamine repeat, expansion of which is responsible for dentatorubral and pallidoluysian atrophy. The protein encoded by this gene interacts with atrophin-1. This encoded protein is a closely related member of the NEDD4-like protein family. This family of proteins are E3 ubiquitin-ligase molecules and regulate key trafficking decisions, including targeting of proteins to proteosomes or lysosomes. This encoded protein contains four tandem WW domains and a HECT (homologous to the E6-associated protein carboxyl terminus) domain. It can act as a transcriptional corepressor of p45/NFE2 and may participate in the regulation of immune responses by modifying Notch-mediated signaling. It is highly similar to the mouse Itch protein, which has been implicated in the regulation and differentiation of erythroid and lymphoid cells.	88592.2	63490.92	25101.28	0.480629
212285_s_at	AGRN	375790	agrin	molecular_function unknown; structural molecule activity	[SUMMARY:] Agrin is a neuronal aggregating factor that induces the aggregation of acetylcholine receptors and other postsynaptic proteins on muscle fibers and is crucial for the formation of the neuromuscular junction.[supplied by OMIM]	77358.03	52734.88	24623.15	0.552793
AFFX-HUMGAPDH/M33197_M_at	GAPD	2597	glyceraldehyde-3-phosphate dehydrogenase	cytoplasm; glucose metabolism; glyceraldehyde-3-phosphate dehydrogenase (phosphorylating) activity; glycolysis; oxidoreductase activity	[SUMMARY:] Glyceraldehyde-3-phosphate dehydrogenase catalyzes an important energy-yielding step in carbohydrate metabolism, the reversible oxidative phosphorylation of glyceraldehyde-3-phosphate in the presence of inorganic phosphate and nicotinamide adenine dinucleotide (NAD). The enzyme exists as a tetramer of identical chains. A GAPD pseudogene has been mapped to Xp21-p11 and 15 GAPD-like loci have been identified.	78804.93	54292.45	24512.48	0.537534
204180_s_at	ZNF297B	23099	zinc finger protein 297B	DNA binding; nucleus; protein binding; regulation of transcription, DNA-dependent		68349.23	44280.2	24069.02	0.626263
205164_at	GCAT	23464	glycine C-acetyltransferase (2-amino-3-ketobutyrate coenzyme A ligase)	5-aminolevulinate synthase activity; acyltransferase activity; amino acid metabolism; biosynthesis; glycine C-acetyltransferase activity; heme biosynthesis; mitochondrion; transaminase activity; transferase activity	[SUMMARY:] The degradation of L-threonine to glycine consists of a two-step biochemical pathway involving the enzymes L-threonine dehydrogenase and 2-amino-3-ketobutyrate coenzyme A ligase. L-Threonine is first converted into 2-amino-3-ketobutyrate by L-threonine dehydrogenase. This gene encodes the second enzyme in this pathway, which then catalyzes the reaction between 2-amino-3-ketobutyrate and coenzyme A to form glycine and acetyl-CoA. The encoded enzyme is considered a class II pyridoxal-phosphate-dependent aminotransferase.	78601.2	55859.66	22741.53	0.492744

201430_s_at	DPYSL3	1809	dihydropyrimidinase-like 3	dihydropyrimidinase activity; hydrolase activity; neurogenesis; nucleobase, nucleoside, nucleotide and nucleic acid metabolism; signal transduction		83176.43	62218.5	20957.93	0.418831
208835_s_at	LUC7A	51747	cisplatin resistance-associated overexpressed protein	RNA splicing; nucleus		69018.43	48787.54	20230.89	0.500469
204632_at	RPS6KA4	8986	ribosomal protein S6 kinase, 90kDa, polypeptide 4	ATP binding; mitogen-activated protein kinase p38 binding; nucleus; protein amino acid phosphorylation; protein kinase cascade; protein serine/threonine kinase activity; regulation of transcription, DNA-dependent; ribosomal protein S6 kinase activity; transferase activity		63530.73	43855.98	19674.76	0.534681
205554_s_at	DNASE1L3	1776	deoxyribonuclease I-like 3	DNA binding; DNA catabolism; apoptosis; calcium ion binding; deoxyribonuclease activity; endonuclease activity; hydrolase activity; nucleus		48131.06	28579.2	19551.86	0.752003
208826_x_at	HINT1	3094	histidine triad nucleotide binding protein 1			67723.57	48601.18	19122.39	0.478667
206394_at	MYBPC2	4606	myosin binding protein C, fast type	actin binding; cell adhesion; muscle development; protein binding; striated muscle contraction; striated muscle thick filament; structural constituent of muscle		58706.33	39721.84	18984.5	0.563584
212655_at	BDG29	23174	BDG-29 proten	nucleic acid binding		58348.8	39763.68	18585.12	0.553252
AFFX-r2-Bs-						60436.11	42533.36	17902.74	0.506816
213202_at	KIAA0339	9739	KIAA0339 gene product	nucleic acid binding		61070	43555.92	17514.08	0.487595
201541_s_at	ZNHIT1	10467	zinc finger, HIT domain containing 1			64493.56	47037.94	17455.63	0.45533
211944_at	XTP2	23215	HBxAg transactivated protein 2			61819.47	44389.98	17429.49	0.477827
216521_s_at						65489.5	48105.48	17384.02	0.445062
203013_at	HSGT1	11319	suppressor of S. cerevisiae gcr2	regulation of glycolysis; transcription coactivator activity; transcription from Pol II promoter		55351.17	39483.2	15867.97	0.487375
201162_at	IGFBP7	3490	insulin-like growth factor binding protein 7	extracellular; insulin-like growth factor binding; negative regulation of cell proliferation; regulation of cell growth		44785.5	29291.72	15493.78	0.612539
206117_at	TPM1	7168	tropomyosin 1 (alpha)	actin binding; biological_process unknown; cellular_component unknown; cytoskeleton; molecular_function unknown; muscle development; muscle thin filament tropomyosin; regulation of heart rate; regulation of muscle contraction; structural constituent of cytoskeleton; structural constituent of muscle	[SUMMARY:] Tropomyosins are ubiquitous proteins of 35 to 45 kD associated with the actin filaments of myofibrils and stress fibers. In vertebrates, 4 known tropomyosin genes code for diverse isoforms that are expressed in a tissue-specific manner and regulated by an alternative splicing mechanism (Lees-Miller and Helfman, 1991 [PubMed 1796905]). The vertebrate alpha-tropomyosin gene consists of 15 exons; 5 exons are found in all transcripts, while 10 exons are alternatively used in different alpha-tropomyosin RNAs (Lees-Miller and Helfman, 1991 [PubMed 1796905]). The striated muscle isoform is expressed in both cardiac and skeletal muscle tissues.[supplied by OMIM]	51518.33	36089.52	15428.81	0.513506
213085_s_at	KIBRA	23286	KIBRA protein			62949.64	47891.58	15058.05	0.394426
201540_at	FHL1	2273	four and a half LIM domains 1	cell differentiation; cell growth; cellular_component unknown; molecular_function unknown; muscle development		60810	46278.8	14531.2	0.393957

205578_at	ROR2	4920	receptor tyrosine kinase-like orphan receptor 2	ATP binding; development; integral to plasma membrane; protein amino acid phosphorylation; signal transduction; transferase activity; transmembrane receptor activity; transmembrane receptor protein tyrosine kinase activity	[SUMMARY:] The protein encoded by this gene is a receptor protein tyrosine kinase and type I transmembrane protein that belongs to the ROR subfamily of cell surface receptors. The protein may be involved in the early formation of the chondrocytes and may be required for cartilage and growth plate development. Mutations in this gene can cause brachydactyly type B, a skeletal disorder characterized by hypoplasia/aplasia of distal phalanges and nails. In addition, mutations in this gene can cause the autosomal recessive form of Robinow syndrome, which is characterized by skeletal dysplasia with generalized limb bone shortening, segmental defects of the spine, brachydactyly, and a dysmorphic facial appearance.	58130.23	43758.96	14371.27	0.40971
205445_at	PRL	5617	prolactin	cell proliferation; cell surface receptor linked signal transduction; extracellular space; hemocyte development; hormone activity; lactation; pregnancy; prolactin receptor binding; soluble fraction		66977.97	52770.1	14207.87	0.343966
209622_at	STK16	8576	serine/threonine kinase 16			52137.77	38043.2	14094.57	0.45469
200764_s_at	CTNNA1	1495	catenin (cadherin-associated protein), alpha 1, 102kDa	cell adhesion; cytoskeleton; protein binding; structural molecule activity		49250.07	35599.58	13650.48	0.468265
AFFX-r2-P1-cre-						46502.7	33782.66	12720.04	0.461031
200964_at	UBE1	7317	ubiquitin-activating enzyme E1 (A1S9T and BN75 temperature sensitivity complementing)	DNA replication; ligase activity; ubiquitin activating enzyme activity; ubiquitin cycle	[SUMMARY:] The protein encoded by this gene catalyzes the first step in ubiquitin conjugation to mark cellular proteins for degradation. This gene complements an X-linked mouse temperature-sensitive defect in DNA synthesis, and thus may function in DNA repair. It is part of a gene cluster on chromosome Xp11.23. Alternative splicing results in 2 transcript variants encoding the same protein, but with different 5' UTR.	41861	29550.76	12310.24	0.502411
205055_at	ITGAE	3682	integrin, alpha E (antigen CD103, human mucosal lymphocyte antigen 1; alpha polypeptide)	cell-matrix adhesion; integral to membrane; integrin complex; integrin-mediated signaling pathway; leukocyte cell adhesion; magnesium ion binding; protein binding; receptor activity	[SUMMARY:] ITGAE encodes integrin alpha chain E. Integrins are heterodimeric integral membrane proteins composed of an alpha chain and a beta chain. This I-domain containing alpha integrin undergoes post-translational cleavage in the extracellular domain that yields disulfide-linked heavy and light chains, In combination with beta 7 (ITGB7) these chains form the E-cadherin binding integrin known as 'human mucosal lymphocyte-1' antigen (HML-1). This integrin is preferentially expressed on human intestinal intraepithelial lymphocytes (IEL), and in addition to adhesion, it may serve as an accessory molecule for IEL activation.	57957.2	45971.46	11985.74	0.334249
AFFX-DapX-						37985.13	26266.96	11718.17	0.532186

202223_at	ITM1	3703	integral membrane protein 1	integral to plasma membrane; membrane; membrane fraction; oligosaccharyl transferase activity; protein amino acid glycosylation		54612.57	43236.48	11376.09	0.336984
201493_s_at	PUM2	23369	pumilio homolog 2 (Drosophila)			56014.14	45055.86	10958.28	0.314076
205178_s_at	RBBP6	5930	retinoblastoma binding protein 6	nucleic acid binding; protein binding; regulation of cell cycle	[SUMMARY:] The retinoblastoma tumor suppressor (pRB) protein binds with many other proteins. In various human cancers, pRB suppresses cellular proliferation and is inactivated. Cell cycle-dependent phosphorylation regulates the activity of pRB. This gene encodes a protein which binds to underphosphorylated but not phosphorylated pRB. Multiple alternatively spliced transcript variants that encode different isoforms have been found for this gene.	49670.5	38879.16	10791.34	0.353392
208981_at	PECAM1	5175	platelet/endothelial cell adhesion molecule (CD31 antigen)	cell motility; cell recognition; integral to membrane; intercellular junction; plasma membrane; protein binding; signal transduction		42241.93	32287.14	9954.797	0.387716
201319_at	MRCL3	10627	myosin regulatory light chain MRCL3			32239.7	22302.14	9937.559	0.531656
201258_at	RPS16	6217	ribosomal protein S16		[SUMMARY:] Ribosomes, the organelles that catalyze protein synthesis, consist of a small 40S subunit and a large 60S subunit. Together these subunits are composed of 4 RNA species and approximately 80 structurally distinct proteins. This gene encodes a ribosomal protein that is a component of the 40S subunit. The protein belongs to the S9P family of ribosomal proteins. It is located in the cytoplasm. As is typical for genes encoding ribosomal proteins, there are multiple processed pseudogenes of this gene dispersed through the genome.	52614.2	43013.04	9601.16	0.290678
201050_at	PLD3	23646	phospholipase D3	catalytic activity; metabolism		48556.86	39088.7	9468.164	0.312924
204893_s_at	MADHIP	9372	MAD, mothers against decapentaplegic homolog (Drosophila) interacting protein, receptor activation anchor	SMAD protein heteromerization; SMAD protein nuclear translocation; cytoplasm; early endosome; endocytosis; protein binding; receptor activity; serine-type peptidase activity; transforming growth factor beta receptor complex assembly; zinc ion binding	[SUMMARY:] MADHIP is a double zinc finger (FYVE domain) protein that interacts directly with SMAD2 and SMAD3, and is involved in Alzheimer's disease. SMAD proteins transmit signals from transmembrane serine/threonine kinase receptors to the nucleus. The FYVE domain has been identified in a number of unrelated signaling molecules. MADHIP functions to recruit SMAD2 to the transforming growth factor-beta receptor. The FYVE domain is required to maintain the normal localization of MADHIP but is not involved in mediating interaction with SMADs. The C-terminal domain of MADHIP interacts with the TGFB receptor. MADHIP is a component of the TGFB pathway that brings the SMAD substrate to the receptor. MADHIP has three known alternatively spliced transcripts that differ in their carboxy termini. One variant also differs in the 5'UTR region.	31726.27	22284.58	9441.688	0.509632

204174_at	ALOX5AP	241	arachidonate 5-lipoxygenase-activating protein	binding; enzyme activator activity; inflammatory response; integral to membrane; leukotriene biosynthesis; membrane fraction	[SUMMARY:] This gene encodes a protein which, with 5-lipoxygenase, is required for leukotriene synthesis. Leukotrienes are arachidonic acid metabolites which have been implicated in various types of inflammatory responses, including asthma, arthritis and psoriasis. This protein localizes to the plasma membrane. Inhibitors of its function impede translocation of 5-lipoxygenase from the cytoplasm to the cell membrane and inhibit 5-lipoxygenase activation.	24233.5	14873.78	9359.721	0.704232
208582_s_at	DUX1	26584	double homeobox, 1	nucleus; regulation of transcription, DNA-dependent; transcription factor activity; transcription from Pol II promoter		8544.966	17859.88	9314.913	-1.063576
201255_x_at	BAT3	7917	HLA-B associated transcript 3		[SUMMARY:] A cluster of genes, BAT1-BAT5, has been localized in the vicinity of the genes for TNF alpha and TNF beta. These genes are all within the human major histocompatibility complex class III region. The protein encoded by this gene is a nuclear protein. It has been implicated in the control of apoptosis and regulating heat shock protein. There are three alternatively spliced transcript variants described for this gene.	33386.63	24144.2	9242.434	0.467594
200675_at	CD81	975	CD81 antigen (target of antiproliferative antibody 1)		[SUMMARY:] The protein encoded by this gene is a member of the transmembrane 4 superfamily, also known as the tetraspanin family. Most of these members are cell-surface proteins that are characterized by the presence of four hydrophobic domains. The proteins mediate signal transduction events that play a role in the regulation of cell development, activation, growth and motility. This encoded protein is a cell surface glycoprotein that is known to complex with integrins. This protein appears to promote muscle cell fusion and support myotube maintenance. Also it may be involved in signal transduction. This gene is localized in the tumor-suppressor gene region and thus it is a candidate gene for malignancies.	44947.17	35719.66	9227.504	0.331512
212392_s_at	PDE4DIP	9659	phosphodiesterase 4D interacting protein (myomegalin)			50836.93	41952.6	8884.332	0.277117
205694_at	TYRP1	7306	tyrosinase-related protein 1	integral to membrane; melanin biosynthesis from tyrosine; metabolism; monooxygenase activity		28478.9	19617.1	8861.803	0.537782

205952_at	KCNK3	3777	potassium channel, subfamily K, member 3	integral to plasma membrane; ion transport; potassium channel activity; potassium ion transport; synaptic transmission; voltage-gated ion channel activity	[SUMMARY:] This gene encodes one of the members of the superfamily of potassium channel proteins containing two pore-forming P domains. The gene product is an outwardly rectifying channel that is sensitive to changes in extracellular pH and is inhibited by extracellular acidification. Also referred to as an acid-sensitive potassium channel, it is activated by the anesthetics halothane and isoflurane. Although three transcripts are detected in northern blots, there is currently no sequence available to confirm transcript variants for this gene.	3706.433	12558.18	8851.747	-1.760524
208646_at	RPS14	6208	ribosomal protein S14	RNA binding; cytosolic small ribosomal subunit (sensu Eukarya); intracellular; protein biosynthesis; ribosome; structural constituent of ribosome	[SUMMARY:] Ribosomes, the organelles that catalyze protein synthesis, consist of a small 40S subunit and a large 60S subunit. Together these subunits are composed of 4 RNA species and approximately 80 structurally distinct proteins. This gene encodes a ribosomal protein that is a component of the 40S subunit. The protein belongs to the S11P family of ribosomal proteins. It is located in the cytoplasm. Transcript variants utilizing alternative transcription initiation sites have been described in the literature. As is typical for genes encoding ribosomal proteins, there are multiple processed pseudogenes of this gene dispersed through the genome. In Chinese hamster ovary cells, mutations in this gene can lead to resistance to emetine, a protein synthesis inhibitor.	38421.67	29710.6	8711.068	0.370942
204738_s_at	CCM1	889	cerebral cavernous malformations 1	cytoskeleton		71679.77	63414.32	8265.445	0.176757
200927_s_at	RAB14	51552	RAB14, member RAS oncogene family	GTP binding; RAB small monomeric GTPase activity; cellular_component unknown; intracellular protein transport; neurotransmitter secretion; protein transporter activity; small GTPase mediated signal transduction; vesicle-mediated transport		49437.03	41239.66	8197.367	0.26156
210300_at	REM	28954	RAS (RAD and GEM)-like GTP-binding	GTP binding; calmodulin binding; small GTPase mediated signal transduction; small monomeric GTPase activity	[SUMMARY:] The protein encoded by this gene is a GTPase and member of the RAS-like GTP-binding protein family. The encoded protein is expressed in endothelial cells, where it promotes reorganization of the actin cytoskeleton and morphological changes in the cells.	31709	23563.3	8145.699	0.428351
206161_s_at	SYT5	6861	synaptotagmin V	integral to membrane; synapse; synaptic transmission; synaptic vesicle; transport; transporter activity		31954.83	24005.98	7948.854	0.41264
200063_s_at	NPM1	4869	nucleophosmin (nucleolar phosphoprotein B23, numatrin)	nucleolus	[Proteome FUNCTION:] Nucleophosmin (nucleolar phosphoprotein B23, numatrin, protein B23); RNA-binding nucleolar phosphoprotein	44849.1	37064.2	7784.898	0.275053

200717_x_at	RPL7	6129	ribosomal protein L7	RNA binding; cytosolic large ribosomal subunit (sensu Eukarya); intracellular; protein biosynthesis; structural constituent of ribosome; transcription regulator activity	[SUMMARY:] Ribosomes, the organelles that catalyze protein synthesis, consist of a small 40S subunit and a large 60S subunit. Together these subunits are composed of 4 RNA species and approximately 80 structurally distinct proteins. This gene encodes a ribosomal protein that is a component of the 60S subunit. The protein belongs to the L30P family of ribosomal proteins. It contains an N-terminal basic region-leucine zipper (BZIP)-like domain and the RNP consensus submotif RNP2. In vitro the BZIP-like domain mediates homodimerization and stable binding to DNA and RNA, with a preference for 28S rRNA and mRNA. The protein can inhibit cell-free translation of mRNAs, suggesting that it plays a regulatory role in the translation apparatus. It is located in the cytoplasm. The protein has been shown to be an autoantigen in patients with systemic autoimmune diseases, such as systemic lupus erythematosus. As is typical for genes encoding ribosomal proteins, there are multiple processed pseudogenes of this gene dispersed through the genome.	47451.33	39781.58	7669.754	0.254348
200096_s_at	ATP6V0E	8992	ATPase, H+ transporting, lysosomal 9kDa, V0 subunit e		[SUMMARY:] This gene encodes a component of vacuolar ATPase (V-ATPase), a multisubunit enzyme that mediates acidification of eukaryotic intracellular organelles. V-ATPase dependent organelle acidification is necessary for such intracellular processes as protein sorting, zymogen activation, and receptor-mediated endocytosis. V-ATPase is comprised of a cytosolic V1 domain and a transmembrane V0 domain. The V1 domain consists of a hexamer of three A and three B subunits plus the C, D, and E subunits. It contains the ATP catalytic site. The encoded protein is part of the V0 subunit. Since two nontranscribed pseudogenes have been found in dog, it is possible that the localization to chromosome 2 for this gene by radiation hybrid mapping is representing a pseudogene. Genomic mapping puts the chromosomal location on 5q35.3.	41049.46	33450.18	7599.285	0.295349
214088_s_at	FUT3	2525	fucosyltransferase 3 (galactoside 3(4)-L-fucosyltransferase, Lewis blood group included)	3-galactosyl-N-acetylglucosaminide 4-alpha-L-fucosyltransferase activity; Golgi apparatus; carbohydrate metabolism; integral to membrane; membrane fraction; protein amino acid glycosylation; transferase activity, transferring glycosyl groups		54213.17	46756.06	7457.109	0.21349
216595_at	FLJ13236	79962	hypothetical protein FLJ13236			4696.8	12024.1	7327.299	-1.356179

211543_s_at	GRK6	2870	G protein-coupled receptor kinase 6	ATP binding; G-protein coupled receptor kinase activity; protein amino acid phosphorylation; regulation of G-protein coupled receptor protein signaling pathway; signal transducer activity; signal transduction; transferase activity	[SUMMARY:] Phosphorylation by receptor-specific and second messenger-activated protein kinases is a mechanism for regulation of G protein-coupled receptors. G protein-coupled receptors are 7-transmembrane domain-containing proteins and are triggered by a variety of signals.[supplied by OMIM]	52193.8	45027.22	7166.582	0.213081
204541_at	SEC14L2	23541	SEC14-like 2 (S. cerevisiae)	carrier activity; cytoplasm; nucleus; phospholipid binding; positive regulation of transcription, DNA-dependent; regulation of cholesterol biosynthesis; tocopherol binding; transcriptional activator activity; transport	[SUMMARY:] This gene encodes a cytosolic protein which belongs to a family of lipid-binding proteins including Sec14p, alpha-tocopherol transfer protein, and cellular retinol-binding protein. The encoded protein stimulates squalene monooxygenase which is a downstream enzyme in the cholesterol biosynthetic pathway.	40992.23	33841.46	7150.777	0.276559
206159_at	GDF10	2662	growth differentiation factor 10	cytokine activity; growth factor activity; skeletal development; transforming growth factor beta receptor signaling pathway	[SUMMARY:] The protein encoded by this gene is a member of the bone morphogenetic protein (BMP) family and the TGF-beta superfamily. This group of proteins is characterized by a polybasic proteolytic processing site which is cleaved to produce a mature protein containing seven conserved cysteine residues. The members of this family are regulators of cell growth and differentiation in both embryonic and adult tissues. Studies in mice suggest that the protein encoded by this gene plays a role in skeletal morphogenesis.	27385.43	20241.96	7143.473	0.43606
201009_s_at	TXNIP	10628	thioredoxin interacting protein	biological_process unknown; cellular_component unknown; molecular_function unknown		20970.93	14012.4	6958.531	0.581687
200818_at	ATP5O	539	ATP synthase, H+ transporting, mitochondrial F1 complex, O subunit (oligomycin sensitivity conferring protein)	ATP biosynthesis; hydrogen ion transporter activity; hydrolase activity; membrane fraction; mitochondrion; proton transport; proton-transporting ATP synthase complex (sensu Eukarya); transporter activity	[SUMMARY:] The protein encoded by this gene is a component of the F-type ATPase found in the mitochondrial matrix. F-type ATPases are composed of a catalytic core and a membrane proton channel. The encoded protein appears to be part of the connector linking these two components and may be involved in transmission of conformational changes or proton conductance.	46483.6	39618.76	6864.836	0.230538

213933_at	PTGER3	5733	prostaglandin E receptor 3 (subtype EP3)	G-protein coupled receptor protein signaling pathway; biological_process unknown; integral to membrane; prostaglandin E receptor activity; receptor activity; rhodopsin-like receptor activity	[SUMMARY:] The protein encoded by this gene is a member of the G-protein coupled receptor family. This protein is one of four receptors identified for prostaglandin E2 (PGE2). This receptor may have many biological functions, which involve digestion, nervous system, kidney reabsorption, and uterine contraction activities. Studies of the mouse counterpart suggest that this receptor may also mediate adrenocorticotropic hormone response as well as fever generation in response to exogenous and endogenous stimuli. Multiple alternatively spliced transcript variants encoding eight distinct isoforms have been reported.	7053.867	13911.98	6858.113	-0.979841
200032_s_at	RPL9	6133	ribosomal protein L9	RNA binding; intracellular; protein biosynthesis; ribosome; structural constituent of ribosome	[SUMMARY:] Ribosomes, the organelles that catalyze protein synthesis, consist of a small 40S subunit and a large 60S subunit. Together these subunits are composed of 4 RNA species and approximately 80 structurally distinct proteins. This gene encodes a ribosomal protein that is a component of the 60S subunit. The protein belongs to the L6P family of ribosomal proteins. It is located in the cytoplasm. As is typical for genes encoding ribosomal proteins, there are multiple processed pseudogenes of this gene dispersed through the genome.	38002.83	31180.2	6822.633	0.285477
213478_at	KIAA1026	23254	KIAA1026 protein			28715.8	21977.84	6737.959	0.385795
1007_s_at	DDR1	780	discoidin domain receptor family, member 1	ATP binding; cell adhesion; integral to plasma membrane; protein amino acid phosphorylation; receptor activity; transferase activity; transmembrane receptor protein tyrosine kinase activity; transmembrane receptor protein tyrosine kinase signaling pathway	[SUMMARY:] Receptor tyrosine kinases (RTKs) play a key role in the communication of cells with their microenvironment. These molecules are involved in the regulation of cell growth, differentiation and metabolism. The protein encoded by this gene is a RTK that is widely expressed in normal and transformed epithelial cells and is activated by various types of collagen. This protein belongs to a subfamily of tyrosine kinase receptors with a homology region to the Dictyostelium discoideum protein discoidin I in their extracellular domain. Its autophosphorylation is achieved by all collagens so far tested (type I to type VI). In situ studies and Northern-blot analysis showed that expression of this encoded protein is restricted to epithelial cells, particularly in the kidney, lung, gastrointestinal tract, and brain. In addition, this protein is significantly over-expressed in several human tumors from breast, ovarian, esophageal, and pediatric brain. This gene is located on chromosome 6p21.3 in proximity to several HLA class I genes. Three isoforms of this gene are generated by alternative splic	32292.37	25592.58	6699.789	0.335468

212274_at	LPIN1	23175	lipin 1	adipocyte differentiation; molecular_function unknown; nucleus	[SUMMARY:] This gene represents a candidate gene for human lipodystrophy, characterized by loss of body fat, fatty liver, hypertriglyceridemia, and insulin resistance. Mouse studies suggest that this gene functions during normal adipose tissue development and may also play a role in human triglyceride metabolism.	38152.73	31458.98	6693.754	0.278315
206118_at	STAT4	6775	signal transducer and activator of transcription 4	JAK-STAT cascade; intracellular signaling cascade; nucleus; regulation of transcription from Pol II promoter; signal transducer activity; transcription factor activity	[SUMMARY:] The protein encoded by this gene is a member of the STAT family of transcription factors. In response to cytokines and growth factors, STAT family members are phosphorylated by the receptor associated kinases, and then form homo- or heterodimers that translocate to the cell nucleus where they act as transcription activators. This protein is essential for mediating responses to IL12 in lymphocytes, and regulating the differentiation of T helper cells.	39275.33	32639.14	6636.191	0.26702
201259_s_at	SYPL	6856	synaptophysin-like protein	integral to plasma membrane; synaptic transmission; synaptic vesicle; transport; transporter activity		20640.57	14208.22	6432.348	0.538757
200726_at	PPP1CC	5501	protein phosphatase 1, catalytic subunit, gamma isoform	cytokinesis; glycogen metabolism; hydrolase activity; manganese ion binding; phosphoprotein phosphatase activity		33571.73	27199.04	6372.689	0.303691
200982_s_at	ANXA6	309	annexin A6		[SUMMARY:] Annexin VI belongs to a family of calcium-dependent membrane and phospholipid binding proteins. Although their functions are still not clearly defined, several members of the annexin family have been implicated in membrane-related events along exocytotic and endocytotic pathways. The annexin VI gene is approximately 60 kbp long and contains 26 exons. It encodes a protein of about 68 kDa that consists of eight 68-amino acid repeats separated by linking sequences of variable lengths. It is highly similar to human annexins I and II sequences, each of which contain four such repeats. Exon 21 of annexin VI is alternatively spliced, giving rise to two isoforms that differ by a 6-amino acid insertion at the start of the seventh repeat. Annexin VI has been implicated in mediating the endosome aggregation and vesicle fusion in secreting epithelia during exocytosis.	41190.97	34918.36	6272.605	0.238342
213970_at	RABL3	285282	RAB, member of RAS oncogene family-like 3	GTP binding; small GTPase mediated signal transduction; small monomeric GTPase activity		33659.14	27438.9	6220.236	0.294776

215314_at	ANK3	288	ankyrin 3, node of Ranvier (ankyrin G)	Golgi apparatus; cellular_component unknown; cytoskeletal anchoring; cytoskeleton; endoplasmic reticulum; molecular_function unknown; protein targeting; signal transduction; structural constituent of cytoskeleton	[SUMMARY:] Ankyrins are a family of proteins that are believed to link the integral membrane proteins to the underlying spectrin-actin cytoskeleton and play key roles in activities such as cell motility, activation, proliferation, contact, and the maintenance of specialized membrane domains. Multiple isoforms of ankyrin with different affinities for various target proteins are expressed in a tissue-specific, developmentally regulated manner. Most ankyrins are typically composed of three structural domains: an amino-terminal domain containing multiple ankyrin repeats; a central region with a highly conserved spectrin binding domain; and a carboxy-terminal regulatory domain which is the least conserved and subject to variation. Ankyrin 3 is an immunologically distinct gene product from ankyrins 1 and 2, and was originally found at the axonal initial segment and nodes of Ranvier of neurons in the central and peripheral nervous systems. Alternatively spliced variants may be expressed in other tissues. Although multiple transcript variants encoding several different i	6017.233	12203.1	6185.867	-1.020076
204152_s_at	MFNG	4242	manic fringe homolog (Drosophila)		[SUMMARY:] This gene is a member of the fringe gene family which also includes Radical and Lunatic fringe. They all encode evolutionarily conserved secreted proteins that act in Notch receptor pathway to demarcate boundaries during embryonic development. The genomic structure of each fringe gene consists of eight exons. Fringe proteins are related to a family of glycosyltransferase, but their genomic structure is distinct from the glycosyltransferases.	6062.934	12217	6154.066	-1.010802
200082_s_at	RPS7	6201	ribosomal protein S7	RNA binding; cytosolic small ribosomal subunit (sensu Eukarya); intracellular; protein biosynthesis; ribosome; structural constituent of ribosome	[SUMMARY:] Ribosomes, the organelles that catalyze protein synthesis, consist of a small 40S subunit and a large 60S subunit. Together these subunits are composed of 4 RNA species and approximately 80 structurally distinct proteins. This gene encodes a ribosomal protein that is a component of the 40S subunit. The protein belongs to the S7E family of ribosomal proteins. It is located in the cytoplasm. As is typical for genes encoding ribosomal proteins, there are multiple processed pseudogenes of this gene dispersed through the genome.	21790.2	15831.26	5958.94	0.460903
211697_x_at	LOC56902	56902	putatative 28 kDa protein	nucleic acid binding		19356.87	25292.98	5936.115	-0.385892

200824_at	GSTP1	2950	glutathione S-transferase pi		[SUMMARY:] Glutathione S-transferases (GSTs) are a family of enzymes that play an important role in detoxification by catalyzing the conjugation of many hydrophobic and electrophilic compounds with reduced glutathione. Based on their biochemical, immunologic, and structural properties, the soluble GSTs are categorized into 4 main classes: alpha, mu, pi, and theta. The glutathione S-transferase pi gene (GSTP1) is a polymorphic gene encoding active, functionally different GSTP1 variant proteins that are thought to function in xenobiotic metabolism and play a role in susceptibility to cancer, and other diseases.	23253.13	17469.06	5784.074	0.412623
204084_s_at	CLN5	1203	ceroid-lipofuscinosis, neuronal 5	cell growth and/or maintenance; integral to membrane; lysosome	[SUMMARY:] The neuronal ceroid lipofuscinoses (CLN or NCL) are a group of autosomal recessive, progressive encephalopathies in children. They are characterized by psychomotor deterioration, visual failure, and the accumulation of autofluorescent lipopigment in neurons and other cell types. The main childhood forms are the infantile type (Santavuori-Haltia disease; MIM 256730), the late infantile type (Jansky-Bielschowsky disease; MIM 204500), and the juvenile type (Batten disease; MIM 204200) based on the age of onset, clinical course, neurologic and ophthalmologic findings, and ultrastructural analysis (Carpenter et al., 1977 [PubMed 193610]).[supplied by OMIM]	43916.7	38158.84	5757.855	0.202752
202030_at	BCKDK	10295	branched chain alpha-ketoacid dehydrogenase kinase	ATP binding; [3-methyl-2-oxobutanoate dehydrogenase (lipoamide)] kinase activity; alpha-ketoglutarate dehydrogenase complex (sensu Eukarya); kinase activity; mitochondrion; signal transduction; transferase activity; two-component sensor molecule activity		37014.04	31273.2	5740.836	0.243146
200092_s_at	RPL37	6167	ribosomal protein L37		[SUMMARY:] Ribosomes, the organelles that catalyze protein synthesis, consist of a small 40S subunit and a large 60S subunit. Together these subunits are composed of 4 RNA species and approximately 80 structurally distinct proteins. This gene encodes a ribosomal protein that is a component of the 60S subunit. The protein belongs to the L37E family of ribosomal proteins. It is located in the cytoplasm. The protein contains a C2C2-type zinc finger-like motif. As is typical for genes encoding ribosomal proteins, there are multiple processed pseudogenes of this gene dispersed through the genome.	20505.77	14808.08	5697.688	0.469645

204327_s_at	ZNF202	7753	zinc finger protein 202	lipid metabolism; negative regulation of transcription from Pol II promoter; nucleus; regulation of transcription, DNA-dependent; specific RNA polymerase II transcription factor activity; transcription factor activity		7694.534	13375.06	5680.526	-0.79764
AFFX-CreX-						20531.17	14897.1	5634.066	0.462784
207877_s_at	NVL	4931	nuclear VCP-like	ATP binding; nucleus		15010.47	20616.18	5605.716	-0.457808
208813_at	GOT1	2805	glutamic-oxaloacetic transaminase 1, soluble (aspartate aminotransferase 1)		[SUMMARY:] Glutamic-oxaloacetic transaminase is a pyridoxal phosphate-dependent enzyme which exists in cytoplasmic and mitochondrial forms, GOT1 and GOT2, respectively. GOT plays a role in amino acid metabolism and the urea and tricarboxylic acid cycles. The two enzymes are homodimeric and show close homology.	3950.2	9501.521	5551.32	-1.266233
213378_s_at		338812	similar to DEAD/H (Asp-Glu-Ala-Asp/His) box polypeptide 11; DEAD/H box-11 (CHL1-related helicase gene-1); yeast CHL1 homolog			30871.9	25342	5529.9	0.284764
213615_at	C3F	10162	putative protein similar to nessy (Drosophila)		[Proteome FUNCTION:] Low similarity to Hs.78768 BB1	26765.33	21242.46	5522.875	0.333415
203296_s_at	ATP1A2	477	ATPase, Na+/K+ transporting, alpha 2 (+) polypeptide	ATP binding; ATP hydrolysis coupled proton transport; hydrogen ion homeostasis; hydrolase activity; hydrolase activity, acting on acid anhydrides, catalyzing transmembrane movement of substances; magnesium ion binding; metabolism; monovalent inorganic cation transporter activity; potassium ion transport; sodium ion transport; sodium/potassium-exchanging ATPase activity; sodium/potassium-exchanging ATPase complex; sperm motility		14210.33	8702.88	5507.453	0.707376

212391_x_at	RPS3A	6189	ribosomal protein S3A		[SUMMARY:] Ribosomes, the organelles that catalyze protein synthesis, consist of a small 40S subunit and a large 60S subunit. Together these subunits are composed of 4 RNA species and approximately 80 structurally distinct proteins. This gene encodes a ribosomal protein that is a component of the 40S subunit. The protein belongs to the S3AE family of ribosomal proteins. It is located in the cytoplasm. Disruption of the gene encoding rat ribosomal protein S3a, also named v-fos transformation effector protein, in v-fos-transformed rat cells results in reversion of the transformed phenotype. Transcript variants utilizing alternative transcription start sites have been described. This gene is co-transcribed with the U73A and U73B small nucleolar RNA genes, which are located in its fourth and third introns, respectively. As is typical for genes encoding ribosomal proteins, there are multiple processed pseudogenes of this gene dispersed through the genome.	29852.9	24373.2	5479.701	0.292575
200781_s_at	RPS15A	6210	ribosomal protein S15a	3'-5'-exoribonuclease activity; RNA binding; RNA processing	[SUMMARY:] Ribosomes, the organelles that catalyze protein synthesis, consist of a small 40S subunit and a large 60S subunit. Together these subunits are composed of 4 RNA species and approximately 80 structurally distinct proteins. This gene encodes a ribosomal protein that is a component of the 40S subunit. The protein belongs to the S8P family of ribosomal proteins. It is located in the cytoplasm. As is typical for genes encoding ribosomal proteins, there are multiple processed pseudogenes of this gene dispersed through the genome.	39033.27	33569.94	5463.324	0.217534

200033_at	DDX5	1655	DEAD (Asp-Glu-Ala-Asp) box polypeptide 5	ATP binding; ATP-dependent helicase activity; RNA binding; RNA helicase activity; cell growth; nucleus	[SUMMARY:] DEAD box proteins, characterized by the conserved motif Asp-Glu-Ala-Asp (DEAD), are putative RNA helicases. They are implicated in a number of cellular processes involving alteration of RNA secondary structure, such as translation initiation, nuclear and mitochondrial splicing, and ribosome and spliceosome assembly. Based on their distribution patterns, some members of this family are believed to be involved in embryogenesis, spermatogenesis, and cellular growth and division. This gene encodes a DEAD box protein, which is a RNA-dependent ATPase, and also a proliferation-associated nuclear antigen, specifically reacting with the simian virus 40 tumor antigen. This gene consists of 13 exons, and alternatively spliced transcripts containing several intron sequences have been detected, but no isoforms encoded by these transcripts have been identified.	37182.04	31721.4	5460.635	0.229149
205590_at	RASGRP1	10125	RAS guanyl releasing protein 1 (calcium and DAG-regulated)	RAS protein signal transduction; Ras guanyl-nucleotide exchange factor activity; calcium ion binding; lipid binding; membrane fraction	[SUMMARY:] RAS guanyl nucleotide-releasing protein (RASGRP) is a member of a family of genes characterized by the presence of a Ras superfamily guanine nucleotide exchange factor (GEF) domain. The corresponding rat gene rbc7, which lacks a 5-prime exon, represents a 5-prime and 3-prime truncated version of a larger normal rat transcript that encodes a predicted 90-kD protein. This shorter transcript has not been found in humans.	24630.17	19173.7	5456.467	0.361298
200027_at	NARS	4677	asparaginyl-tRNA synthetase		[SUMMARY:] Aminoacyl-tRNA synthetases are a class of enzymes that charge tRNAs with their cognate amino acids. Asparaginyl-tRNA synthetase is localized to the cytoplasm and belongs to the class II family of tRNA synthetases. The N-terminal domain represents the signature sequence for the eukaryotic asparaginyl-tRNA synthetases.	23966.77	18547.38	5419.385	0.36982
217729_s_at	AES	166	amino-terminal enhancer of split	Wnt receptor signaling pathway; development; nucleus; organogenesis; regulation of transcription, DNA-dependent	[SUMMARY:] The protein encoded by this gene is similar in sequence to the amino terminus of Drosophila enhancer of split groucho, a protein involved in neurogenesis during embryonic development. The encoded protein, which belongs to the groucho/TLE family of proteins, can function as a homooligomer or as a heteroologimer with other family members to dominantly repress the expression of other family member genes. Three transcript variants encoding different isoforms have been found for this gene.	10772.4	5377.44	5394.96	1.002348

209141_at	UBE2G1	7326	ubiquitin-conjugating enzyme E2G 1 (UBC7 homolog, C. elegans)		[SUMMARY:] The modification of proteins with ubiquitin is an important cellular mechanism for targeting abnormal or short-lived proteins for degradation. Ubiquitination involves at least three classes of enzymes: ubiquitin-activating enzymes, or E1s, ubiquitin-conjugating enzymes, or E2s, and ubiquitin-protein ligases, or E3s. This gene encodes a member of the E2 ubiquitin-conjugating enzyme family. The encoded protein shares 98-100% sequence identity with the zebrafish, frog, rat and mouse counterparts, which indicates that this enzyme is highly conserved in eukaryotes. Two alternatively spliced transcript variants encoding distinct isoforms have been found for this gene.	4368	9746.761	5378.761	-1.15795
217233_at						13859.83	19234.14	5374.308	-0.472759
211654_x_at	HLA-DQB1	3119	major histocompatibility complex, class II, DQ beta 1	MHC class II receptor activity; antigen presentation, exogenous antigen; antigen processing, exogenous antigen via MHC class II; immune response; integral to membrane	[SUMMARY:] HLA-DQB1 belongs to the HLA class II beta chain paralogues. This class II molecule is a heterodimer consisting of an alpha (DQA) and a beta chain (DQB), both anchored in the membrane. It plays a central role in the immune system by presenting peptides derived from extracellular proteins. Class II molecules are expressed in antigen presenting cells (APC: B lymphocytes, dendritic cells, macrophages). The beta chain is approximately 26-28 kDa and it contains 6 exons. Exon one encodes the leader peptide, exons 2 and 3 encode the two extracellular domains, exon 4 encodes the transmembrane domain and exon 5 encodes the cytoplasmic tail. Within the DQ molecule both the alpha chain and the beta chain contain the polymorphisms specifying the peptide binding specificities, resulting in up to 4 different molecules. Typing for these polymorphisms is routinely done for bone marrow transplantation.	4880.5	10209.1	5328.6	-1.064755

208692_at	RPS3	6188	ribosomal protein S3	RNA binding; cytosolic small ribosomal subunit (sensu Eukarya); intracellular; protein biosynthesis; structural constituent of ribosome	[SUMMARY:] Ribosomes, the organelles that catalyze protein synthesis, consist of a small 40S subunit and a large 60S subunit. Together these subunits are composed of 4 RNA species and approximately 80 structurally distinct proteins. This gene encodes a ribosomal protein that is a component of the 40S subunit, where it forms part of the domain where translation is initiated. The protein belongs to the S3P family of ribosomal proteins. Studies of the mouse and rat proteins have demonstrated that the protein has an extraribosomal role as an endonuclease involved in the repair of UV-induced DNA damage. The protein appears to be located in both the cytoplasm and nucleus but not in the nucleolus. Higher levels of expression of this gene in colon adenocarcinomas and adenomatous polyps compared to adjacent normal colonic mucosa have been observed. This gene is co-transcribed with the small nucleolar RNA genes U15A and U15B, which are located in its first and fifth introns, respectively. As is typical for genes encoding ribosomal proteins, there are multiple processed pseudo	3745.567	9064.521	5318.954	-1.275047
217741_s_at	ZNF216	7763	zinc finger protein 216	DNA binding; biological_process unknown; cellular_component unknown; molecular_function unknown; zinc ion binding		29216.33	23929.32	5287.016	0.287996
210647_x_at	PLA2G6	8398	phospholipase A2, group VI (cytosolic, calcium-independent)	catalytic activity; cytoplasm; hydrolase activity; lipid catabolism; membrane; nutrient reservoir activity; phospholipase A2 activity; phospholipid metabolism		55863.9	50584.06	5279.844	0.143234
212791_at	FLJ38984	127703	hypothetical protein FLJ38984			49083.3	43828.08	5255.219	0.163377
219773_at	NOX4	50507	NADPH oxidase 4	electron transport; electron transporter activity; energy pathways; membrane; nucleotide binding; oxidoreductase activity	[SUMMARY:] Oxygen sensing is essential for homeostasis in all aerobic organisms. A phagocyte-type oxidase, similar to that responsible for the production of large amounts of reactive oxygen species (ROS) in neutrophil granulocytes, with resultant antimicrobial activity, has been postulated to function in the kidney as an oxygen sensor that regulates the synthesis of erythropoietin (EPO; MIM 133170) in the renal cortex.[supplied by OMIM]	26349.63	21150.6	5199.033	0.317084

214630_at	CYP11B1	1584	cytochrome P450, family 11, subfamily B, polypeptide 1	C21-steroid hormone biosynthesis; electron transport; membrane; mitochondrion; monooxygenase activity; steroid 11-beta-monooxygenase activity	[SUMMARY:] This gene encodes a member of the cytochrome P450 superfamily of enzymes. The cytochrome P450 proteins are monooxygenases which catalyze many reactions involved in drug metabolism and synthesis of cholesterol, steroids and other lipids. This protein localizes to the mitochondrial inner membrane and is involved in the conversion of progesterone to cortisol in the adrenal cortex. Mutations in this gene cause congenital adrenal hyperplasia due to 11-beta-hydroxylase deficiency.	22741.03	17553.94	5187.092	0.373503
212735_at	KIAA0226	9711	KIAA0226 gene product			31349.9	26309.9	5040	0.252855
213685_at	TCEB3	6924	transcription elongation factor B (SIII), polypeptide 3 (110kDa, elongin A)	defense response; nucleus; protein binding; regulation of transcription from Pol II promoter; transcription; transcriptional elongation regulator activity	[SUMMARY:] This gene encodes the protein elongin A, which is a subunit of the transcription factor B (SIII) complex. The SIII complex is composed of elongins A/A2, B and C. It activates elongation by RNA polymerase II by suppressing transient pausing of the polymerase at many sites within transcription units. Elongin A functions as the transcriptionally active component of the SIII complex, whereas elongins B and C are regulatory subunits. Elongin A2 is specifically expressed in the testis, and capable of forming a stable complex with elongins B and C. The von Hippel-Lindau tumor suppressor protein binds to elongins B and C, and thereby inhibits transcription elongation.	29163.37	24130.06	5033.309	0.273326
209700_x_at	PDE4DIP	9659	phosphodiesterase 4D interacting protein (myomegalin)			4394.867	9368.78	4973.914	-1.092042
221052_at	TDRKH	11022	tudor and KH domain containing	RNA binding		6164.867	1198.58	4966.287	2.362743
200093_s_at	HINT1	3094	histidine triad nucleotide binding protein 1			27120.33	22154.26	4966.072	0.291791
216527_at						6099.534	11021.92	4922.386	-0.853605
205133_s_at	HSPE1	3336	heat shock 10kDa protein 1 (chaperonin 10)			9418.333	4517.88	4900.453	1.059826
212552_at	HPCAL1	3241	hippocalcin-like 1	actin binding; calcium ion binding; clathrin binding; clathrin coat of trans-Golgi network vesicle; cytosol; tubulin binding; vesicle-mediated transport	[SUMMARY:] The protein encoded by this gene is a member of neuron-specific calcium-binding proteins family found in the retina and brain. It is highly similar to human hippocalcin protein and nearly identical to the rat and mouse hippocalcin like-1 proteins. It may be involved in the calcium-dependent regulation of rhodopsin phosphorylation and may be of relevance for neuronal signalling in the central nervous system. There are two alternatively spliced transcript variants of this gene, with multiple polyadenylation sites.	15543.07	10675.84	4867.228	0.541922

200062_s_at	RPL30	6156	ribosomal protein L30		[SUMMARY:] Ribosomes, the organelles that catalyze protein synthesis, consist of a small 40S subunit and a large 60S subunit. Together these subunits are composed of 4 RNA species and approximately 80 structurally distinct proteins. This gene encodes a ribosomal protein that is a component of the 60S subunit. The protein belongs to the L30E family of ribosomal proteins. It is located in the cytoplasm. This gene is co-transcribed with the U72 small nucleolar RNA gene, which is located in its fourth intron. As is typical for genes encoding ribosomal proteins, there are multiple processed pseudogenes of this gene dispersed through the genome.	37526.63	32681.72	4844.914	0.199431
210969_at	PRKCL2	5586	protein kinase C-like 2	ATP binding; intracellular; protein amino acid phosphorylation; protein serine/threonine kinase activity; signal transduction; transferase activity		22960.83	18172.36	4788.473	0.337429
209117_at	WBP2	23558	WW domain binding protein 2	protein binding; signal transduction	[SUMMARY:] The globular WW domain is composed of 38 to 40 semiconserved amino acids shared by proteins of diverse functions including structural, regulatory, and signaling proteins. The domain is involved in mediating protein-protein interactions through the binding of polyproline ligands. This gene encodes a WW domain binding protein, which binds to the WW domain of Yes kinase-associated protein by its PY motifs. The function of this protein has not been determined.	15290.5	19928.14	4637.641	-0.382172
200820_at	PSMD8	5714	proteasome (prosome, macropain) 26S subunit, non-ATPase, 8	proteasome regulatory particle (sensu Eukarya); proteolysis and peptidolysis; regulation of cell cycle	[SUMMARY:] The 26S proteasome is a multicatalytic proteinase complex with a highly ordered structure composed of 2 complexes, a 20S core and a 19S regulator. The 20S core is composed of 4 rings of 28 non-identical subunits; 2 rings are composed of 7 alpha subunits and 2 rings are composed of 7 beta subunits. The 19S regulator is composed of a base, which contains 6 ATPase subunits and 2 non-ATPase subunits, and a lid, which contains up to 10 non-ATPase subunits. Proteasomes are distributed throughout eukaryotic cells at a high concentration and cleave peptides in an ATP/ubiquitin-dependent process in a non-lysosomal pathway. An essential function of a modified proteasome, the immunoproteasome, is the processing of class I MHC peptides. This gene encodes a non-ATPase subunit of the 19S regulator. A pseudogene has been identified on chromosome 1.	34525	29888.62	4636.379	0.208045

201135_at	ECHS1	1892	enoyl Coenzyme A hydratase, short chain, 1, mitochondrial	energy pathways; enoyl-CoA hydratase activity; fatty acid beta-oxidation; fatty acid metabolism; lyase activity; mitochondrion	[SUMMARY:] The protein encoded by this gene functions in the second step of the mitochondrial fatty acid beta-oxidation pathway. It catalyzes the hydration of 2-trans-enoyl-coenzyme A (CoA) intermediates to L-3-hydroxyacyl-CoAs. The gene product is a member of the hydratase/isomerase superfamily. It localizes to the mitochondrial matrix. Transcript variants utilizing alternative transcription initiation sites have been described in the literature.	29011.57	24379.32	4632.248	0.25097
200003_s_at	RPL28	6158	ribosomal protein L28		[SUMMARY:] Ribosomes, the organelles that catalyze protein synthesis, consist of a small 40S subunit and a large 60S subunit. Together these subunits are composed of 4 RNA species and approximately 80 structurally distinct proteins. This gene encodes a ribosomal protein that is a component of the 60S subunit. The protein belongs to the L28E family of ribosomal proteins. It is located in the cytoplasm. Variable expression of this gene in colorectal cancers compared to adjacent normal tissues has been observed, although no correlation between the level of expression and the severity of the disease has been found. As is typical for genes encoding ribosomal proteins, there are multiple processed pseudogenes of this gene dispersed through the genome.	22142.57	17519.96	4622.609	0.337823
203035_s_at	PIAS3	10401	protein inhibitor of activated STAT3	DNA binding; nucleus; transcription corepressor activity; zinc ion binding		38035.4	33444.34	4591.063	0.185581

202647_s_at	NRAS	4893	neuroblastoma RAS viral (v-ras) oncogene homolog	GTP binding; cell growth and/or maintenance; regulation of cell cycle; small GTPase mediated signal transduction; small monomeric GTPase activity	[SUMMARY:] The N-ras oncogene is a member of the RAS gene family. It is mapped on chromosome 1, and it is activated in HL60, a promyelocytic leukemia line. The order of nearby genes is as follows: cen--CD2--NGFB--NRAS--tel. The mammalian ras gene family consists of the harvey and kirsten ras genes (c-Hras1 and c-Kras2), an inactive pseudogene of each (c-Hras2 and c-Kras1) and the N-ras gene. They differ significantly only in the C-terminal 40 amino acids. These ras genes have GTP/GDP binding and GTPase activity, and their normal function may be as G-like regulatory proteins involved in the normal control of cell growth. Mutations which change amino acid residues 12, 13 or 61 activate the potential of N-ras to transform cultured cells and are implicated in a variety of human tumors. The N-ras gene specifies two main transcripts of 2Kb and 4.3Kb. The difference between the two transcripts is a simple extension through the termination site of the 2Kb transcript. The N-ras gene consists of seven exons (-I, I, II, III, IV, V, VI). The smaller 2Kb transcript conta	21657.8	17077.42	4580.381	0.342797
201969_at	NASP	4678	nuclear autoantigenic sperm protein (histone-binding)	DNA packaging; nucleus; spermatogenesis	[SUMMARY:] This gene encodes a H1 histone binding protein that is involved in transporting histones into the nucleus of dividing cells. Multiple isoforms are encoded by transcript variants of this gene. The somatic form is expressed in all mitotic cells, is localized to the nucleus, and is coupled to the cell cycle. The testicular form is expressed in embryonic tissues, tumor cells, and the testis. In male germ cells, this protein is localized to the cytoplasm of primary spermatocytes, the nucleus of spermatids, and the periacrosomal region of mature spermatozoa.	32582.07	28016.72	4565.35	0.21779
210533_at	MSH4	4438	mutS homolog 4 (E. coli)	ATP binding; damaged DNA binding; meiosis; meiotic recombination; mismatch repair; nucleus		18347.67	22896.06	4548.393	-0.319503

200020_at	TARDBP	23435	TAR DNA binding protein	RNA binding; microtubule binding; mitosis; nuclear mRNA splicing, via spliceosome; nucleus; regulation of transcription, DNA-dependent; transcription factor activity; transcription from Pol II promoter	[SUMMARY:] HIV-1, the causative agent of acquired immunodeficiency syndrome (AIDS), contains an RNA genome that produces a chromosomally integrated DNA during the replicative cycle. Activation of HIV-1 gene expression by the transactivator Tat is dependent on an RNA regulatory element (TAR) located downstream of the transcription initiation site. The protein encoded by this gene is a transcriptional repressor that binds to chromosomally integrated TAR DNA and represses HIV-1 transcription. In addition, this protein regulates alternate splicing of the CFTR gene. A similar pseudogene is present on chromosome 20.	17150.07	12608.26	4541.807	0.443845
203567_s_at	TRIM38	10475	tripartite motif-containing 38	biological_process unknown; cellular_component unknown; intracellular; molecular_function unknown; zinc ion binding	[SUMMARY:] The protein encoded by this gene is a member of the tripartite motif (TRIM) family. The TRIM motif includes three zinc-binding domains, a RING, a B-box type 1 and a B-box type 2, and a coiled-coil region. The function of this protein has not been identified.	17316.63	12784.7	4531.933	0.43774
213891_s_at	TCF4	6925	transcription factor 4	DNA binding; RNA polymerase II transcription factor activity; nucleus; regulation of transcription from Pol II promoter	[SUMMARY:] TCF4 encodes transcription factor 4, a basic helix-turn-helix transcription factor. The protein recognizes an Ephrussi-box ('E-box') binding site ('CANNTG') - a motif first identified in immunoglobulin enhancers. TCF4 is expressed predominantly in pre-B-cells, although it is found in other tissues as well. TCF4 is known to produce multiple transcripts; however as the complete structure is only known for the transcript that encodes the b isoform, that is the variant presented here.	33043.6	28515.88	4527.717	0.212605
214460_at	LSAMP	4045	limbic system-associated membrane protein	cell adhesion; membrane fraction; neurogenesis; protein binding	[SUMMARY:] Limbic system-associated membrane protein is a neuronal surface glycoprotein found in cortical and subcortical regions of the limbic system. During development of the limbic system, LSAMP is found on the surface of axonal membranes and growth cones, where it acts as a selective homophilic adhesion molecule, and guides the development of specific patterns of neuronal connections.	3467.533	7907.399	4439.866	-1.189294
205873_at	PIGL	9487	phosphatidylinositol glycan, class L	GPI anchor biosynthesis; N-acetylglucosaminylphosphatidylinositol deacetylase activity; endoplasmic reticulum; hydrolase activity; integral to membrane	[SUMMARY:] This gene encodes an enzyme that catalyzes the second step of glycosylphosphatidylinositol (GPI) biosynthesis, which is the de-N-acetylation of N-acetylglucosaminylphosphatidylinositol (GlcNAc-PI). Study of a similar rat enzyme suggests that this protein localizes to the endoplasmic reticulum.	18340.37	13916.16	4424.206	0.398261

221995_s_at	MRP63	78988	mitochondrial ribosomal protein 63	structural constituent of ribosome	[SUMMARY:] Mammalian mitochondrial ribosomal proteins are encoded by nuclear genes and help in protein synthesis within the mitochondrion. Mitochondrial ribosomes (mitoribosomes) consist of a small 28S subunit and a large 39S subunit. They have an estimated 75% protein to rRNA composition compared to prokaryotic ribosomes, where this ratio is reversed. Another difference between mammalian mitoribosomes and prokaryotic ribosomes is that the latter contain a 5S rRNA. Among different species, the proteins comprising the mitoribosome differ greatly in sequence, and sometimes in biochemical properties, which prevents easy recognition by sequence homology. This gene encodes a protein which belongs to an undetermined ribosomal subunit and which seems to be specific to animal mitoribosomes. Pseudogenes corresponding to this gene are found on chromosomes 1p, 1q, 3p, 5q, 8q, 14q, and Y.	20096.67	15699.46	4397.209	0.356241
219510_at	POLQ	10721	polymerase (DNA directed), theta			41389.3	37014.6	4374.699	0.161163
200007_at	SRP14	6727	signal recognition particle 14kDa (homologous Alu RNA binding protein)	7S RNA binding; cotranslational membrane targeting; protein targeting; signal recognition particle		16086.17	20411.16	4324.993	-0.343538
211928_at	DNCH1	1778	dynein, cytoplasmic, heavy polypeptide 1	ATP binding; ATPase activity, coupled; cytoplasmic dynein complex; microtubule motor activity; microtubule-based movement; mitotic spindle assembly; nucleotide binding		46563.97	42253.08	4310.887	0.140157
215001_s_at	GLUL	2752	glutamate-ammonia ligase (glutamine synthase)	glutamate-ammonia ligase activity; glutamine biosynthesis; ligase activity; nitrogen fixation; regulation of neurotransmitter levels		21030.53	25311.44	4280.908	-0.267304
201106_at	GPX4	2879	glutathione peroxidase 4 (phospholipid hydroperoxidase)	development; electron transporter activity; glutathione peroxidase activity; mitochondrion; oxidoreductase activity; peroxidase activity; phospholipid metabolism; response to oxidative stress	[SUMMARY:] Glutathione peroxidase catalyzes the reduction of hydrogen peroxide, organic hydroperoxide, and lipid peroxides by reduced glutathione and functions in the protection of cells against oxidative damage. Human plasma glutathione peroxidase has been shown to be a selenium-containing enzyme.	25159.27	20990.36	4168.906	0.261363
207750_at	EPS15L2	55380	epidermal growth factor receptor pathway substrate 15-like 2			7916	3764.06	4151.94	1.072482

200011_s_at	ARF3	377	ADP-ribosylation factor 3		[SUMMARY:] ADP-ribosylation factor 3 (ARF3) is a member of the human ARF gene family. These genes encode small guanine nucleotide-binding proteins that stimulate the ADP-ribosyltransferase activity of cholera toxin and play a role in vesicular trafficking and as activators of phospholipase D. The gene products include 6 ARF proteins and 11 ARF-like proteins and constitute 1 family of the RAS superfamily. The ARF proteins are categorized as class I (ARF1, ARF2,and ARF3), class II (ARF4 and ARF5) and class III (ARF6) and members of each class share a common gene organization. The ARF3 gene contains five exons and four introns.	19022.97	14886.46	4136.506	0.353741
209200_at	MEF2C	4208	MADS box transcription enhancer factor 2, polypeptide C (myocyte enhancer factor 2C)	RNA polymerase II transcription factor activity; muscle development; neurogenesis; nucleus; regulation of transcription, DNA-dependent; transcription coactivator activity; transcription factor activity; transcription from Pol II promoter		20832.2	16724.32	4107.881	0.316868
200910_at	CCT3	7203	chaperonin containing TCP1, subunit 3 (gamma)	ATP binding; chaperone activity; cytoskeleton; cytosol; protein folding		21416.73	17310.94	4105.791	0.307054
200035_at	DULLARD	23399	dullard homolog (Xenopus laevis)		[Proteome FUNCTION:] Moderately similar to a region of yeast Nem1p	18977	14882.62	4094.38	0.350623
201010_s_at	TXNIP	10628	thioredoxin interacting protein	biological_process unknown; cellular_component unknown; molecular_function unknown		26123.03	22030.28	4092.754	0.245835
200634_at	PFN1	5216	profilin 1		[SUMMARY:] The protein encoded by this gene is a ubiquitous actin monomer-binding protein belonging to the profilin family. It is thought to regulate actin polymerization in response to extracellular signals. Deletion of this gene is associated with Miller-Dieker syndrome.	45793.37	41790.88	4002.488	0.131951

202588_at	AK1	203	adenylate kinase 1	ATP binding; ATP metabolism; adenylate kinase activity; cytosol; kinase activity; transferase activity	[SUMMARY:] Adenylate kinase is an enzyme involved in regulating the adenine nucleotide composition within a cell by catalyzing the reversible transfer of phosphate group among adinine nucleotides. Three isozymes of adenylate kinase have been identified in vertebrates, adenylate isozyme 1 (AK1), 2 (AK2) and 3 (AK3). AK1 is found in the cytosol of skeletal muscle, brain and erythrocytes, whereas AK2 and AK3 are found in the mitochondria of other tissues including liver and heart. AK1 was identified because of its association with a rare genetic disorder causing nonspherocytic hemolytic anemia where a mutation in the AK1 gene was found to reduce the catalytic activity of the enzyme. Alternative splicing of the AK1 gene yields two transcripts of 0.9 kb and 2.5 kb. The two transcripts differ in the 3-prime non-coding region and use two distinct polyadenylation signals.	18102.53	14120.7	3981.835	0.35838
200652_at	SSR2	6746	signal sequence receptor, beta (translocon-associated protein beta)	cotranslational membrane targeting; endoplasmic reticulum; integral to membrane; signal sequence binding	[SUMMARY:] The signal sequence receptor (SSR) is a glycosylated endoplasmic reticulum (ER) membrane receptor associated with protein translocation across the ER membrane. The SSR consists of 2 subunits, a 34-kD glycoprotein (alpha-SSR or SSR1) and a 22-kD glycoprotein (beta-SSR or SSR2). The human beta-signal sequence receptor gene (SSR2) maps to chromosome bands 1q21-q23.	21745.37	17795.7	3949.668	0.289179
211814_s_at	CCNE2	9134	cyclin E2	cell cycle checkpoint; cytokinesis; nucleus; regulation of CDK activity; regulation of cell cycle	[SUMMARY:] The protein encoded by this gene belongs to the highly conserved cyclin family, whose members are characterized by a dramatic periodicity in protein abundance through the cell cycle. Cyclins function as regulators of CDK kinases. Different cyclins exhibit distinct expression and degradation patterns which contribute to the temporal coordination of each mitotic event. This cyclin forms a complex with and functions as a regulatory subunit of CDK2. This cyclin has been shown to specifically interact with CIP/KIP family of CDK inhibitors, and plays a role in cell cycle G1/S transition. The expression of this gene peaks at the G1-S phase and exhibits a pattern of tissue specificity distinct from that of cyclin E1. A significantly increased expression level of this gene was observed in tumor-derived cells. Three alternatively spliced transcript variants, which encode distinct isoforms, have been reported.	16268.67	12331.52	3937.147	0.399745

213688_at	CALM1	801	calmodulin 1 (phosphorylase kinase, delta)	G-protein coupled receptor protein signaling pathway; calcium ion binding; cytoplasm; plasma membrane; protein binding	[SUMMARY:] Calmodulin is the archetype of the family of calcium-modulated proteins of which nearly 20 members have been found. They are identified by their occurrence in the cytosol or on membranes facing the cytosol and by a high affinity for calcium. Calmodulin contains 149 amino acids and has 4 calcium-binding domains. Its functions include roles in growth and the cell cycle as well as in signal transduction and the synthesis and release of neurotransmitters.[supplied by OMIM]	15296.77	11386.88	3909.886	0.425854
201842_s_at	EFEMP1	2202	EGF-containing fibulin-like extracellular matrix protein 1	calcium ion binding; extracellular matrix; visual perception	[SUMMARY:] This gene spans approximately 18 kb of genomic DNA and consists of 12 exons. Two transcripts with distinct 5' UTR have been described; the resulting proteins have distinct N-terminal amino acid sequences. Translation initiation from internal methionine residues was observed with in vitro translation. A signal peptide sequence is predicted for translation initiation sites 1, 2, and 4. The protein isoforms contain 5 or 6 calcium-binding EGF2 domains and 5 or 6 EGF2 domains. Mutations in this gene cause the retinal disease Malattia Leventinese.	42821.83	38922.86	3898.969	0.137729
200022_at	RPL18	6141	ribosomal protein L18		[SUMMARY:] Ribosomes, the organelles that catalyze protein synthesis, consist of a small 40S subunit and a large 60S subunit. Together these subunits are composed of 4 RNA species and approximately 80 structurally distinct proteins. This gene encodes a ribosomal protein that is a component of the 60S subunit. The protein belongs to the L18E family of ribosomal proteins. It is located in the cytoplasm. As is typical for genes encoding ribosomal proteins, there are multiple processed pseudogenes of this gene dispersed through the genome.	9782.533	13668.32	3885.787	-0.482556
203141_s_at	AP3B1	8546	adaptor-related protein complex 3, beta 1 subunit	Golgi apparatus; intracellular protein transport; protein transporter activity	[SUMMARY:] This gene encodes a protein that may play a role in organelle biogenesis associated with melanosomes, platelet dense granules, and lysosomes. The encoded protein is part of the heterotetrameric AP-3 protein complex which interacts with the scaffolding protein clathrin. Mutations in this gene are associated with Hermansky-Pudlak syndrome type 2.	15056.7	11214.86	3841.841	0.424994
201047_x_at	RAB6A	5870	RAB6A, member RAS oncogene family	GTP binding; Golgi apparatus; RAB small monomeric GTPase activity; intracellular protein transport; protein transporter activity; small GTPase mediated signal transduction		11876.2	15695.82	3819.618	-0.402307

201033_x_at	RPLP0	6175	ribosomal protein, large, P0	RNA binding; cytosolic large ribosomal subunit (sensu Eukarya); intracellular; protein biosynthesis; ribosome; structural constituent of ribosome; translational elongation	[SUMMARY:] Ribosomes, the organelles that catalyze protein synthesis, consist of a small 40S subunit and a large 60S subunit. Together these subunits are composed of 4 RNA species and approximately 80 structurally distinct proteins. This gene encodes a ribosomal protein that is a component of the 60S subunit. The protein, which is the functional equivalent of the E. coli L10 ribosomal protein, belongs to the L10P family of ribosomal proteins. It is a neutral phosphoprotein with a C-terminal end that is nearly identical to the C-terminal ends of the acidic ribosomal phosphoproteins P1 and P2. The P0 protein can interact with P1 and P2 to form a pentameric complex consisting of P1 and P2 dimers, and a P0 monomer. The protein is located in the cytoplasm. Transcript variants derived from alternative splicing exist; they encode the same protein. As is typical for genes encoding ribosomal proteins, there are multiple processed pseudogenes of this gene dispersed through the genome.	13183.3	9372.84	3810.46	0.492153
200023_s_at	EIF3S5	8665	eukaryotic translation initiation factor 3, subunit 5 epsilon, 47kDa	eukaryotic translation initiation factor 3 complex; protein biosynthesis; regulation of translational initiation; translation initiation factor activity		14555.83	10753.68	3802.153	0.436767
200651_at	GNB2L1	10399	guanine nucleotide binding protein (G protein), beta polypeptide 2-like 1			26330.77	30119.76	3788.994	-0.193961
211943_x_at	TPT1	7178	tumor protein, translationally-controlled 1	cytoplasm; extracellular space; molecular_function unknown		27677.2	23908.38	3768.82	0.211182
200026_at	RPL34	6164	ribosomal protein L34		[SUMMARY:] Ribosomes, the organelles that catalyze protein synthesis, consist of a small 40S subunit and a large 60S subunit. Together these subunits are composed of 4 RNA species and approximately 80 structurally distinct proteins. This gene encodes a ribosomal protein that is a component of the 60S subunit. The protein belongs to the L34E family of ribosomal proteins. It is located in the cytoplasm. This gene originally was thought to be located at 17q21, but it has been mapped to 4q. Transcript variants derived from alternative splicing, alternative transcription initiation sites, and/or alternative polyadenylation exist; these variants encode the same protein. As is typical for genes encoding ribosomal proteins, there are multiple processed pseudogenes of this gene dispersed through the genome.	35084.77	31322.88	3761.885	0.163628

200823_x_at	RPL29	6159	ribosomal protein L29	GTPase activity; small GTPase mediated signal transduction	[SUMMARY:] Ribosomes, the organelles that catalyze protein synthesis, consist of a small 40S subunit and a large 60S subunit. Together these subunits are composed of 4 RNA species and approximately 80 structurally distinct proteins. This gene encodes a cytoplasmic ribosomal protein that is a component of the 60S subunit. The protein belongs to the L29E family of ribosomal proteins. The protein is also a peripheral membrane protein expressed on the cell surface that directly binds heparin. Although this gene was previously reported to map to 3q29-qter, it is believed that it is located at 3p21.3-p21.2. As is typical for genes encoding ribosomal proteins, there are multiple processed pseudogenes of this gene dispersed through the genome.	19916.63	23665.88	3749.246	-0.248835
212362_at	ATP2A2	488	ATPase, Ca++ transporting, cardiac muscle, slow twitch 2		[SUMMARY:] This gene encodes one of the SERCA Ca(2+)-ATPases, which are intracellular pumps located in the sarcoplasmic or endoplasmic reticula of muscle cells. This enzyme catalyzes the hydrolysis of ATP coupled with the translocation of calcium from the cytosol to the sarcoplasmic reticulum lumen, and is involved in regulation of the contraction/relaxation cycle. Mutations in this gene cause Darier-White disease, also known as keratosis follicularis, an autosomal dominant skin disorder characterized by loss of adhesion between epidermal cells and abnormal keratinization. Alternative splicing results in two transcript variants encoding different isoforms.	39270.33	35525.86	3744.469	0.14457
200782_at	ANXA5	308	annexin A5	blood coagulation; calcium ion binding; calcium-dependent phospholipid binding; negative regulation of coagulation; phospholipase inhibitor activity	[SUMMARY:] The protein encoded by this gene belongs to the annexin family of calcium-dependent phospholipid binding proteins some of which have been implicated in membrane-related events along exocytotic and endocytotic pathways. Annexin 5 is a phospholipase A2 and protein kinase C inhibitory protein with calcium channel activity and a potential role in cellular signal transduction, inflammation, growth and differentiation. Annexin 5 has also been described as placental anticoagulant protein I, vascular anticoagulant-alpha, endonexin II, lipocortin V, placental protein 4 and anchorin CII. The gene spans 29 kb containing 13 exons, and encodes a single transcript of approximately 1.6 kb and a protein product with a molecular weight of about 35 kDa.	36492.14	32796.88	3695.258	0.154027

206769_at	TMSB4Y	9087	thymosin, beta 4, Y-linked		[SUMMARY:] This gene lies within the male specific region of chromosome Y. Its homolog on chromosome X escapes X inactivation and encodes an actin sequestering protein.	10384.3	14078.26	3693.961	-0.439065
200835_s_at	MAP4	4134	microtubule-associated protein 4		[SUMMARY:] This gene encodes a protein that belongs to the microtubule-associated protein family. The proteins of this family are thought to be involved in microtublule dynamics, which is important for cell polarization and movement. This protein was detected predominately in nonneuronal cells. Although the exact function of this protein has not been determined, there is evidence suggesting a role in microtubule stabilization as well as promoting microtubule assembly. Four alternatively spliced variants have been described.	23966	20289.72	3676.281	0.24024
210171_s_at	CREM	1390	cAMP responsive element modulator	DNA binding; cAMP response element binding protein binding; nucleus; protein binding; regulation of transcription, DNA-dependent; signal transduction; transcription factor activity	[SUMMARY:] This gene encodes a bZIP transcription factor that binds to the cAMP responsive element found in many viral and cellular promoters. It is an important component of cAMP-mediated signal transduction during the spermatogenetic cycle, as well as other complex processes. Alternative promoter and translation initiation site usage allows this gene to exert spatial and temporal specificity to cAMP responsiveness. Multiple alternatively spliced transcript variants encoding several different isoforms have been found for this gene, with some of them functioning as activators and some as repressors of transcription.	12223.8	8548.82	3674.979	0.515896
217415_at	POLR2A	5430	polymerase (RNA) II (DNA directed) polypeptide A, 220kDa	DNA binding; DNA-directed RNA polymerase II, core complex; DNA-directed RNA polymerase activity; nucleus; protein binding; regulation of transcription, DNA-dependent; transcription; transcription from Pol II promoter; transcription, DNA-dependent; transferase activity	[SUMMARY:] This gene encodes the largest subunit of RNA polymerase II, the polymerase responsible for synthesizing messenger RNA in eukaryotes. The product of this gene contains a carboxy terminal domain composed of heptapeptide repeats that are essential for polymerase activity. These repeats contain serine and threonine residues that are phosphorylated in actively transcribing RNA polymerase. In addition, this subunit, in combination with several other polymerase subunits, forms the DNA binding domain of the polymerase, a groove in which the DNA template is transcribed into RNA.	9392.601	13058.64	3666.039	-0.475408
211938_at	EIF4B	1975	eukaryotic translation initiation factor 4B	RNA binding; eukaryotic translation initiation factor 4F complex; protein biosynthesis; regulation of translational initiation; translation initiation factor activity		7788.934	4153.84	3635.094	0.90698

200030_s_at	SLC25A3	5250	solute carrier family 25 (mitochondrial carrier; phosphate carrier), member 3	binding; energy pathways; integral to plasma membrane; mitochondrial inner membrane; mitochondrion; phosphate carrier activity; symporter activity; transport	[SUMMARY:] The phosphate carrier (SLC25A3) catalyzes the transport of phosphate into the mitochondrial matrix, either by proton cotransport or in exchange for hydroxyl ions. The protein contains three related segments arranged in tandem which are related to those found in other characterized members of the mitochondrial carrier family. Both the N-terminal and C-terminal regions of the phosphate carrier protrude toward the cytosol. Two transcripts containing either exon IIIA or exon IIIB have been isolated. The variant containing exon IIIA is predominant in heart and liver, while that containing IIIB has greater expression in lung tissue.	23215.17	19600.64	3614.525	0.244167
205031_at	EFNB3	1949	ephrin-B3	cell-cell signaling; development; integral to plasma membrane; neurogenesis; transmembrane-ephrin receptor activity	[SUMMARY:] EFNB3, a member of the ephrin gene family, is important in brain development as well as in its maintenance. Moreover, since levels of EFNB3 expression were particularly high in several forebrain subregions compared to other brain subregions, it may play a pivotal role in forebrain function. The EPH and EPH-related receptors comprise the largest subfamily of receptor protein-tyrosine kinases and have been implicated in mediating developmental events, particularly in the nervous system. EPH Receptors typically have a single kinase domain and an extracellular region containing a Cys-rich domain and 2 fibronectin type III repeats. The ephrin ligands and receptors have been named by the Eph Nomenclature Committee (1997). Based on their structures and sequence relationships, ephrins are divided into the ephrin-A (EFNA) class, which are anchored to the membrane by a glycosylphosphatidylinositol linkage, and the ephrin-B (EFNB) class, which are transmembrane proteins. The Eph family of receptors are similarly divided into 2 groups based on the similarity of their extracellular d[illegible]	1135.767	4721.5	3585.733	-2.055579

205737_at	KCNQ2	3785	potassium voltage-gated channel, KQT-like subfamily, member 2	cation transport; integral to membrane; membrane fraction; neurogenesis; potassium ion transport; synaptic transmission; voltage-gated potassium channel activity; voltage-gated potassium channel complex	[SUMMARY:] The M channel is a slowly activating and deactivating potassium channel that plays a critical role in the regulation of neuronal excitability. The M channel is formed by the association of the protein encoded by this gene and a related protein encoded by the KCNQ3 gene, both integral membrane proteins. M channel currents are inhibited by M1 muscarinic acetylcholine receptors and activated by retigabine, a novel anti-convulsant drug. Defects in this gene are a cause of benign familial neonatal convulsions type 1 (BFNC), also known as epilepsy, benign neonatal type 1 (EBN1). At least five transcript variants encoding five different isoforms have been found for this gene.	12031.6	8477.24	3554.358	0.505162
214768_x_at						21695.7	18177.7	3518.002	0.25524
201853_s_at	CDC25B	994	cell division cycle 25B	M phase of mitotic cell cycle; cytokinesis; hydrolase activity; intracellular; mitosis; positive regulation of cell proliferation; protein amino acid dephosphorylation; protein-tyrosine-phosphatase activity; regulation of cell cycle	[SUMMARY:] CDC25B is a member of the CDC25 family of phosphatases. CDC25B activates the cyclin dependent kinase CDC2 by removing two phosphate groups and it is required for entry into mitosis. CDC25B shuttles between the nucleus and the cytoplasm due to nuclear localization and nuclear export signals. The protein is nuclear in the M and G1 phases of the cell cycle and moves to the cytoplasm during S and G2. CDC25B has oncogenic properties, although its role in tumor formation has not been determined. At least four transcript variants for this gene exist.	8941.399	5441.06	3500.339	0.716613
212789_at	KIAA0056	23310	KIAA0056 protein			16872.67	20364.2	3491.533	-0.271347
201666_at	TIMP1	7076	tissue inhibitor of metalloproteinase 1 (erythroid potentiating activity, collagenase inhibitor)	development; extracellular matrix; metalloendopeptidase inhibitor activity; metallopeptidase activity; positive regulation of cell proliferation; proteolysis and peptidolysis	[SUMMARY:] This gene belongs to the TIMP gene family. The proteins encoded by this gene family are natural inhibitors of the matrix metalloproteinases, a group of peptidases involved in degradation of the extracellular matrix. This gene located within intron 6 of the synapsin I gene and is transcribed in the opposite direction. TIMP1 is highly inducible at the transcriptional level in response to many cytokines and hormones. In addition, the expression from some but not all inactive X chromosomes suggests that this gene inactivation is polymorphic in human females.	23601.23	20110.7	3490.535	0.230899
AFFX-r2-P1-cre-						11026.27	7554.819	3471.447	0.545475
208696_at	CCT5	22948	chaperonin containing TCP1, subunit 5 (epsilon)	ATP binding; chaperone activity; protein folding		36859.9	33417.24	3442.66	0.14146
212043_at	TGOLN2	10618	trans-golgi network protein 2	Golgi trans face; integral to membrane; transport vesicle		19632	16191.2	3440.8	0.277997
206560_s_at	MIA	8190	melanoma inhibitory activity	cell proliferation; extracellular space; growth factor activity		28774.23	25337.08	3437.152	0.183527
221672_s_at	T1	83696	Tularik gene 1			383.9	3814.5	3430.6	-3.312692
222230_s_at	ACTR10	55860	actin-related protein 10 homolog (S. cerevisiae)			11142.3	7739.22	3403.08	0.525787

219646_at	FLJ20186	54849	hypothetical protein FLJ20186			24643.07	28036.14	3393.072	-0.186106
209019_s_at	PINK1	65018	PTEN induced putative kinase 1	ATP binding; protein amino acid phosphorylation; protein serine/threonine kinase activity; transferase activity		7638.367	10969.7	3331.333	-0.522188
208747_s_at	C1S	716	complement component 1, s subcomponent	calcium ion binding; chymotrypsin activity; complement activation, classical pathway; complement component C1s activity; hydrolase activity; proteolysis and peptidolysis; trypsin activity		9789.833	13114.98	3325.146	-0.421859
214939_x_at	MLLT4	4301	myeloid/lymphoid or mixed-lineage leukemia (trithorax homolog, Drosophila); translocated to, 4	biological_process unknown; cell adhesion; cell growth and/or maintenance; cell-cell signaling; cellular_component unknown; intercellular junction; molecular_function unknown; neuropeptide signaling pathway; protein C-terminus binding; signal transduction		13136.2	16429.14	3292.94	-0.322709
200934_at	DEK	7913	DEK oncogene (DNA binding)	DNA binding; GTP binding; RNA binding; SRP-dependent cotranslational membrane targeting; cell growth and/or maintenance; nucleus; regulation of transcription from Pol II promoter; signal recognition particle; signal transduction; specific RNA polymerase II transcription factor activity; viral genome replication		30666.93	27382.52	3284.412	0.163429
217774_s_at	HSPC152	51504	hypothetical protein HSPC152			23540.47	20273.8	3266.666	0.215526
202650_s_at	KIAA0195	9772	KIAA0195 gene product	ATP binding; hydrolase activity, acting on acid anhydrides, catalyzing transmembrane movement of substances; membrane		14022.2	10764.22	3257.98	0.381469
211601_at	CATR1	856	CATR tumorigenicity conversion 1			30688.6	27444.08	3244.518	0.161208
214280_x_at	HNRPA1	3178	heterogeneous nuclear ribonucleoprotein A1		[SUMMARY:] This gene belongs to the A/B subfamily of ubiquitously expressed heterogeneous nuclear ribonucleoproteins (hnRNPs). The hnRNPs are RNA binding proteins and they complex with heterogeneous nuclear RNA (hnRNA). These proteins are associated with pre-mRNAs in the nucleus and appear to influence pre-mRNA processing and other aspects of mRNA metabolism and transport. While all of the hnRNPs are present in the nucleus, some seem to shuttle between the nucleus and the cytoplasm. The hnRNP proteins have distinct nucleic acid binding properties. The protein encoded by this gene has two repeats of quasi-RRM domains that bind to RNAs. It is one of the most abundant core proteins of hnRNP complexes and it is localized to the nucleoplasm. This protein, along with other hnRNP proteins, is exported from the nucleus, probably bound to mRNA, and is immediately re-imported. Its M9 domain acts as both a nuclear localization and nuclear export signal. The encoded protein is involved in the packaging of pre-mRNA into hnRNP particles, transport of poly A+ mRNA from the nucleus to the cytoplasm,	11656.83	8432.36	3224.473	0.467167

201161_s_at	CSDA	8531	cold shock domain protein A	DNA binding; RNA polymerase II transcription factor activity; cytoplasm; double-stranded DNA binding; negative regulation of transcription from Pol II promoter; perinuclear space; regulation of transcription, DNA-dependent; response to cold; transcription corepressor activity; transcription factor activity		33453.1	30234.5	3218.598	0.145944
203108_at	RAI3	9052	retinoic acid induced 3	integral to plasma membrane; membrane; metabotropic glutamate, GABA-B-like receptor activity; receptor activity; signal transduction	[SUMMARY:] Retinoic acid plays a critical role in development, cellular growth, and differentiation. The specific function for this retinoic acid-induced gene has not yet been determined; however, it may play a role in embryonic development and epithelial cell differentiation. This gene is a member of the type 3 G protein-coupling receptor family, characterized by the signature 7-transmembrane domain motif. This gene may also be involved in interaction between retinoid acid and G protein signalling pathways.	42510.2	39301.56	3208.641	0.113222
211941_s_at	PBP	5037	prostatic binding protein			13175.4	9990.78	3184.621	0.399178
204019_s_at	SH3YL1	26751	SH3 domain containing, Ysc84-like 1 (S. cerevisiae)		[Proteome FUNCTION:] Highly similar to murine Sh3 domain YSC-like 1; may function in hair follicle development during the anagen phase	15359.43	18530.5	3171.067	-0.270777
200870_at	UNRIP	11171	unr-interacting protein			19842.03	16671.64	3170.395	0.251164
200749_at	RAN	5901	RAN, member RAS oncogene family	DNA metabolism; GTP binding; RAN small monomeric GTPase activity; RNA-nucleus export; chromatin; intracellular protein transport; mitotic spindle assembly; nuclear pore; nucleus; protein transporter activity; protein-nucleus export; regulation of cell cycle; signal transduction; small GTPase mediated signal transduction	[SUMMARY:] RAN (ras-related nuclear protein) is a small GTP binding protein belonging to the RAS superfamily that is essential for the translocation of RNA and proteins through the nuclear pore complex. The RAN protein is also involved in control of DNA synthesis and cell cycle progression. Nuclear localization of RAN requires the presence of regulator of chromosome condensation 1 (RCC1). Mutations in RAN disrupt DNA synthesis. Because of its many functions, it is likely that RAN interacts with several other proteins. RAN regulates formation and organization of the microtubule network independently of its role in the nucleus-cytosol exchange of macromolecules. RAN could be a key signaling molecule regulating microtubule polymerization during mitosis. RCC1 generates a high local concentration of RAN-GTP around chromatin which, in turn, induces the local nucleation of microtubules. RAN is an androgen receptor (AR) coactivator that binds differentially with different lengths of polyglutamine within the androgen receptor. Polyglutamine repeat expansion in the AR is linked to Kennedy's di	23499.77	20340.98	3158.787	0.208257
200083_at	USP22	23326	ubiquitin specific protease 22			14532.57	11373.96	3158.606	0.353555
213525_at	LOC146542	146542	similar to hypothetical protein MGC13138	intracellular; nucleic acid binding; regulation of transcription, DNA-dependent		11407.3	14562.68	3155.38	-0.352319

203981_s_at	ABCD4	5826	ATP-binding cassette, sub-family D (ALD), member 4	ATP binding; ATP-binding cassette (ABC) transporter activity; integral to membrane; membrane fraction; nucleotide binding; peroxisomal membrane; transport; transporter activity	[SUMMARY:] The protein encoded by this gene is a member of the superfamily of ATP-binding cassette (ABC) transporters. ABC proteins transport various molecules across extra- and intra-cellular membranes. ABC genes are divided into seven distinct subfamilies (ABC1, MDR/TAP, MRP, ALD, OABP, GCN20, White). This protein is a member of the ALD subfamily, which is involved in peroxisomal import of fatty acids and/or fatty acyl-CoAs in the organelle. All known peroxisomal ABC transporters are half transporters which require a partner half transporter molecule to form a functional homodimeric or heterodimeric transporter. The function of this peroxisomal membrane protein is unknown. However, it is speculated that it may function as a heterodimer for another peroxisomal ABC transporter and, therefore, may modify the adrenoleukodystrophy phenotype. It may also play a role in the process of peroxisome biogenesis. Alternative splicing of this gene by exon deletion and retained introns results in five observed products.	4327.6	7464.74	3137.14	-0.786525
AFFX-CreX-						13347.63	10224.4	3123.232	0.384568
202567_at	SNRPD3	6634	small nuclear ribonucleoprotein D3 polypeptide 18kDa	RNA splicing; nuclear mRNA splicing, via spliceosome; pre-mRNA splicing factor activity; small nuclear ribonucleoprotein complex; small nucleolar ribonucleoprotein complex; spliceosome complex	[SUMMARY:] The protein encoded by this gene belongs to the small nuclear ribonucleoprotein core protein family. It is required for pre-mRNA splicing and small nuclear ribonucleoprotein biogenesis.	16672.27	13553.66	3118.608	0.298768
39891_at						8595.866	5490.92	3104.946	0.646595
201817_at	KIAA0010	9690	ubiquitin-protein isopeptide ligase (E3)			14398.23	11298.66	3099.574	0.34974
213868_s_at	DHRS7	51635	dehydrogenase/reductase (SDR family) member 7	metabolism; oxidoreductase activity		13125.5	16214.24	3088.74	-0.304889

200709_at	FKBP1A	2280	FK506 binding protein 1A, 12kDa		[SUMMARY:] The protein encoded by this gene is a member of the immunophilin protein family, which play a role in immunoregulation and basic cellular processes involving protein folding and trafficking. This encoded protein is a cis-trans prolyl isomerase that binds the immunosuppressants FK506 and rapamycin. It interacts with several intracellular signal transduction proteins including type I TGF-beta receptor. It also interacts with multiple intracellular calcium release channels including the tetrameric skeletal muscle ryanodine receptor. In mouse, deletion of this homologous gene causes congenital heart disorder known as noncompaction of left ventricular myocardium. There is evidence of multiple alternatively spliced transcript variants for this gene, but the full length nature of some variants has not been determined.	9024.767	12074.52	3049.753	-0.420004
200913_at	PPM1G	5496	protein phosphatase 1G (formerly 2C), magnesium-dependent, gamma isoform	cell cycle arrest; hydrolase activity; magnesium ion binding; manganese ion binding; nucleus; protein amino acid dephosphorylation; protein phosphatase type 2C activity; protein serine/threonine phosphatase complex	[SUMMARY:] The protein encoded by this gene is a member of the PP2C family of Ser/Thr protein phosphatases. PP2C family members are known to be negative regulators of cell stress response pathways. This phosphatase is found to be responsible for the dephosphorylation of Pre-mRNA splicing factors, which is important for the formation of functional spliceosome. Studies of a similar gene in mice suggested a role of this phosphatase in regulating cell cycle progression. Alternatively spliced transcript variants encoding the same protein have been described.	26152.47	23116.02	3036.449	0.178054
211746_x_at	PSMA1	5682	proteasome (prosome, macropain) subunit, alpha type, 1	RNA binding; endopeptidase activity; polysome; proteasome core complex (sensu Eukarya); ubiquitin-dependent protein catabolism	[SUMMARY:] The proteasome is a multicatalytic proteinase complex with a highly ordered ring-shaped 20S core structure. The core structure is composed of 4 rings of 28 non-identical subunits; 2 rings are composed of 7 alpha subunits and 2 rings are composed of 7 beta subunits. Proteasomes are distributed throughout eukaryotic cells at a high concentration and cleave peptides in an ATP/ubiquitin-dependent process in a non-lysosomal pathway. An essential function of a modified proteasome, the immunoproteasome, is the processing of class I MHC peptides. This gene encodes a member of the peptidase T1A family, that is a 20S core alpha subunit. Two alternative transcripts encoding different isoforms have been identified.	16036.7	19014.72	2978.019	-0.245739
218575_at	ANAPC1	64682	anaphase promoting complex subunit 1			12680.4	9715.14	2965.26	0.384294

212186_at	ACACA	31	acetyl-Coenzyme A carboxylase alpha	ATP binding; acetyl-CoA carboxylase activity; biotin binding; biotin carboxylase activity; biotin carboxylase complex; fatty acid biosynthesis; ligase activity; metabolism	[SUMMARY:] Acetyl-CoA carboxylase (ACC) is a complex multifunctional enzyme system. ACC is a biotin-containing enzyme which catalyzes the carboxylation of acetyl-CoA to malonyl-CoA, the rate-limiting step in fatty acid synthesis. There are two ACC forms, alpha and beta, encoded by two different genes. ACC-alpha is highly enriched in lipogenic tissues. The enzyme is under long term control at the transcriptional and translational levels and under short term regulation by the phosphorylation/dephosphorylation of targeted serine residues and by allosteric transformation by citrate or palmitoyl-CoA. Multiple alternatively spliced transcript variants divergent in the 5' sequence and encoding distinct isoforms have been found for this gene.	10016.5	12969.3	2952.801	-0.372722
214092_x_at	SFRS14	10147	splicing factor, arginine/serine-rich 14			7093.167	10043.5	2950.333	-0.50176
211912_at						3723.433	6664.04	2940.607	-0.839764
206462_s_at	NTRK3	4916	neurotrophic tyrosine kinase, receptor, type 3	ATP binding; integral to plasma membrane; kinase activity; neurogenesis; neurotrophin TRKC receptor activity; protein amino acid phosphorylation; receptor activity; transferase activity; transmembrane receptor protein tyrosine kinase activity; transmembrane receptor protein tyrosine kinase signaling pathway		3044.533	5945.46	2900.927	-0.965567
