# Supplementary material for: Unsupervised clustering of gene expression data points at hypoxia as possible trigger for metabolic syndrome
Source: BMC Genomics. 2006 Dec 19;7:318. doi: 10.1186/1471-2164-7-318 (PMC1770922; doi:10.1186/1471-2164-7-318)
Supplement: Additional file 4 — Supplementary Table 3. This PDF file contains the table of genes, found differentially expressed between the most and the least metabolically sound clusters in our analysis of Patti et al. data set. [file 1471-2164-7-318-S4.pdf]

AFFYID	SYMBOL	LOCUSLINK	OMIM	GENENAME	GENEONTOLOGY	SUMMARY	expr1(least	expr2 (mos	diff	logratio
X04434_at	IGF1R	3480	147370	insulin-like growth factor 1 receptor	ATP binding; anti-apoptosis; epidermal growth factor receptor activity; insulin receptor signaling pathway; insulin-like growth factor receptor activity; integral to membrane; positive regulation of cell proliferation; protein amino acid phosphorylation; protein binding; receptor activity; regulation of cell cycle; signal transduction; transferase activity	[SUMMARY:] This receptor binds insulin-like growth factor with a high affinity. It has tyrosine kinase activity. The insulin-like growth factor I receptor plays a critical role in transformation events. Cleavage of the precursor generates alpha and beta subunits. It is highly overexpressed in most malignant tissues where it functions as an anti-apoptotic agent by enhancing cell survival.	10339.54	13798.5	3458.961	-0.41634
U75370_at							19880.42	23341.57	3461.15	-0.231553
U15008_at	SNRPD2	6633	601061	small nuclear ribonucleoprotein D2 polypeptide 16.5kDa	RNA splicing; pre-mRNA splicing factor activity; small nuclear ribonucleoprotein complex; small nucleolar ribonucleoprotein complex; spliceosome assembly; spliceosome complex	[SUMMARY:] The protein encoded by this gene belongs to the small nuclear ribonucleoprotein core protein family. It is required for pre-mRNA splicing and small nuclear ribonucleoprotein biogenesis. Alternative splicing occurs at this locus and two transcript variants encoding the same protein have been identified.	11805.96	15278.93	3472.972	-0.372028
M31525_at	HLA-DOA	3111	142930	major histocompatibility complex, class II, DO alpha	MHC class II receptor activity; antigen presentation, exogenous antigen; antigen processing, exogenous antigen via MHC class II; immune response; integral to membrane; plasma membrane		32979.52	36465.84	3486.313	-0.144975
M80629_at	CDC2L5	8621	603309	cell division cycle 2-like 5 (cholinesterase-related cell division controller)	ATP binding; cytokinesis; development; positive regulation of cell proliferation; protein amino acid phosphorylation; protein serine/threonine kinase activity; regulation of mitosis; transferase activity	[SUMMARY:] The protein encoded by this gene is a member of the cyclin-dependent serine/threonine protein kinase family. Members of this family are well known for their essential roles as master switches in cell cycle control. Some of the cell cycle control kinases are able to phosphorylate proteins that are important for cell differentiation and apoptosis, thus provide connections between cell proliferation, differentiation, and apoptosis. Proteins of this family may also be involved in non-cell cycle-related functions, such as neurocytoskeleton dynamics. The exact function of this protein has not yet been determined. It has unusually large N- and C-termini and is ubiquitously expressed in many tissues. Two alternatively spliced variants are described.	7363.6	3863.7	3499.9	0.930428

D13969_at	RNF110	7703	600346	ring finger protein 110		[SUMMARY:] The protein encoded by this gene contains a RING finger motif and is similar to the polycomb group (PcG) gene products. PcG gene products form complexes via protein-protein interaction and maintain the transcription repression of genes involved in embryogenesis, cell cycles, and tumorigenesis. This protein was shown to act as a negative regulator of transcription and has tumor suppressor activity. The expression of this gene was detected in various tumor cells, but is limited in neural organs in normal tissues. Knockout studies in mice suggested that this protein may negatively regulate the expression of different cytokines, chemokines, and chemokine receptors, and thus plays an important role in lymphocyte differentiation and migration, as well as in immune responses.	13723.06	17229.13	3506.073	-0.328248
M19989_cds1_at	PDGFA	5154	173430	platelet-derived growth factor alpha polypeptide	cell proliferation; cell surface receptor linked signal transduction; cell-cell signaling; extracellular space; growth factor activity; membrane; platelet-derived growth factor receptor binding; regulation of cell cycle	[SUMMARY:] The protein encoded by this gene is a member of the platelet-derived growth factor family. The four members of this family are mitogenic factors for cells of mesenchymal origin and are characterized by a motif of eight cysteines. This gene product can exist either as a homodimer or as a heterodimer with the platelet-derived growth factor beta polypeptide, where the dimers are connected by disulfide bonds. Studies using knockout mice have shown cellular defects in oligodendrocytes, alveolar smooth muscle cells, and Leydig cells in the testis; knockout mice die either as embryos or shortly after birth. Two splice variants have been identified for this gene.	30628.26	34146.43	3518.174	-0.156872
M55024_s_at	ICAM1	3383	147840	intercellular adhesion molecule 1 (CD54), human rhinovirus receptor	cell-cell adhesion; integral to plasma membrane; protein binding; transmembrane receptor activity	[SUMMARY:] ICAM1 (CD54) is typically expressed on endothelial cells and cells of the immune system. ICAM1 binds to integrins of type CD11a / CD18, or CD11b / CD18. ICAM1 is also exploited by Rhinovirus as a receptor.	139301	135756.3	3544.781	0.037187
X76732_at	NUCB2	4925	608020	nucleobindin 2	DNA binding; calcium ion binding; cytosol; extracellular space; plasma membrane		16895.94	20451.7	3555.762	-0.275544
U17894_at	FUT2	2524	182100	fucosyltransferase 2 (secretor status included)	Golgi apparatus; L-fucose catabolism; carbohydrate metabolism; galactoside 2-alpha-L-fucosyltransferase activity; integral to Golgi membrane; protein amino acid glycosylation; transferase activity, transferring glycosyl groups		8494.479	4921.066	3573.413	0.787555

D13168_at	EDNRB		1910	131244	endothelin receptor type B	G-protein signaling, coupled to IP3 second messenger (phospholipase C activating); endothelin receptor activity; integral to plasma membrane; negative regulation of adenylate cyclase activity; neurogenesis; perception of sound; rhodopsin-like receptor activity	[SUMMARY:] Endothelin receptor type B is a G protein-coupled receptor which activates a phosphatidylinoitol-calcium second messenger system. Its ligand, endothelin, consists of a family of three potent vasoactive peptides: ET1, ET2, and ET3. Studies suggest that the multigenic disorder, Hirschsprung disease type 2, is due to mutation in endothelin receptor type B gene. A splice variant, named SVR, has been described; the sequence of the ETB-SVR receptor is identical to ETRB except for the intracellular C-terminal domain. While both splice variants bind ET1, they exhibit different responses upon binding which suggests that they may be functionally distinct.	23044.54	26651.03	3606.492	-0.209766	
HG3432-HT3620_s_at	FGFR2		2263	176943	fibroblast growth factor receptor 2 (bacteria-expressed kinase, keratinocyte growth factor receptor, craniofacial dysostosis 1, Crouzon syndrome, Pfeiffer syndrome, Jackson-Weiss syndrome)	ATP binding; fibroblast growth factor receptor activity; integral to membrane; protein amino acid phosphorylation; protein-tyrosine kinase activity; receptor activity; transferase activity	[SUMMARY:] The protein encoded by this gene is a member of the fibroblast growth factor receptor family, where amino acid sequence is highly conserved between members and throughout evolution. FGFR family members differ from one another in their ligand affinities and tissue distribution. A full-length representative protein consists of an extracellular region, composed of three immunoglobulin-like domains, a single hydrophobic membrane-spanning segment and a cytoplasmic tyrosine kinase domain. The extracellular portion of the protein interacts with fibroblast growth factors, setting in motion a cascade of downstream signals, ultimately influencing mitogenesis and differentiation. This particular family member is a high-affinity receptor for acidic, basic and/or keratinocyte growth factor, depending on the isoform. Mutations in this gene are associated with many craniosynostotic syndromes and bone malformations. The genomic organization of this gene encompasses 20 exons. Alternative splicing in multiple exons, including those encoding the Ig-like domains,	10513.26	6889.767	3623.493	0.609683	
U37146_at	NCOR2		9612	600848	nuclear receptor co-repressor 2	DNA binding; nucleus; regulation of transcription, DNA-dependent; transcription corepressor activity		9068.38	12700.5	3632.12	-0.485969	

HG371-HT26388_s_at	MUC1	4582	158340	mucin 1, transmembrane	actin binding; cytoskeleton; integral to plasma membrane	[SUMMARY:] CD227 (MUC1)is a large cell surface mucin glycoprotein expressed by most glandular and ductal epithelial cells and some hematopoietic cell lineages. The MUC1 gene contains seven exons and produces several different alternatively spliced variants. The major expressed form of CD227 uses all seven exons and is a type 1 transmembrane protein with a large extracellular tandem repeat domain. The tandem repeat domain is highly O-glycosylated and alterations in glycosylation have been shown in epithelial cancer cells [PROW]	-5958.919	-9605.167	3646.248	-0.68876
M11718_at	COL5A2	1290	120190	collagen, type V, alpha 2	cell growth and/or maintenance; collagen; collagen type V; extracellular matrix structural constituent	[SUMMARY:] This gene encodes an alpha chain for one of the low abundance fibrillar collagens. Fibrillar collagen molecules are trimers that can be composed of one or more types of alpha chains. Type V collagen is found in tissues containing type I collagen and appears to regulate the assembly of heterotypic fibers composed of both type I and type V collagen. This gene product is closely related to type XI collagen and it is possible that the collagen chains of types V and XI constitute a single collagen type with tissue-specific chain combinations. Mutations in this gene are associated with Ehlers-Danlos syndrome, types I and II. Two transcripts that differ in the length of the 3'UTR due to the use of alternative polyadenylation signals have been identified for this gene.	7910.12	11564.6	3654.48	-0.547944
X16662_at	ANXA8	244	602396	annexin A8		[SUMMARY:] Annexin VIII belong to the family of Ca (2+) dependent phospholipid binding proteins (annexins), and has a high 56% identity to annexin V (vascular anticoagulant-alpha). It was initially isolated as 2.2 kb vascular anticoagulant-beta transcript from human placenta, a Ca (2+) dependent phospholipid binding protein that inhibits coagulation and phospholipase A2 activity. However, the fact that annexin VIII is neither an extracellular protein nor associated with the cell surface suggests that it may not play a role in blood coagulation in vivo and its physiological role remains unknown. It is expressed at low levels in human placenta and shows restricted expression in lung endothelia, skin, liver, and kidney. The gene is also found to be selectively overexpressed in acute myelocytic leukemia.	13306.82	16970.27	3663.449	-0.350844

U12465_at	RPL35	11224		ribosomal protein L35		[SUMMARY:] Ribosomes, the organelles that catalyze protein synthesis, consist of a small 40S subunit and a large 60S subunit. Together these subunits are composed of 4 RNA species and approximately 80 structurally distinct proteins. This gene encodes a ribosomal protein that is a component of the 60S subunit. The protein belongs to the L29P family of ribosomal proteins. It is located in the cytoplasm. As is typical for genes encoding ribosomal proteins, there are multiple processed pseudogenes of this gene dispersed through the genome.	41716.48	45380.64	3664.16	-0.12146
X93499_at	RAB7	7879	602298	RAB7, member RAS oncogene family	GTP binding; RAB small monomeric GTPase activity; endocytosis; intracellular protein transport; late endosome; protein transporter activity; small GTPase mediated signal transduction	[SUMMARY:] Members of the RAB family of RAS-related GTP-binding proteins are important regulators of vesicular transport and are located in specific intracellular compartments. RAB7 has been localized to late endosomes and shown to be important in the late endocytic pathway. In addition, it has been shown to have a fundamental role in the cellular vacuolation induced by the cytotoxin VacA of Helicobacter pylori.[supplied by OMIM]	11067.66	14747.33	3679.673	-0.414104
X91992_at	ALKBH	8846	605345	alkB, alkylation repair homolog (E. coli)	DNA dealkylation	[SUMMARY:] This gene encodes a homolog to the E. coli alkB gene product. The E. coli alkB protein is part of the adaptive response mechanism of DNA alkylation damage repair; however, its precise biochemical function is not clear.	26409.18	30092.07	3682.891	-0.188344
X60592_at	TNFRSF5	958	109535	tumor necrosis factor receptor superfamily, member 5	B-cell proliferation; antimicrobial humoral response (sensu Vertebrata); apoptosis; development; immune response; inflammatory response; integral to plasma membrane; platelet activation; protein complex assembly; signal transduction; transmembrane receptor activity	[SUMMARY:] The protein encoded by this gene is a member of the TNF-receptor superfamily. This receptor has been found to be essential in mediating a broad variety of immune and inflammatory responses including T cell-dependent immunoglobulin class switching, memory B cell development, and germinal center formation. AT-hook transcription factor AKNA is reported to coordinately regulate the expression of this receptor and its ligand, which may be important for homotypic cell interactions. Adaptor protein TNFR2 interacts with this receptor and serves as a mediator of the signal transduction. The interaction of this receptor and its ligand is found to be necessary for amyloid-beta-induced microglial activation, and thus is thought to be an early event in Alzheimer disease pathogenesis. Two alternatively spliced transcript variants of this gene encoding distinct isoforms have been reported.	7769.36	11458.5	3689.14	-0.56055

M60094_rna1_at	HIST1H1T	3010	142712	histone 1, H1t		[SUMMARY:] Histones are basic nuclear proteins that are responsible for the nucleosome structure of the chromosomal fiber in eukaryotes. Two molecules of each of the four core histones (H2A, H2B, H3, and H4) form an octamer, around which approximately 146 bp of DNA is wrapped in repeating units, called nucleosomes. The linker histone, H1, interacts with linker DNA between nucleosomes and functions in the compaction of chromatin into higher order structures. This gene is intronless and encodes a member of the histone H1 family. Transcripts from this gene lack polyA tails but instead contain a palindromic termination element. This gene is found in the large histone gene cluster on chromosome 6.	30287.84	33980.64	3692.797	-0.165974
X58234_at	TIMM44	10469	605058	translocase of inner mitochondrial membrane 44 homolog (yeast)	ATP binding; RNA binding; inner membrane; mitochondrial inner membrane presequence translocase complex; mitochondrial matrix; protein translocase activity; protein-mitochondrial targeting; regulation of transcription, DNA-dependent		6913	10610.53	3697.534	-0.618113
Z71389_at	DEFB4	1673	602215	defensin, beta 4	G-protein coupled receptor protein signaling pathway; chemotaxis; extracellular; immune response; response to pest/pathogen/parasite; xenobiotic metabolism	[SUMMARY:] Defensins form a family of microbicidal and cytotoxic peptides made by neutrophils. Members of the defensin family are highly similar in protein sequence. This gene encodes defensin, beta 4, an antibiotic peptide which is locally regulated by inflammation.	2378.86	6093.267	3714.407	-1.356946
M81758_at	SCN4A	6329	603967	sodium channel, voltage-gated, type IV, alpha	cation channel activity; cation transport; membrane fraction; muscle contraction; sodium ion transport; voltage-gated sodium channel activity; voltage-gated sodium channel complex		25560.62	29280.67	3720.045	-0.196025
S46622_at	PPP3CC	5533	114107	protein phosphatase 3 (formerly 2B), catalytic subunit, gamma isoform (calcineurin A gamma)	hydrolase activity; manganese ion binding; phosphoprotein phosphatase activity		-4366.78	-8098.467	3731.687	-0.891079
U68488_at	HTR7	3363	182137	5-hydroxytryptamine (serotonin) receptor 7 (adenylate cyclase-coupled)	G-protein signaling, coupled to cyclic nucleotide second messenger; circadian rhythm; circulation; integral to plasma membrane; melanocortin receptor activity; rhodopsin-like receptor activity; serotonin receptor activity; synaptic transmission	[SUMMARY:] The neurotransmitter, serotonin, is thought to play a role in various cognitive and behavioral functions. The serotonin receptor encoded by this gene belongs to the superfamily of G protein-coupled receptors and the gene is a candidate locus for involvement in autistic disorder and other neuropsychiatric disorders. Three splice variants have been identified which encode proteins that differ in the length of their carboxy terminal ends.	12540.26	16289.1	3748.839	-0.37734

HG2167-HT2237_at	AKAP13	11214	604686	A kinase (PRKA) anchor protein 13	Rho guanyl-nucleotide exchange factor activity; cell growth and/or maintenance; intracellular signaling cascade; kinase activity; membrane fraction; oncogenesis; signal transducer activity	[SUMMARY:] The A-kinase anchor proteins (AKAPs) are a group of structurally diverse proteins, which have the common function of binding to the regulatory subunit of protein kinase A (PKA) and confining the holoenzyme to discrete locations within the cell. This gene encodes a member of the AKAP family. Alternative splicing of this gene results in at least 3 transcript variants encoding different isoforms containing a dbl oncogene homology (DH) domain and a pleckstrin homology (PH) domain. The DH domain is associated with guanine nucleotide exchange activation for the Rho/Rac family of small GTP binding proteins, resulting in the conversion of the inactive GTPase to the active form capable of transducing signals. The PH domain has multiple functions. Therefore, these isoforms function as scaffolding proteins to coordinate a Rho signaling pathway and, in addition, function as protein kinase A-anchoring proteins.	-3013.34	-6778.8	3765.46	-1.169667
J04456_at	LGALS1	3956	150570	lectin, galactoside-binding, soluble, 1 (galectin 1)		[SUMMARY:] The galectins are a family of beta-galactoside-binding proteins implicated in modulating cell-cell and cell-matrix interactions. LGALS1 may act as an autocrine negative growth factor that regulates cell proliferation.	19700.56	23471.13	3770.576	-0.252651
X60382_rna1_at	COL10A1	1300	120110	collagen, type X, alpha 1(Schmid metaphyseal chondrodysplasia)	collagen; extracellular matrix structural constituent; skeletal development	[SUMMARY:] This gene encodes the alpha chain of type X collagen, a short chain collagen expressed by hypertrophic chondrocytes during endochondral ossification. Unlike type VIII collagen, the other short chain collagen, type X collagen is a homotrimer. Mutations in this gene are associated with Schmid type metaphyseal chondrodysplasia (SMCD) and Japanese type spondylometaphyseal dysplasia (SMD).	14655.06	18437.13	3782.073	-0.331215
AFFX-							3669.14	-141.8667	3811.007	4.692834

L04510_at	ARFD1	373	601747	ADP-ribosylation factor domain protein 1, 64kDa	GTP binding; enzyme activator activity; intracellular; small GTPase mediated signal transduction; small monomeric GTPase activity; zinc ion binding	[SUMMARY:] The protein encoded by this gene is a member of the tripartite motif (TRIM) family. The TRIM motif includes three zinc-binding domains, a RING, a B-box type 1 and a B-box type 2, and a coiled-coil region. This protein is also a member of the ADP ribosylation factor family of guanine nucleotide-binding family of proteins. Its carboxy terminus contains an ADP-ribosylation factor domain and a guanine nucleotide binding site, while the amino terminus contains a GTPase activating protein domain which acts on the guanine nucleotide binding site. The protein localizes to lysosomes and the Golgi apparatus. It plays a role in the formation of intracellular transport vesicles, their movement from one compartment to another, and phopholipase D activation. Three alternatively spliced transcript variants for this gene have been described.	4149.16	7981.4	3832.24	-0.943823
Z29572_at	TNFRSF17	608	109545	tumor necrosis factor receptor superfamily, member 17	antimicrobial humoral response (sensu Vertebrata); cell proliferation; development; immune response; integral to membrane; plasma membrane; receptor activity; signal transduction	[SUMMARY:] The protein encoded by this gene is a member of the TNF-receptor superfamily. This receptor is preferentially expressed in mature B lymphocytes, and may be important for B cell development and autoimmune response. This receptor has been shown to specifically bind to the tumor necrosis factor (ligand) superfamily, member 13b (TNFSF13B/TALL-1/BAFF), and to lead to NF-kappaB and MAPK8/JNK activation. This receptor also binds to various TRAF family members, and thus may transduce signals for cell survival and proliferation.	14902.66	11037.23	3865.428	0.433191
M37190_at	RIN2	54453		Ras and Rab interactor 2	GTPase activator activity; Rab guanyl-nucleotide exchange factor activity; endocytosis; intracellular signaling cascade; neuropeptide signaling pathway	[SUMMARY:] The RAB5 protein is a small GTPase involved in membrane trafficking in the early endocytic pathway. The protein encoded by this gene binds the GTP-bound form of the RAB5 protein preferentially over the GDP-bound form, and functions as a guanine nucleotide exchange factor for RAB5. The encoded protein is found primarily as a tetramer in the cytoplasm and does not bind other members of the RAB family.	9996.561	13896.5	3899.939	-0.475218
M31776_s_at	NPPB	4879	600295	natriuretic peptide precursor B	diuretic hormone activity; extracellular space; fluid secretion		11382	15283.43	3901.433	-0.425215

M97935_s_at	STAT1		6772	600555	signal transducer and activator of transcription 1, 91kDa	NIK-I-kappaB/NF-kappaB cascade; STAT protein dimerization; STAT protein nuclear translocation; caspase activation; cytoplasm; hematopoietin/interferon-class (D200-domain) cytokine receptor signal transducer activity; intracellular signaling cascade; nucleus; regulation of cell cycle; regulation of transcription, DNA-dependent; response to pest/pathogen/parasite; signal transducer activity; transcription factor activity; transcription from Pol II promoter; tyrosine phosphorylation of STAT protein	[SUMMARY:] The protein encoded by this gene is a member of the STAT protein family. In response to cytokines and growth factors, STAT family members are phosphorylated by the receptor associated kinases, and then form homo- or heterodimers that translocate to the cell nucleus where they act as transcription activators. This protein can be activated by various ligands including interferon-alpha, interferon-gamma, EGF, PDGF and IL6. This protein mediates the expression of a variety of genes, which is thought to be important for cell viability in response to different cell stimuli and pathogens. Two alternatively spliced transcript variants encoding distinct isoforms have been described.	25884.32	21979.07	3905.25	0.235948
HG620-HT620_at	PTPRE		5791	600926	protein tyrosine phosphatase, receptor type, E	cytoplasm; hydrolase activity; integral to plasma membrane; protein amino acid dephosphorylation; protein-tyrosine-phosphatase activity; soluble fraction; transmembrane receptor protein tyrosine phosphatase activity	[SUMMARY:] The protein encoded by this gene is a member of the protein tyrosine phosphatase (PTP) family. PTPs are known to be signaling molecules that regulate a variety of cellular processes including cell growth, differentiation, mitotic cycle, and oncogenic transformation. Two alternatively spliced transcript variants of this gene have been reported, one of which encodes a receptor-type PTP that possesses a short extracellular domain, a single transmembrane region, and two tandem intracytoplasmic catalytic domains; Another one encodes a PTP that contains a distinct hydrophilic N-terminus, and thus represents a nonreceptor-type isoform of this PTP. Studies of the similar gene in mice suggested the regulatory roles of this PTP in RAS related signal transduction pathways, cytokines induced SATA signaling, as well as the activation of voltage-gated K+ channels.	32061.02	35983.37	3922.346	-0.16651
J05125_at	PNLIP		5406	246600	pancreatic lipase	hydrolase activity; lipid catabolism; triacylglycerol lipase activity; triacylglycerol metabolism		6301.38	10294.97	3993.586	-0.708199
AFFX-HUMTFRR/M11507_5_at	TFRC		7037	190010	transferrin receptor (p90, CD71)	endocytosis; endosome; extracellular; integral to plasma membrane; iron ion homeostasis; iron ion transport; peptidase activity; proteolysis and peptidolysis; receptor activity; transferrin receptor activity		26712.58	22691.83	4020.748	0.235346

U14969_at	RPL28	6158	603638	ribosomal protein L28		[SUMMARY:] Ribosomes, the organelles that catalyze protein synthesis, consist of a small 40S subunit and a large 60S subunit. Together these subunits are composed of 4 RNA species and approximately 80 structurally distinct proteins. This gene encodes a ribosomal protein that is a component of the 60S subunit. The protein belongs to the L28E family of ribosomal proteins. It is located in the cytoplasm. Variable expression of this gene in colorectal cancers compared to adjacent normal tissues has been observed, although no correlation between the level of expression and the severity of the disease has been found. As is typical for genes encoding ribosomal proteins, there are multiple processed pseudogenes of this gene dispersed through the genome.	25375.92	21348.83	4027.086	0.249303
L40380_at	TRIP11	9321	604505	thyroid hormone receptor interactor 11	Golgi apparatus; protein binding; transcription coactivator activity; transcription from Pol II promoter	[SUMMARY:] TRIP11 was first identified through its ability to interact functionally with thyroid hormone receptor-beta (THRB; MIM 190160). It has also been found in association with the Golgi apparatus and microtubules.[supplied by OMIM]	10686.9	14715.8	4028.899	-0.461522
L21954_at	BZRP	706	109610	benzodiazapine receptor (peripheral)	benzodiazepine receptor activity; integral to membrane; mitochondrial outer membrane; mitochondrion; protein-mitochondrial targeting; receptor activity; signal transduction	[SUMMARY:] Present mainly in the mitochondrial compartment of peripheral tissues, PBR interacts with some benzodiazepines and has different affinities than its endogenous counterpart. PBR appears to be a key factor in the flow of cholesterol into mitochondria to permit the initiation of steroid hormone synthesis. It is speculated that patients with congenital lipoid adrenal hyperplasia, who cannot make any steroids, might have a genetic lesion in BZRP. A short form, PBR-S is also expressed in the same tissues, but at a level about ten times that of PBR.	14308.12	18338.53	4030.414	-0.358044
U35835_s_at	PRKDC	5591	600899	protein kinase, DNA-activated, catalytic polypeptide	DNA recombination; double-strand break repair; inositol/phosphatidylinositol kinase activity; nucleus; protein modification; protein serine/threonine kinase activity; transferase activity		10678.72	14728.4	4049.681	-0.463862
X57025_at	IGF1	3479	147440	insulin-like growth factor 1 (somatomedin C)	DNA replication; RAS protein signal transduction; cell motility; extracellular; glycolate metabolism; growth factor activity; hormone activity; insulin-like growth factor receptor binding; muscle development; physiological process; positive regulation of cell proliferation; signal transduction; skeletal development		24813.12	28863.33	4050.215	-0.218135

L06139_at	TEK	7010	600221	TEK tyrosine kinase, endothelial (venous malformations, multiple cutaneous and mucosal)		[SUMMARY:] The TEK receptor tyrosine kinase is expressed almost exclusively in endothelial cells in mice, rats, and humans. This receptor possesses a unique extracellular domain containing 2 immunoglobulin-like loops separated by 3 epidermal growth factor-like repeats that are connected to 3 fibronectin type III-like repeats. The ligand for the receptor is angiopoietin-1. Defects in TEK are associated with inherited venous malformations; the TEK signaling pathway appears to be critical for endothelial cell-smooth muscle cell communication in venous morphogenesis. TEK is closely related to the TIE receptor tyrosine kinase.	20815.78	24883.6	4067.818	-0.257517
X56654_at	DSG1	1828	125670	desmoglein 1	calcium ion binding; cell adhesion; cytoskeleton; homophilic cell adhesion; integral to membrane; intercellular junction; protein binding	[SUMMARY:] Desmosomes are cell-cell junctions between epithelial, myocardial and certain other cell types. Desmoglein 1 is a calcium-binding transmembrane glycoprotein component of desmosomes in vertebrate epithelial cells. Currently, three desmoglein subfamily members have been identified and all are members of the cadherin cell adhesion molecule superfamily. These desmoglein gene family members are located in a cluster on chromosome 18. The protein encoded by this gene has been identified as the autoantigen of the autoimmune skin blistering disease pemphigus foliaceus.	47336.82	51422.11	4085.285	-0.119426
U94592_at	UCP2	7351	601693	uncoupling protein 2 (mitochondrial, proton carrier)	binding; integral to membrane; membrane fraction; mitochondrial inner membrane; mitochondrial transport; mitochondrion; proton transport; transport; transporter activity; uncoupling protein activity	[SUMMARY:] Mitochondrial uncoupling proteins (UCP) are members of the larger family of mitochondrial anion carrier proteins (MACP). UCPs separate oxidative phosphorylation from ATP synthesis with energy dissipated as heat, also referred to as the mitochondrial proton leak. UCPs facilitate the transfer of anions from the inner to the outer mitochondrial membrane and the return transfer of protons from the outer to the inner mitochondrial membrane. They also reduce the mitochondrial membrane potential in mammalian cells. Tissue specificity occurs for the different UCPs and the exact methods of how UCPs transfer H+/OH- are not known. UCPs contain the three homologous protein domains of MACPs. This gene is expressed in many tissues, with the greatest expression in skeletal muscle. It is thought to play a role in nonshivering thermogenesis, obesity and diabetes. Chromosomal order is 5'-UCP3-UCP2-3'.	10569.62	14661.63	4092.013	-0.472122

X04297_at	ATP1A1	476	182310	ATPase, Na+/K+ transporting, alpha 1 polypeptide	ATP binding; ATP hydrolysis coupled proton transport; hydrogen ion homeostasis; hydrolase activity; hydrolase activity, acting on acid anhydrides, catalyzing transmembrane movement of substances; magnesium ion binding; membrane fraction; metabolism; monovalent inorganic cation transporter activity; potassium ion transport; sodium ion transport; sodium/potassium-exchanging ATPase activity; sodium/potassium-exchanging ATPase complex; sperm motility		10798.5	6676.633	4121.866	0.693638
U49928_at	MAP3K7IP1	10454	602615	mitogen-activated protein kinase kinase kinase 7 interacting protein 1	activation of MAPKKK; catalytic activity; enzyme activator activity; protein binding; transforming growth factor beta receptor, cytoplasmic mediator activity	[SUMMARY:] The protein encoded by this gene is responsible for the activation of TAK1 kinase activity. The C-terminal 68 amino acids of TAB1 are sufficient for binding and activation of TAK1 in mammalian cells, while the N-terminal 418 amino acids act as a dominant-negative inhibitor of transforming growth factor-beta-induced gene expression. Its role as a mitogen-activated protein kinase kinase kinase may play a significant role in the mediation of TGF-beta receptors and TAK1.	20949.48	25101.17	4151.688	-0.26084
AFFX-							4659.38	457.8334	4201.546	3.347243
J05096_rna1_at	ATP1A2	477	182340	ATPase, Na+/K+ transporting, alpha 2 (+) polypeptide	ATP binding; ATP hydrolysis coupled proton transport; hydrogen ion homeostasis; hydrolase activity; hydrolase activity, acting on acid anhydrides, catalyzing transmembrane movement of substances; magnesium ion binding; metabolism; monovalent inorganic cation transporter activity; potassium ion transport; sodium ion transport; sodium/potassium-exchanging ATPase activity; sodium/potassium-exchanging ATPase complex; sperm motility		44844.64	40638.13	4206.512	0.142102
D10656_at	CRK	1398	164762	v-crk sarcoma virus CT10 oncogene homolog (avian)	SH3/SH2 adaptor protein activity; actin cytoskeleton organization and biogenesis; cell growth and/or maintenance; cell motility; cytoplasm; intracellular signaling cascade; nucleus; regulation of transcription from Pol II promoter	[SUMMARY:] This gene encodes a member of an adapter protein family that binds to several tyrosine-phosphorylated proteins. The product of this gene has several SH2 and SH3 domains (src-homology domains) and is involved in several signaling pathways, recruiting cytoplasmic proteins in the vicinity of tyrosine kinase through SH2-phosphotyrosine interaction. The N-terminal SH2 domain of this protein functions as a positive regulator of transformation whereas the C-terminal SH3 domain functions as a negative regulator of transformation. Two alternative transcripts encoding different isoforms with distinct biological activity have been described.	7990.94	12241	4250.06	-0.615284

U29615_at	CHIT1	1118	600031	chitinase 1 (chitotriosidase)	carbohydrate metabolism; chitin binding; chitin catabolism; chitinase activity; extracellular space; hydrolase activity, acting on glycosyl bonds; response to bacteria; response to pest/pathogen/parasite	[SUMMARY:] Chitotriosidase is secreted by activated human macrophages and is markedly elevated in plasma of Gaucher disease patients. The expression of chitotriosidase occurs only at a late stage of differentiation of monocytes to activated macrophages in culture. Human macrophages can synthesize a functional chitotriosidase, a highly conserved enzyme with a strongly regulated expression. This enzyme may play a role in the degradation of chitin-containing pathogens.	9476.8	13751.37	4274.567	-0.537103
X16546_at	RNASE2	6036	131410	ribonuclease, RNase A family, 2 (liver, eosinophil-derived neurotoxin)	RNA catabolism; chemotaxis; endonuclease activity; extracellular; hydrolase activity; nucleic acid binding; pancreatic ribonuclease activity; ribonuclease activity		35982.92	40307.1	4324.18	-0.163722
HG2873-HT3017_at	RPL30	6156	180467	ribosomal protein L30		[SUMMARY:] Ribosomes, the organelles that catalyze protein synthesis, consist of a small 40S subunit and a large 60S subunit. Together these subunits are composed of 4 RNA species and approximately 80 structurally distinct proteins. This gene encodes a ribosomal protein that is a component of the 60S subunit. The protein belongs to the L30E family of ribosomal proteins. It is located in the cytoplasm. This gene is co-transcribed with the U72 small nucleolar RNA gene, which is located in its fourth intron. As is typical for genes encoding ribosomal proteins, there are multiple processed pseudogenes of this gene dispersed through the genome.	15588.28	19995.03	4406.752	-0.35918
U72512_at	REA	11331		repressor of estrogen receptor activity			10983.54	15407.07	4423.526	-0.488249

S57887_at	PCDHA4	56144	606310	protocadherin alpha 4	calcium ion binding; cell adhesion; homophilic cell adhesion; integral to membrane; protein binding	[SUMMARY:] This gene is a member of the protocadherin alpha gene cluster, one of three related gene clusters tandemly linked on chromosome five that demonstrate an unusual genomic organization similar to that of B-cell and T-cell receptor gene clusters. The alpha gene cluster is composed of 15 cadherin superfamily genes related to the mouse CNR genes and consists of 13 highly similar and 2 more distantly related coding sequences. The tandem array of 15 N-terminal exons, or variable exons, are followed by downstream C-terminal exons, or constant exons, which are shared by all genes in the cluster. The large, uninterrupted N-terminal exons each encode six cadherin ectodomains while the C-terminal exons encode the cytoplasmic domain. These neural cadherin-like cell adhesion proteins are integral plasma membrane proteins that most likely play a critical role in the establishment and function of specific cell-cell connections in the brain. Alternative splicing has been observed and additional variants have been suggested but their full-length nature has yet to be determined.	13271.92	8839.134	4432.786	0.5864
V00574_s_at	HRAS	3265	190020	v-Ha-ras Harvey rat sarcoma viral oncogene homolog	GTP binding; RAS small monomeric GTPase activity; cell growth and/or maintenance; cell motility; cell surface receptor linked signal transduction; chemotaxis; cytoplasm; organogenesis; plasma membrane; regulation of cell cycle; small GTPase mediated signal transduction; small monomeric GTPase activity		3827.86	8286.434	4458.574	-1.114213
J03068_at	APEH	327	102645	N-acylaminoacyl-peptide hydrolase		[SUMMARY:] This gene encodes the enzyme acylpeptide hydrolase, which catalyzes the hydrolysis of the terminal acetylated amino acid preferentially from small acetylated peptides. The acetyl amino acid formed by this hydrolase is further processed to acetate and a free amino acid by an aminoacylase. This gene is located within the same region of chromosome 3 (3p21) as the aminoacylase gene, and deletions at this locus are also associated with a decrease in aminoacylase activity. The acylpeptide hydrolase is a homotetrameric protein of 300 kDa with each subunit consisting of 732 amino acid residues. It can play an important role in destroying oxidatively damaged proteins in living cells. Deletions of this gene locus are found in various types of carcinomas, including small cell lung carcinoma and renal cell carcinoma.	10522.24	6051.167	4471.074	0.798157

U80073_at	NXF1	10482	602647	nuclear RNA export factor 1	RNA binding; mRNA processing; mRNA-nucleus export; nucleus; protein transporter activity; protein-nucleus import; transport	[SUMMARY:] This gene is one member of a family of nuclear RNA export factor genes. Common domain features of this family are a noncanonical RNP-type RNA-binding domain (RBD), 4 leucine-rich repeats (LRRs), a nuclear transport factor 2 (NTF2)-like domain that allows heterodimerization with NTF2-related export protein-1 (NXT1), and a ubiquitin-associated domain that mediates interactions with nucleoporins. The LRRs and NTF2-like domains are required for export activity. Alternative splicing seems to be a common mechanism in this gene family. The encoded protein of this gene shuttles between the nucleus and the cytoplasm and binds in vivo to poly(A)+ RNA. It is the vertebrate homologue of the yeast protein Mex67p. The encoded protein overcomes the mRNA export block caused by the presence of saturating amounts of CTE (constitutive transport element) RNA of type D retroviruses.	5961.94	10437.73	4475.793	-0.807955
M98776_rna1_at	KRT1	3848	139350	keratin 1 (epidermolytic hyperkeratosis)		[SUMMARY:] The protein encoded by this gene is a member of the keratin gene family. The type II cytokeratins consist of basic or neutral proteins which are arranged in pairs of heterotypic keratin chains coexpressed during differentiation of simple and stratified epithelial tissues. This type II cytokeratin is specifically expressed in the spinous and granular layers of the epidermis with family member KRT10 and mutations in these genes have been associated with bullous congenital ichthyosiform erythroderma. The type II cytokeratins are clustered in a region of chromosome 12q12-q13.	9706.2	5207.6	4498.6	0.898288
L09190_rna1_at	THH	7062	190370	trichohyalin	biological_process unknown; calcium ion binding; cytoskeleton		13491.32	17993.23	4501.912	-0.415423
X90976_s_at	RUNX1	861	151385	runt-related transcription factor 1 (acute myeloid leukemia 1; aml1 oncogene)	ATP binding; cell growth and/or maintenance; development; nucleus; regulation of transcription, DNA-dependent; transcription factor activity		13799.28	9197.667	4601.612	0.585253
M37075_at	MYL4	4635	160770	myosin, light polypeptide 4, alkali; atrial, embryonic	calcium ion binding; muscle development; muscle myosin; myosin; structural constituent of muscle	[SUMMARY:] Myosin is a hexameric ATPase cellular motor protein. It is composed of two myosin heavy chains, two nonphosphorylatable myosin alkali light chains, and two phosphorylatable myosin regulatory light chains. This gene encodes a myosin alkali light chain that is found in embryonic muscle and adult atria.	38600.04	43276.34	4676.297	-0.164976

X54326_at	EPRS	2058	138295	glutamyl-prolyl-tRNA synthetase		[SUMMARY:] Aminoacyl-tRNA synthetases are a class of enzymes that charge tRNAs with their cognate amino acids. In humans, the glutamyl-tRNA synthetase (GluRS) and prolyl-tRNA synthetase (ProRS) activities are contained within a single polypeptide chain, even though these enzymes belong to different classes and are thought to have evolved along independent evolutionary pathways. Glutamyl-prolyl-tRNA synthetase is made up of 1,440 amino acids encoded by 29 exons. The exons encoding the glutamyl-specific and prolyl-specific parts of the enzyme are clustered at opposite ends of the gene, separated by a long intervening DNA section with a number of exons which encode functions that may be involved in the organization of the mammalian multienzyme synthetase complex.	23997.96	28688.3	4690.34	-0.257551
M17754_at	POLR3D	661	187280	polymerase (RNA) III (DNA directed) polypeptide D, 44kDa		[SUMMARY:] This gene complements a temperature-sensitive mutant isolated from the BHK-21 Syrian hamster cell line. It leads to a block in progression through the G1 phase of the cell cycle at nonpermissive temperatures.	26876.34	22161.4	4714.939	0.278288
J04543_at	ANXA7	310	186360	annexin A7	calcium ion binding; calcium-dependent phospholipid binding; negative regulation of coagulation; voltage-gated calcium channel activity	[SUMMARY:] Annexin VII is a member of the annexin family of calcium-dependent phospholipid binding proteins. The Annexin VII gene contains 14 exons and spans approximately 34 kb of DNA. An alternatively spliced cassette exon results in two mRNA transcripts of 2.0 and 2.4 kb which are predicted to generate two protein isoforms differing in their N-terminal domain. The alternative splicing event is tissue specific and the mRNA containing the cassette exon is prevalent in brain, heart and skeletal muscle. The transcripts also differ in their 3'-non coding regions by the use of two alternative poly(A) signals. The selection of poly(A) signals is independent of the mRNA splicing pattern. ~Annexin VII encodes a protein with a molecular weight of approximately 51 kDa with a unique, highly hydrophobic N-terminal domain of 167 amino acids and a conserved C-terminal region of 299 amino acids. The latter domain is composed of alternating hydrophobic and hydrophilic segments. Structural analysis of the protein suggests that Annexin VII is a membrane binding protein with diverse prope	15044.36	19791.93	4747.572	-0.39569
X80907_at	PIK3R2	5296	603157	phosphoinositide-3-kinase, regulatory subunit, polypeptide 2 (p85 beta)	intracellular signaling cascade; phosphatidylinositol 3-kinase activity; phosphoinositide 3-kinase complex		21148.96	16350.1	4798.862	0.371287

X55733_at	EIF4B	1975	603928	eukaryotic translation initiation factor 4B	RNA binding; eukaryotic translation initiation factor 4F complex; protein biosynthesis; regulation of translational initiation; translation initiation factor activity		44812.02	49629.9	4817.883	-0.147324
X90761_at	KRTHA2	3882	602760	keratin, hair, acidic, 2	epidermal differentiation; intermediate filament	[SUMMARY:] The protein encoded by this gene is a member of the keratin gene family. As a type I hair keratin, it is an acidic protein which heterodimerizes with type II keratins to form hair and nails. The type I hair keratins are clustered in a region of chromosome 17q12-q21 and have the same direction of transcription.	27863.98	32688	4824.02	-0.23036
U67191_at	EXTL1	2134	601738	exostoses (multiple)-like 1	cell growth and/or maintenance; endoplasmic reticulum; glucuronosyl-N-acetylglucosaminyl proteoglycan 4-alpha-N-acetylglucosaminyltransferase activity; integral to membrane; skeletal development; transferase activity, transferring glycosyl groups		44415.46	49255.67	4840.207	-0.149228
L25286_s_at	COL15A1	1306	120325	collagen, type XV, alpha 1		[SUMMARY:] This gene encodes the alpha chain of type XV collagen, a member of the FACIT collagen family (fibril-associated collagens with interrupted helices). Type XV collagen has a wide tissue distribution but the strongest expression is localized to basement membrane zones so it may function to adhere basement membranes to underlying connective tissue stroma. Mouse studies have shown that collagen XV deficiency is associated with muscle and microvessel deterioration.	6638.44	11507	4868.56	-0.793596
U46752_at	OSIL	54211		oxidative stress induced like	biological_process unknown; cytosol; molecular_function unknown; zinc ion binding		19979.68	24862.83	4883.156	-0.315457
X12451_at	CTSL	1514	116880	cathepsin L	cathepsin L activity; hydrolase activity; lysosome; proteolysis and peptidolysis	[SUMMARY:] The protein encoded by this gene is a lysosomal cysteine proteinase that plays a major role in intracellular protein catabolism. Its substrates include collagen and elastin, as well as alpha-1 protease inhibitor, a major controlling element of neutrophil elastase activity. The encoded protein has been implicated in several pathologic processes, including myofibril necrosis in myopathies and in myocardial ischemia, and in the renal tubular response to proteinuria. This protein, which is a member of the peptidase C1 family, is a dimer composed of disulfide-linked heavy and light chains, both produced from a single protein precursor. At least two transcript variants encoding the same protein have been found for this gene.	10309.92	15228.17	4918.247	-0.562709
L15309_at	ZNF141	7700	194648	zinc finger protein 141 (clone pHZ-44)	DNA binding; morphogenesis; nucleus; regulation of transcription, DNA-dependent; specific RNA polymerase II transcription factor activity		-2750.04	-7697.233	4947.193	-1.484887

L19593_at	IL8RB	3579	146928	interleukin 8 receptor, beta	G-protein signaling, coupled to IP3 second messenger (phospholipase C activating); cell motility; cell proliferation; cellular defense response; chemotaxis; cytoplasm; inflammatory response; integral to plasma membrane; interleukin-8 receptor activity; rhodopsin-like receptor activity; signal transducer activity	[SUMMARY:] The protein encoded by this gene is a member of the G-protein-coupled receptor family. This protein is a receptor for interleukin 8 (IL8). It binds to IL8 with high affinity, and transduces the signal through a G-protein activated second messenger system. This receptor also binds to chemokine (C-X-C motif) ligand 1 (CXCL1/MGSA), a protein with melanoma growth stimulating activity, and has been shown to be a major component required for serum-dependent melanoma cell growth. This receptor mediates neutrophil migration to sites of inflammation. The angiogenic effects of IL8 in intestinal microvascular endothelial cells are found to be mediated by this receptor. Knockout studies in mice suggested that this receptor controls the positioning of oligodendrocyte precursors in developing spinal cord by arresting their migration. This gene, IL8RA, a gene encoding another high affinity IL8 receptor, as well as IL8RBP, a pseudogene of IL8RB, form a gene cluster in a region mapped to chromosome 2q33-q36.	47707.4	52664.7	4957.301	-0.142623
Z14982_rna 1_at	PSMB8	5696	177046	proteasome (prosome, macropain) subunit, beta type, 8 (large multifunctional protease 7)	endopeptidase activity; immune response; proteasome core complex (sensu Eukarya); proteolysis and peptidolysis; ubiquitin-dependent protein catabolism	[SUMMARY:] The proteasome is a multicatalytic proteinase complex with a highly ordered ring-shaped 20S core structure. The core structure is composed of 4 rings of 28 non-identical subunits; 2 rings are composed of 7 alpha subunits and 2 rings are composed of 7 beta subunits. Proteasomes are distributed throughout eukaryotic cells at a high concentration and cleave peptides in an ATP/ubiquitin-dependent process in a non-lysosomal pathway. An essential function of a modified proteasome, the immunoproteasome, is the processing of class I MHC peptides. This gene encodes a member of the proteasome B-type family, also known as the T1B family, that is a 20S core beta subunit. This gene is located in the class II region of the MHC (major histocompatibility complex). Expression of this gene is induced by gamma interferon and this gene product replaces catalytic subunit 3 (proteasome beta 5 subunit) in the immunoproteasome. Proteolytic processing is required to generate a mature subunit. Two alternative transcripts encoding two isoforms have been identified; both isoforms are processed to yiel	162887.3	157926.2	4961.047	0.044623
M32598_at	PYGM	5837	232600	phosphorylase, glycogen; muscle (McArdle syndrome, glycogen storage disease type V)			32425.86	37412.33	4986.473	-0.206369

U80456_at	SIM2		6493	600892 single-minded homolog 2 (Drosophila)	development; neurogenesis; nucleus; regulation of transcription, DNA-dependent; signal transducer activity; signal transduction; transcription factor activity	[SUMMARY:] SIM1 and SIM2 genes are Drosophila single-minded (sim) gene homologs. The Drosophila sim gene encodes a transcription factor that is a master regulator of fruit fly neurogenesis. SIM2 maps within the so-called Down syndrome chromosomal region. Based on the mapping position, its potential function as transcriptional repressor and similarity to Drosophila sim, it is proposed that SIM2 may contribute to some specific Down syndrome phenotypes	7423.32	12439.13	5015.813	-0.744749
L05072_s_at	IRF1		3659	147575 interferon regulatory factor 1	immune response; negative regulation of cell cycle; nucleus; regulation of transcription, DNA-dependent; transcription factor activity; transcription from Pol II promoter	[SUMMARY:] IRF1 encodes interferon regulatory factor 1, a member of the interferon regulatory transcription factor (IRF) family. IRF1 serves as an activator of interferons alpha and beta transcription, and in mouse it has been shown to be required for double-stranded RNA induction of these genes. IRF1 also functions as a transcription activator of genes induced by interferons alpha, beta, and gamma. Further, IRF1 has been shown to play roles in regulating apoptosis and tumor-suppressoion.	78243.16	83268.96	5025.797	-0.089814
HG2887-HT3031_r_at	SOX15		6665	601297 SRY (sex determining region Y)-box 15	DNA binding; RNA polymerase II transcription factor activity; chromatin; establishment and/or maintenance of chromatin architecture; male gonad development; nucleus; regulation of transcription from Pol II promoter; regulation of transcription, DNA-dependent; transcription factor activity	[SUMMARY:] This gene encodes a member of the SOX (SRY-related HMG-box) family of transcription factors involved in the regulation of embryonic development and in the determination of the cell fate. The encoded protein may act as a transcriptional regulator after forming a protein complex with other proteins.	-44933.12	-49968.27	5035.148	-0.153233
U44848_at	NRF1		4899	600879 nuclear respiratory factor 1	energy pathways; nucleus; regulation of transcription from Pol II promoter; regulation of transcription, DNA-dependent; transcription factor activity	[SUMMARY:] Gopalakrishnan and Scarpulla (1995) [PubMed 7629110] noted that the electron transport chain and oxidative phosphorylation system rely on the functional interplay of gene products expressed from both nuclear and mitochondrial genomes. Because of the limited coding capacity of the mitochondrial chromosome, nuclear genes must provide most of the respiratory subunits and all of the gene products necessary for mitochondrial DNA transcription and replication. Nuclear respiratory factor-1 (NRF1) is a transcription factor that acts on nuclear genes encoding respiratory subunits and components of the mitochondrial transcription and replication machinery.[supplied by OMIM]	12300.08	17361.07	5060.986	-0.497188
M83181_at	HTR1A		3350	109760 5-hydroxytryptamine (serotonin) receptor 1A	G-protein coupled receptor protein signaling pathway; behavior; integral to plasma membrane; positive regulation of cell proliferation; rhodopsin-like receptor activity; serotonin receptor activity		61271.34	56209.67	5061.668	0.124394

U46116_at	PTPRG	5793	176886	protein tyrosine phosphatase, receptor type, G	carbonate dehydratase activity; hydrolase activity; integral to plasma membrane; one-carbon compound metabolism; protein amino acid dephosphorylation; transmembrane receptor protein tyrosine kinase signaling pathway; transmembrane receptor protein tyrosine phosphatase activity; zinc ion binding	[SUMMARY:] The protein encoded by this gene is a member of the protein tyrosine phosphatase (PTP) family. PTPs are known to be signaling molecules that regulate a variety of cellular processes including cell growth, differentiation, mitotic cycle, and oncogenic transformation. This PTP possesses an extracellular region, a single transmembrane region, and two tandem intracytoplasmic catalytic domains, and thus represents a receptor-type PTP. The extracellular region of this PTP contains a carbonic anhydrase-like (CAH) domain, which is also found in the extracellular region of PTPRBETA/ZETA. This gene is located in a chromosomal region that is frequently deleted in renal cell carcinoma and lung carcinoma, thus is thought to be a candidate tumor suppressor gene.	33023.4	38088.64	5065.238	-0.205872
X17254_at	GATA1	2623	305371	GATA binding protein 1 (globin transcription factor 1)	development; nucleus; regulation of transcription from Pol II promoter; regulation of transcription, DNA-dependent; transcription factor activity	[SUMMARY:] This gene encodes a protein which belongs to the GATA family of transcription factors. The protein plays an important role in erythroid development by regulating the switch of fetal hemoglobin to adult hemoglobin. Mutations in this gene have been associated with X-linked dyserythropoietic anemia and thrombocytopenia.	17509.86	22604.07	5094.209	-0.368415
M32313_at	SRD5A1	6715	184753	steroid-5-alpha-reductase, alpha polypeptide 1 (3-oxo-5 alpha-steroid delta 4-dehydrogenase alpha 1)	3-oxo-5-alpha-steroid 4-dehydrogenase activity; cell-cell signaling; electron transporter activity; integral to membrane; microsome; oxidoreductase activity; sex determination; sex differentiation	[SUMMARY:] Steroid 5-alpha-reductase (EC 1.3.99.5) catalyzes the conversion of testosterone into the more potent androgen, dihydrotestosterone (DHT). There are 2 isoforms of the enzyme: SRD5A1 and SRD5A2 (MIM 607306).[supplied by OMIM]	36268	31099.6	5168.4	0.221801
Y00083_s_at	TGFB2	7042	190220	transforming growth factor, beta 2	cell growth; cell proliferation; cell-cell signaling; extracellular space; growth; regulation of cell cycle; signal transduction; transforming growth factor beta receptor binding		10551.86	15728.8	5176.938	-0.575911
M31241_s_at	CR1	1378	120620	complement component (3b/4b) receptor 1, including Knops blood group system	complement activation, classical pathway; complement component C3b receptor activity; integral to plasma membrane; receptor activity	[SUMMARY:] This gene encodes a membrane glycoprotein found on peripheral blood cells, glomerular podocytes, and follicular dendritic cells. The protein is a receptor for complement components C3b and C4b and regulates the activity of the complement cascade. Variation in this protein is the basis of the Knops blood group system. The two most common alleles, F and S, differ by 8 exons and are thought to be the result of an unequal crossover event. A secreted form of the protein present in plasma has been described, but its full length nature has not been determined.	15233.8	10051.07	5182.733	0.599927

X73460_at	RPL3	6122	604163	ribosomal protein L3	RNA binding; cytosolic large ribosomal subunit (sensu Eukarya); intracellular; nucleolus; protein biosynthesis; ribosome; structural constituent of ribosome	[SUMMARY:] Ribosomes, the organelles that catalyze protein synthesis, consist of a small 40S subunit and a large 60S subunit. Together these subunits are composed of 4 RNA species and approximately 80 structurally distinct proteins. This gene encodes a ribosomal protein that is a component of the 60S subunit. The protein belongs to the L3P family of ribosomal proteins. It is located in the cytoplasm. The protein can bind to the HIV-1 TAR mRNA, and it has been suggested that the protein contributes to tat-mediated transactivation. This gene is co-transcribed with the small nucleolar RNA genes U43, U86, U83a, and U83b, which are located in its first, third, fifth, and seventh introns, respectively. As is typical for genes encoding ribosomal proteins, there are multiple processed pseudogenes of this gene dispersed through the genome.	21324.28	16133.33	5190.948	0.402453
X62429_s_at	POU1F1	5449	173110	POU domain, class 1, transcription factor 1 (Pit1, growth hormone factor 1)	negative regulation of cell proliferation; nucleus; organogenesis; regulation of transcription, DNA-dependent; transcription factor activity; transcription from Pol II promoter	[SUMMARY:] PIT1 is a pituitary-specific transcription factor responsible for pituitary development and hormone expression in mammals and is a member of the POU family of transcription factors that regulate mammalian development. The POU family is so named because the first 3 members identified were PIT1 and OCT1 (MIM 164175) of mammals, and Unc-86 of C. elegans (Herr et al., 1988 [PubMed 3215510]). PIT1 contains 2 protein domains, termed POU-specific and POU-homeo, which are both necessary for high affinity DNA binding on genes encoding growth hormone (GH; MIM 139250) and prolactin (PRL; MIM 176760). PIT1 is also important for regulation of the genes encoding prolactin and thyroid-stimulating hormone beta subunit (TSHB; MIM 188540) by thyrotropin-releasing hormone (TRH; MIM 257120) and cyclic AMP.[supplied by OMIM]	34470.24	29272.57	5197.67	0.235802

L07592_at	PPARD	5467	600409	peroxisome proliferative activated receptor, delta	energy pathways; lipid metabolism; nucleus; regulation of transcription from Pol II promoter; regulation of transcription, DNA-dependent; steroid hormone receptor activity; transcription; transcription factor activity	[SUMMARY:] The protein encoded by this gene is a member of the peroxisome proliferator-activated receptor (PPAR) family. PPARs are nuclear hormone receptors that bind peroxisome proliferators and control the size and number of peroxisomes produced by cells. PPARs mediate a variety of biological processes, and may be involved in the development of several chronic diseases, including diabetes, obesity, atherosclerosis, and cancer. This protein is a potent inhibitor of ligand-induced transcription activity of PPAR delta and PPAR gamma. It may function as an integrator of transcription repression and nuclear receptor signaling. The expression of this gene is found to be elevated in colorectal cancer cells. The elevated expression can be repressed by adenomatosis polyposis coli (APC), a tumor suppressor protein related to APC/beta-catenin signaling pathway. Knockout studies in mice suggested the role of this protein in myelination of the corpus callosum, lipid metabolism, and epidermal cell proliferation. Alternatively spliced transcript variants encoding distinct isoforms have been reportec	6826.159	12050.13	5223.974	-0.819903
X67318_at	CPA1	1357	114850	carboxypeptidase A1 (pancreatic)	carboxypeptidase A activity; carboxypeptidase activity; hydrolase activity; metallopeptidase activity; proteolysis and peptidolysis	[SUMMARY:] Three different forms of human pancreatic procarboxypeptidase A have been isolated. The A1 and A2 forms are monomeric proteins with different biochemical properties. Carboxypeptidase A1 is a monomeric pancreatic exopeptidase. It is involved in zymogen inhibition.	26935.84	32181.9	5246.061	-0.256722
X77383_at	CTSO	1519	600550	cathepsin O	cysteine-type endopeptidase activity; hydrolase activity; proteolysis and peptidolysis	[SUMMARY:] The protein encoded by the gene is a cysteine proteinase and a member of the papain superfamily. This proteolytic enzyme is involved in cellular protein degradation and turnover. The recombinant form of this enzyme was shown to degrade synthetic peptides typically used as substrates for cysteine proteinases and its proteolytic activity was abolished by an inhibitor of cyteine proteinase.	10912.58	16223.47	5310.886	-0.57209
U73843_at	ELF3	1999	602191	E74-like factor 3 (ets domain transcription factor, epithelial-specific)	epidermal differentiation; morphogenesis; nucleus; regulation of transcription, DNA-dependent; transcription coactivator activity; transcription factor activity; transcription from Pol II promoter		11488.88	16816.47	5327.586	-0.549636

D10667_s_at	MYH11	4629	160745	myosin, heavy polypeptide 11, smooth muscle	ATP binding; actin binding; calmodulin binding; cell growth and/or maintenance; motor activity; muscle development; muscle myosin; myosin; striated muscle contraction; striated muscle thick filament	[SUMMARY:] The protein encoded by this gene is a smooth muscle myosin belonging to the myosin heavy chain family. The gene product is a subunit of a hexameric protein that consists of two heavy chain subunits and two pairs of non-identical light chain subunits. It functions as a major contractile protein, converting chemical energy into mechanical energy through the hydrolysis of ATP. The gene encoding a human ortholog of rat NUDE1 is transcribed from the reverse strand of this gene, and its 3' end overlaps with that of the latter. The pericentric inversion of chromosome 16 [inv(16)(p13q22)] produces a chimeric transcript that encodes a protein consisting of the first 165 residues from the N terminus of core-binding factor beta in a fusion with the C-terminal portion of the smooth muscle myosin heavy chain. This chromosomal rearrangement is associated with acute myeloid leukemia of the M4Eo subtype. Alternative splicing generates isoforms that are differentially expressed, with ratios changing during muscle cell maturation. Additional splice variants have been describe	8320.02	13725.37	5405.347	-0.722186
X60188_at	MAPK3	5595	601795	mitogen-activated protein kinase 3	ATP binding; MAP kinase activity; cellular_component unknown; protein amino acid phosphorylation; protein serine/threonine kinase activity; regulation of cell cycle; transferase activity		12190.32	17606.03	5415.713	-0.530334
X70070_at	NTSR1	4923	162651	neurotensin receptor 1 (high affinity)	G-protein coupled receptor protein signaling pathway; Golgi apparatus; endoplasmic reticulum; integral to plasma membrane; neurotensin receptor activity, G-protein coupled; rhodopsin-like receptor activity; synaptic transmission	[SUMMARY:] Neurotensin receptor 1 belongs to the large superfamily of G-protein coupled receptors. NTSR1 mediates the multiple functions of neurotensin, such as hypotension, hyperglycemia, hypothermia, antinociception, and regulation of intestinal motility and secretion.	34867.3	29445.67	5421.635	0.243819
M58460_at	PMSCL1	5393	606180	polymyositis/scleroderma autoantigen 1, 75kDa	3'-5'-exoribonuclease activity; RNA binding; exonuclease activity; hydrolase activity; immune response; nuclear exosome (RNase complex); nucleolus; rRNA processing		6967.359	1487.633	5479.726	2.227593
X69398_at	CD47	961	601028	CD47 antigen (Rh-related antigen, integrin-associated signal transducer)	cell-matrix adhesion; integral to plasma membrane; integrin-mediated signaling pathway; protein binding	[SUMMARY:] This gene encodes a membrane protein, which is involved in the increase in intracellular calcium concentration that occurs upon cell adhesion to extracellular matrix. The encoded protein is also a receptor for the C-terminal cell binding domain of thrombospondin, and it may paly a role in membrane transport and signal transduction. This gene has broad tissue distribution, and is reduced in expression on Rh erythrocytes. Two alternatively spliced transcript variants encoding distinct isoforms have been found for this gene.	6608.8	1103.067	5505.733	2.582868
AFFX-BioB-							12708.06	7108.7	5599.361	0.838086

HG1067-							7550.72	13152.27	5601.546	-0.800625
M21388_at							5473.62	11111.63	5638.013	-1.021504
U60116_at	FHL3	2275	602790	four and a half LIM domains 3	muscle development		50932.45	56642.3	5709.855	-0.153295
U12471_cds							8994.061	14820.03	5825.974	-0.720504
S74720_at	NR0B1	190	300473	nuclear receptor subfamily 0, group B, member 1	nucleus; regulation of transcription, DNA-dependent; sex determination; steroid biosynthesis; steroid hormone receptor activity; transcription factor activity	[SUMMARY:] Adrenal hypoplasia protein is an orphan nuclear hormone receptor and contains a DNA-binding domain. The AHC protein acts as a dominant-negative regulator of transcription which is mediated by the retinoic acid receptor. AHC also functions as an anti-testis gene by acting antagonistically to Sry. Mutations in AHC result in both X-linked congenital adrenal hypoplasia and hypogonadotropic hypogonadism.	12965.8	18837.57	5871.767	-0.538901
Z27113_at	POLR2F	5435	604414	polymerase (RNA) II (DNA directed) polypeptide F	DNA binding; DNA-directed RNA polymerase II, core complex; DNA-directed RNA polymerase activity; nucleus; transcription from Pol II promoter; transferase activity	[SUMMARY:] This gene encodes the sixth largest subunit of RNA polymerase II, the polymerase responsible for synthesizing messenger RNA in eukaryotes, that is also shared by the other two DNA-directed RNA polymerases. In yeast, this polymerase subunit, in combination with at least two other subunits, forms a structure that stabilizes the transcribing polymerase on the DNA template.	10616.62	16551.2	5934.581	-0.640611
M17886_at	RPLP1	6176	180520	ribosomal protein, large, P1	RNA binding; cytosolic large ribosomal subunit (sensu Eukarya); intracellular; protein biosynthesis; ribosome; structural constituent of ribosome; translational elongation	[SUMMARY:] Ribosomes, the organelles that catalyze protein synthesis, consist of a small 40S subunit and a large 60S subunit. Together these subunits are composed of 4 RNA species and approximately 80 structurally distinct proteins. This gene encodes a ribosomal phosphoprotein that is a component of the 60S subunit. The protein, which is a functional equivalent of the E. coli L7/L12 ribosomal protein, belongs to the L12P family of ribosomal proteins. It plays an important role in the elongation step of protein synthesis. Unlike most ribosomal proteins, which are basic, the encoded protein is acidic. Its C-terminal end is nearly identical to the C-terminal ends of the ribosomal phosphoproteins P0 and P2. The P1 protein can interact with P0 and P2 to form a pentameric complex consisting of P1 and P2 dimers, and a P0 monomer. The protein is located in the cytoplasm. As is typical for genes encoding ribosomal proteins, there are multiple processed pseudogenes of this gene dispersed through the genome.	77963.54	83901.37	5937.828	-0.105895

D90224_at	TNFSF4	7292	603594	tumor necrosis factor (ligand) superfamily, member 4 (tax-transcriptionally activated glycoprotein 1, 34kDa)	cell-cell signaling; immune response; integral to plasma membrane; positive regulation of cell proliferation; signal transduction; tumor necrosis factor receptor binding	[SUMMARY:] The protein encoded by this gene is a cytokine that belongs to the tumor necrosis factor (TNF) ligand family. This cytokine is a ligand for receptor TNFRSF4/OX4. It is found to be involved in T cell antigen-presenting cell (APC) interactions. In surface Ig- and CD40-stimulated B cells, this cytokine along with CD70 has been shown to provide CD28-independent costimulatory signals to T cells. This protein and its receptor are reported to directly mediate adhesion of activated T cells to vascular endothelial cells.	19792.96	25773.13	5980.174	-0.380881
M35851_s_at	AR	367	313700	androgen receptor (dihydrotestosterone receptor; testicular feminization; spinal and bulbar muscular atrophy; Kennedy disease)	androgen binding; androgen receptor activity; cell proliferation; cell-cell signaling; nucleus; prostate gland development; protein dimerization activity; receptor activity; regulation of transcription, DNA-dependent; sex differentiation; signal transduction; transcription factor activity; transport	[SUMMARY:] The androgen receptor gene is more than 90 kb long and codes for a protein that has 3 major functional domains: the N-terminal domain, DNA-binding domain, and androgen-binding domain. The protein functions as a steroid-hormone activated transcription factor. Upon binding the hormone ligand, the receptor dissociates from accessory proteins, translocates into the nucleus, dimerizes, and then stimulates transcription of androgen responsive genes. This gene contains 2 polymorphic trinucleotide repeat segments that encode polyglutamine and polyglycine tracts in the N-terminal transactivation domain of its protein. Expansion of the polyglutamine tract causes spinal bulbar muscular atrophy (Kennedy disease). Mutations in this gene are also associated with complete androgen insensitivity (CAIS).	161645.5	167782.9	6137.422	-0.053763
U96113_at	WWP1	11059	602307	WW domain-containing protein 1	T-cell differentiation; central nervous system development; ligase activity; lung development; negative regulation of transcription; protein binding; protein ubiquitination; signal transduction; ubiquitin ligase complex; ubiquitin-protein ligase activity; viral entry	[SUMMARY:] WW domain-containing proteins are found in all eukaryotes and play an important role in the regulation of a wide variety of cellular functions such as protein degradation, transcription, and RNA splicing. This gene encodes a protein which contains 4 tandem WW domains and a HECT (homologous to the E6-associated protein carboxyl terminus) domain. The encoded protein belongs to a family of NEDD4-like proteins, which are E3 ubiquitin-ligase molecules and regulate key trafficking decisions, including targeting of proteins to proteosomes or lysosomes. Alternative splicing of this gene generates at least 6 transcript variants; however, the full length nature of these transcripts has not been defined.	175702.3	169506.9	6195.469	0.05179

AFFX-HSAC07/X00351_M_at	ACTB	60	102630	actin, beta	actin filament; cell motility; motor activity; structural constituent of cytoskeleton	[SUMMARY:] Beta actin is one of six different actin isoforms which have been identified. ACTB is one of the two nonmuscle cytoskeletal actins. Actins are highly conserved proteins that are involved in cell motility, structure and integrity. Alpha actins are a major constituent of the contractile apparatus.	57205.7	63528.47	6322.77	-0.151244
X00588_at	EGFR	1956	131550	epidermal growth factor receptor (erythroblastic leukemia viral (v-erb-b) oncogene homolog, avian)	ATP binding; cell proliferation; cytoskeleton; electron transport; electron transporter activity; endosome; epidermal growth factor receptor activity; epidermal growth factor receptor signaling pathway; integral to plasma membrane; protein amino acid phosphorylation; receptor activity; transferase activity		60924.52	67247.87	6323.348	-0.142466
X15187_at	TRA1	7184	191175	tumor rejection antigen (gp96) 1	ATP binding; binding; calcium ion binding; endoplasmic reticulum; heat shock protein activity; plasma membrane; protein folding; response to stress		62818.3	56459.57	6358.734	0.153967
Z14000_at	RING1	6015	602045	ring finger protein 1	chromatin modification; nucleus; regulation of transcription, DNA-dependent; transcriptional repressor activity; zinc ion binding	[SUMMARY:] This gene belongs to the RING finger family, members of which encode proteins characterized by a RING domain, a zinc-binding motif related to the zinc finger domain. The gene product can bind DNA and can act as a transcriptional repressor. It is associated with the multimeric polycomb group protein complex. The gene product interacts with the polycomb group proteins BMI1, EDR1, and CBX4, and colocalizes with these proteins in large nuclear domains. It interacts with the CBX4 protein via its glycine-rich C-terminal domain. The gene maps to the HLA class II region, where it is contiguous with the RING finger genes FABGL and HKE4.	13418.22	19818.43	6400.213	-0.56265
J04810_s_at	MSH3	4437	600887	mutS homolog 3 (E. coli)			14251.02	7831.833	6419.187	0.863643
U90902_at	TIAM1	7074	600687	T-cell lymphoma invasion and metastasis 1	Rho guanyl-nucleotide exchange factor activity; intracellular signaling cascade; protein binding; receptor signaling protein activity		29388.56	35837.1	6448.541	-0.286199
M86407_at	ACTN3	89	102574	actinin, alpha 3	actin binding; actin filament; calcium ion binding; structural constituent of muscle	[SUMMARY:] Alpha-actinin is an actin-binding protein with multiple roles in different cell types. ACTN3 expression is limited to skeletal muscle. It is localized to the Z-disc and analogous dense bodies, where it helps to anchor the myofibrillar actin filaments	14970.12	21485.57	6515.446	-0.521282

D25304_at	ARHGEF6	9459	300267	Rac/Cdc42 guanine nucleotide exchange factor (GEF) 6	GTPase activator activity; JNK cascade; Rho guanyl-nucleotide exchange factor activity; Rho interactor activity; apoptosis; intracellular	[SUMMARY:] Rho GTPases play a fundamental role in numerous cellular processes that are initiated by extracellular stimuli that work through G protein coupled receptors. The encoded protein belongs to a family of cytoplasmic proteins that activate the Ras-like family of Rho proteins by exchanging bound GDP for GTP. It may form a complex with G proteins and stimulate Rho-dependent signals. This protein is activated by PI3-kinase. Mutations in this gene can cause X-chromosomal non-specific mental retardation.	25138.48	31739.03	6600.553	-0.336361
X58022_at	CRHBP	1393	122559	corticotropin releasing hormone binding protein	learning and/or memory; pregnancy; protein binding; signal transduction; soluble fraction	[SUMMARY:] Corticotropin-releasing hormone is a potent stimulator of synthesis and secretion of preopiomelanocortin-derived peptides. Although CRH concentrations in the human peripheral circulation are normally low, they increase throughout pregnancy and fall rapidly after parturition. Maternal plasma CRH probably originates from the placenta. Human plasma contains a CRH-binding protein which inactivates CRH and which may prevent inappropriate pituitary-adrenal stimulation in pregnancy.	20156.4	13530.9	6625.499	0.57498
X66894_s_at	FANCC	2176	227645	Fanconi anemia, complementation group C	DNA repair; cytoplasm; nucleus; protein complex assembly	[SUMMARY:] The function of FANCC is unknown but defects in this gene have been associated with Fanconi anemia.	44157	50803.86	6646.863	-0.202296
D17532_at	DDX6	1656	600326	DEAD (Asp-Glu-Ala-Asp) box polypeptide 6	ATP binding; ATP-dependent helicase activity; RNA binding; RNA helicase activity; cell growth and/or maintenance; nucleus	[SUMMARY:] DEAD box proteins, characterized by the conserved motif Asp-Glu-Ala-Asp (DEAD), are putative RNA helicases. They are implicated in a number of cellular processes involving alteration of RNA secondary structure such as translation initiation, nuclear and mitochondrial splicing, and ribosome and spliceosome assembly. Based on their distribution patterns, some members of this family are believed to be involved in embryogenesis, spermatogenesis, and cellular growth and division. This gene encodes a DEAD box protein. It may contribute to the cell proliferation and carcinogenesis.	13173.34	19900.87	6727.524	-0.59521

HG2538-HT2634_at	HNRPD	3184	601324	heterogeneous nuclear ribonucleoprotein D (AU-rich element RNA binding protein 1, 37kDa)	DNA binding; RNA binding; RNA catabolism; RNA processing; chromosome, telomeric region; cytoplasm; mRNA binding; mRNA catabolism; nucleic acid binding; nucleus; regulation of transcription, DNA-dependent; ribonucleoprotein complex; telomerase-dependent telomere maintenance	[SUMMARY:] This gene belongs to the subfamily of ubiquitously expressed heterogeneous nuclear ribonucleoproteins (hnRNPs). The hnRNPs are RNA binding proteins and they complex with heterogeneous nuclear RNA (hnRNA). These proteins are associated with pre-mRNAs in the nucleus and appear to influence pre-mRNA processing and other aspects of mRNA metabolism and transport. While all of the hnRNPs are present in the nucleus, some seem to shuttle between the nucleus and the cytoplasm. The hnRNP proteins have distinct nucleic acid binding properties. The protein encoded by this gene has two repeats of quasi-RRM domains that bind to RNAs. It localizes to both the nucleus and the cytoplasm. This protein is implicated in the regulation of mRNA stability. Multiple alternatively spliced transcript variants have been described for this gene but only three have been fully described.	6041.02	12785.5	6744.48	-1.081645
D50312_at	KCNJ8	3764	600935	potassium inwardly-rectifying channel, subfamily J, member 8	ATP-activated inward rectifier potassium channel activity; integral to membrane; ion transport; membrane fraction; potassium channel activity; potassium ion transport; voltage-gated ion channel activity; voltage-gated potassium channel complex	[SUMMARY:] Potassium channels are present in most mammalian cells, where they participate in a wide range of physiologic responses. The protein encoded by this gene is an integral membrane protein and inward-rectifier type potassium channel. The encoded protein, which has a greater tendency to allow potassium to flow into a cell rather than out of a cell, is controlled by G-proteins.	14797.08	21563.1	6766.02	-0.543252

L38951_at	KPNB1	3837	602738	karyopherin (importin) beta 1	NLS-bearing substrate-nucleus import; cytoplasm; intracellular protein transport; nuclear localization sequence binding; nuclear pore; nucleus; protein transporter activity; protein-nucleus import, docking; protein-nucleus import, translocation; zinc ion binding	[SUMMARY:] Nucleocytoplasmic transport, a signal- and energy-dependent process, takes place through nuclear pore complexes embedded in the nuclear envelope. The import of proteins containing a nuclear localization signal (NLS) requires the NLS import receptor, a heterodimer of importin alpha and beta subunits also known as karyopherins. Importin alpha binds the NLS-containing cargo in the cytoplasm and importin beta docks the complex at the cytoplasmic side of the nuclear pore complex. In the presence of nucleoside triphosphates and the small GTP binding protein Ran, the complex moves into the nuclear pore complex and the importin subunits dissociate. Importin alpha enters the nucleoplasm with its passenger protein and importin beta remains at the pore. Interactions between importin beta and the FG repeats of nucleoporins are essential in translocation through the pore complex. The protein encoded by this gene is a member of the importin beta family.	38220.86	45026.94	6806.082	-0.236428
J05428_at	UGT2B7	7364	600068	UDP glycosyltransferase 2 family, polypeptide B7	glucuronosyltransferase activity; integral to membrane; lipid metabolism; membrane fraction; microsome		21618.6	28489.23	6870.635	-0.398144
M32639_at	KIAA0992	23022	608092	palladin			66827.77	73751	6923.234	-0.142215
D55696_at	LGMN	5641	602620	legumain	hydrolase activity; legumain activity; lysosome; proteolysis and peptidolysis		31957.98	38883.27	6925.285	-0.282973
X16699_at	CYP4B1	1580	124075	cytochrome P450, family 4, subfamily B, polypeptide 1	electron transport; endoplasmic reticulum; membrane; microsome; monooxygenase activity; oxygen binding; unspecific monooxygenase activity	[SUMMARY:] This gene encodes a member of the cytochrome P450 superfamily of enzymes. The cytochrome P450 proteins are monooxygenases which catalyze many reactions involved in drug metabolism and synthesis of cholesterol, steroids and other lipids. This protein localizes to the endoplasmic reticulum. In rodents, the homologous protein has been shown to metabolize certain carcinogens; however, the specific function of the human protein has not been determined.	73063.56	80028.07	6964.508	-0.131354

X91196_s_at	ATM	472	607585	ataxia telangiectasia mutated (includes complementation groups A, C and D)	DNA binding; DNA repair; inositol/phosphatidylinositol kinase activity; meiotic recombination; negative regulation of cell cycle; nucleus; protein serine/threonine kinase activity; signal transduction; transferase activity	[SUMMARY:] The protein encoded by this gene belongs to the PI3/PI4-kinase family. This protein is an important cell cycle checkpoint kinase that phosphorylates; thus, it functions as a regulator of a wide variety of downstream proteins, including tumor suppressor proteins p53 and BRCA1, checkpoint kinase CHK2, checkpoint proteins RAD17 and RAD9, and DNA repair protein NBS1. This protein and the closely related kinase ATR are thought to be master controllers of cell cycle checkpoint signaling pathways that are required for cell response to DNA damage and for genome stability. Mutations in this gene are associated with ataxia telangiectasia, an autosomal recessive disorder. At least three alternatively spliced transcript variants, which encode distinct isoforms, have been identified.	6257.6	13250.87	6993.268	-1.082406
J05252_s_at	PCSK2	5126	162151	proprotein convertase subtilisin/kexin type 2	Golgi apparatus; cell-cell signaling; extracellular space; hydrolase activity; proprotein convertase 2 activity; proteolysis and peptidolysis; subtilase activity	[SUMMARY:] The protein encoded by this gene belongs to the subtilisin-like proprotein convertase family. The members of this family are proprotein convertases that process latent precursor proteins into their biologically active products. This encoded protein is a proinsulin-processing enzyme that plays a key role in regulating insulin biosynthesis. It is also known to cleave proopiomelanocortin, proenkephalin, prodynorphin and proluteinizing-hormone-releasing hormone. The use of alternate polyadenylation sites has been found for this gene.	25366.9	32416.23	7049.334	-0.353769

M84739_at	CALR	811	109091	calreticulin	calcium ion storage activity; chaperone activity; endoplasmic reticulum lumen; heterophilic cell adhesion; nucleus; regulation of transcription, DNA-dependent; sugar binding; transcription corepressor activity	[SUMMARY:] Calreticulin is a multifunctional protein that acts as a major Ca(2+)-binding (storage) protein in the lumen of the endoplasmic reticulum. It is also found in the nucleus, suggesting that it may have a role in transcription regulation. Calreticulin binds to the synthetic peptide KLGFFKR, which is almost identical to an amino acid sequence in the DNA-binding domain of the superfamily of nuclear receptors. Calreticulin binds to antibodies in certain sera of systemic lupus and Sjogren patients which contain anti-Ro/SSA antibodies, it is highly conserved among species, and it is located in the endoplasmic and sarcoplasmic reticulum where it may bind calcium. The amino terminus of calreticulin interacts with the DNA-binding domain of the glucocorticoid receptor and prevents the receptor from binding to its specific glucocorticoid response element. Calreticulin can inhibit the binding of androgen receptor to its hormone-responsive DNA element and can inhibit androgen receptor and retinoic acid receptor transcriptional activities in vivo, as well as retinoic acid-induced neuronal diffe	35205.62	42382.73	7177.113	-0.267671
U57627_at	OCRL	4952	309000	oculocerebrorenal syndrome of Lowe	Golgi membrane; Golgi stack; Golgi vesicle; hydrolase activity; lipid metabolism; phosphoinositide 5-phosphatase activity	[SUMMARY:] Mutations linked to the disease oculocerebrorenal syndrome of Lowe led to the identification of the OCRL gene. The encoded protein is a phospatidylinositol polyphosphate 5-phosphatase that is found in golgi cisternae.	107000.3	99658.87	7341.406	0.102544
M22877_at	CYCS	54205	123970	cytochrome c, somatic		[SUMMARY:] This gene encodes cytochrome c, a component of the electron transport chain in mitochondria. The heme group of cytochrome c accepts electrons from the b-c1 complex and transfers electrons to the cytochrome oxidase complex. Cytochrome c is also involved in initiation of apoptosis. Upon release of cytochrome c to the cytoplasm, the protein binds apoptotic protease activating factor which activates the apoptotic initiator procaspase 9. Many cytochrome c pseudogenes exist, scattered throughout the human genome.	19781.5	27161.27	7379.766	-0.457399

L33243_at	PKD1	5310	601313	polycystic kidney disease 1 (autosomal dominant)	calcium-independent cell-matrix adhesion; heterophilic cell adhesion; homophilic cell adhesion; integral to plasma membrane; morphogenesis; neuropeptide signaling pathway; sugar binding	[SUMMARY:] This gene encodes a member of the polycystin protein family. Expression of this gene has been linked to the Beta-catenin/TCF pathway. The encoded glycoprotein contains a large N-terminal extracellular region, multiple transmembrane domains, and a cytoplasmic C-tail. The encoded protein may undergo cleavage at a G protein coupled receptor proteolytic site in a process that requires the receptor for egg jelly domain. This protein may function as an integral membrane protein involved in cell-cell/matrix interactions and may modulate intracellular calcium homoeostasis and other signal-transduction pathways. The encoded protein plays a role in renal tubular development. Interactions of this protein with polycystin 2 produce cation-permeable currents. Mutations in this gene have been associated with autosomal dominant polycystic kidney disease. An alternative splice variant has been described but its biological nature has not been determined. Six pseudogenes have been described and are closely linked in a known duplicated region on chromosome 16p.	27591.92	35013.57	7421.645	-0.343668
M18000_at	RPS17	6218	180472	ribosomal protein S17		[SUMMARY:] Ribosomes, the organelles that catalyze protein synthesis, consist of a small 40S subunit and a large 60S subunit. Together these subunits are composed of 4 RNA species and approximately 80 structurally distinct proteins. This gene encodes a ribosomal protein that is a component of the 40S subunit. The protein belongs to the S17E family of ribosomal proteins. It is located in the cytoplasm. As is typical for genes encoding ribosomal proteins, there are multiple processed pseudogenes of this gene dispersed through the genome.	139589.9	132047.6	7542.313	0.080137
M64788_at	RAP1GA1	5909	600278	RAP1, GTPase activating protein 1	GTPase activator activity; membrane; signal transduction		74769.84	67011.5	7758.344	0.158048
L26336_at	HSPA2	3306	140560	heat shock 70kDa protein 2	ATP binding; heat shock protein activity; male meiosis; spermatid development		39190.32	46970.6	7780.277	-0.261261
U14747_at	VSNL1	7447	600817	visinin-like 1			44324.82	52251.44	7926.617	-0.237356
L11672_r_at	ZNF91	7644	603971	zinc finger protein 91 (HPF7, HTF10)	nucleus; regulation of transcription, DNA-dependent; transcription factor activity; zinc ion binding		15645.26	23606.93	7961.672	-0.593485

J04794_at	AKR1A1	10327	103830	aldo-keto reductase family 1, member A1 (aldehyde reductase)		[SUMMARY:] This gene encodes a member of the aldo/keto reductase superfamily, which consists of more than 40 known enzymes and proteins. This member, also known as aldehyde reductase, is involved in the reduction of biogenic and xenobiotic aldehydes and is present in virtually every tissue. Alternative splicing of this gene results in two transcript variants encoding the same protein.	104675	112734.3	8059.305	-0.10701
M63483_at	MATR3	9782	604706	matrin 3	RNA binding; nuclear inner membrane; nucleus; structural molecule activity	[SUMMARY:] The protein encoded by this gene is localized in the nuclear matrix. It may play a role in transcription or may interact with other nuclear matrix proteins to form the internal fibrogranular network.	68875.77	77016.8	8141.039	-0.161177
HG3859-HT4129_at	MAGEA4	4103	300175	melanoma antigen, family A, 4	biological_process unknown; cellular_component unknown; molecular_function unknown	[SUMMARY:] This gene is a member of the MAGEA gene family. The members of this family have their entire coding sequences located in the last exon, and the encoded proteins show 50 to 80% sequence identity between each other. The promoters and first exons of the MAGEA genes show considerable variability, suggesting that the existence of this gene family enables the same function to be expressed under different transcriptional controls. The MAGEA genes are expressed at a high level in a number of tumors of various histologic types, and are silent in normal tissues with the exception of testis and placenta. The MAGEA genes are clustered on chromosome Xq28. They may be implicated in some hereditary disorders, such as dyskeratosis congenita. Multiple alternatively spliced transcript variants differing in the 5' exon have been found for this gene, however, the full length nature of different variants has not been defined.	19511.04	11360.8	8150.241	0.780226
M77235_at	SCN5A	6331	600163	sodium channel, voltage-gated, type V, alpha (long QT syndrome 3)	cation channel activity; cation transport; integral to membrane; membrane; sodium ion transport; voltage-gated sodium channel activity; voltage-gated sodium channel complex	[SUMMARY:] The protein encoded by this gene is an integral membrane protein and tetrodotoxin-resistant voltage-gated sodium channel subunit. The encoded protein is found primarily in cardiac muscle and is responsible for the initial upstroke of the action potential in an electrocardiogram. Defects in this gene are a cause of long QT syndrome type 3 (LQT3), an autosomal dominant cardiac disease. Alternative splicing results in two transcript variants encoding separate isoforms which differ by a single amino acid. Mutation nomenclature has been assigned with respect to the longer isoform.	24003.68	32215.5	8211.82	-0.424499
U08854_s_at	UGT2B15	7366	600069	UDP glycosyltransferase 2 family, polypeptide B15	glucuronosyltransferase activity; integral to membrane; microsome; steroid metabolism; xenobiotic metabolism		25787.14	34000.67	8213.529	-0.398911

U10117_at	SCYE1		9255	603605	small inducible cytokine subfamily E, member 1 (endothelial monocyte-activating)	cell-cell signaling; chemotaxis; cytokine activity; extracellular space; inflammatory response; protein biosynthesis; signal transduction; tRNA aminoacylation for protein translation; tRNA binding	[SUMMARY:] Endothelial monocyte-activating polypeptide (SCYE1) is a cytokine that is specifically induced by apoptosis. The release of SCYE1 renders the tumor-associated vasculature sensitive to tumor necrosis factor. The precursor of SCYE1 (pro-SCYE1) is identical to the p43 subunit, which is associated with the multi-tRNA synthetase complex. Therefore, pro-SCYE1 may function in binding RNA as part of the tRNA synthetase complex in normal cells and in stimulating inflammatory responses after proteolytic cleavage in tumor cells. A conflict report exists about the RNA-binding domain in C-terminal or N-terminal of pro-SCYE1.	37022.58	45280.23	8257.648	-0.290476
U14972_at	RPS10		6204	603632	ribosomal protein S10	RNA binding; cytosolic small ribosomal subunit (sensu Eukarya); protein biosynthesis; structural constituent of ribosome	[SUMMARY:] Ribosomes, the organelles that catalyze protein synthesis, consist of a small 40S subunit and a large 60S subunit. Together these subunits are composed of 4 RNA species and approximately 80 structurally distinct proteins. This gene encodes a ribosomal protein that is a component of the 40S subunit. The protein belongs to the S10E family of ribosomal proteins. It is located in the cytoplasm. Variable expression of this gene in colorectal cancers compared to adjacent normal tissues has been observed, although no correlation between the level of expression and the severity of the disease has been found. As is typical for genes encoding ribosomal proteins, there are multiple processed pseudogenes of this gene dispersed through the genome.	95684.5	87365.34	8319.164	0.131224
X99350_rna1_at	FOXJ1		2302	602291	forkhead box J1	nucleus; regulation of transcription, DNA-dependent; spermatogenesis; transcription factor activity		22343.44	14010.93	8332.508	0.673298
D14533_at	XPA		7507	278700	xeroderma pigmentosum, complementation group A	damaged DNA binding; nucleotide-excision repair; nucleus; protein binding		61125.9	69466.7	8340.797	-0.184538
X03689_s_at	EEF1A1		1915	130590	eukaryotic translation elongation factor 1 alpha 1	GTP binding; cytoplasm; eukaryotic translation elongation factor 1 complex; oncogenesis; regulation of cell shape; regulation of translation; translational elongation	[SUMMARY:] This gene encodes an isoform of the alpha subunit of the elongation factor-1 complex, which is responsible for the enzymatic delivery of aminoacyl tRNAs to the ribosome. This isoform (alpha 1) is expressed in brain, placenta, lung, liver, kidney, and pancreas, and the other isoform (alpha 2) is expressed in brain, heart and skeletal muscle. This isoform is identified as an autoantigen in 66% of patients with Felty's syndrome. This gene has been found to have multiple copies on many chromosomes, some of which, if not all, represent different pseudogenes.	125777.7	117425.9	8351.797	0.099125

Z23115_at	BCL2L1	598	600039	BCL2-like 1	anti-apoptosis; apoptotic mitochondrial changes; integral to membrane; mitochondrion; negative regulation of survival gene product activity; regulation of apoptosis	[SUMMARY:] The protein encoded by this gene belongs to the BCL-2 protein family. BCL-2 family members form hetero- or homodimers and act as anti- or pro-apoptotic regulators that are involved in a wide variety of cellular activities. The proteins encoded by this gene are located at the outer mitochondrial membrane, and have been shown to regulate outer mitochondrial membrane channel (VDAC) opening. VDAC regulates mitochondrial membrane potential, and thus controls the production of reactive oxygen species and release of cytochrome C by mitochondria, both of which are the potent inducers of cell apoptosis. Two alternatively spliced transcript variants, which encode distinct isoforms, have been reported. The longer isoform acts as an apoptotic inhibitor and the shorter form acts as an apoptotic activator.	62890.7	71284.97	8394.27	-0.180751
M19507_at	MPO	4353	606989	myeloperoxidase	anti-apoptosis; calcium ion binding; chromatin binding; defense response; lysosome; nucleus; oxidoreductase activity; peroxidase activity; response to oxidative stress	[SUMMARY:] Myeloperoxidase (MPO) is a heme protein synthesized during myeloid differentiation that constitutes the major component of neutrophil azurophilic granules. Produced as a single chain precursor, myeloperoxidase is subsequently cleaved into a light and heavy chain. The mature myeloperoxidase is a tetramer composed of 2 light chains and 2 heavy chains. This enzyme produces hypohalous acids central to the microbicidal activity of netrophils.	38160.24	46706.64	8546.398	-0.291557
U78027_rna4_at	BTK	695	300300	Bruton agammaglobulinemia tyrosine kinase	ATP binding; cytoplasm; induction of apoptosis by extracellular signals; intracellular signaling cascade; mesoderm development; protein amino acid phosphorylation; protein-tyrosine kinase activity; transferase activity	[SUMMARY:] Defects in the Bruton tyrosine kinase (BTK) gene cause Agammaglobulinemia. Agammaglobulinemia is an X-linked immunodeficiency characterized by failure to produce mature B lymphocyte cells and associated with a failure of Ig heavy chain rearrangement.	21026.68	29652.3	8625.625	-0.495923
D14811_at	MAD2L1BP	9587		MAD2L1 binding protein			57821.04	66719.7	8898.652	-0.206518
U09825_at	TRIM26	7726	600830	tripartite motif-containing 26	DNA binding; intracellular; protein binding; zinc ion binding	[SUMMARY:] The protein encoded by this gene is a member of the tripartite motif (TRIM) family. The TRIM motif includes three zinc-binding domains, a RING, a B-box type 1 and a B-box type 2, and a coiled-coil region. The protein localizes to cytoplasmic bodies. Although the function of the protein is unknown, the RING domain suggests that the protein may have DNA-binding activity. The gene localizes to the major histocompatibility complex (MHC) class I region on chromosome 6.	39481.28	48579.56	9098.281	-0.299181

X59434_at	MPST	4357	602496	mercaptopyruvate sulfurtransferase		[SUMMARY:] This gene encodes a protein which can function as a monomer or as a disulfide-linked homodimer and which catalyzes the transfer of a sulfur ion from 3-mercaptopyruvate to cyanide or other thiol compounds. It may be involved in cyanide degradation and in thiosulfate biosynthesis. The encoded cytoplasmic protein is a member of the rhodanese family but is not rhodanese itself, which is a mitochondrial protein. At least three transcript variants have been found for this gene, but the full-length nature of only one of them has been characterized.	32606.8	41706.66	9099.863	-0.355105
HG2260-HT2349_s_at	DMD	1756	300377	dystrophin (muscular dystrophy, Duchenne and Becker types)	actin binding; biological_process unknown; calcium ion binding; cellular_component unknown; cytoskeletal anchoring; cytoskeletal anchoring activity; cytoskeleton; dystrophin-associated glycoprotein complex; membrane; molecular_function unknown; muscle contraction; muscle development; structural constituent of cytoskeleton; zinc ion binding	[SUMMARY:] The dystrophin gene is the largest gene found in nature, measuring 2.4 Mb. The gene was identified through a positional cloning approach, targeted at the isolation of the gene responsible for Duchenne (DMD) and Becker (BMD) Muscular Dystrophies. DMD is a recessive, fatal, X-linked disorder occurring at a frequency of about 1 in 3,500 new-born males. BMD is a milder allelic form. In general, DMD patients carry mutations which cause premature translation termination (nonsense or frame shift mutations), while in BMD patients dystrophin is reduced either in molecular weight (derived from in-frame deletions) or in expression level. The dystrophin gene is highly complex, containing at least eight independent, tissue-specific promoters and two polyA-addition sites. Furthermore, dystrophin RNA is differentially spliced, producing a range of different transcripts, encoding a large set of protein isoforms. Dystrophin (as encoded by the Dp427 transcripts) is a large, rod-like cytoskeletal protein which is found at the inner surface of muscle fibers. Dystrophin is p	19113.08	28264.57	9151.488	-0.564434
M22638_at	LYL1	4066	151440	lymphoblastic leukemia derived sequence 1			20839.52	29991.33	9151.814	-0.525224
X78342_at	CDK10	8558	603464	cyclin-dependent kinase (CDC2-like) 10	ATP binding; cyclin-dependent protein kinase activity; kinase activity; negative regulation of cell proliferation; protein amino acid phosphorylation; protein serine/threonine kinase activity; transferase activity; traversing start control point of mitotic cell cycle	[SUMMARY:] The protein encoded by this gene belongs to the CDK subfamily of the Ser/Thr protein kinase family. The CDK subfamily members are highly similar to the gene products of S. cerevisiae cdc28, and S. pombe cdc2, and are known to be essential for cell cycle progression. This kinase has been shown to play a role in cellular proliferation. Its function is limited to cell cycle G2-M phase. At least three alternatively spliced transcript variants encoding different isoforms have been reported, two of which contain multiple non-AUG translation initiation sites.	70217.9	60763.06	9454.836	0.208644

J04990_at	CTSG	1511	116830	cathepsin G	cathepsin G activity; chymotrypsin activity; hydrolase activity; immune response; insoluble fraction; proteolysis and peptidolysis; trypsin activity	[SUMMARY:] The protein encoded by this gene, a member of the peptidase S1 protein family, is found in azurophil granules of neutrophilic polymorphonuclear leukocytes. The encoded protease has a specificity similar to that of chymotrypsin C, and may participate in the killing and digestion of engulfed pathogens, and in connective tissue remodeling at sites of inflammation. Transcript variants utilizing alternative polyadenylation signals exist for this gene.	34037.68	43691.67	9653.988	-0.360225
D30742_at	CAMK4	814	114080	calcium/calmodulin-dependent protein kinase IV	ATP binding; calcium/calmodulin-dependent protein kinase activity; calmodulin binding; protein amino acid phosphorylation; protein serine/threonine kinase activity; signal transduction; transferase activity	[SUMMARY:] The product of this gene belongs to the serine/threonine protein kinase family, and to the Ca(2+)/calmodulin-dependent protein kinase subfamily. This enzyme is a multifunctional serine/threonine protein kinase with limited tissue distribution, that has been implicated in transcriptional regulation in lymphocytes, neurons and male germ cells.	20766.64	30642.03	9875.391	-0.561244
J04988_at	HSPCB	3326	140572	heat shock 90kDa protein 1, beta	ATP binding; cytoplasm; heat shock protein activity; protein folding		19209.88	29284.17	10074.29	-0.608272
X66171_at	CMRF35	10871	606786	CMRF35 leukocyte immunoglobulin-like receptor	cellular defense response; integral to plasma membrane; transmembrane receptor activity	[SUMMARY:] The CMRF35 antigen, which was identified by reactivity with a monoclonal antibody, is present on monocytes, neutrophils, and some T and B lymphocytes (Jackson et al., 1992 [PubMed 1349532]).[supplied by OMIM]	126660.1	116500.3	10159.83	0.120629
X53331_at	MGP	4256	154870	matrix Gla protein	calcium ion binding; cartilage condensation; extracellular matrix; extracellular matrix structural constituent; ossification; structural constituent of bone		19587.82	29806.47	10218.65	-0.605669
Y00796_at	ITGAL	3683	153370	integrin, alpha L (antigen CD11A (p180), lymphocyte function-associated antigen 1; alpha polypeptide)	cell motility; cell-matrix adhesion; integral to membrane; integrin complex; integrin-mediated signaling pathway; magnesium ion binding; protein binding; receptor activity	[SUMMARY:] ITGAL encodes the integrin alpha L chain. Integrins are heterodimeric integral membrane proteins composed of an alpha chain and a beta chain. This I-domain containing alpha integrin combines with the beta 2 chain (ITGB2) to form the integrin lymphocyte function-associated antigen-1 (LFA-1), which is expressed on all leukocytes. LFA-1 plays a central role in leukocyte intercellular adhesion through interactions with its ligands, ICAMs 1-3 (intercellular adhesion molecules 1 through 3), and also functions in lymphocyte costimulatory signaling.	138421.2	128185.2	10235.95	0.110835

U90916_at	SORL1	6653	602005	sortilin-related receptor, L(DLR class) A repeats-containing	cholesterol metabolism; integral to plasma membrane; internalization receptor activity; lipid transport; lipid transporter activity; receptor mediated endocytosis; transmembrane receptor activity	[SUMMARY:] This gene encodes a protein that belongs to the families of vacuolar protein sorting 10 (VPS10) domain-containing receptor proteins, of low density lipoprotein receptor (LDLR) proteins, and of fibronectin type III repeats proteins. In addition to VPS10, LDLR and fibronectin type 3 domains, this protein also includes an epidermal growth factor precursor-like module, a single transmembrane segment and a cytoplasmic tail with features similar to endocytosis- and sorting-competent receptors. Members of the VPS10 domain-containing receptor family are large with many exons but the CDS lengths are usually less than 3700 nt; this gene is an exception to the pattern with a CDS length greater than 6600 nt. Very large introns typically separate the exons encoding the VPS10 domain; the remaining exons are separated by much smaller-sized introns. The encoded protein is mainly intracellular and localizes in the paranuclear compartment. It is synthesized as a preproprotein, and when the propeptide is still attached, no binding occurs to the VPS10 domain. This gene is strongly express	52062.48	62604.47	10541.99	-0.266022
AFFX-BioB-							17035.72	6482.434	10553.29	1.393956
HG825-HT825_at	GNA12	2768	604394	guanine nucleotide binding protein (G protein) alpha 12			51281.08	61877.47	10596.39	-0.270987
AC002115_c	ETV2	2116		ets variant gene 2			48666.66	60280.57	11613.9	-0.308759
HG511-HT511_at	MAPKAP1	79109		mitogen-activated protein kinase associated protein 1	Ras interactor activity; biological_process unknown; cellular_component unknown		56260.78	44414.9	11845.88	0.341086
AFFX-BioC-							16855.82	4937.7	11918.12	1.771336
M29474_at	RAG1	5896	179615	recombination activating gene 1	DNA binding; DNA recombination; endonuclease activity; hemocyte development; hydrolase activity; immune response; nucleus	[SUMMARY:] The linked genes RAG1 and RAG2 act together to activate immunoglobulin V-D-J recombination. RAG1 is involved in recognition of the DNA substrate.	63512.43	51032.4	12480.02	0.315625
S77361_at	MMP19	4327	601807	matrix metalloproteinase 19	angiogenesis; collagen catabolism; extracellular matrix; hydrolase activity; metalloendopeptidase activity; zinc ion binding	[SUMMARY:] Proteins of the matrix metalloproteinase (MMP) family are involved in the breakdown of extracellular matrix in normal physiological processes, such as embryonic development,reproduction, and tissue remodeling, as well as in disease processes, such as arthritis and metastasis. Most MMP's are secreted as inactive proproteins which are activated when cleaved by extracellular proteinases. The function of the protein encoded by this gene has not been determined. This gene was previously referred to as MMP18 but has been renamed matrix metalloproteinase 19 (MMP19). This gene encodes four transcript variants.	85159.58	72483.87	12675.71	0.232509
AFFX-BioC-							33434.58	19979.17	13455.42	0.742845

M86933_at	AMELY	266	410000	amelogenin, Y-linked	bone mineralization; development; extracellular matrix; extracellular matrix structural constituent; odontogenesis; structural constituent of tooth enamel	[SUMMARY:] This gene encodes a member of the amelogenin family of extracellular matrix proteins. Amelogenins are involved in biomineralization during tooth enamel development. Mutations in a related gene on chromosome X cause X-linked amelogenesis imperfecta.	57191.92	71060.73	13868.81	-0.313241
X72304_at	CRHR1	1394	122561	corticotropin releasing hormone receptor 1	G-protein coupled receptor activity; G-protein signaling, coupled to cAMP nucleotide second messenger; adenylate cyclase activation; corticotrophin-releasing factor receptor activity; immune response; integral to plasma membrane; parturition; pregnancy	[SUMMARY:] The corticotropin-releasing hormone receptor binds to corticotropin-releasing hormone (MIM 122560), a potent mediator of endocrine, autonomic, behavioral, and immune responses to stress.[supplied by OMIM]	160964.7	146863.9	14100.78	0.132264
D87017_cds3_at	IGL@	3535		immunoglobulin lambda locus		[SUMMARY:] Immunoglobulins recognize foreign antigens and initiate immune responses such as phagocytosis and the complement system. Each immunoglobulin molecule consists of two identical heavy chains and two identical light chains. There are two classes of light chains, kappa and lambda. This region represents the germline organization of the lambda light chain locus. The locus includes V (variable), J (joining), and C (constant) segments. During B cell development, a recombination event at the DNA level joins a single V segment with a J segment; the C segment is later joined by splicing at the RNA level. Recombination of many different V segments with several J segments provides a wide range of antigen recognition. Additional diversity is attained by junctional diversity, resulting from the random additional of nucleotides by terminal deoxynucleotidyltransferase, and by somatic hypermutation, which occurs during B cell maturation in the spleen and lymph nodes. Several V segments and three C segments are known to be incapable of encoding a protein and are considered pseudogenes. The	44030.48	29535.7	14494.78	0.576043

M57466_s_at	HLA-DPB1	3115	142858	major histocompatibility complex, class II, DP beta 1	MHC class II receptor activity; antigen presentation, exogenous antigen; antigen processing, exogenous antigen via MHC class II; immune response; integral to membrane	[SUMMARY:] HLA-DPB belongs to the HLA class II beta chain paralogues. This class II molecule is a heterodimer consisting of an alpha (DPA) and a beta chain (DPB), both anchored in the membrane. It plays a central role in the immune system by presenting peptides derived from extracellular proteins. Class II molecules are expressed in antigen presenting cells (APC: B lymphocytes, dendritic cells, macrophages). The beta chain is approximately 26-28 kDa and its gene contains 6 exons. Exon one encodes the leader peptide, exons 2 and 3 encode the two extracellular domains, exon 4 encodes the transmembrane domain and exon 5 encodes the cytoplasmic tail. Within the DP molecule both the alpha chain and the beta chain contain the polymorphisms specifying the peptide binding specificities, resulting in up to 4 different molecules.	17470.2	2903.9	14566.3	2.588833
U49844_at	ATR	545	601215	ataxia telangiectasia and Rad3 related	DNA repair; cell cycle; cell cycle checkpoint; development; inositol/phosphatidylinositol kinase activity; protein kinase activity; transferase activity	[SUMMARY:] The protein encoded by this gene belongs the PI3/PI4-kinase family, and is most closely related to ATM, a protein kinase encoded by the gene mutated in ataxia telangiectasia. This protein and ATM share similarity with Schizosaccharomyces pombe rad3, a cell cycle checkpoint gene required for cell cycle arrest and DNA damage repair in response to DNA damage. This kinase has been shown to phosphorylate checkpoint kinase CHK1, checkpoint proteins RAD17, and RAD9, as well as tumor suppressor protein BRCA1. Mutations of this gene are associated with Seckel syndrome. An alternatively spliced transcript variant of this gene has been reported, however, its full length nature is not known. Transcript variants utilizing alternative polyA sites exist.	87535.27	102424.7	14889.42	-0.226627
M38591_at	S100A10	6281	114085	S100 calcium binding protein A10 (annexin II ligand, calpactin I, light polypeptide (p11))		[SUMMARY:] The protein encoded by this gene is a member of the S100 family of proteins containing 2 EF-hand calcium-binding motifs. S100 proteins are localized in the cytoplasm and/or nucleus of a wide range of cells, and involved in the regulation of a number of cellular processes such as cell cycle progression and differentiation. S100 genes include at least 13 members which are located as a cluster on chromosome 1q21. This protein may function in exocytosis and endocytosis.	206496.6	191293.7	15202.89	0.110329
HG2460-							103952.7	88735.1	15217.63	0.228351

U34360_at	LAF4	3899	601464	lymphoid nuclear protein related to AF4	DNA binding; development; nucleus; regulation of transcription, DNA-dependent	[SUMMARY:] Lymphoid nuclear protein related to AF4 (LAF4) is related to MLLT2 which is also referred to as AF4. LAF4 is a tissue-restricted nuclear transcriptional activator that is preferentially expressed in lymphoid tissue. Isolation of LAF4 has defined a highly conserved LAF4/MLLT2 gene family of nuclear transcription factors that may function in lymphoid development and oncogenesis.	112782.9	97324.16	15458.7	0.212678
M31667_f_at	CYP1A2	1544	124060	cytochrome P450, family 1, subfamily A, polypeptide 2		[SUMMARY:] This gene, CYP1A2, encodes a member of the cytochrome P450 superfamily of enzymes. The cytochrome P450 proteins are monooxygenases which catalyze many reactions involved in drug metabolism and synthesis of cholesterol, steroids and other lipids. The protein encoded by this gene localizes to the endoplasmic reticulum and its expression is induced by some polycyclic aromatic hydrocarbons (PAHs), some of which are found in cigarette smoke. The enzyme's endogenous substrate is unknown; however, it is able to metabolize some PAHs to carcinogenic intermediates. Other xenobiotic substrates for this enzyme include caffeine, aflatoxin B1, and acetaminophen. The transcript from this gene contains four Alu sequences flanked by direct repeats in the 3' untranslated region. A related family member, CYP1A1, is located approximately 25 kb away from CYP1A2 on chromosome 15.	230515.2	214901.2	15614.03	0.101189
AFFX-BioB-							184982.9	169111	15871.92	0.129422
X02158_rna1_at	EPO	2056	133170	erythropoietin	cell-cell signaling; circulation; development; erythropoietin receptor binding; extracellular space; hormone activity; response to stress; signal transduction	[SUMMARY:] Human erythropoietin is an acidic glycoprotein hormone with a molecular mass of 34 kD. As the prime regulator of red cell production, its major functions are to promote erythroid differentiation and to initiate hemoglobin synthesis.[supplied by OMIM]	41131.96	24246.6	16885.36	0.762477
HG3636-HT3846_at	MYH9	4627	160775	myosin, heavy polypeptide 9, non-muscle	ATP binding; actin binding; calmodulin binding; cellular morphogenesis; motor activity; myosin; non-muscle myosin; perception of sound		41936.68	24546.9	17389.78	0.772672
Z84721_cds1_at	HBZ	3050	142310	hemoglobin, zeta		[SUMMARY:] Zeta-globin (HBZ) is an alpha-like hemoglobin. The zeta-globin polypeptide is synthesized in the yolk sac of the early embryo, while alpha-globin is produced throughout fetal and adult like. The zeta-globin gene is a member of the human alpha-globin gene cluster that involves 4 functional genes and 3 nonfunctional pseudogenes. The order of genes is: 5'-zeta -- pseudozeta -- pseudoalpha2 -- pseudoalpha1 -- alpha2 -- alph1 -- theta1-3'.	96124.27	78470.2	17654.07	0.292756

M13955_at	KRT7	3855	148059	keratin 7	cytoskeleton organization and biogenesis; intermediate filament; structural molecule activity	[SUMMARY:] The protein encoded by this gene is a member of the keratin gene family. The type II cytokeratins consist of basic or neutral proteins which are arranged in pairs of heterotypic keratin chains coexpressed during differentiation of simple and stratified epithelial tissues. This type II cytokeratin is specifically expressed in the simple epithelia lining the cavities of the internal organs and in the gland ducts and blood vessels. The genes encoding the type II cytokeratins are clustered in a region of chromosome 12q12-q13. Alternative splicing may result in several transcript variants; however, not all variants have been fully described.	54281.02	72043.6	17762.58	-0.408422
M30607_s_at	ZFY	7544	490000	zinc finger protein, Y-linked	DNA binding; nucleus; regulation of transcription, DNA-dependent	[SUMMARY:] This gene encodes a zinc finger-containing protein that may function as a transcription factor. This gene was once a candidate gene for the testis-determining factor (TDF) and was erroneously referred to as TDF.	91409.68	109347.2	17937.52	-0.258497
M83221_at	RELB	5971	604758	v-rel reticuloendotheliosis viral oncogene homolog B, nuclear factor of kappa light polypeptide gene enhancer in B-cells 3 (avian)	nucleus; protein binding; regulation of transcription, DNA-dependent; transcription corepressor activity; transcription factor activity		153747.2	135060.4	18686.83	0.186956
L21893_at	SLC10A1	6554	182396	solute carrier family 10 (sodium/bile acid cotransporter family), member 1	bile acid:sodium symporter activity; integral to plasma membrane; organic anion transport; sodium ion transport; symporter activity; transport		128722.6	109526.5	19196.09	0.232985
M21551_rna1_at	NMB	4828	162340	neuromedin B	cell-cell signaling; hormone activity; neuropeptide signaling pathway; signal transduction; soluble fraction		255836.5	236600.2	19236.22	0.11277
AFFX-BioDn-							39139.82	19000.37	20139.46	1.04261
M86528_at	NTF5	4909	162662	neurotrophin 5 (neurotrophin 4/5)	growth factor activity	[SUMMARY:] This gene is a member of a family of neurotrophic factors, neurotrophins, that control survival and differentiation of mammalian neurons. The expression of this gene is ubiquitous and less influenced by environmental signals. While knock-outs of other neurotrophins including nerve growth factor, brain-derived neurotrophic factor, and neurotrophin 3 prove lethal during early postnatal development, NTF5-deficient mice only show minor cellular deficits and develop normally to adulthood.	51495.74	30964.84	20530.91	0.733822

L06505_at	RPL12	6136	180475	ribosomal protein L12	RNA binding; cytosolic large ribosomal subunit (sensu Eukarya); intracellular; protein biosynthesis; ribosome; structural constituent of ribosome	[SUMMARY:] Ribosomes, the organelles that catalyze protein synthesis, consist of a small 40S subunit and a large 60S subunit. Together these subunits are composed of 4 RNA species and approximately 80 structurally distinct proteins. This gene encodes a ribosomal protein that is a component of the 60S subunit. The protein belongs to the L11P family of ribosomal proteins. It is located in the cytoplasm. The protein binds directly to the 26S rRNA. This gene is co-transcribed with the U65 snoRNA, which is located in its fourth intron. As is typical for genes encoding ribosomal proteins, there are multiple processed pseudogenes of this gene dispersed through the genome.	193816.7	170552.6	23264.09	0.184476
AFFX-HUMGAPDH/M33197_M_at	GAPD	2597	138400	glyceraldehyde-3-phosphate dehydrogenase	cytoplasm; glucose metabolism; glyceraldehyde-3-phosphate dehydrogenase (phosphorylating) activity; glycolysis; oxidoreductase activity	[SUMMARY:] Glyceraldehyde-3-phosphate dehydrogenase catalyzes an important energy-yielding step in carbohydrate metabolism, the reversible oxidative phosphorylation of glyceraldehyde-3-phosphate in the presence of inorganic phosphate and nicotinamide adenine dinucleotide (NAD). The enzyme exists as a tetramer of identical chains. A GAPD pseudogene has been mapped to Xp21-p11 and 15 GAPD-like loci have been identified.	213745	190082.3	23662.78	0.169267
M20681_at	SLC2A3	6515	138170	solute carrier family 2 (facilitated glucose transporter), member 3	carbohydrate metabolism; carbohydrate transport; glucose transport; glucose transporter activity; integral to membrane; membrane fraction; sugar porter activity; transporter activity		157438.5	133289.1	24149.44	0.24023
AFFX-HSAC07/X00351_5_at	ACTB	60	102630	actin, beta	actin filament; cell motility; motor activity; structural constituent of cytoskeleton	[SUMMARY:] Beta actin is one of six different actin isoforms which have been identified. ACTB is one of the two nonmuscle cytoskeletal actins. Actins are highly conserved proteins that are involved in cell motility, structure and integrity. Alpha actins are a major constituent of the contractile apparatus.	244351.5	220120.9	24230.56	0.150662
AFFX-BioDn-							40794.68	16342.4	24452.28	1.319761
AFFX-DapX-							257094.3	232534.5	24559.73	0.144852
M83363_at	ATP2B4	493	108732	ATPase, Ca++ transporting, plasma membrane 4	ATP binding; calcium ion binding; calcium ion transport; calcium-transporting ATPase activity; calmodulin binding; cation transport; hydrolase activity; hydrolase activity, acting on acid anhydrides, catalyzing transmembrane movement of substances; integral to plasma membrane; magnesium ion binding; metabolism; transport		167702.2	143104	24598.2	0.228838

U96915_at	SAP18	10284	602949	sin3-associated polypeptide, 18kDa	histone deacetylase complex; regulation of transcription from Pol II promoter; transcription corepressor activity	[SUMMARY:] Histone acetylation plays a key role in the regulation of eukaryotic gene expression. Histone acetylation and deacetylation are catalyzed by multisubunit complexes. The protein encoded by this gene is a component of the histone deacetylase complex which includes SIN3, SAP30, HDAC1, HDAC2, RbAp46, RbAp48 and other polypeptides. This protein directly interacts with SIN3 and enhances SIN3-mediated transcriptional repression when tethered to the promoter.	32689.56	57367.67	24678.11	-0.811408
X89399_s_at	RASA3	22821	605182	RAS p21 protein activator 3	GTPase activator activity; intracellular signaling cascade; plasma membrane	[SUMMARY:] The protein encoded by this gene is member of the GAP1 family of GTPase-activating proteins. The gene product stimulates the GTPase activity of normal RAS p21 but not its oncogenic counterpart. Acting as a suppressor of RAS function, the protein enhances the weak intrinsic GTPase activity of RAS proteins resulting in the inactive GDP-bound form of RAS, thereby allowing control of cellular proliferation and differentiation. This family member is an inositol 1,3,4,5-tetrakisphosphate-binding protein, like the closely related RAS p21 protein activator 2. The two family members have distinct pleckstrin-homology domains, with this particular member having a domain consistent with its localization to the plasma membrane.	178460.7	153255.3	25205.41	0.219669
M20747_s_at	SLC2A4	6517	138190	solute carrier family 2 (facilitated glucose transporter), member 4	carbohydrate metabolism; carbohydrate transport; glucose transport; glucose transporter activity; integral to plasma membrane; membrane fraction; sugar porter activity; transporter activity		134870.9	109573.3	25297.63	0.299683
S73813_at	ENTPD1	953	601752	ectonucleoside triphosphate diphosphohydrolase 1	antimicrobial humoral response (sensu Vertebrata); apyrase activity; blood coagulation; cell adhesion; cell-cell signaling; hydrolase activity; integral to plasma membrane; magnesium ion binding		172200.3	146896.8	25303.52	0.229285
X15943_at	CALCA	796	114130	calcitonin/calcitonin-related polypeptide, alpha	G-protein signaling, coupled to cAMP nucleotide second messenger; adenylate cyclase activation; cell-cell signaling; cellular_component unknown; cytosolic calcium ion concentration elevation; endoplasmic reticulum; extracellular space; hormone activity; phospholipase C activation; regulation of blood pressure; skeletal development; soluble fraction		171293.1	144588.8	26704.36	0.244512

X16135_at	HNRPL	3191	603083	heterogeneous nuclear ribonucleoprotein L	RNA binding; heterogeneous nuclear ribonucleoprotein complex; mRNA processing; nucleoplasm	[SUMMARY:] Heterogeneous nuclear RNAs (hnRNAs) which include mRNA precursors and mature mRNAs are associated with specific proteins to form heterogenous ribonucleoprotein (hnRNP) complexes. Heterogeneous nuclear ribonucleoprotein L is among the proteins that are stably associated with hnRNP complexes and along with other hnRNP proteins is likely to play a major role in the formation, packaging, processing, and function of mRNA. Heterogeneous nuclear ribonucleoprotein L is present in the nucleoplasm as part of the HNRP complex. HNRP proteins have also been identified outside of the nucleoplasm. Exchange of hnRNP for mRNA-binding proteins accompanies transport of mRNA from the nucleus to the cytoplasm. Since HNRP proteins have been shown to shuttle between the nucleus and the cytoplasm, it is possible that they also have cytoplasmic functions.~Strucutrally HNRPL contains 2 segments of approximately 80 amino acids each which are weakly related to each other and to the ribonucleoprotein consensus sequence-type RNA-binding domains of other hnRNP and snRNP proteins.	167805.6	140601.1	27204.48	0.255183
X64728_at	OPN3	23596	606695	opsin 3 (encephalopsin, panopsin)	G-protein coupled photoreceptor activity; G-protein coupled receptor protein signaling pathway; integral to membrane; phototransduction; regulation of circadian rhythm; visual perception	[SUMMARY:] Opsins, including OPN3, are members of the superfamily of guanine nucleotide-binding protein (G protein)-coupled receptors, which function through the activation of a G protein and an effector enzyme. Opsin proteins consist of a single polypeptide chain of 340 to 500 amino acids that form 7 alpha-helical transmembrane regions connected by cytoplasmic and extracellular loops.[supplied by OMIM]	215214.1	187963.8	27250.28	0.195318
HG1437-HT1437_s_at	NTRK1	4914	191315	neurotrophic tyrosine kinase, receptor, type 1	ATP binding; biological_process unknown; cell growth and/or maintenance; cellular_component unknown; integral to membrane; integral to plasma membrane; molecular_function unknown; neurogenesis; neurotrophin TRKA receptor activity; protein amino acid phosphorylation; receptor activity; transferase activity; transmembrane receptor protein tyrosine kinase activity; transmembrane receptor protein tyrosine kinase signaling pathway		155065.5	126077.9	28987.57	0.298562

AFFX-HUMGAPDH/M33197_3_at	GAPD	2597	138400	glyceraldehyde-3-phosphate dehydrogenase	cytoplasm; glucose metabolism; glyceraldehyde-3-phosphate dehydrogenase (phosphorylating) activity; glycolysis; oxidoreductase activity	[SUMMARY:] Glyceraldehyde-3-phosphate dehydrogenase catalyzes an important energy-yielding step in carbohydrate metabolism, the reversible oxidative phosphorylation of glyceraldehyde-3-phosphate in the presence of inorganic phosphate and nicotinamide adenine dinucleotide (NAD). The enzyme exists as a tetramer of identical chains. A GAPD pseudogene has been mapped to Xp21-p11 and 15 GAPD-like loci have been identified.	243353.8	214355.2	28998.55	0.183052
S67156_at	ASPA	443	608034	aspartoacylase (aminoacylase 2, Canavan disease)	aminoacylase activity; aspartate catabolism; aspartoacylase activity; hydrolase activity	[SUMMARY:] The ASPA gene product, aspartoacylase, catalyzes the conversion of N-acetyl_L-aspartic acid (NAA) to aspartate and acetate. NAA is abundant in the brain where hydrolysis by aspartoacylase is thought to help maintain white matter. The ASPA gene product is an NAA scavenger in other tissues. Defects in ASPA cause Canavan disease.	97751.66	68185.73	29565.93	0.519651
M21985_at	NR2C1	7181	601529	nuclear receptor subfamily 2, group C, member 1	nucleus; regulation of transcription, DNA-dependent; steroid hormone receptor activity; transcription; transcription factor activity		183606.8	153605.8	30000.94	0.257386
X07820_at	MMP10	4319	185260	matrix metalloproteinase 10 (stromelysin 2)	collagen catabolism; extracellular matrix; extracellular space; hydrolase activity; stromelysin 2 activity; zinc ion binding	[SUMMARY:] Proteins of the matrix metalloproteinase (MMP) family are involved in the breakdown of extracellular matrix in normal physiological processes, such as embryonic development, reproduction, and tissue remodeling, as well as in disease processes, such as arthritis and metastasis. Most MMP's are secreted as inactive proproteins which are activated when cleaved by extracellular proteinases. The enzyme encoded by this gene degrades proteoglycans and fibronectin. The gene is part of a cluster of MMP genes which localize to chromosome 11q22.3.	196873	162145.1	34727.91	0.27998
U95006_at	STRA13	201254		stimulated by retinoic acid 13			216587.8	181254.8	35333.06	0.256933
M21904_at	SLC3A2	6520	158070	solute carrier family 3 (activators of dibasic and neutral amino acid transport), member 2	alpha-amylase activity; amino acid transport; amino acid transporter activity; carbohydrate metabolism; cell growth; integral to membrane; sodium:calcium exchange		255137.4	217035.3	38102.08	0.233345
Z46632_at	PDE4C	5143	600128	phosphodiesterase 4C, cAMP-specific (phosphodiesterase E1 dunce homolog, Drosophila)	3',5'-cyclic-nucleotide phosphodiesterase activity; catalytic activity; cellular_component unknown; signal transduction		189884.8	151458.5	38426.28	0.326202

M19508_xpt3_s_at	MPO	4353	606989	myeloperoxidase	anti-apoptosis; calcium ion binding; chromatin binding; defense response; lysosome; nucleus; oxidoreductase activity; peroxidase activity; response to oxidative stress	[SUMMARY:] Myeloperoxidase (MPO) is a heme protein synthesized during myeloid differentiation that constitutes the major component of neutrophil azurophilic granules. Produced as a single chain precursor, myeloperoxidase is subsequently cleaved into a light and heavy chain. The mature myeloperoxidase is a tetramer composed of 2 light chains and 2 heavy chains. This enzyme produces hypohalous acids central to the microbicidal activity of netrophils.	180978.2	141433.8	39544.45	0.355689
V00594_s_at	MT2A	4502	156360	metallothionein 2A	copper ion binding; copper ion homeostasis; metal ion binding		243129	197424.1	45704.83	0.300423
M26730_s_at	UQCRB	7381	191330	ubiquinol-cytochrome c reductase binding protein			246359.2	186997.5	59361.61	0.397744
HG1428-HT1428_s_at	HBB	3043	141900	hemoglobin, beta	hemoglobin complex; oxygen transport; oxygen transporter activity; transport	[SUMMARY:] The alpha (HBA) and beta (HBB) loci determine the structure of the 2 types of polypeptide chains in adult hemoglobin, Hb A. The normal adult hemoglobin tetramer consists of two alpha chains and two beta chains. Mutant beta globin causes sickle cell anemia. Absence of beta chain causes beta-zero-thalassemia. Reduced amounts of detectable beta globin causes beta-plus-thalassemia. The order of the genes in the beta-globin cluster is 5'-epsilon -- gamma-G -- gamma-A -- delta -- beta--3'.	277172.2	217189.6	59982.56	0.351827
AFFX-CreX-							183641.7	119723.9	63917.72	0.617182
Z84722_at	C16orf35	8131	600928	chromosome 16 open reading frame 35	biological_process unknown; cellular_component unknown; molecular_function unknown					
