# Supplementary material for: Unsupervised clustering of gene expression data points at hypoxia as possible trigger for metabolic syndrome
Source: BMC Genomics. 2006 Dec 19;7:318. doi: 10.1186/1471-2164-7-318 (PMC1770922; doi:10.1186/1471-2164-7-318)
Supplement: Additional file 6 — Supplementary Table 5. This PDF file contains the table of genes, forming the largest cluster in FOREL clustering of genes in Mootha et al. data set. [file 1471-2164-7-318-S6.pdf]

Annotation Tool

AFFYID	VALUE	SYMBOL	LOCUSLINK	OMIM	GENENAME	GENEONTOLOGY	SUMMARY
203054_s_at		TCTA	6988	600690	T-cell leukemia translocation altered gene	tumor suppressor	[Proteome FUNCTION:] Expressed ubiquitously
44563_at		FLJ10385	55135		hypothetical protein FLJ10385		[Proteome FUNCTION:] May be involved in protein-protein interactions; contains five WD domains (WD-40 repeats)
212261_at		TNRC15	26058		trinucleotide repeat containing 15		[Proteome FUNCTION:] Weakly similarity to a region of rat nestin (Rn.9701)
203872_at		ACTA1	58	102610	actin, alpha 1, skeletal muscle	actin filament; motor activity; muscle contraction; muscle development; structural constituent of cytoskeleton	[SUMMARY:] Actin alpha 1 which is expressed in skeletal muscle is one of six different actin isoforms which have been identified. Actins are highly conserved proteins that are involved in cell motility, structure and integrity. Alpha actins are a major constituent of the contractile apparatus.
203074_at		ANXA8	244	602396	annexin A8		[SUMMARY:] Annexin VIII belong to the family of Ca (2+) dependent phospholipid binding proteins (annexins), and has a high 56% identity to annexin V (vascular anticoagulant-alpha). It was initially isolated as 2.2 kb vascular anticoagulant-beta transcript from human placenta, a Ca (2+) dependent phospholipid binding protein that inhibits coagulation and phospholipase A2 activity. However, the fact that annexin VIII is neither an extracellular protein nor associated with the cell surface suggests that it may not play a role in blood coagulation in vivo and its physiological role remains unknown. It is expressed at low levels in human placenta and shows restricted expression in lung endothelia, skin, liver, and kidney. The gene is also found to be selectively overexpressed in acute myelocytic leukemia.
AFFX-HSAC07/X00351_5_at		ACTB	60	102630	actin, beta	actin filament; cell motility; motor activity; structural constituent of cytoskeleton	[SUMMARY:] Beta actin is one of six different actin isoforms which have been identified. ACTB is one of the two nonmuscle cytoskeletal actins. Actins are highly conserved proteins that are involved in cell motility, structure and integrity. Alpha actins are a major constituent of the contractile apparatus.
212581_x_at		GAPD	2597	138400	glyceraldehyde-3-phosphate dehydrogenase	cytoplasm; glucose metabolism; glyceraldehyde-3-phosphate dehydrogenase (phosphorylating) activity; glycolysis; oxidoreductase activity	[SUMMARY:] Glyceraldehyde-3-phosphate dehydrogenase catalyzes an important energy-yielding step in carbohydrate metabolism, the reversible oxidative phosphorylation of glyceraldehyde-3-phosphate in the presence of inorganic phosphate and nicotinamide adenine dinucleotide (NAD). The enzyme exists as a tetramer of identical chains. A GAPD pseudogene has been mapped to Xp21-p11 and 15 GAPD-like loci have been identified.

213453_x_at		GAPD	2597	138400	glyceraldehyde-3-phosphate dehydrogenase	cytoplasm; glucose metabolism; glyceraldehyde-3-phosphate dehydrogenase (phosphorylating) activity; glycolysis; oxidoreductase activity	[SUMMARY:] Glyceraldehyde-3-phosphate dehydrogenase catalyzes an important energy-yielding step in carbohydrate metabolism, the reversible oxidative phosphorylation of glyceraldehyde-3-phosphate in the presence of inorganic phosphate and nicotinamide adenine dinucleotide (NAD). The enzyme exists as a tetramer of identical chains. A GAPD pseudogene has been mapped to Xp21-p11 and 15 GAPD-like loci have been identified.
217398_x_at		GAPD	2597	138400	glyceraldehyde-3-phosphate dehydrogenase	cytoplasm; glucose metabolism; glyceraldehyde-3-phosphate dehydrogenase (phosphorylating) activity; glycolysis; oxidoreductase activity	[SUMMARY:] Glyceraldehyde-3-phosphate dehydrogenase catalyzes an important energy-yielding step in carbohydrate metabolism, the reversible oxidative phosphorylation of glyceraldehyde-3-phosphate in the presence of inorganic phosphate and nicotinamide adenine dinucleotide (NAD). The enzyme exists as a tetramer of identical chains. A GAPD pseudogene has been mapped to Xp21-p11 and 15 GAPD-like loci have been identified.
AFFX-HUMGAPDH/M33197_3_at		GAPD	2597	138400	glyceraldehyde-3-phosphate dehydrogenase	cytoplasm; glucose metabolism; glyceraldehyde-3-phosphate dehydrogenase (phosphorylating) activity; glycolysis; oxidoreductase activity	[SUMMARY:] Glyceraldehyde-3-phosphate dehydrogenase catalyzes an important energy-yielding step in carbohydrate metabolism, the reversible oxidative phosphorylation of glyceraldehyde-3-phosphate in the presence of inorganic phosphate and nicotinamide adenine dinucleotide (NAD). The enzyme exists as a tetramer of identical chains. A GAPD pseudogene has been mapped to Xp21-p11 and 15 GAPD-like loci have been identified.
AFFX-HUMGAPDH/M33197_5_at		GAPD	2597	138400	glyceraldehyde-3-phosphate dehydrogenase	cytoplasm; glucose metabolism; glyceraldehyde-3-phosphate dehydrogenase (phosphorylating) activity; glycolysis; oxidoreductase activity	[SUMMARY:] Glyceraldehyde-3-phosphate dehydrogenase catalyzes an important energy-yielding step in carbohydrate metabolism, the reversible oxidative phosphorylation of glyceraldehyde-3-phosphate in the presence of inorganic phosphate and nicotinamide adenine dinucleotide (NAD). The enzyme exists as a tetramer of identical chains. A GAPD pseudogene has been mapped to Xp21-p11 and 15 GAPD-like loci have been identified.
AFFX-HUMGAPDH/M33197_M_at		GAPD	2597	138400	glyceraldehyde-3-phosphate dehydrogenase	cytoplasm; glucose metabolism; glyceraldehyde-3-phosphate dehydrogenase (phosphorylating) activity; glycolysis; oxidoreductase activity	[SUMMARY:] Glyceraldehyde-3-phosphate dehydrogenase catalyzes an important energy-yielding step in carbohydrate metabolism, the reversible oxidative phosphorylation of glyceraldehyde-3-phosphate in the presence of inorganic phosphate and nicotinamide adenine dinucleotide (NAD). The enzyme exists as a tetramer of identical chains. A GAPD pseudogene has been mapped to Xp21-p11 and 15 GAPD-like loci have been identified.

38447_at		ADRBK1	156	109635	adrenergic, beta, receptor kinase 1	ATP binding; G-protein coupled receptor kinase activity; beta-adrenergic-receptor kinase activity; cytoplasm; protein amino acid phosphorylation; signal transducer activity; signal transduction; soluble fraction; transferase activity	[SUMMARY:] Beta-adrenergic receptor kinase (ADRBK1) phosphorylates the beta-2-adrenergic receptor and appears to mediate agonist-specific desensitization observed at high agonist concentrations. ADRBK1 is an ubiquitous cytosolic enzyme that specifically phosphorylates the activated form of the beta-adrenergic and related G-protein-coupled receptors. The ADRBK1 gene spans approximately 23 kb and is composed of 21 exons. Heart failure is accompanied by severely impaired beta-adrenergic receptor (beta-AR) function. An important mechanism for the rapid desensitization of beta-AR function is agonist-stimulated receptor phosphorylation by the beta-AR kinase (beta-ARK1), an enzyme known to be elevated in failing human heart tissue. Abnormal coupling of beta-adrenergic receptor to G protein is involved in the pathogenesis of the failing heart. Inhibition of ADRBK1 is a novel mode of therapy.
203899_s_at		RCP9	27297	606121	calcitonin gene-related peptide-receptor component protein	acrosome reaction; calcitonin receptor activity; cellular_component unknown	[SUMMARY:] Calcitonin gene-related peptide (CGRP; MIM 114130), a 37-amino acid neuropeptide, induces increased intracellular cAMP levels. This occurs when CGRP binds to a CGRP receptor (CRLR, or CGRPR; MIM 114190) after the latter is transported to the cell surface by RAMP1 (MIM 605153). CGRPR component protein, or CGRPRCP, modulates CGRP responsiveness in a variety of cell types.[supplied by OMIM]
203658_at		SLC25A20	788	212138	solute carrier family 25 (carnitine/acylcarnitine translocase), member 20	binding; integral to membrane; lipid transporter activity; mitochondrial inner membrane; mitochondrion; transport	[SUMMARY:] Carnitine-acylcarnitine translocase is located at the mitochondrial inner membrane where it transfers fatty acylcarnitines into mitochondria. Thus it is critical in the fatty acid oxidation process. A defect in this translocase impairs oxidation of fatty acids and can cause a varieties of pathological conditions such as hypoglycemia, cardiac arrest, hepatomegaly, hepatic dysfunction and muscle weakness.
205021_s_at		CHES1	1112	602628	checkpoint suppressor 1	DNA damage response, signal transduction resulting in cell cycle arrest; G2 phase of mitotic cell cycle; nucleus; regulation of transcription, DNA-dependent; transcription factor activity	[SUMMARY:] Checkpoint suppresor 1 is a member of the forkhead/winged helix transcription factor family. Checkpoints are eukaryotic DNA damage-inducible cell cycle arrests at G1 and G2. Checkpoint suppresor 1 suppresses multiple yeast checkpoint mutations including mec1, rad9, rad53 and dun1 by activating a MEC1-independent checkpoint pathway.

200933_x_at		RPS4X	6191	312760	ribosomal protein S4, X-linked		[SUMMARY:] Cytoplasmic ribosomes, organelles that catalyze protein synthesis, consist of a small 40S subunit and a large 60S subunit. Together these subunits are composed of 4 RNA species and approximately 80 structurally distinct proteins. This gene encodes ribosomal protein S4, a component of the 40S subunit. Ribosomal protein S4 is the only ribosomal protein known to be encoded by more than one gene, namely this gene and ribosomal protein S4, Y-linked (RPS4Y). The 2 isoforms encoded by these genes are not identical, but are functionally equivalent. Ribosomal protein S4 belongs to the S4E family of ribosomal proteins. This gene is not subject to X-inactivation. It has been suggested that haploinsufficiency of the ribosomal protein S4 genes plays a role in Turner syndrome; however, this hypothesis is controversial. As is typical for genes encoding ribosomal proteins, there are multiple processed pseudogenes of this gene dispersed through the genome.
213347_x_at		RPS4X	6191	312760	ribosomal protein S4, X-linked		[SUMMARY:] Cytoplasmic ribosomes, organelles that catalyze protein synthesis, consist of a small 40S subunit and a large 60S subunit. Together these subunits are composed of 4 RNA species and approximately 80 structurally distinct proteins. This gene encodes ribosomal protein S4, a component of the 40S subunit. Ribosomal protein S4 is the only ribosomal protein known to be encoded by more than one gene, namely this gene and ribosomal protein S4, Y-linked (RPS4Y). The 2 isoforms encoded by these genes are not identical, but are functionally equivalent. Ribosomal protein S4 belongs to the S4E family of ribosomal proteins. This gene is not subject to X-inactivation. It has been suggested that haploinsufficiency of the ribosomal protein S4 genes plays a role in Turner syndrome; however, this hypothesis is controversial. As is typical for genes encoding ribosomal proteins, there are multiple processed pseudogenes of this gene dispersed through the genome.
220725_x_at		FLJ23558	80165		hypothetical protein FLJ23558		[SUMMARY:] DISCONTINUED: LocusID 80165 was defined by NM_025095.1 and AK027211.1 which do not appear to represent a protein coding gene
208120_x_at		FKSG63	83399		hypothetical protein FKSG63		[SUMMARY:] DISCONTINUED: LocusID 83399 was defined by AF338192.1, which is comprised of repeat sequence and does not appear to represent a mRNA from a protein-coding gene.

1598_g_at		GAS6	2621	600441	growth arrest-specific 6	calcium ion binding; cell proliferation; extracellular; receptor binding; signal transduction	[SUMMARY:] GAS6 is a gamma-carboxyglutamic acid (Gla)-containing protein thought to be involved in the stimulation of cell proliferation
206846_s_at		HDAC6	10013	300272	histone deacetylase 6	actin binding; cell cycle; chromatin modification; cytoplasm; development; enzyme binding; histone deacetylation; hydrolase activity; nucleus; regulation of transcription, DNA-dependent; specific transcriptional repressor activity; zinc ion binding	[SUMMARY:] Histones play a critical role in transcriptional regulation, cell cycle progression, and developmental events. Histone acetylation/deacetylation alters chromosome structure and affects transcription factor access to DNA. The protein encoded by this gene belongs to class II of the histone deacetylase/acuc/apha family. It contains an internal duplication of two catalytic domains which appear to function independently of each other. This protein possesses histone deacetylase activity and represses transcription.
211252_x_at		PTCRA	171558	606817	pre T-cell antigen receptor alpha		[SUMMARY:] In immature T cells the T-cell receptor beta-chain gene (TCRB; MIM 186930) is rearranged and expressed before the TCRA (MIM 186880) chain. At this early stage, TCRB can associate with the pre-T-cell receptor alpha chain (PTCRA). The PTCRA, together with TCRB and the CD3 complex (see MIM 186740), minimally make up the pre-T cell receptor (pre-TCR), which regulates early T cell development.[supplied by OMIM]
207187_at		JAK3	3718	600173	Janus kinase 3 (a protein tyrosine kinase, leukocyte)	cell growth and/or maintenance; mesoderm development; protein amino acid phosphorylation; protein-tyrosine kinase activity	[SUMMARY:] JAK3 encodes janus kinase 3, a tyrosine kinase that belongs to the janus family. JAK3 functions in signal transduction and interacts with members of the STAT (signal transduction and activators of transcription) family. JAK3 is predominantly expressed in immune cells and transduces a signal in response to its activation via tyrosine phosphorylation by interleukin receptors. Mutations that abrogate janus kinase 3 function cause an autosomal SCID (severe combined immunodeficiency disease).

208035_at		GRM6	2916	604096	glutamate receptor, metabotropic 6	G-protein coupled receptor protein signaling pathway; detection of visible light; integral to plasma membrane; metabotropic glutamate receptor signaling pathway; metabotropic glutamate, GABA-B-like receptor activity; visual perception	[SUMMARY:] L-glutamate is the major excitatory neurotransmitter in the central nervous system and activates both ionotropic and metabotropic glutamate receptors. Glutamatergic neurotransmission is involved in most aspects of normal brain function and can be perturbed in many neuropathologic conditions. The metabotropic glutamate receptors are a family of G protein-coupled receptors, that have been divided into 3 groups on the basis of sequence homology, putative signal transduction mechanisms, and pharmacologic properties. Group I includes GRM1 and GRM5 and these receptors have been shown to activate phospholipase C. Group II includes GRM2 and GRM3 while Group III includes GRM4, GRM6, GRM7 and GRM8. Group II and III receptors are linked to the inhibition of the cyclic AMP cascade but differ in their agonist selectivities.
202292_x_at		LYPLA2	11313		lysophospholipase II	fatty acid metabolism; hydrolase activity	[SUMMARY:] Lysophospholipases are enzymes that act on biological membranes to regulate the multifunctional lysophospholipids. There are alternatively spliced transcript variants described for this gene but the full length nature is not known yet.
36907_at		MVK	4598	251170	mevalonate kinase (mevalonic aciduria)	ATP binding; biosynthesis; cholesterol biosynthesis; cytoplasm; isoprenoid biosynthesis; mevalonate kinase activity; peroxisome; protein amino acid phosphorylation; transferase activity	[SUMMARY:] MVK encodes the peroxisomal enzyme mevalonate kinase. Mevalonate is a key intermediate, and mevalonate kinase a key early enzyme, in isoprenoid and sterol synthesis. Mevalonate kinase deficiency caused by mutation of MVK results in mevalonic aciduria.
206263_at		FMO4	2329	136131	flavin containing monooxygenase 4	dimethylaniline monooxygenase (N-oxide-forming) activity; electron transport; integral to membrane; microsome; xenobiotic metabolism	[SUMMARY:] Metabolic N-oxidation of the diet-derived amino-trimethylamine (TMA) is mediated by flavin-containing monooxygenase and is subject to an inherited FMO3 polymorphism in man resulting in a small subpopulation with reduced TMA N-oxidation capacity resulting in fish odor syndrome Trimethylaminuria. Three forms of the enzyme, FMO1 found in fetal liver, FMO2 found in adult liver, and FMO3 are encoded by genes clustered in the 1q23-q25 region. Flavin-containing monooxygenases are NADPH-dependent flavoenzymes that catalyzes the oxidation of soft nucleophilic heteroatom centers in drugs, pesticides, and xenobiotics.

211364_at		MTAP	4507	156540	methylthioadenosine phosphorylase	5'-methylthioadenosine phosphorylase activity; nucleobase, nucleoside, nucleotide and nucleic acid metabolism; phosphorylase activity; transferase activity, transferring glycosyl groups	[SUMMARY:] Methylthioadenosine phosphorylase plays a major role in polyamine metabolism and is important for the salvage of both adenine and methionine. Methylthioadenosine phosphorylase is deficienct in many cancers due primarily to codeletion of MTAP and the tumor suppressor gene p16 gene.
209888_s_at		MYL1	4632	160780	myosin, light polypeptide 1, alkali; skeletal, fast	calcium ion binding; muscle development; muscle myosin; myosin; structural constituent of muscle	[SUMMARY:] Myosin is a hexameric ATPase cellular motor protein. It is composed of two heavy chains, two nonphosphorylatable alkali light chains, and two phosphorylatable regulatory light chains. This gene encodes a myosin alkali light chain expressed in fast skeletal muscle. Two transcript variants have been identified for this gene.
216054_x_at		MYL4	4635	160770	myosin, light polypeptide 4, alkali; atrial, embryonic	calcium ion binding; muscle development; muscle myosin; myosin; structural constituent of muscle	[SUMMARY:] Myosin is a hexameric ATPase cellular motor protein. It is composed of two myosin heavy chains, two nonphosphorylatable myosin alkali light chains, and two phosphorylatable myosin regulatory light chains. This gene encodes a myosin alkali light chain that is found in embryonic muscle and adult atria.
206323_x_at		OPHN1	4983	300127	oligophrenin 1	Rho GTPase activator activity; axon guidance; neurogenesis; signal transduction; substrate-bound cell migration, cell extension	[SUMMARY:] Oligophrenin 1 has 25 exons and encodes a Rho-GTPase-activating protein. The Rho proteins are important mediators of intracellular signal transduction, which affects cell migration and cell morphogenesis. Mutations in this gene are responsible for non-specific X-linked mental retardation.
160020_at		MMP14	4323	600754	matrix metalloproteinase 14 (membrane-inserted)		[SUMMARY:] Proteins of the matrix metalloproteinase (MMP) family are involved in the breakdown of extracellular matrix in normal physiological processes, such as embryonic development, reproduction, and tissue remodeling, as well as in disease processes, such as arthritis and metastasis. Most MMP's are secreted as inactive proproteins which are activated when cleaved by extracellular proteinases. However, the protein encoded by this gene is a member of the membrane-type MMP (MT-MMP) subfamily; each member of this subfamily contains a potential transmembrane domain suggesting that these proteins are expressed at the cell surface rather than secreted. This protein activates MMP2 protein, and this activity may be involved in tumor invasion.

200763_s_at		RPLP1	6176	180520	ribosomal protein, large, P1	RNA binding; cytosolic large ribosomal subunit (sensu Eukarya); intracellular; protein biosynthesis; ribosome; structural constituent of ribosome; translational elongation	[SUMMARY:] Ribosomes, the organelles that catalyze protein synthesis, consist of a small 40S subunit and a large 60S subunit. Together these subunits are composed of 4 RNA species and approximately 80 structurally distinct proteins. This gene encodes a ribosomal phosphoprotein that is a component of the 60S subunit. The protein, which is a functional equivalent of the E. coli L7/L12 ribosomal protein, belongs to the L12P family of ribosomal proteins. It plays an important role in the elongation step of protein synthesis. Unlike most ribosomal proteins, which are basic, the encoded protein is acidic. Its C-terminal end is nearly identical to the C-terminal ends of the ribosomal phosphoproteins P0 and P2. The P1 protein can interact with P0 and P2 to form a pentameric complex consisting of P1 and P2 dimers, and a P0 monomer. The protein is located in the cytoplasm. As is typical for genes encoding ribosomal proteins, there are multiple processed pseudogenes of this gene dispersed through the genome.
201094_at		RPS29	6235	603633	ribosomal protein S29		[SUMMARY:] Ribosomes, the organelles that catalyze protein synthesis, consist of a small 40S subunit and a large 60S subunit. Together these subunits are composed of 4 RNA species and approximately 80 structurally distinct proteins. This gene encodes a ribosomal protein that is a component of the 40S subunit and a member of the S14P family of ribosomal proteins. The protein, which contains a C2-C2 zinc finger-like domain that can bind to zinc, can enhance the tumor suppressor activity of Ras related protein 1A (KREV1). It is located in the cytoplasm. Variable expression of this gene in colorectal cancers compared to adjacent normal tissues has been observed, although no correlation between the level of expression and the severity of the disease has been found. As is typical for genes encoding ribosomal proteins, there are multiple processed pseudogenes of this gene dispersed through the genome.

200095_x_at		RPS10	6204	603632	ribosomal protein S10	RNA binding; cytosolic small ribosomal subunit (sensu Eukarya); protein biosynthesis; structural constituent of ribosome	[SUMMARY:] Ribosomes, the organelles that catalyze protein synthesis, consist of a small 40S subunit and a large 60S subunit. Together these subunits are composed of 4 RNA species and approximately 80 structurally distinct proteins. This gene encodes a ribosomal protein that is a component of the 40S subunit. The protein belongs to the S10E family of ribosomal proteins. It is located in the cytoplasm. Variable expression of this gene in colorectal cancers compared to adjacent normal tissues has been observed, although no correlation between the level of expression and the severity of the disease has been found. As is typical for genes encoding ribosomal proteins, there are multiple processed pseudogenes of this gene dispersed through the genome.
208645_s_at		RPS14	6208	130620	ribosomal protein S14	RNA binding; cytosolic small ribosomal subunit (sensu Eukarya); intracellular; protein biosynthesis; ribosome; structural constituent of ribosome	[SUMMARY:] Ribosomes, the organelles that catalyze protein synthesis, consist of a small 40S subunit and a large 60S subunit. Together these subunits are composed of 4 RNA species and approximately 80 structurally distinct proteins. This gene encodes a ribosomal protein that is a component of the 40S subunit. The protein belongs to the S11P family of ribosomal proteins. It is located in the cytoplasm. Transcript variants utilizing alternative transcription initiation sites have been described in the literature. As is typical for genes encoding ribosomal proteins, there are multiple processed pseudogenes of this gene dispersed through the genome. In Chinese hamster ovary cells, mutations in this gene can lead to resistance to emetine, a protein synthesis inhibitor.
200926_at		RPS23	6228	603683	ribosomal protein S23		[SUMMARY:] Ribosomes, the organelles that catalyze protein synthesis, consist of a small 40S subunit and a large 60S subunit. Together these subunits are composed of 4 RNA species and approximately 80 structurally distinct proteins. This gene encodes a ribosomal protein that is a component of the 40S subunit. The protein belongs to the S12P family of ribosomal proteins. It is located in the cytoplasm. The protein shares significant amino acid similarity with S. cerevisiae ribosomal protein S28. As is typical for genes encoding ribosomal proteins, there are multiple processed pseudogenes of this gene dispersed through the genome.

200018_at		RPS13	6207	180476	ribosomal protein S13		[SUMMARY:] Ribosomes, the organelles that catalyze protein synthesis, consist of a small 40S subunit and a large 60S subunit. Together these subunits are composed of 4 RNA species and approximately 80 structurally distinct proteins. This gene encodes a ribosomal protein that is a component of the 40S subunit. The protein belongs to the S15P family of ribosomal proteins. It is located in the cytoplasm. The protein has been shown to bind to the 5.8S rRNA in rat. The gene product of the E. coli ortholog (ribosomal protein S15) functions at early steps in ribosome assembly. This gene is co-transcribed with two U14 small nucleolar RNA genes, which are located in its third and fifth introns. As is typical for genes encoding ribosomal proteins, there are multiple processed pseudogenes of this gene dispersed through the genome.
200031_s_at		RPS11	6205	180471	ribosomal protein S11	intracellular; protein biosynthesis; rRNA binding; ribosome; structural constituent of ribosome	[SUMMARY:] Ribosomes, the organelles that catalyze protein synthesis, consist of a small 40S subunit and a large 60S subunit. Together these subunits are composed of 4 RNA species and approximately 80 structurally distinct proteins. This gene encodes a ribosomal protein that is a component of the 40S subunit. The protein belongs to the S17P family of ribosomal proteins. It is located in the cytoplasm. The gene product of the E. coli ortholog (ribosomal protein S17) is thought to be involved in the recognition of termination codons. This gene is co-transcribed with a small nucleolar RNA gene, which is located in its third intron. As is typical for genes encoding ribosomal proteins, there are multiple processed pseudogenes of this gene dispersed through the genome.

200741_s_at		RPS27	6232	603702	ribosomal protein S27 (metallopanstimulin 1)		[SUMMARY:] Ribosomes, the organelles that catalyze protein synthesis, consist of a small 40S subunit and a large 60S subunit. Together these subunits are composed of 4 RNA species and approximately 80 structurally distinct proteins. This gene encodes a ribosomal protein that is a component of the 40S subunit. The protein belongs to the S27E family of ribosomal proteins. It contains a C4-type zinc finger domain that can bind to zinc. The encoded protein has been shown to be able to bind to nucleic acid. It is located in the cytoplasm as a ribosomal component, but it has also been detected in the nucleus. Studies in rat indicate that ribosomal protein S27 is located near ribosomal protein S18 in the 40S subunit and is covalently linked to translation initiation factor eIF3. As is typical for genes encoding ribosomal proteins, there are multiple processed pseudogenes of this gene dispersed through the genome.
201257_x_at		RPS3A	6189	180478	ribosomal protein S3A		[SUMMARY:] Ribosomes, the organelles that catalyze protein synthesis, consist of a small 40S subunit and a large 60S subunit. Together these subunits are composed of 4 RNA species and approximately 80 structurally distinct proteins. This gene encodes a ribosomal protein that is a component of the 40S subunit. The protein belongs to the S3AE family of ribosomal proteins. It is located in the cytoplasm. Disruption of the gene encoding rat ribosomal protein S3a, also named v-fos transformation effector protein, in v-fos-transformed rat cells results in reversion of the transformed phenotype. Transcript variants utilizing alternative transcription start sites have been described. This gene is co-transcribed with the U73A and U73B small nucleolar RNA genes, which are located in its fourth and third introns, respectively. As is typical for genes encoding ribosomal proteins, there are multiple processed pseudogenes of this gene dispersed through the genome.

212391_x_at		RPS3A	6189	180478	ribosomal protein S3A		[SUMMARY:] Ribosomes, the organelles that catalyze protein synthesis, consist of a small 40S subunit and a large 60S subunit. Together these subunits are composed of 4 RNA species and approximately 80 structurally distinct proteins. This gene encodes a ribosomal protein that is a component of the 40S subunit. The protein belongs to the S3AE family of ribosomal proteins. It is located in the cytoplasm. Disruption of the gene encoding rat ribosomal protein S3a, also named v-fos transformation effector protein, in v-fos-transformed rat cells results in reversion of the transformed phenotype. Transcript variants utilizing alternative transcription start sites have been described. This gene is co-transcribed with the U73A and U73B small nucleolar RNA genes, which are located in its fourth and third introns, respectively. As is typical for genes encoding ribosomal proteins, there are multiple processed pseudogenes of this gene dispersed through the genome.
213890_x_at		RPS16	6217	603675	ribosomal protein S16		[SUMMARY:] Ribosomes, the organelles that catalyze protein synthesis, consist of a small 40S subunit and a large 60S subunit. Together these subunits are composed of 4 RNA species and approximately 80 structurally distinct proteins. This gene encodes a ribosomal protein that is a component of the 40S subunit. The protein belongs to the S9P family of ribosomal proteins. It is located in the cytoplasm. As is typical for genes encoding ribosomal proteins, there are multiple processed pseudogenes of this gene dispersed through the genome.
203034_s_at		RPL27A	6157	603637	ribosomal protein L27a		[SUMMARY:] Ribosomes, the organelles that catalyze protein synthesis, consist of a small 40S subunit and a large 60S subunit. Together these subunits are composed of 4 RNA species and approximately 80 structurally distinct proteins. This gene encodes a ribosomal protein that is a component of the 60S subunit. The protein belongs to the L15P family of ribosomal proteins. It is located in the cytoplasm. Variable expression of this gene in colorectal cancers compared to adjacent normal tissues has been observed, although no correlation between the level of expression and the severity of the disease has been found. As is typical for genes encoding ribosomal proteins, multiple processed pseudogenes derived from this gene are dispersed through the genome.

200869_at		RPL18A	6142	604178	ribosomal protein L18a	RNA binding; cytosolic large ribosomal subunit (sensu Eukarya); intracellular; protein biosynthesis; ribosome; structural constituent of ribosome	[SUMMARY:] Ribosomes, the organelles that catalyze protein synthesis, consist of a small 40S subunit and a large 60S subunit. Together these subunits are composed of 4 RNA species and approximately 80 structurally distinct proteins. This gene encodes a ribosomal protein that is a component of the 60S subunit. The protein belongs to the L18AE family of ribosomal proteins. It is located in the cytoplasm. This gene is co-transcribed with the U68 snoRNA, which is located in its third intron. As is typical for genes encoding ribosomal proteins, there are multiple processed pseudogenes of this gene dispersed through the genome.
208825_x_at		RPL23A	6147	602326	ribosomal protein L23a	cytosolic large ribosomal subunit (sensu Eukarya); intracellular; protein biosynthesis; rRNA binding; ribosome; structural constituent of ribosome	[SUMMARY:] Ribosomes, the organelles that catalyze protein synthesis, consist of a small 40S subunit and a large 60S subunit. Together these subunits are composed of 4 RNA species and approximately 80 structurally distinct proteins. This gene encodes a ribosomal protein that is a component of the 60S subunit. The protein belongs to the L23P family of ribosomal proteins. It is located in the cytoplasm. The protein may be one of the target molecules involved in mediating growth inhibition by interferon. In yeast, the corresponding protein binds to a specific site on the 26S rRNA. This gene is co-transcribed with the U42A, U42B, U101A, and U101B small nucleolar RNA genes, which are located in its third, first, second, and fourth introns, respectively. As is typical for genes encoding ribosomal proteins, there are multiple processed pseudogenes of this gene dispersed through the genome.

208834_x_at		RPL23A	6147	602326	ribosomal protein L23a	cytosolic large ribosomal subunit (sensu Eukarya); intracellular; protein biosynthesis; rRNA binding; ribosome; structural constituent of ribosome	[SUMMARY:] Ribosomes, the organelles that catalyze protein synthesis, consist of a small 40S subunit and a large 60S subunit. Together these subunits are composed of 4 RNA species and approximately 80 structurally distinct proteins. This gene encodes a ribosomal protein that is a component of the 60S subunit. The protein belongs to the L23P family of ribosomal proteins. It is located in the cytoplasm. The protein may be one of the target molecules involved in mediating growth inhibition by interferon. In yeast, the corresponding protein binds to a specific site on the 26S rRNA. This gene is co-transcribed with the U42A, U42B, U101A, and U101B small nucleolar RNA genes, which are located in its third, first, second, and fourth introns, respectively. As is typical for genes encoding ribosomal proteins, there are multiple processed pseudogenes of this gene dispersed through the genome.
213084_x_at		RPL23A	6147	602326	ribosomal protein L23a	cytosolic large ribosomal subunit (sensu Eukarya); intracellular; protein biosynthesis; rRNA binding; ribosome; structural constituent of ribosome	[SUMMARY:] Ribosomes, the organelles that catalyze protein synthesis, consist of a small 40S subunit and a large 60S subunit. Together these subunits are composed of 4 RNA species and approximately 80 structurally distinct proteins. This gene encodes a ribosomal protein that is a component of the 60S subunit. The protein belongs to the L23P family of ribosomal proteins. It is located in the cytoplasm. The protein may be one of the target molecules involved in mediating growth inhibition by interferon. In yeast, the corresponding protein binds to a specific site on the 26S rRNA. This gene is co-transcribed with the U42A, U42B, U101A, and U101B small nucleolar RNA genes, which are located in its third, first, second, and fourth introns, respectively. As is typical for genes encoding ribosomal proteins, there are multiple processed pseudogenes of this gene dispersed through the genome.

201429_s_at		RPL37A	6168		ribosomal protein L37a		[SUMMARY:] Ribosomes, the organelles that catalyze protein synthesis, consist of a small 40S subunit and a large 60S subunit. Together these subunits are composed of 4 RNA species and approximately 80 structurally distinct proteins. This gene encodes a ribosomal protein that is a component of the 60S subunit. The protein belongs to the L37AE family of ribosomal proteins. It is located in the cytoplasm. The protein contains a C4-type zinc finger-like domain. As is typical for genes encoding ribosomal proteins, there are multiple processed pseudogenes of this gene dispersed through the genome.
202029_x_at		RPL38	6169	604182	ribosomal protein L38		[SUMMARY:] Ribosomes, the organelles that catalyze protein synthesis, consist of a small 40S subunit and a large 60S subunit. Together these subunits are composed of 4 RNA species and approximately 80 structurally distinct proteins. This gene encodes a ribosomal protein that is a component of the 60S subunit. The protein belongs to the L38E family of ribosomal proteins. It is located in the cytoplasm. As is typical for genes encoding ribosomal proteins, there are multiple processed pseudogenes of this gene dispersed through the genome, including one located in the promoter region of the type 1 angiotensin II receptor gene.
208695_s_at		RPL39	6170	601904	ribosomal protein L39	RNA binding; cytosolic large ribosomal subunit (sensu Eukarya); protein biosynthesis; structural protein of ribosome	[SUMMARY:] Ribosomes, the organelles that catalyze protein synthesis, consist of a small 40S subunit and a large 60S subunit. Together these subunits are composed of 4 RNA species and approximately 80 structurally distinct proteins. This gene encodes a ribosomal protein that is a component of the 60S subunit. The protein belongs to the S39E family of ribosomal proteins. It is located in the cytoplasm. In rat, the protein is the smallest, and one of the most basic, proteins of the ribosome. This gene is co-transcribed with the U69 small nucleolar RNA gene, which is located in its second intron. As is typical for genes encoding ribosomal proteins, there are multiple processed pseudogenes of this gene dispersed through the genome.

201492_s_at		RPL41	6171		ribosomal protein L41	RNA binding; cytosolic large ribosomal subunit (sensu Eukarya); protein biosynthesis; structural constituent of ribosome	[SUMMARY:] Ribosomes, the organelles that catalyze protein synthesis, consist of a small 40S subunit and a large 60S subunit. Together these subunits are composed of 4 RNA species and approximately 80 structurally distinct proteins. This gene encodes a ribosomal protein that is a component of the 60S subunit. The protein, which shares sequence similarity with the yeast ribosomal protein YL41, belongs to the L41E family of ribosomal proteins. It is located in the cytoplasm. The protein can interact with the beta subunit of protein kinase CKII and can stimulate the phosphorylation of DNA topoisomerase II-alpha by CKII. As is typical for genes encoding ribosomal proteins, there are multiple processed pseudogenes of this gene dispersed through the genome.
221489_s_at		SPRY4	81848	607984	sprouty homolog 4 (Drosophila)		[SUMMARY:] SPRY4 is an inhibitor of the receptor-transduced mitogen-activated protein kinase (MAPK) signaling pathway. It is positioned upstream of RAS (see HRAS; MIM 190020) activation and impairs the formation of active GTP-RAS (Leeksma et al., 2002 [PubMed 12027893]).[supplied by OMIM]
206057_x_at		SPN	6693	182160	sialophorin (gpL115, leukosialin, CD43)	binding; cellular defense response; chemotaxis; establishment and/or maintenance of cell polarity; integral to plasma membrane; negative regulation of cell adhesion; signal transduction; transmembrane receptor activity	[SUMMARY:] Sialophorin (leukosialin) is a major sialoglycoprotein on the surface of human T lymphocytes, monocytes, granulocytes, and some B lymphocytes, which appears to be important for immune function and may be part of a physiologic ligand-receptor complex involved in T-cell activation.[supplied by OMIM]
216981_x_at		SPN	6693	182160	sialophorin (gpL115, leukosialin, CD43)	binding; cellular defense response; chemotaxis; establishment and/or maintenance of cell polarity; integral to plasma membrane; negative regulation of cell adhesion; signal transduction; transmembrane receptor activity	[SUMMARY:] Sialophorin (leukosialin) is a major sialoglycoprotein on the surface of human T lymphocytes, monocytes, granulocytes, and some B lymphocytes, which appears to be important for immune function and may be part of a physiologic ligand-receptor complex involved in T-cell activation.[supplied by OMIM]
209845_at		MKRN1	23608	607754	makorin, ring finger protein, 1	biological_process unknown; cellular_component unknown; molecular_function unknown; nucleic acid binding	[SUMMARY:] The Makorin ring finger protein-1 gene (MKRN1) is a highly transcribed, intron-containing source for a family of intronless mammalian genes encoding a novel class of zinc finger proteins. Phylogenetic analyses indicate that the MKRN1 gene is the ancestral founder of this gene family.[supplied by OMIM]

71933_at		WNT6	7475	604663	wingless-type MMTV integration site family, member 6	cell-cell signaling; development; extracellular; extracellular matrix structural constituent; frizzled-2 signaling pathway; signal transducer activity	[SUMMARY:] The WNT gene family consists of structurally related genes which encode secreted signaling proteins. These proteins have been implicated in oncogenesis and in several developmental processes, including regulation of cell fate and patterning during embryogenesis. This gene is a member of the WNT gene family. It is overexpressed in cervical cancer cell line and strongly coexpressed with another family member, WNT10A, in colorectal cancer cell line. The gene overexpression may play key roles in carcinogenesis. This gene and the WNT10A gene are clustered in the chromosome 2q35 region. The protein encoded by this gene is 97% identical to the mouse Wnt6 protein at the amino acid level.
206586_at		CNR2	1269	605051	cannabinoid receptor 2 (macrophage)	G-protein signaling, coupled to cyclic nucleotide second messenger; behavior; cannabinoid receptor activity; immune response; integral to plasma membrane	[SUMMARY:] The cannabinoid delta-9-tetrahydrocannabinol is the principal psychoactive ingredient of marijuana. The proteins encoded by this gene and the cannabinoid receptor 1 (brain) (CNR1) gene have the characteristics of a guanine nucleotide-binding protein (G-protein)-coupled receptor for cannabinoids. They inhibit adenylate cyclase activity in a dose-dependent, stereoselective, and pertussis toxin-sensitive manner. These proteins have been found to be involved in the cannabinoid-induced CNS effects (including alterations in mood and cognition) experienced by users of marijuana. The cannabinoid receptors are members of family 1 of the G-protein-coupled receptors.
396_f_at		EPOR	2057	133171	erythropoietin receptor	erythropoietin receptor activity; guanyl-nucleotide exchange factor activity; integral to plasma membrane; intracellular signaling cascade; neuropeptide signaling pathway; signal transduction	[SUMMARY:] The erythropoietin receptor is a member of the cytokine receptor family. Upon erythropoietin binding, the erythropoietin receptor activates Jak2 tyrosine kinase which activates different intracellular pathways including: Ras/MAP kinase, phosphatidylinositol 3-kinase and STAT transcription factors. The stimulated erythropoietin receptor appears to have a role in erythroid cell survival. Defects in the erythropoietin receptor may produce erythroleukemia and familial erythrocytosis.

207524_at		ST7	7982	600833	suppression of tumorigenicity 7		[SUMMARY:] The gene for this product maps to a region on chromosome 7 identified as an autism-susceptibility locus. Mutation screening of the entire coding region in autistic individuals failed to identify phenotype-specific variants, suggesting that coding mutations for this gene are unlikely to be involved in the etiology of autism. The function of this gene product has not been determined. Transcript variants encoding different isoforms of this protein have been described.
204179_at		MB	4151	160000	myoglobin		[SUMMARY:] The human myoglobin gene is 10.4 kb long and has a three exon/two intron structure with long non-coding regions. It encodes the protein myoglobin, which is a haemoprotein contributing to intracellular oxygen storage and transcellular facilitated diffusion of oxygen. Myoglobin is a member of the globin superfamily and present in skeletal and cardiac muscle. At least three alternatively spliced transcript variants encoding the same protein have been reported.
215582_x_at		MCM3AP	8888	603294	MCM3 minichromosome maintenance deficient 3 (S. cerevisiae) associated protein	DNA binding; DNA replication; nucleus; protein-nucleus import	[SUMMARY:] The minichromosome maintenance protein 3 (MCM3) is one of the MCM proteins essential for the initiation of DNA replication. The protein encoded by this gene is a MCM3 binding protein. It was reported to have phosphorylation-dependent DNA-primase activity, which was up-regulated in antigen immunization induced germinal center. This protein was demonstrated to be an acetyltransferase that acetylates MCM3 and plays a role in DNA replication. The mutagenesis of a nuclear localization signal of MCM3 affects the binding of this protein with MCM3, suggesting that this protein may also facilitate MCM3 nuclear localization.

202513_s_at		PPP2R5D	5528	601646	protein phosphatase 2, regulatory subunit B (B56), delta isoform	neurogenesis; nucleus; protein phosphatase type 2A complex; protein phosphatase type 2A regulator activity; signal transduction	[SUMMARY:] The product of this gene belongs to the phosphatase 2A regulatory subunit B family. Protein phosphatase 2A is one of the four major Ser/Thr phosphatases, and it is implicated in the negative control of cell growth and division. It consists of a common heteromeric core enzyme, which is composed of a catalytic subunit and a constant regulatory subunit, that associates with a variety of regulatory subunits. The B regulatory subunit might modulate substrate selectivity and catalytic activity. This gene encodes a delta isoform of the regulatory subunit B56 subfamily. Alternatively spliced transcript variants encoding different isoforms have been identified.
201404_x_at		PSMB2	5690	602175	proteasome (prosome, macropain) subunit, beta type, 2	endopeptidase activity; proteasome core complex (sensu Eukarya); ubiquitin-dependent protein catabolism	[SUMMARY:] The proteasome is a multicatalytic proteinase complex with a highly ordered ring-shaped 20S core structure. The core structure is composed of 4 rings of 28 non-identical subunits; 2 rings are composed of 7 alpha subunits and 2 rings are composed of 7 beta subunits. Proteasomes are distributed throughout eukaryotic cells at a high concentration and cleave peptides in an ATP/ubiquitin-dependent process in a non-lysosomal pathway. An essential function of a modified proteasome, the immunoproteasome, is the processing of class I MHC peptides. This gene encodes a member of the proteasome B-type family, also known as the T1B family, that is a 20S core beta subunit.
215511_at		TCF20	6942	603107	transcription factor 20 (AR1)	DNA binding; nucleus; regulation of transcription, DNA-dependent; transcription coactivator activity	[SUMMARY:] The protein encoded by this gene binds a platelet-derived growth factor-responsive element in the matrix metalloproteinase 3 (stromelysin 1) promoter. The protein localizes to the nucleus and displays DNA-binding and transactivation activities. It is thought to be a transcriptional coactivator, enhancing the activity of transcription factors such as JUN and SP1. Alternative splicing results in two transcript variants encoding different isoforms.
216153_x_at		RECK	8434	605227	reversion-inducing-cysteine-rich protein with kazal motifs	membrane; membrane fraction; metalloendopeptidase inhibitor activity; negative regulation of cell cycle; serine-type endopeptidase inhibitor activity	[SUMMARY:] The protein encoded by this gene is a cysteine-rich, extracellular protein with protease inhibitor-like domains whose expression is suppressed strongly in many tumors and cells transformed by various kinds of oncogenes. In normal cells, this membrane-anchored glycoprotein may serve as a negative regulator for matrix metalloproteinase-9, a key enzyme involved in tumor invasion and metastasis.

204810_s_at		CKM	1158	123310	creatine kinase, muscle	creatine kinase activity; transferase activity, transferring phosphorus-containing groups	[SUMMARY:] The protein encoded by this gene is a cytoplasmic enzyme involved in energy homeostasis and is an important serum marker for myocardial infarction. The encoded protein reversibly catalyzes the transfer of phosphate between ATP and various phosphogens such as creatine phosphate. It acts as a homodimer in striated muscle as well as in other tissues, and as a heterodimer with a similar brain isozyme in heart. The encoded protein is a member of the ATP:guanido phosphotransferase protein family.
38707_r_at		E2F4	1874	600659	E2F transcription factor 4, p107/p130-binding		[SUMMARY:] The protein encoded by this gene is a member of the E2F family of transcription factors. The E2F family plays a crucial role in the control of cell cycle and action of tumor suppressor proteins and is also a target of the transforming proteins of small DNA tumor viruses. The E2F proteins contain several evolutionally conserved domains found in most members of the family. These domains include a DNA binding domain, a dimerization domain which determines interaction with the differentiation regulated transcription factor proteins (DP), a transactivation domain enriched in acidic amino acids, and a tumor suppressor protein association domain which is embedded within the transactivation domain. This protein binds to all three of the tumor suppressor proteins pRB, p107 and p130, but with higher affinity to the last two. It plays an important role in the suppression of proliferation-associated genes, and its gene mutation and increased expression may be associated with human cancer.

35150_at		TNFRSF5	958	109535	tumor necrosis factor receptor superfamily, member 5	B-cell proliferation; antimicrobial humoral response (sensu Vertebrata); apoptosis; development; immune response; inflammatory response; integral to plasma membrane; platelet activation; protein complex assembly; signal transduction; transmembrane receptor activity	[SUMMARY:] The protein encoded by this gene is a member of the TNF-receptor superfamily. This receptor has been found to be essential in mediating a broad variety of immune and inflammatory responses including T cell-dependent immunoglobulin class switching, memory B cell development, and germinal center formation. AT-hook transcription factor AKNA is reported to coordinately regulate the expression of this receptor and its ligand, which may be important for homotypic cell interactions. Adaptor protein TNFR2 interacts with this receptor and serves as a mediator of the signal transduction. The interaction of this receptor and its ligand is found to be necessary for amyloid-beta-induced microglial activation, and thus is thought to be an early event in Alzheimer disease pathogenesis. Two alternatively spliced transcript variants of this gene encoding distinct isoforms have been reported.
207908_at		KRT2A	3849	600194	keratin 2A (epidermal ichthyosis bullosa of Siemens)	epidermal differentiation; intermediate filament; structural constituent of cytoskeleton	[SUMMARY:] The protein encoded by this gene is a member of the keratin gene family. The type II cytokeratins consist of basic or neutral proteins which are arranged in pairs of heterotypic keratin chains coexpressed during differentiation of simple and stratified epithelial tissues. This type II cytokeratin is expressed largely in the upper spinous layer of epidermal keratinocytes and mutations in this gene have been associated with bullous congenital ichthyosiform erythroderma. The type II cytokeratins are clustered in a region of chromosome 12q12-q13.
208384_s_at		MID2	11043	300204	midline 2		[SUMMARY:] The protein encoded by this gene is a member of the tripartite motif (TRIM) family. The TRIM motif includes three zinc-binding domains, a RING, a B-box type 1 and a B-box type 2, and a coiled-coil region. The protein localizes to microtubular structures in the cytoplasm. Its function has not been identified. Alternate splicing of this gene results in two transcript variants encoding different isoforms.

221627_at		TRIM10	10107	605701	tripartite motif-containing 10	hemopoiesis; intracellular; zinc ion binding	[SUMMARY:] The protein encoded by this gene is a member of the tripartite motif (TRIM) family. The TRIM motif includes three zinc-binding domains, a RING, a B-box type 1 and a B-box type 2, and a coiled-coil region. This protein localizes to cytoplasmic bodies. Studies in mice suggest that this protein plays a role in terminal differentiation of erythroid cells. Alternate splicing of this gene generates two transcript variants encoding different isoforms.
212184_s_at		MAP3K7IP2	23118	605101	mitogen-activated protein kinase kinase kinase 7 interacting protein 2	kinase activity	[SUMMARY:] The protein encoded by this gene is an activator of MAP3K7/TAK1, which is required for for the IL-1 induced activation of nuclear factor kappaB and MAPK8/JNK. This protein forms a kinase complex with TRAF6, MAP3K7 and TAB1, thus serves as an adaptor linking MAP3K7 and TRAF6. This protein, TAB1, and MAP3K7 also participate in the signal transduction induced by TNFSF11/RANKl through the activation of the receptor activator of NF-kappB (TNFRSF11A/RANK), which may regulate the development and function of osteoclasts. Two alternatively spliced transcript variants of this gene encoding distinct isoforms have been reported.
210644_s_at		LAIR1	3903	602992	leukocyte-associated Ig-like receptor 1	immune response; integral to plasma membrane; signal transduction; transmembrane receptor activity	[SUMMARY:] The protein encoded by this gene is an inhibitory receptor found on peripheral mononuclear cells, including NK cells, T cells, and B cells. Inhibitory receptors regulate the immune response to prevent lysis of cells recognized as self. The gene is a member of both the immunoglobulin superfamily and the leukocyte-associated inhibitory receptor family. The gene maps to a region of 19q13.4 called the leukocyte receptor cluster, which contains at least 29 genes encoding leukocyte-expressed receptors of the immunoglobulin superfamily.
49878_at		PEX16	9409	603360	peroxisomal biogenesis factor 16	integral to membrane; integral to peroxisomal membrane; peroxisome; peroxisome organization and biogenesis	[SUMMARY:] The protein encoded by this gene is an integral peroxisomal membrane protein. An inactivating nonsense mutation localized to this gene was observed in a patient with Zellweger syndrome of the complementation group CGD/CG9. Expression of this gene product morphologically and biochemically restores the formation of new peroxisomes, suggesting a role in peroxisome organization and biogenesis. Alternative splicing has been observed for this gene and two variants have been described.

202716_at		PTPN1	5770	176885	protein tyrosine phosphatase, non-receptor type 1		[SUMMARY:] The protein encoded by this gene is the founding member of the protein tyrosine phosphatase (PTP) family, which was isolated and identified based on its enzymatic activity and amino acid sequence. PTPs catalyze the hydrolysis of the phosphate monoesters specifically on tyrosine residues. Members of the PTP family share a highly conserved catalytic motif, which is essential for the catalytic activity. PTPs are known to be signaling molecules that regulate a variety of cellular processes including cell growth, differentiation, mitotic cycle, and oncogenic transformation. This PTP has been shown to act as a negative regulator of insulin signaling by dephosphorylating the phosphotryosine residues of insulin receptor kinase. This PTP was also reported to dephosphorylate epidermal growth factor receptor kinase, as well as JAK2 and TYK2 kinases, which implicated the role of this PTP in cell growth control, and cell response to interferon stimulation.
209057_x_at		CDC5L	988	602868	CDC5 cell division cycle 5-like (S. pombe)	DNA binding; cytokinesis; nucleus	[SUMMARY:] The protein encoded by this gene shares a significant similarity with Schizosaccharomyces pombe cdc5 gene product, which is a cell cycle regulator important for G2/M transition. This protein has been demonstrated to act as a positive regulator of cell cycle G2/M progression. It was also found to be an essential component of a non-snRNA spliceosome, which contains at least five additional protein factors and is required for the second catalytic step of pre-mRNA splicing.
215383_x_at		ACP33	51324	608181	acid cluster protein 33	catalytic activity	[SUMMARY:] The protein encoded by this gene was identified by a two-hybrid screen using CD4 as the bait. It binds to the hydrophobic C-terminal amino acids of CD4 which are involved in repression of T cell activation. The interaction with CD4 is mediated by the noncatalytic alpha/beta hydrolase fold domain of this protein. It is thus proposed that this gene product modulates the stimulatory activity of CD4.

205600_x_at		HOXB5	3215	142960	homeo box B5	morphogenesis; nucleus; regulation of transcription, DNA-dependent; transcription factor activity	[SUMMARY:] This gene belongs to the homeobox family of genes. The homeobox genes encode a highly conserved family of transcription factors that play an important role in morphogenesis in all multicellular organisms. Mammals possess four similar homeobox gene clusters, HOXA, HOXB, HOXC and HOXD, located on different chromosomes, consisting of 9 to 11 genes arranged in tandem. This gene is one of several homeobox HOXB genes located in a cluster on chromosome 17. The exact role of this gene has yet to be determined.
201132_at		HNRPH2	3188	601036	heterogeneous nuclear ribonucleoprotein H2 (H')	RNA binding; heterogeneous nuclear ribonucleoprotein complex	[SUMMARY:] This gene belongs to the subfamily of ubiquitously expressed heterogeneous nuclear ribonucleoproteins (hnRNPs). The hnRNPs are RNA binding proteins and they complex with heterogeneous nuclear RNA (hnRNA). These proteins are associated with pre-mRNAs in the nucleus and appear to influence pre-mRNA processing and other aspects of mRNA metabolism and transport. While all of the hnRNPs are present in the nucleus some seem to shuttle between the nucleus and the cytoplasm. The hnRNP proteins have distinct nucleic acid binding properties. The protein encoded by this gene has three repeats of quasi-RRM domains that binds to RNAs. It is very similar to the family member HNRPH1. This gene is thought to be involved in Fabray disease and X-linked agammaglobulinemia phenotype.

202488_s_at		FXYD3	5349	604996	FXYD domain containing ion transport regulator 3		[SUMMARY:] This gene encodes a member of a family of small membrane proteins that share a 35-amino acid signature sequence domain, beginning with the sequence PFXYD and containing 7 invariant and 6 highly conserved amino acids. The approved human gene nomenclature for the family is FXYD-domain containing ion transport regulator. Mouse FXYD5 has been termed RIC (Related to Ion Channel). FXYD2, also known as the gamma subunit of the Na,K-ATPase, regulates the properties of that enzyme. FXYD1 (phospholemman), FXYD2 (gamma), FXYD3 (MAT-8), FXYD4 (CHIF), and FXYD5 (RIC) have been shown to induce channel activity in experimental expression systems. Transmembrane topology has been established for two family members (FXYD1 and FXYD2), with the N-terminus extracellular and the C-terminus on the cytoplasmic side of the membrane. The protein encoded by this gene may function as a chloride channel or as a chloride channel regulator. Two transcript variants encode two different isoforms of the protein; in addition, transcripts utilizing alternative polyA signals have been described in the liter
45572_s_at		GGA1	26088	606004	golgi associated, gamma adaptin ear containing, ARF binding protein 1	Golgi stack; intra-Golgi transport; intracellular protein transport; molecular_function unknown; protein transporter activity	[SUMMARY:] This gene encodes a member of the Golgi-localized, gamma adaptin ear-containing, ARF-binding (GGA) family. This family includes ubiquitous coat proteins that regulate the trafficking of proteins between the trans-Golgi network and the lysosome. These proteins share an amino-terminal VHS domain which mediates sorting of the mannose 6-phosphate receptors at the trans-Golgi network. They also contain a carboxy-terminal region with homology to the ear domain of gamma-adaptins.
50277_at		GGA1	26088	606004	golgi associated, gamma adaptin ear containing, ARF binding protein 1	Golgi stack; intra-Golgi transport; intracellular protein transport; molecular_function unknown; protein transporter activity	[SUMMARY:] This gene encodes a member of the Golgi-localized, gamma adaptin ear-containing, ARF-binding (GGA) family. This family includes ubiquitous coat proteins that regulate the trafficking of proteins between the trans-Golgi network and the lysosome. These proteins share an amino-terminal VHS domain which mediates sorting of the mannose 6-phosphate receptors at the trans-Golgi network. They also contain a carboxy-terminal region with homology to the ear domain of gamma-adaptins.

210200_at		WWP2	11060	602308	Nedd-4-like ubiquitin-protein ligase	ligase activity; ubiquitin cycle; ubiquitin ligase complex; ubiquitin-protein ligase activity	[SUMMARY:] This gene encodes a member of the NEDD4-like protein family. The family of proteins is known to possess ubiquitin-protein ligase activity. The encoded protein contains 4 tandem WW domains. The WW domain is a protein motif consisting of 35 to 40 amino acids and is characterized by 4 conserved aromatic residues. The WW domain may mediate specific protein-protein interactions. Three alternatively spliced transcript variants encoding distinct isoforms have been found for this gene.
201723_s_at		GALNT1	2589	602273	UDP-N-acetyl-alpha-D-galactosamine:polypeptide N-acetylgalactosaminyltransferase 1 (GalNAc-T1)	Golgi apparatus; O-linked glycosylation; heterophilic cell adhesion; integral to membrane; manganese ion binding; polypeptide N-acetylgalactosaminyltransferase activity; sugar binding; transferase activity, transferring glycosyl groups	[SUMMARY:] This gene encodes a member of the UDP-N-acetyl-alpha-D-galactosamine:polypeptide N-acetylgalactosaminyltransferase (GalNAc-T) family of enzymes. GalNAc-Ts initiate mucin-type O-linked glycosylation in the Golgi apparatus by catalyzing the transfer of GalNAc to serine and threonine residues on target proteins. They are characterized by an N-terminal transmembrane domain, a stem region, a lumenal catalytic domain containing a GT1 motif and Gal/GalNAc transferase motif, and a C-terminal ricin/lectin-like domain. GalNAc-Ts have different, but overlapping, substrate specificities and patterns of expression. Transcript variants derived from this gene that utilize alternative polyA signals have been described in the literature.
216025_x_at		CYP2C9	1559	601130	cytochrome P450, family 2, subfamily C, polypeptide 9	(S)-limonene 7-monooxygenase activity; electron transport; endoplasmic reticulum; membrane; microsome; monooxygenase activity; unspecific monooxygenase activity	[SUMMARY:] This gene encodes a member of the cytochrome P450 superfamily of enzymes. The cytochrome P450 proteins are monooxygenases which catalyze many reactions involved in drug metabolism and synthesis of cholesterol, steroids and other lipids. This protein localizes to the endoplasmic reticulum and its expression is induced by rifampin. The enzyme is known to metabolize many xenobiotics, including phenytoin, tolbutamide, ibuprofen and S-warfarin. Studies identifying individuals who are poor metabolizers of phenytoin and tolbutamide suggest that this gene is polymorphic. The gene is located within a cluster of cytochrome P450 genes on chromosome 10q24.

207718_x_at		CYP2A7	1549	608054	cytochrome P450, family 2, subfamily A, polypeptide 7	electron transport; endoplasmic reticulum; membrane; microsome; monooxygenase activity; oxygen binding; unspecific monooxygenase activity	[SUMMARY:] This gene encodes a member of the cytochrome P450 superfamily of enzymes. The cytochrome P450 proteins are monooxygenases which catalyze many reactions involved in drug metabolism and synthesis of cholesterol, steroids and other lipids. This protein localizes to the endoplasmic reticulum; its substrate has not yet been determined. This gene, which produces two transcript variants, is part of a large cluster of cytochrome P450 genes from the CYP2A, CYP2B and CYP2F subfamilies on chromosome 19q.
49327_at		SIRT3	23410	604481	sirtuin (silent mating type information regulation 2 homolog) 3 (S. cerevisiae)		[SUMMARY:] This gene encodes a member of the sirtuin family of proteins, homologs to the yeast Sir2 protein. Members of the sirtuin family are characterized by a sirtuin core domain and grouped into four classes. The functions of human sirtuins have not yet been determined; however, yeast sirtuin proteins are known to regulate epigenetic gene silencing and suppress recombination of rDNA. Studies suggest that the human sirtuins may function as intracellular regulatory proteins with mono-ADP-ribosyltransferase activity. The protein encoded by this gene is included in class I of the sirtuin family.
205482_x_at		SNX15	29907	605964	sorting nexin 15	cytosol; intracellular protein transport; intracellular signaling cascade; membrane; protein transporter activity	[SUMMARY:] This gene encodes a member of the sorting nexin family. Members of this family contain a phox (PX) domain, which is a phosphoinositide binding domain, and are involved in intracellular trafficking. Overexpression of this gene results in a decrease in the processing of insulin and hepatocyte growth factor receptors to their mature subunits. This decrease is caused by the mislocalization of furin, the endoprotease responsible for cleavage of insulin and hepatocyte growth factor receptors. This protein is involved in endosomal trafficking from the plasma membrane to recycling endosomes or the trans-Golgi network. This gene encodes two transcript variants encoding distinct isoforms.
207960_at		PAP	23496	167805	pancreatitis-associated protein	acute-phase response; cell growth; cell proliferation; cytoplasm; development; extracellular; extracellular space; growth factor activity; hematopoietin/interferon-class (D200-domain) cytokine receptor activity; heterophilic cell adhesion; inflammatory response; membrane; soluble fraction; sugar binding	[SUMMARY:] This gene encodes a pancreatic secretory protein that may be involved in cell proliferation or differentiation. It has similarity to the C-type lectin superfamily. The enhanced expression of this gene is observed during pancreatic inflammation and liver carcinogenesis. Multiple alternatively spliced transcript variants encoding the same protein have been described for this gene but the full length nature of some transcripts is not yet known.

210686_x_at		SLC25A16	8034	139080	solute carrier family 25 (mitochondrial carrier; Graves disease autoantigen), member 16	binding; integral to membrane; membrane; mitochondrial inner membrane; mitochondrion; solute:solute antiporter activity; solute:solute exchange; transport	[SUMMARY:] This gene encodes a protein that contains three tandemly repeated mitochondrial carrier protein domains. The encoded protein is localized in the inner membrane and facilitates the rapid transport and exchange of molecules between the cytosol and the mitochondrial matrix space. This gene has a possible role in Graves' disease.
36019_at		STK19	8859	604977	serine/threonine kinase 19	ATP binding; manganese ion binding; nucleus; protein amino acid phosphorylation; protein serine/threonine kinase activity; transferase activity	[SUMMARY:] This gene encodes a serine/threonine kinase which localizes predominantly to the nucleus. Its specific function is unknown; it is possible that phosphorylation of this protein is involved in transcriptional regulation. This gene localizes to the major histocompatibility complex (MHC) class III region on chromosome 6 and expresses two transcript variants.
64440_at		IL17RC	84818		interleukin 17 receptor C		[SUMMARY:] This gene encodes a single-pass transmembrane protein that shares limited similarity with the interleukin-17 receptor. At least eleven alternatively spliced transcript variants of this gene have been detected. The full-length nature of five of the variants encoding distinct isoforms have been reported.
206776_x_at		ACRV1	56	102525	acrosomal vesicle protein 1	development	[SUMMARY:] This gene encodes a testis-specific, differentiation antigen, acrosomal vesicle protein 1, that arises within the acrosomal vesicle during spermatogenesis, and is associated with the acrosomal membranes and matrix of mature sperm. This gene consists of 4 exons and its alternative splicing generates 11 distinct transcripts, which encode protein isoforms ranging from 81 to 265 amino acids. The longest transcript is the most abundant, comprising 53-72% of the total acrosomal vesicle protein 1 messages; the second largest transcript comprises 15-32%; the third and the fourth largest transcripts account for 3.4-8.3% and 8.7-12.5%, respectively; and the remaining 7 transcripts combined account for < 1% of the total acrosomal vesicle protein 1 message. It is suggested that phenomena of cryptic splicing and exon skipping occur within this gene. The acrosomal vesicle protein 1 may be involved in sperm-zona binding or penetration, and it is a potential contraceptive vaccine immunogen for humans.

207969_x_at		ACRV1	56	102525	acrosomal vesicle protein 1	development	[SUMMARY:] This gene encodes a testis-specific, differentiation antigen, acrosomal vesicle protein 1, that arises within the acrosomal vesicle during spermatogenesis, and is associated with the acrosomal membranes and matrix of mature sperm. This gene consists of 4 exons and its alternative splicing generates 11 distinct transcripts, which encode protein isoforms ranging from 81 to 265 amino acids. The longest transcript is the most abundant, comprising 53-72% of the total acrosomal vesicle protein 1 messages; the second largest transcript comprises 15-32%; the third and the fourth largest transcripts account for 3.4-8.3% and 8.7-12.5%, respectively; and the remaining 7 transcripts combined account for < 1% of the total acrosomal vesicle protein 1 message. It is suggested that phenomena of cryptic splicing and exon skipping occur within this gene. The acrosomal vesicle protein 1 may be involved in sperm-zona binding or penetration, and it is a potential contraceptive vaccine immunogen for humans.
207973_x_at		ACRV1	56	102525	acrosomal vesicle protein 1	development	[SUMMARY:] This gene encodes a testis-specific, differentiation antigen, acrosomal vesicle protein 1, that arises within the acrosomal vesicle during spermatogenesis, and is associated with the acrosomal membranes and matrix of mature sperm. This gene consists of 4 exons and its alternative splicing generates 11 distinct transcripts, which encode protein isoforms ranging from 81 to 265 amino acids. The longest transcript is the most abundant, comprising 53-72% of the total acrosomal vesicle protein 1 messages; the second largest transcript comprises 15-32%; the third and the fourth largest transcripts account for 3.4-8.3% and 8.7-12.5%, respectively; and the remaining 7 transcripts combined account for < 1% of the total acrosomal vesicle protein 1 message. It is suggested that phenomena of cryptic splicing and exon skipping occur within this gene. The acrosomal vesicle protein 1 may be involved in sperm-zona binding or penetration, and it is a potential contraceptive vaccine immunogen for humans.

217301_x_at		RBBP4	5928	602923	retinoblastoma binding protein 4		[SUMMARY:] This gene encodes a ubiquitously expressed nuclear protein which belongs to a highly conserved subfamily of WD-repeat proteins. It is present in protein complexes involved in histone acetylation and chromatin assembly. It is part of the Mi-2 complex which has been implicated in chromatin remodeling and transcriptional repression associated with histone deacetylation. This encoded protein is also part of co-repressor complexes, which is an integral component of transcriptional silencing. It is found among several cellular proteins that bind directly to retinoblastoma protein to regulate cell proliferation. This protein also seems to be involved in transcriptional repression of E2F-responsive genes.
222187_x_at		G3BP	10146	608431	Ras-GTPase-activating protein SH3-domain-binding protein	ATP-dependent DNA helicase activity; ATP-dependent RNA helicase activity; RAS protein signal transduction; RNA binding; cytoplasm; nucleus; protein binding; protein transporter activity; protein-nucleus import; transport	[SUMMARY:] This gene encodes one of the DNA-unwinding enzymes which prefers partially unwound 3'-tailed substrates and can also unwind partial RNA/DNA and RNA/RNA duplexes in an ATP-dependent fashion. This enzyme is a member of the heterogeneous nuclear RNA-binding proteins and is also an element of the Ras signal transduction pathway. It binds specifically to the Ras-GTPase-activating protein by associating with its SH3 domain. Several alternatively spliced transcript variants of this gene have been described, but the full-length nature of some of these variants has not been determined.
216554_s_at		ENO1	2023	172430	enolase 1, (alpha)	DNA binding; glycolysis; lyase activity; magnesium ion binding; negative regulation of cell growth; negative regulation of transcription from Pol II promoter; nucleus; phosphopyruvate hydratase activity; phosphopyruvate hydratase complex; protein binding; transcription corepressor activity; transcription factor activity; transcriptional repressor activity	[SUMMARY:] This gene encodes one of three enolase isoenzymes found in mammals; it encodes alpha-enolase, a homodimeric soluble enzyme, and also encodes a shorter monomeric structural lens protein, tau-crystallin. The two proteins are made from the same message. The full length protein, the isoenzyme, is found in the cytoplasm. The shorter protein is produced from an alternative translation start, is localized to the nucleus, and has been found to bind to an element in the c-myc promoter. A pseudogene has been identified that is located on the other arm of the same chromosome.

36829_at		PER1	5187	602260	period homolog 1 (Drosophila)	entrainment of circadian clock; nucleus; regulation of transcription, DNA-dependent; signal transducer activity; signal transduction	[SUMMARY:] This gene is a member of the Period family of genes and is expressed in a circadian pattern in the suprachiasmatic nucleus, the primary circadian pacemaker in the mammalian brain. Genes in this family encode components of the circadian rhythms of locomotor activity, metabolism, and behavior. Circadian expression in the suprachiasmatic nucleus continues in constant darkness, and a shift in the light/dark cycle evokes a proportional shift of gene expression in the suprachiasmatic nucleus. The specific function of this gene is not yet known. Alternative splicing has been observed in this gene; however, these variants have not been fully described.
121_at		PAX8	7849	167415	paired box gene 8	biological_process unknown; cell differentiation; molecular_function unknown; morphogenesis; nucleus; regulation of transcription, DNA-dependent; thyroid-stimulating hormone receptor activity; transcription factor activity	[SUMMARY:] This gene is a member of the paired box (PAX) family of transcription factors. Members of this gene family typically contain a paired box domain, an octapeptide, and a paired-type homeodomain. These genes play critical roles during fetal development and cancer growth. The specific function of the paired box gene 8 is unknown but it may involve kidney cell differentiation, thyroid development, or thyroid dysgenesis. Alternative splicing in this gene by inclusion or exclusion of exons 7 and/or 8 has produced several known products but the biological significance of the variants is unknown. Several other splice variants have been proposed but the full nature of these products has not been described.
203328_x_at		IDE	3416	146680	insulin-degrading enzyme		[SUMMARY:] This gene may belong to a protease family responsible for intercellular peptide signalling. Though its role in the cellular processing of insulin has not yet been defined, insulin-degrading enzyme is thought to be involved in the termination of the insulin response.
203187_at		DOCK1	1793	601403	dedicator of cytokinesis 1	GTPase activator activity; apoptosis; cytoskeleton; integrin-mediated signaling pathway; membrane; phagocytosis, engulfment; signal transduction; small GTPase mediated signal transduction	[SUMMARY:] This gene product binds to the SH3 domain of CRK protein. It may regulate cell surface extension and may have a role in the cell surface extension of an engulfing cell around a dying cell during apoptosis.

200966_x_at		ALDOA	226	103850	aldolase A, fructose-bisphosphate	fructose metabolism; fructose-bisphosphate aldolase activity; glycolysis; lyase activity; striated muscle contraction	[SUMMARY:] This gene product, Aldolase A (fructose-bisphosphate aldolase) is a glycolytic enzyme that catalyzes the reversible conversion of fructose-1,6-bisphosphate to glyceraldehyde 3-phosphate and dihydroxyacetone phosphate. Three aldolase isozymes (A, B, and C), encoded by three different genes, are differentially expressed during development. Aldolase A is found in the developing embryo and is produced in even greater amounts in adult muscle. Aldolase A expression is repressed in adult liver, kidney and intestine and similar to aldolase C levels in brain and other nervous tissue. Aldolase A deficiency has been associated with myopathy and hemolytic anemia. Alternative splicing of this gene results in multiple transcript variants which encode the same protein.
214687_x_at		ALDOA	226	103850	aldolase A, fructose-bisphosphate	fructose metabolism; fructose-bisphosphate aldolase activity; glycolysis; lyase activity; striated muscle contraction	[SUMMARY:] This gene product, Aldolase A (fructose-bisphosphate aldolase) is a glycolytic enzyme that catalyzes the reversible conversion of fructose-1,6-bisphosphate to glyceraldehyde 3-phosphate and dihydroxyacetone phosphate. Three aldolase isozymes (A, B, and C), encoded by three different genes, are differentially expressed during development. Aldolase A is found in the developing embryo and is produced in even greater amounts in adult muscle. Aldolase A expression is repressed in adult liver, kidney and intestine and similar to aldolase C levels in brain and other nervous tissue. Aldolase A deficiency has been associated with myopathy and hemolytic anemia. Alternative splicing of this gene results in multiple transcript variants which encode the same protein.

207608_x_at		CYP1A2	1544	124060	cytochrome P450, family 1, subfamily A, polypeptide 2		[SUMMARY:] This gene, CYP1A2, encodes a member of the cytochrome P450 superfamily of enzymes. The cytochrome P450 proteins are monooxygenases which catalyze many reactions involved in drug metabolism and synthesis of cholesterol, steroids and other lipids. The protein encoded by this gene localizes to the endoplasmic reticulum and its expression is induced by some polycyclic aromatic hydrocarbons (PAHs), some of which are found in cigarette smoke. The enzyme's endogenous substrate is unknown; however, it is able to metabolize some PAHs to carcinogenic intermediates. Other xenobiotic substrates for this enzyme include caffeine, aflatoxin B1, and acetaminophen. The transcript from this gene contains four Alu sequences flanked by direct repeats in the 3' untranslated region. A related family member, CYP1A1, is located approximately 25 kb away from CYP1A2 on chromosome 15.
211295_x_at		CYP2A6	1548	122720	cytochrome P450, family 2, subfamily A, polypeptide 6	coumarin 7-hydroxylase activity; electron transport; endoplasmic reticulum; membrane; microsome; oxygen binding; unspecific monooxygenase activity	[SUMMARY:] This gene, CYP2A6, encodes a member of the cytochrome P450 superfamily of enzymes. The cytochrome P450 proteins are monooxygenases which catalyze many reactions involved in drug metabolism and synthesis of cholesterol, steroids and other lipids. This protein localizes to the endoplasmic reticulum and its expression is induced by phenobarbital. The enzyme is known to hydroxylate coumarin, and also metabolizes nicotine, aflatoxin B1, nitrosamines, and some pharmaceuticals. Individuals with certain allelic variants are said to have a poor metaboliser phenotype, meaning they do not efficiently metabolize coumarin or nicotine. This gene is part of a large cluster of cytochrome P450 genes from the CYP2A, CYP2B and CYP2F subfamilies on chromosome 19q. The gene was formerly referred to as CYP2A3; however, it has been renamed CYP2A6.

203608_at		ALDH5A1	7915	271980	aldehyde dehydrogenase 5 family, member A1 (succinate-semialdehyde dehydrogenase)	aminobutyrate catabolism; electron transporter activity; metabolism; mitochondrion; oxidoreductase activity; succinate-semialdehyde dehydrogenase activity	[SUMMARY:] This protein belongs to the aldehyde dehydrogenase family of proteins. This gene encodes a mitochondrial NAD(+)-dependent succinic semialdehyde dehydrogenase. A deficiency of this enzyme, known as 4-hydroxybutyricaciduria, is a rare inborn error in the metabolism of the neurotransmitter 4-aminobutyric acid (GABA). In response to the defect, physiologic fluids from patients accumulate GHB, a compound with numerous neuromodulatory properties. Two transcript variants encoding distinct isoforms have been identified for this gene.
206212_at		CPA2	1358	600688	carboxypeptidase A2 (pancreatic)	carboxypeptidase A activity; carboxypeptidase activity; hydrolase activity; metallopeptidase activity; proteolysis and peptidolysis; vacuolar protein catabolism	[SUMMARY:] Three different forms of human pancreatic procarboxypeptidase A have been isolated. The A1 and A2 forms are monomeric proteins with different biochemical properties. The A2 form of pancreatic procarboxypeptidase acts on aromatic C-terminal residues
205016_at		TGFA	7039	190170	transforming growth factor, alpha	cell proliferation; cell-cell signaling; epidermal growth factor receptor activating ligand activity; extracellular space; growth factor activity; integral to plasma membrane; protein binding; protein-tyrosine kinase activity; regulation of cell cycle; signal transducer activity; soluble fraction	[SUMMARY:] Transforming growth factors (TGFs) are biologically active polypeptides that reversibly confer the transformed phenotype on cultured cells. Alpha-TGF shows about 40% sequence homology with epidermal growth factor (EGF; MIM 131530) and competes with EGF for binding to the EGF receptor (MIM 131550), stimulating its phosphorylation and producing a mitogenic response.[supplied by OMIM]
205388_at		TNNC2	7125	191039	troponin C2, fast		[SUMMARY:] Troponin (Tn), a key protein complex in the regulation of striated muscle contraction, is composed of 3 subunits. The Tn-I subunit inhibits actomyosin ATPase, the Tn-T subunit binds tropomyosin and Tn-C, while the Tn-C subunit binds calcium and overcomes the inhibitory action of the troponin complex on actin filaments. The protein encoded by this gene is the Tn-C subunit.

210695_s_at		WWOX	51741	605131	WW domain containing oxidoreductase	biological_process unknown; cellular_component unknown; electron transporter activity; metabolism; oxidoreductase activity; protein binding; steroid metabolism	[SUMMARY:] WW domain-containing proteins are found in all eukaryotes and play an important role in the regulation of a wide variety of cellular functions such as protein degradation, transcription, and RNA splicing. This gene encodes a protein which contains 2 WW domains and a short-chain dehydrogenase/reductase domain (SRD). The highest normal expression of this gene is detected in hormonally regulated tissues such as testis, ovary, and prostate. This expression pattern and the presence of an SRD domain suggest a role for this gene in steroid metabolism. The encoded protein is more than 90% identical to the mouse protein, which is an essential mediator of tumor necrosis factor-alpha-induced apoptosis, suggesting a similar, important role in apoptosis for the human protein. In addition, there is evidence that this gene behaves as a suppresor of tumor growth. Alternative splicing of this gene generates 7 transcript variants, which encode different isoforms.
205660_at		OASL	8638	603281	2'-5'-oligoadenylate synthetase-like	ATP binding; DNA binding; biological_process unknown; cytoplasm; double-stranded RNA binding; immune response; nucleolus; thyroid hormone receptor binding; transferase activity	
31861_at		IGHMBP2	3508	600502	immunoglobulin mu binding protein 2	ATP binding; DNA helicase activity; DNA recombination; DNA repair; DNA replication; hydrolase activity; nucleus; regulation of transcription, DNA-dependent; single-stranded DNA binding	
204370_at		HEAB	10978		ATP/GTP-binding protein	ATP binding; GTP binding; mRNA processing; nucleus	
32811_at		MYO1C	4641	606538	myosin IC	ATP binding; actin binding; calmodulin binding; detection of sound; motor activity; unconventional myosin	
217033_x_at		NTRK3	4916	191316	neurotrophic tyrosine kinase, receptor, type 3	ATP binding; integral to plasma membrane; kinase activity; neurogenesis; neurotrophin TRKC receptor activity; protein amino acid phosphorylation; receptor activity; transferase activity; transmembrane receptor protein tyrosine kinase activity; transmembrane receptor protein tyrosine kinase signaling pathway	
52169_at		LYK5	92335		protein kinase LYK5	ATP binding; protein amino acid phosphorylation; protein kinase activity; transferase activity	

211919_s_at		CXCR4	7852	162643	chemokine (C-X-C motif) receptor 4	C-C chemokine receptor activity; C-X-C chemokine receptor activity; G-protein coupled receptor protein signaling pathway; activation of MAPK; apoptosis; chemotaxis; coreceptor activity; cytoplasm; cytosolic calcium ion concentration elevation; immune response; inflammatory response; integral to plasma membrane; neurogenesis; response to virus; rhodopsin-like receptor activity	
220195_at		MBD5	55777		methyl-CpG binding domain protein 5	DNA binding	
201160_s_at		CSDA	8531	603437	cold shock domain protein A	DNA binding; RNA polymerase II transcription factor activity; cytoplasm; double-stranded DNA binding; negative regulation of transcription from Pol II promoter; perinuclear space; regulation of transcription, DNA-dependent; response to cold; transcription corepressor activity; transcription factor activity	
210573_s_at		RPC62	10623		polymerase (RNA) III (DNA directed) (62kD)	DNA-directed RNA polymerase III complex; DNA-directed RNA polymerase activity; regulation of transcription from Pol III promoter	
207554_x_at		TBXA2R	6915	188070	thromboxane A2 receptor	G-protein coupled receptor protein signaling pathway; integral to plasma membrane; muscle contraction; respiratory gaseous exchange; rhodopsin-like receptor activity; thromboxane A2 receptor activity	
213835_x_at		GTPBP3	84705		GTP binding protein 3 (mitochondrial)	GTPase activity; tRNA modification	
220944_at		PGLYRPIbeta	57115	608198	peptidoglycan recognition protein-I-beta precursor	N-acetylmuramoyl-L-alanine amidase activity; defense response to Gram-positive bacteria; detection of bacteria; innate immune response; intracellular; membrane; peptidoglycan binding; peptidoglycan catabolism; peptidoglycan recognition activity	
221817_at		DOLPP1	57171		dolichyl pyrophosphate phosphatase 1	N-linked glycosylation; biological_process unknown; cellular_component unknown; dolichyldiphosphatase activity; endoplasmic reticulum; hydrolase activity; integral to endoplasmic reticulum membrane; molecular_function unknown	
206936_x_at		NDUFC2	4718	603845	NADH dehydrogenase (ubiquinone) 1, subcomplex unknown, 2, 14.5kDa	NADH dehydrogenase (ubiquinone) activity; NADH dehydrogenase activity; membrane fraction; mitochondrion; oxidoreductase activity	
36004_at		IKBKG	8517	300248	inhibitor of kappa light polypeptide gene enhancer in B-cells, kinase gamma	NIK-I-kappaB/NF-kappaB cascade; immune response; induction of apoptosis; nucleus; regulation of transcription, DNA-dependent; signal transducer activity	
212295_s_at		SLC7A1	6541	104615	solute carrier family 7 (cationic amino acid transporter, y+ system), member 1	amino acid metabolism; amino acid permease activity; amino acid transport; basic amino acid transporter activity; integral to plasma membrane; receptor activity; transport	

204404_at		SLC12A2	6558	600840	solute carrier family 12 (sodium/potassium/chloride transporters), member 2	amino acid transport; amino acid-polyamine transporter activity; chloride transport; integral to plasma membrane; ion transport; membrane fraction; potassium ion transport; sodium ion transport; sodium:chloride/potassium:chloride symporter activity; symporter activity; transporter activity	
211316_x_at		CFLAR	8837	603599	CASP8 and FADD-like apoptosis regulator	anti-apoptosis; caspase activity; induction of apoptosis by extracellular signals; molecular_function unknown; protein binding; proteolysis and peptidolysis; regulation of apoptosis	
220048_at		EDAR	10913	604095	ectodysplasin 1, anhidrotic receptor	apoptosis; biological_process unknown; cell differentiation; integral to membrane; signal transduction; transmembrane receptor activity	
208014_x_at		AD7C-NTP	27308	607413	neuronal thread protein	apoptosis; central nervous system development; extracellular space; integral to membrane	
210524_x_at		MT1F	4494	156352	metallothionein 1F (functional)	biological_process unknown; cadmium ion binding; copper ion binding; cytoplasm; metal ion binding; zinc ion binding	
217473_x_at		CTDSP1	58190	605323	CTD (carboxy-terminal domain, RNA polymerase II, polypeptide A) small phosphatase 1	biological_process unknown; hydrolase activity; molecular_function unknown; nucleus; phosphoprotein phosphatase activity	
219746_at		DPF3	8110	601672	D4, zinc and double PHD fingers, family 3	biological_process unknown; nucleus; regulation of transcription, DNA-dependent; zinc ion binding	
204613_at		PLCG2	5336	600220	phospholipase C, gamma 2 (phosphatidylinositol-specific)	calcium ion binding; cell surface receptor linked signal transduction; hydrolase activity; intracellular signaling cascade; lipid catabolism; phosphoinositide phospholipase C activity; phospholipid metabolism; signal transducer activity	
56197_at		PLSCR3	57048	607611	phospholipid scramblase 3	calcium ion binding; integral to membrane; phospholipid scramblase activity; phospholipid scrambling; plasma membrane	
214080_x_at		PRKCSH	5589	177060	protein kinase C substrate 80K-H	calcium ion binding; intracellular; molecular_function unknown; protein kinase cascade	
205163_at		HUMMLC2B	29895		myosin light chain 2	calcium ion binding; muscle myosin; myosin; structural constituent of muscle	
65635_at		FLJ21865	64772		endo-beta-N-acetylglucosaminidase	carbohydrate metabolism; hydrolase activity, acting on glycosyl bonds; intracellular	
205832_at		CPA4	51200	607635	carboxypeptidase A4	carboxypeptidase A activity; carboxypeptidase activity; cellular_component unknown; histone acetylation; hydrolase activity; metallopeptidase activity; proteolysis and peptidolysis	
216606_x_at		LYPLA2L	80734		lysophospholipase 2 like	catalytic activity	

210647_x_at		PLA2G6	8398	603604	phospholipase A2, group VI (cytosolic, calcium-independent)	catalytic activity; cytoplasm; hydrolase activity; lipid catabolism; membrane; nutrient reservoir activity; phospholipase A2 activity; phospholipid metabolism	
48030_i_at		C5orf4	10826		chromosome 5 open reading frame 4	catalytic activity; metabolism	
49077_at		PME-1	51400		protein phosphatase methylesterase-1	catalytic activity; protein amino acid demethylation; protein phosphatase inhibitor activity	
219175_s_at		SLC41A3	54946		solute carrier family 41, member 3	cation transport; cation transporter activity	
91920_at		BCAN	63827		chondroitin sulfate proteoglycan BEHAB	cell adhesion; hyaluronic acid binding; sugar binding	
201540_at		FHL1	2273	300163	four and a half LIM domains 1	cell differentiation; cell growth; cellular_component unknown; molecular_function unknown; muscle development	
213332_at		PLAC3	60676		placenta-specific 3	cell differentiation; intracellular; membrane; metallopeptidase activity; proteolysis and peptidolysis; regulation of cell growth; zinc ion binding	
211956_s_at		SUI1	10209		putative translation initiation factor	cell growth and/or maintenance; cellular_component unknown; cytoplasm; regulation of protein biosynthesis; regulation of translation; regulation of translational initiation; response to stress; translation initiation factor activity; translational initiation	
209124_at		MYD88	4615	602170	myeloid differentiation primary response gene (88)	cell surface receptor linked signal transduction; death receptor binding; immune response; inflammatory response; membrane; transmembrane receptor activity	
33768_at		DMWD	1762		dystrophia myotonica-containing WD repeat motif	cellular_component unknown; meiosis; molecular_function unknown	
207783_x_at		TPT1	7178	600763	tumor protein, translationally-controlled 1	cytoplasm; extracellular space; molecular_function unknown	
211943_x_at		TPT1	7178	600763	tumor protein, translationally-controlled 1	cytoplasm; extracellular space; molecular_function unknown	
212284_x_at		TPT1	7178	600763	tumor protein, translationally-controlled 1	cytoplasm; extracellular space; molecular_function unknown	
212869_x_at		TPT1	7178	600763	tumor protein, translationally-controlled 1	cytoplasm; extracellular space; molecular_function unknown	
214327_x_at		TPT1	7178	600763	tumor protein, translationally-controlled 1	cytoplasm; extracellular space; molecular_function unknown	
216520_s_at		TPT1	7178	600763	tumor protein, translationally-controlled 1	cytoplasm; extracellular space; molecular_function unknown	
219590_x_at		CGI-30	51611		CGI-30 protein	diphthine synthase activity; metabolism; methyltransferase activity; peptidyl-diphthamide biosynthesis from peptidyl-histidine; transferase activity	
206861_s_at		CGGBP1	8545	603363	CGG triplet repeat binding protein 1	double-stranded DNA binding; nucleus	
214205_x_at		TXNL2	10539		thioredoxin-like 2	electron transport; electron transporter activity	
220232_at		SCD4	79966	608370	stearoyl-CoA desaturase 4	endoplasmic reticulum; fatty acid biosynthesis; iron ion binding; membrane; oxidoreductase activity; stearoyl-CoA 9-desaturase activity	
202529_at		PRPSAP1	5635	601249	phosphoribosyl pyrophosphate synthetase-associated protein 1	enzyme inhibitor activity; nucleoside metabolism; nucleotide biosynthesis; ribose-phosphate diphosphokinase activity	

205374_at		SLN	6588	602203	sarcolipin	enzyme regulator activity; integral to membrane; sarcoplasmic reticulum; smooth endoplasmic reticulum; transport	
208106_x_at		PSG6	5675	176395	pregnancy specific beta-1-glycoprotein 6	extracellular space; pregnancy	
210288_at		KLRG1	10219	604874	killer cell lectin-like receptor subfamily G, member 1	heterophilic cell adhesion; receptor activity; sugar binding	
210325_at		CD1A	909	188370	CD1A antigen, a polypeptide	immune response; integral to plasma membrane	
210422_x_at		SLC11A1	6556	600266	solute carrier family 11 (proton-coupled divalent metal ion transporters), member 1	integral to plasma membrane; iron ion transport; membrane; membrane fraction; response to bacteria; response to pest/pathogen/parasite; transport; transporter activity	
58367_s_at		FLJ23233	79744		hypothetical protein FLJ23233	intracellular; nucleic acid binding; regulation of transcription, DNA-dependent	
206180_x_at		MGC2474	65988		hypothetical protein MGC2474	intracellular; nucleic acid binding; regulation of transcription, DNA-dependent	
217750_s_at		FLJ13855	65264		hypothetical protein FLJ13855	ligase activity; ubiquitin conjugating enzyme activity; ubiquitin cycle; ubiquitin-protein ligase activity	
51774_s_at		LOC51619	51619		ubiquitin-conjugating enzyme HBUCE1	ligase activity; ubiquitin conjugating enzyme activity; ubiquitin cycle; ubiquitin-protein ligase activity	
58900_at		LOC51619	51619		ubiquitin-conjugating enzyme HBUCE1	ligase activity; ubiquitin conjugating enzyme activity; ubiquitin cycle; ubiquitin-protein ligase activity	
65521_at		LOC51619	51619		ubiquitin-conjugating enzyme HBUCE1	ligase activity; ubiquitin conjugating enzyme activity; ubiquitin cycle; ubiquitin-protein ligase activity	
201339_s_at		SCP2	6342	184755	sterol carrier protein 2	lipid binding; lipid transport; mitochondrion; peroxisome; steroid biosynthesis; sterol carrier activity	
205788_s_at		KIAA0663	9877		KIAA0663 gene product	nucleic acid binding	
211697_x_at		LOC56902	56902		putatative 28 kDa protein	nucleic acid binding	
204334_at		KLF7	8609	604865	Kruppel-like factor 7 (ubiquitous)	nucleus; regulation of transcription from Pol II promoter; transcription coactivator activity; transcription factor activity; zinc ion binding	
207936_x_at		RFPL3	10738	605970	ret finger protein-like 3	protein binding	
218115_at		ASF1B	55723		ASF1 anti-silencing function 1 homolog B (S. cerevisiae)		
212333_at		DKFZP564F0522	25940		DKFZP564F0522 protein		
204218_at		DKFZP564M082	25906		DKFZP564M082 protein		
36129_at		KIAA0397	9905		KIAA0397 gene product		
48612_at		N4BP1	9683		Nedd4 binding protein 1		
38710_at		OTUB1	55611	608337	OTU domain, ubiquitin aldehyde binding 1		
220167_s_at		TP53TG3	24150		TP53TG3 protein		
219071_x_at		LOC51236	51236		brain protein 16		
212801_at		CIT	11113	605629	citron (rho-interacting, serine/threonine kinase 21)		
48659_at		FLJ12438	60672		hypothetical protein FLJ12438		
206438_x_at		FLJ12975	79867		hypothetical protein FLJ12975		
45749_at		FLJ13725	79567		hypothetical protein FLJ13725		
218460_at		FLJ20397	54919		hypothetical protein FLJ20397		
43977_at		FLJ20422	54929		hypothetical protein FLJ20422		
218648_at		FLJ21868	64784		hypothetical protein FLJ21868		
55705_at		MGC16353	91300		hypothetical protein MGC16353		
220251_at		MGC29875	27042		hypothetical protein MGC29875		

216126_at		MGC39821	284440		hypothetical protein MGC39821		
204987_at		ITIH2	3698	146640	inter-alpha (globulin) inhibitor, H2 polypeptide		
212611_at		MPEG1	219972		macrophage expressed gene 1		
217117_x_at		MUC3B	57876	605633	mucin 3B		
207309_at		NOS1	4842	163731	nitric oxide synthase 1 (neuronal)		
219428_s_at		PXMP4	11264		peroxisomal membrane protein 4, 24kDa		
212004_at		DKFZp566C0424	26099		putative MAPK activating protein PM20,PM21		
206520_x_at		SIGLEC6	946	604405	sialic acid binding Ig-like lectin 6		
79005_at		SLC35E1	79939		solute carrier family 35, member E1		
205103_at		CROC4	10485		transcriptional activator of the c-fos promoter		
218631_at		VIP32	60370		vasopressin-induced transcript		
211454_x_at							
213893_x_at							
215825_at							
215883_at							
216366_x_at							
216739_at							
217393_x_at							
217541_x_at							
217580_x_at							
217637_at							
222329_x_at							
AFFX-hum_alu_at							

Record Count = 221 It took 2 seconds to process this page
